# Tissue Engineering and Regenerative Medicine: Perspectives and Challenges

**DOI:** 10.1002/mco2.70192

**Published:** 2025-04-24

**Authors:** Van T. Hoang, Quyen Thi Nguyen, Trang Thi Kieu Phan, Trang H. Pham, Nhung Thi Hong Dinh, Le Phuong Hoang Anh, Lan Thi Mai Dao, Van Dat Bui, Hong‐Nhung Dao, Duc Son Le, Anh Thi Lan Ngo, Quang‐Duong Le, Liem Nguyen Thanh

**Affiliations:** ^1^ Vinmec Research Institute of Stem Cell and Gene Technology College of Health Sciences VinUniversity Vinhomes Ocean Park Hanoi Vietnam; ^2^ Vinmec Health Care System Hanoi Vietnam; ^3^ School of Chemical Engineering College of Engineering Sungkyunkwan University (SKKU) Suwon Republic of Korea

**Keywords:** cell therapy, extracellular vesicle‐based therapy, tissue engineering, regenerative medicine, stem cell, bioprinting

## Abstract

From the pioneering days of cell therapy to the achievement of bioprinting organs, tissue engineering, and regenerative medicine have seen tremendous technological advancements, offering solutions for restoring damaged tissues and organs. However, only a few products and technologies have received United States Food and Drug Administration approval. This review highlights significant progress in cell therapy, extracellular vesicle‐based therapy, and tissue engineering. Hematopoietic stem cell transplantation is a powerful tool for treating many diseases, especially hematological malignancies. Mesenchymal stem cells have been extensively studied. The discovery of induced pluripotent stem cells has revolutionized disease modeling and regenerative applications, paving the way for personalized medicine. Gene therapy represents an innovative approach to the treatment of genetic disorders. Additionally, extracellular vesicle‐based therapies have emerged as rising stars, offering promising solutions in diagnostics, cell‐free therapeutics, drug delivery, and targeted therapy. Advances in tissue engineering enable complex tissue constructs, further transforming the field. Despite these advancements, many technical, ethical, and regulatory challenges remain. This review addresses the current bottlenecks, emphasizing novel technologies and interdisciplinary research to overcome these hurdles. Standardizing practices and conducting clinical trials will balance innovation and regulation, improving patient outcomes and quality of life.

## Graphical abstract

*Tissue engineering and regenerative medicine*. This review presents cell therapy, extracellular vesicle therapy, and tissue engineering in regenerative medicine, highlighting their key historical milestones, clinical applications, and current challenges. It emphasizes future directions, focusing on advancing clinical translation and personalized medicine, establishing standardization and regulation, and fostering multidisciplinary research and interdisciplinary collaboration to enhance the clinical impact of these integrated approaches.

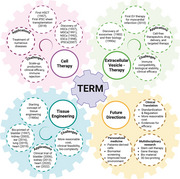

## 1 Introduction

**FIGURE 1:**
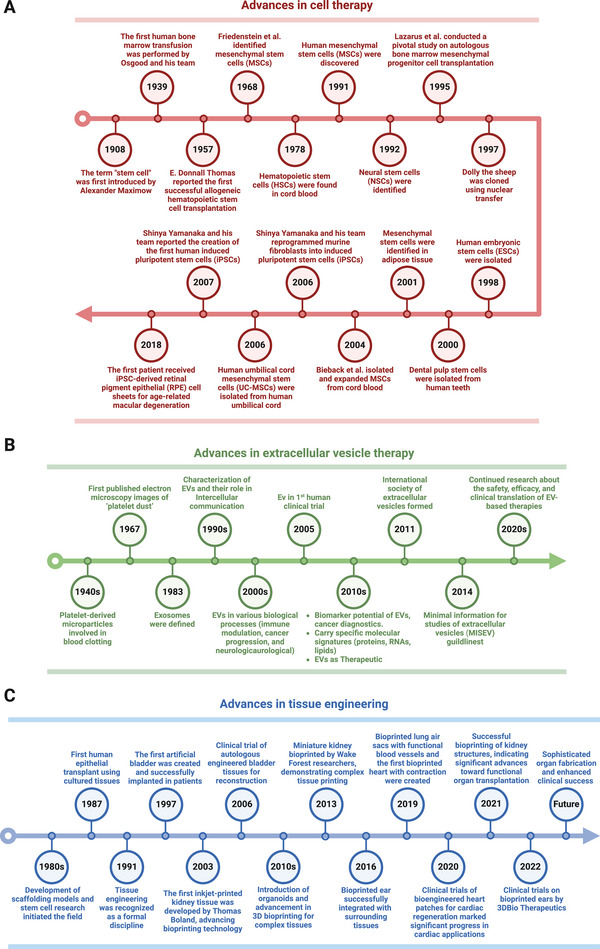
Timeline of tissue engineering and regenerative medicine. (A) Advances in cell therapy. (B) Advances in EV therapy. (C) Advances in tissue engineering. *Abbreviations*: embryonic stem cells (ESCs), extracellular vesicles (EVs), mesenchymal stem cells (MSCs), minimal information for studies of extracellular vesicles (MISEV), neural stem cells (NSCs), induced pluripotent stem cells (iPSCs), retinal pigment epithelial (RPE), and umbilical cord (UC).

Tissue engineering and regenerative medicine (TERM) is at the forefront of modern healthcare innovation, offering transformative potential in treating various diseases. TERM aims to repair, replace, and restore the functions of damaged tissues or organs in the body via multidisciplinary approaches such as cell and gene therapy, cell‐free therapy, biomaterial engineering, and 3D bioprinting [1]. The field of regenerative medicine, with stem cell research at its heart, has a rich history (Figure 1A). The term “stem cell” was first introduced by the Russian histologist Alexander Maximow in 1908 to describe hematopoietic progenitor cells [2]. Thomas et al. [3] reported the first successful allogeneic hematopoietic stem cell transplantation (HSCT) for blood cancers in 1957, marking a significant milestone in medical history. In 1968, Friedenstein et al. [4] identified a unique cell type that laid the groundwork for the concept of mesenchymal stem cells (MSCs). MSCs from many other tissue sources, such as bone marrow (BM), adipose tissue (AT), dental pulp (DP), the placental membrane, the umbilical cord (UC), and umbilical cord blood (UCB), have been intensively investigated in clinical settings [5].

A pivotal advancement in stem cell research occurred in 2007 when Yamanaka and colleagues [6] reprogrammed human fibroblasts into induced pluripotent stem cells (iPSCs), which resemble embryonic stem cells (ESCs). In 2013, the first patient received an iPSC‐derived retinal pigment epithelial (RPE) cell sheet to treat advanced neovascular age‐related macular degeneration [7]. The study reported no serious adverse events (SAEs), and the transplanted cells survived with slight expansion of the pigmented area at the 4‐year follow‐up [8]. Currently, cell therapy has led to both the number of clinical trials and translational success in clinical practice. Recent innovations have explored integrated approaches that combine stem cell biology, gene technology, and bioengineering. These include the use of gene therapy and gene editing to correct genetic mutations or weapon stem cells with advanced features, in vivo reprogramming, the use of scaffolds to provide the natural microenvironment of stem cells, and the encapsulation of allogenic grafts to hijack immune cell attack [9].

Emerging evidence has demonstrated the potential of extracellular vesicles (EVs) as alternatives to cell‐based therapies in regenerative medicine (Figure 1B) [10]. EVs, which include various types of membrane vesicles secreted from cells, carry multiple types of functional cargo from lipids and proteins to ribonucleic acids (RNAs) and consequently play essential roles in physiological or pathological processes such as cell–cell communicators [11]. Since the first description of EVs in 1946 by Chargaff and West [12], many studies have focused on EV‐related therapeutic applications, especially EVs derived from stem cells, immune cells, and fibroblasts for regenerative purposes [13, 14, 15]. For example, MSC‐derived EVs with regenerative functions were first described in 2010 by Lai et al. [16] for use in myocardial ischemia (MI) intervention, which paved the way for subsequent studies on MSC‐EVs. Later, fibroblast‐derived EVs and macrophage‐derived EVs were proven effective delivery systems for RNA and protein, respectively, for treating photodamaged skin and **PD** [17, 18]. EVs are attributed to the functions of cells [19] and offer enhanced safety, stability, and numerous possibilities for content modifications [20]. The ability to engineer EVs to carry specific therapeutic agents further enhances their potential, allowing for targeted delivery and reducing side effects. These attributes make EV‐related therapies increasingly attractive for future drug development.

Progress in TERM represents many breakthroughs in tissue engineering, aiming to create biological substitutes to replace damaged organs (Figure 1C). Burke et al. [21] first reported using artificial skin for extensive burns in 1981. However, Langer and Vacanti [22] are acknowledged as pioneers in tissue engineering for their 1988 introduction of the concept of seeding cells onto biodegradable scaffolds to support tissue regeneration. Their work laid the foundation for modern regenerative medicine by exploring the creation of bioengineered organs to replace irreversibly damaged organs such as the bladder (1997), kidney (2013), ear (2016), and heart (2019) [23]. The field has seen its first successful clinical translations, with bladder and ear transplantation reported for seven and five patients, respectively [23, 24]. Recent advancements include the development of sophisticated scaffolds and biomaterials, integrating stem cells and gene editing technologies, and advances in 3D bioprinting technologies, further enhancing the potential of tissue engineering.

TERM, which leverages cell therapy, EV therapy, and tissue engineering, offers significant potential for advancing healthcare. However, several challenges still need to be overcome. This review provides an overview of TERM, focusing on clinical translation. We explore the current state of translational research in cell therapy, EV‐based therapy, and tissue engineering. Drawing on the latest published data and ongoing clinical trials, we discuss the trends and challenges in the field of regenerative medicine. In addition, we highlight the importance of interdisciplinary collaboration in overcoming these challenges. The integration of stem and immune cell biology, molecular biology, bioengineering, bioinformatics, and clinical expertise is crucial for the successful translation of TERM innovations from the bench to the bedside. By studying these multifaceted aspects, we aim to offer insights and recommendations for the future directions of regenerative medicine.

## 2 Cell Therapy: Applications and Challenges

Cell therapy has progressed from early blood transfusions to advanced stem cell applications, showing the potential to treat diverse diseases by replacing or repairing damaged cells. Breakthroughs in hematopoietic, mesenchymal, pluripotent, and neural stem cell (NSC) therapies have laid a strong foundation for regenerative medicine, with notable achievements in treating hematologic malignancies, autoimmune disorders, and degenerative diseases. However, challenges such as immune rejection, limited cell survival, and complex interactions with the host environment continue to hinder broader clinical success. Advances in gene editing, engineered cell products, and immunomodulatory approaches are poised to overcome these obstacles. By revealing the intricate dynamics between therapeutic cells and the host, the field has moved closer to unlocking the full potential of cell therapy in addressing critical unmet medical needs.

### 2.1 The Fundamental Concept of Stem Cell Therapy

The concept of cell therapy predated HSCT by several centuries, with the discovery of circulating blood in 1628 and the subsequent development of blood transfusion techniques [4]. The first HSCT transplantation by Thomas et al. in 1957 [3], performed on twin siblings to treat leukemia, established a critical milestone in cell therapy. These procedures underscore the therapeutic potential of replacing pathological cells with healthy cells, a principle that remains foundational in cell therapy. Landsteiner and Thomas were awarded Nobel Prizes in 1930 and 1990, respectively, for their seminal contributions to regenerative medicine.

**FIGURE 2:**
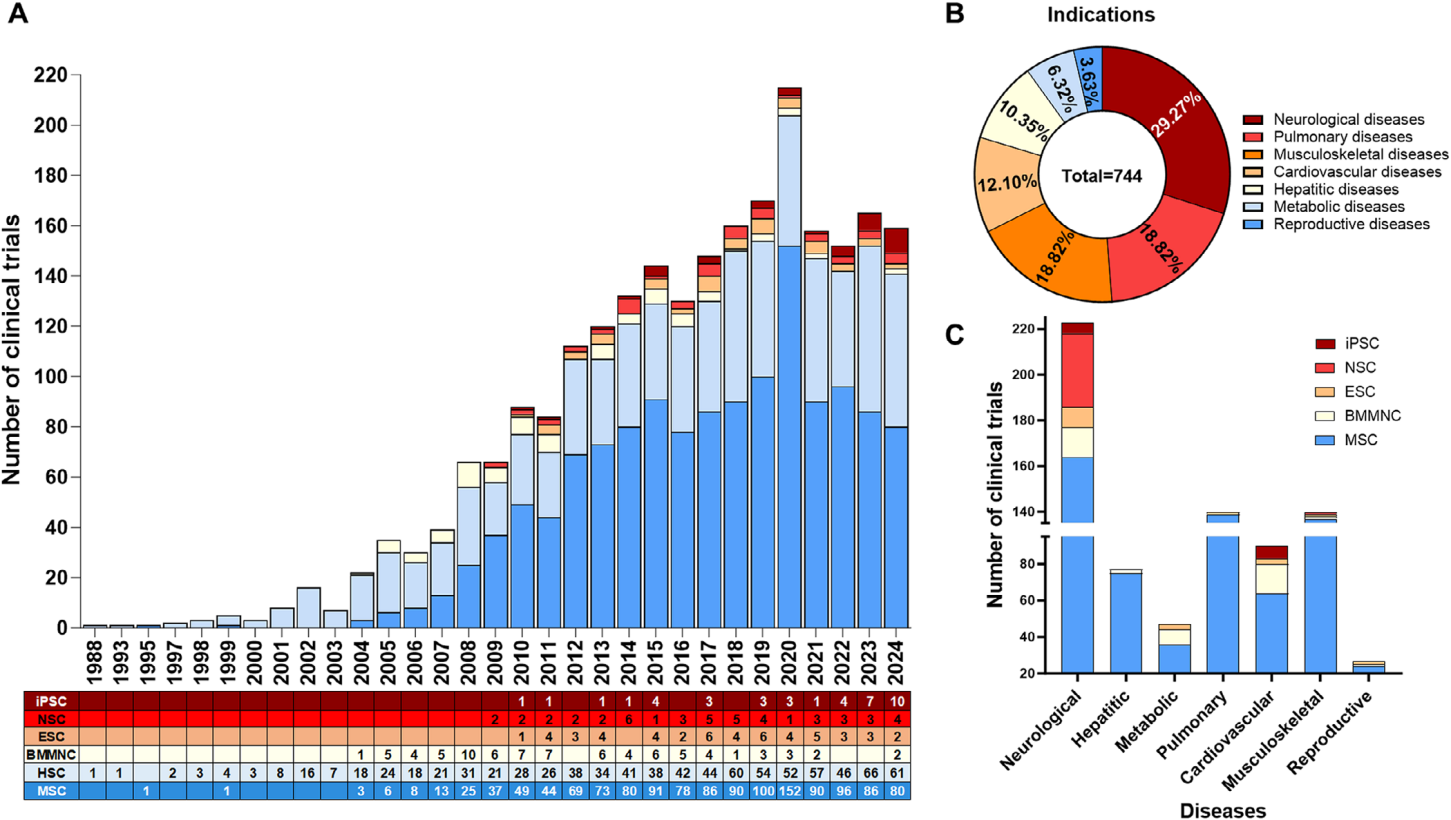
Clinical trials conducted for cell therapy. (A) Studies on cell therapy were registered from 1988 to 2024, with MSCs‐ and HSCs‐based therapies leading in number. The number of trials on iPSCs has increased, whereas the number of trials on BM‐MNCs has decreased in the last decade. (B) Studies were classified on the basis of disease category, with neurological diseases being the most common. (C) Indications of the five cell types (MSCs, NSCs, iPSCs, and ESCs) for each disease category, suggesting that MSCs are overall the most frequently studied, whereas iPSCs are predominantly used in neurological and cardiovascular diseases. *Abbreviations*: bone marrow mononuclear cells (BM‐MNCs), embryonic stem cells (ESCs), hematopoietic stem cells (HSCs), mesenchymal stem cells (MSCs), neural stem cells (NSCs), and induced pluripotent stem cells (iPSCs).

Stem cell therapy began in the late 20th and early 21st centuries with the findings of hematopoietic stem cells (HSCs) in cord blood in 1978 [25], human MSCs in 1991 [26], NSCs in 1992 [27], human ESCs in 1998 [28], and human iPSCs in 2007 [6]. Stem cells can be identified, isolated, and cultured in vitro, allowing exploration of their potential in numerous diseases (Figure 2A,B). Early clinical trials on cell therapy used HSCs, followed by MSCs. These two cell types are the major contributors to cell therapy clinical research (Figure 2A), according to www.clinicaltrial.org. The clinical translation of pluripotent stem cells (PSCs), including ESCs and iPSCs, began in 2010. While the number of ESC trials has recently decreased, trials using iPSCs have increased owing to advanced reprogramming and differentiation technology, making personalized medicine more accessible. Finally, NSCs rank among the top five most common cell types in cell therapy and are predominantly applied for neurological diseases (Figure 2C).

### 2.2 Stem Cell Types for Cell Therapy

#### 2.2.1 HSC Therapy: The Pioneer of Cell Therapy

Currently, HSCT is the standard of care and a clinical option for treating hematologic malignancies, solid tumors, autoimmune diseases, and numerous congenital diseases [29]. The underlying principle of HSCT is based on the unique ability of HSCs to reconstitute the entire blood system following transplantation in BM‐ablation recipients [30]. HSCs are the progenitors of all blood cell lineages and play a crucial role in sustaining lifelong hematopoiesis. These cells predominantly reside in the adult BM, where their functions are highly regulated by a specialized microenvironment known as the niche [31]. The stem cell niche ensures a balance between HSC self‐renewal and differentiation, adapting to both homeostatic conditions and the physiological demands imposed by infections and injuries.

Since the first HSCT in 1957, both autologous and allogeneic transplantation procedures have undergone continuous development to increase their safety and efficacy [32]. Significant advancements have been achieved in conditioning regimens, preventing and treating graft‐versus‐host disease (GvHD), and infection management. Furthermore, the choice of donor and HSC sources, including BM, mobilized peripheral blood, and UCB has significantly expanded. The number of human leukocyte antigens (HLA)‐haploidentical transplants has increased annually, making HLA‐haploidentical relatives a viable alternative to HLA‐fully matched siblings and unrelated donors. The most recent breakthrough in HSCT involves the treatment of genetic disorders via gene therapy and gene‐editing modified HSCs. The United States Food and Drug Administration (US FDA) has approved ex vivo genetically modified HSC‐based therapeutics, including CASGEVY, LYFGENIA, ZYNTEGLO for the treatment of ß‐thalassemia and sickle cell disease [33], and HEMGENIX for hemophilia B [34], marking a significant step forward in the pioneering applications of stem cells and gene technology to treat unmet medical needs.

#### 2.2.2 MSC Therapy: A Beacon of Hope for Severe Diseases, yet Challenges Remain

**FIGURE 3:**
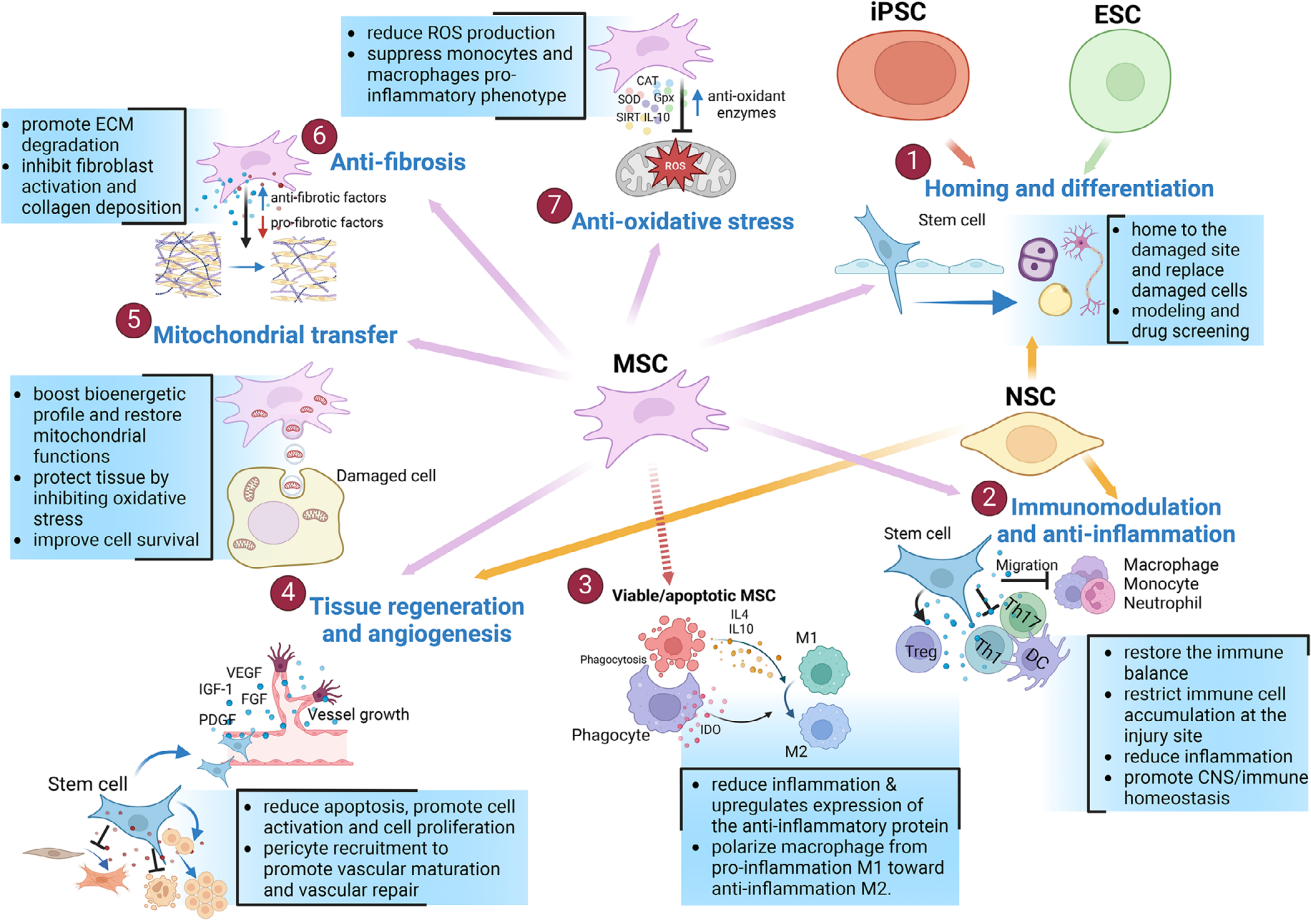
Potential effects of stem cells in regenerative medicine and their mechanisms of action. (1) ESCs, iPSCs, NSCs, and MSCs can differentiate into many cell types, replace damaged cells, and serve as tools for disease modeling and drug screening. (2) NSCs and MSCs suppress immune responses by reducing inflammation, restricting immune cell accumulation, and inhibiting the activity of Th1, Th17, and dendritic cells. They also promote regulatory T cells (Treg) function to restore immune balance. The immunomodulatory ability of these cells is important for maintaining homeostasis in the CNS. (3) Phagocytosis of viable or apoptotic MSCs by phagocytes increases IDO levels and further enhances anti‐inflammatory effects by promoting M2 macrophage polarization. (4) NSCs and MSCs can secrete growth factors, such as BDNF, NGF, VEGF, PDGF, IGF‐1, and FGF, which create a favorable environment for tissue regeneration, stimulate cell proliferation and enhance vessel growth. (5) MSCs transfer mitochondria to damaged cells, improving cellular bioenergetic profiles, restoring mitochondrial functions, and increasing cell survival by protecting tissues from oxidative stress. (6) MSCs release antifibrotic factors that reduce excessive ECM deposition, inhibit fibroblast activation, and prevent collagen buildup, thereby reducing fibrosis. (7) MSCs reduce ROS production and promote the activity of antioxidant enzymes such as CAT, SOD, GPx, SIRT, and IL10, leading to the suppression of proinflammatory phenotypes in monocytes and macrophages. *Abbreviations*: brain‐derived neurotrophic factor (BDNF), catalase (CAT), central nervous system (CNS), extracellular matrix (ECM), embryonic stem cells (ESCs), fibroblast growth factors (FGF), glutathione peroxidase (GPx), mesenchymal stem cells (MSCs), nerve growth factor (NGF), neural stem cells (NSCs), indoleamine‐pyrrole 2,3‐dioxygenase (IDO), insulin‐like growth factor‐1 (IGF‐1), interleukin (IL), induced pluripotent stem cells (iPSCs), reactive oxygen species (ROS), T helper cells (Th), platelet‐derived growth factor (PDGF), regulatory T cells (Treg), sirtuin (SIRT), superoxide dismutase (SOD), and vascular endothelial growth factor (VEGF).

MSC‐based therapy has emerged as the most prevalent approach in cell therapy (Figure 2A). Since the first clinical trial in 1995, the number of trials utilizing MSCs has quickly increased, addressing numerous conditions, such as neurological diseases, musculoskeletal disorders, pulmonary diseases, and autoimmune conditions (Figure 2B,C) [35]. Over 1000 clinical trials and a myriad of preclinical studies have been conducted, shedding light on the clinical potential and mechanisms of action of MSCs (Figure 3) [36].

MSCs possess unique properties that distinguish them from other cell therapies. They are found in various tissues of both neonatal and adult origins. Neonatal MSCs are derived from sources such as the amniotic fluid, placenta, UC, and UCB. In contrast, adult MSCs are present in AT, BM, DP, heart, lung, muscle, oral mucosa, skin, and several other body fluids [5, 37]. MSCs can be easily expanded in vitro and exhibit low immunogenicity, making them promising candidates for allogeneic use [38, 39]. Unlike other stem cell therapies that function primarily through the differentiation and replacement of damaged cells, MSCs exhibit extensive paracrine activity. They secrete various cytokines, growth factors, hormones, and EVs, which carry bioactive molecules such as proteins, lipids, and nucleic acids, to stimulate endogenous stem cells and modulate their microenvironment [35, 36].

MSCs can sense inflammatory signals, enabling them to migrate to injury sites and promote regeneration [5, 40]. They interact with immune cells through their secretome and immune‐mediated phagocytosis to mediate immunomodulation [41, 42]. The immunomodulatory properties of MSCs have been extensively studied. MSCs interact with monocytes/macrophages, NK cells, dendritic cells, neutrophils, T cells, and B cells through both direct cell–cell contact and the secretion of anti‐inflammatory substances [42, 43]. Recent research highlights the significant role of macrophages in MSC‐mediated immunomodulation. MSCs are phagocytosed by monocytes/macrophages, leading to metabolic reprogramming and their transformation into the anti‐inflammatory M2 phenotype [41, 44, 45]. Emerging evidence has demonstrated the immunoregulatory and tissue regenerative effects of dead or apoptotic MSCs, which depend on the efferocytosis of these cells by phagocytes [46, 47, 48].

Additionally, MSCs enhance angiogenesis and vascular regeneration by secreting proangiogenic growth factors and miRNAs, improving the survival and proliferation of endothelial cells, modulating chronic inflammation to support tissue regrowth, and reducing oxidative stress, which impairs vascular function [49, 50]. MSC therapy also influences the mitochondrial functions and energy metabolism of target cells through mitochondrial transfer, impacting various organ systems, including the respiratory, cardiovascular, visual, central nervous, digestive, and urinary systems [51, 52, 53].

Between 2010 and 2018, 11 MSC products received commercial approval: five in Korea, two in Japan, and one each in Europe, Canada, New Zealand, and India. These MSC products are indicated for a variety of conditions, including amyotrophic lateral sclerosis (ALS), critical limb ischemia, Crohn's disease, GvHD, knee articular cartilage defects, MI, spinal cord injury (SCI), and subcutaneous tissue defects [54]. Recently, the US FDA approved the first MSC therapy in the United States and the eleventh MSC product worldwide, which uses allogenic BM‐MSCs to treat steroid‐refractory acute GvHD [55]. The limited number of approved products reflects challenges in MSC therapy, including inconsistent therapeutic outcomes, limited cell survival posttransplantation, and the complexity of interactions between the host environment and injected cells. However, advancements in engineered MSCs and bioengineering approaches offer promising solutions. These innovations aim to increase MSC potency, improve the homing, retention, and immune compatibility of injected MSCs, and ultimately increase the efficacy of MSC‐based therapies [36].

#### 2.2.3 PSC Therapy: Advanced Therapeutics for the Future

PSCs have a remarkable capacity for differentiation into any cell type in the body, making them valuable assets in regenerative medicine (Figure 3). Originally, PSCs, known as ESCs, were first isolated from early‐stage embryos by Thomson in 1998 [28]. These cells can self‐renew and differentiate into all three germ layers (ectoderm, mesoderm, and endoderm) that form the human body. ESCs have been explored for the treatment of various degenerative diseases and injuries, such as SCI, PD, age‐related macular degeneration, type 1 diabetes, and intrauterine adhesion [56, 57]. However, cell therapies using ESCs face several critical issues that have limited the widespread clinical application of hESCs, including (1) the risk of forming teratomas (tumors) if not properly controlled during differentiation, (2) the allogeneic nature of ESC therapies, which may lead to immune rejection, and (3) ethical debates related to the destruction of embryos [58, 59].

Human iPSCs, which were generated from adult somatic cells via the transfer of four reprogramming factors—OCT3/4, SOX2, KLF4, and cMYC—for the first time by Takahashi et al. in 2007 [6], can bypass the ethical concerns linked to hESCs. iPSCs share many similarities with hESCs, such as the ability to self‐renew and differentiate into almost all cell lineages in the human body. However, as they are derived from easily obtainable adult somatic cells, cell therapies using iPSCs are more accessible and ethically acceptable than those from embryonic sources [58, 60]. iPSC‐based therapies, both in autologous and allogenic settings, have been explored in many clinical studies to treat brain, spinal cord, eyes, BM, heart, lungs, and autoimmune system diseases [57, 61]. Key challenges of iPSC‐based therapies include the high complexity of iPSC cell biology, concerns about teratoma formation, incomplete maturation of iPSCs after differentiation into the cells of interest, lack of efficient methods to remove residual undifferentiated iPSCs, genetic heterogeneity, and acquired mutations during reprogramming [61]. Current research is focused on overcoming these challenges to unlock the full potential of PSCs.

#### 2.2.4 NSCs: Targeted Applications for Neurodegenerative Diseases

NSCs are found in the nervous system and can self‐renew and differentiate into several cell types, such as neurons and glial cells (astrocytes and oligodendrocytes). NSCs play crucial roles in brain development and repair. NSCs have essential functions in the treatment of neurological diseases, including immunomodulation and anti‐inflammatory effects, the secretion of growth factors, cell differentiation, neuroprotection, and neurogenesis (Figure 3). Preclinical data have shown that NSCs are favorable sources for treating neurological diseases. Although clinical reports of NSCs for treating neurological diseases are limited, some trials have shown promising outcomes and highlighted the potential utility of NSCs in the treatment of neurological diseases, such as ALS, Parkinson's disease (PD), and stroke [62].

### 2.3 Cell Therapy Applications in Regenerative Medicine

#### 2.3.1 Neurological Diseases

Neurological diseases, encompassing a broad spectrum of conditions that affect the central and peripheral nervous systems, remain a significant challenge in modern medicine because of their complex pathophysiology and often debilitating effects on patients. Current therapeutic approaches for neurological disorders involve controlling symptoms and delaying the course of the disease, which has resulted in limited success in halting disease progression. As a result, interest in regenerative medicine, particularly cell therapy, as a novel strategy to address unmet needs in neurological diseases has increased. Several cell types, such as NSCs, ESCs, MSCs, iPSCs, and mononuclear cells (MNCs), have shown potential for therapeutic use in neurological illnesses [59]. Among these cell types, MSCs are frequently used (Figure 2C).

##### 2.3.1.1 Stroke

Stroke is a leading cause of chronic disability and mortality. The only approved ischemic stroke treatment, tissue plasminogen activator, is limited to 15% of patients because of its 4.5‐h therapeutic window. Thrombectomy therapy can help in some cases but requires specialized resources, thereby restricting global access for most patients [63]. Several stem cells, including MSCs, NSCs, ESCs, and iPSCs, have been used to treat stroke.

MSCs have been widely studied for treating stroke via intravenous, intra‐arterial, or intracerebral injection [64]. MSCs protect the cerebral microvasculature from ischemic–reperfusion injury by reducing inflammation lowering astrocyte and microglial activation, leukocyte infiltration, and the levels of proinflammatory cytokines (interleukin [IL]‐1α, IL‐1β, IL‐6, and tumor necrosis factor [TNF‐α]), and increasing the levels of anti‐inflammatory cytokines (IL‐4, IL‐10, and interferon [IFN]‐β) [65, 66]. MSCs can home to damaged sites and differentiate into neuron‐like cells. Mitochondrial transfer by MSCs aids in stroke recovery by restoring function and enhancing survival, providing neuroprotection, and improving outcomes in stroke models [64]. MSC therapy for stroke is generally safe and shows promise with improvements in motor function, daily activities (higher Barthel Index [BI] scores), disability (lower modified Rankin Scale [mRS] scores), lesion volumes, and brain connectivity. However, these improvements are not consistently observed [5]. A meta‐analysis of nine randomized controlled trials (159 MSC‐treated patients and 147 controls) by Huang et al. [67] revealed that MSC transplantation improved neurological deficits in ischemic stroke patients but had a limited impact on the BI and mRS. Larger, well‐designed phase II trials are needed to confirm the therapeutic benefits of MSC therapy. The therapy shows promise but faces challenges, such as optimal timing for MSC administration. While some studies advocate delivery in the acute phase (within 48 h) for immunomodulation, others highlight the benefits of later administration (up to 1 month) in promoting neurogenesis and neuroplasticity, leaving the ideal timing uncertain.

In addition to MSCs, several NSC lines have been used to treat patients with stroke. The main mechanisms of action of NSCs in stroke include direct replacement of neurons, paracrine effects, angiogenesis, and neurogenesis [68]. Intracerebral injection of human NSC lines, such as NSI‐566 and CTX0E03, was well tolerated and improved the mRS, Fugl–Meyer motor score [69], National Institutes of Health Stroke Scale (NIHSS) score, and MRI in patients with ischemic stroke [69, 70]. However, no improvement in the NIHSS score was observed in patients during the subacute‐to‐chronic recovery phase [71].

ESC treatment improves stroke via angiogenesis and neurogenesis via their secretion [72, 73]. Although preclinical studies have shown benefits in terms of dopaminergic, sensory, and motor functions, clinical trials using ESCs for stroke are rare, likely because of concerns about teratoma formation and malignancy [74]. Only one trial reported improvements in functional recovery and severity of neurological deficits with no complications following ESC therapy [75].

Preclinical studies have demonstrated the role of iPSCs in treating stroke through their ability to replace cells and promote neuroprotection, immunomodulation, angiogenesis, and synapse formation [76]. However, the clinical application of iPSCs for stroke treatment is limited, with one ongoing phase 1 trial (NCT05993884) assessing iPSC‐derived endothelial progenitor cells (EPCs) in 27 acute ischemic stroke patients.

##### 2.3.1.2 Traumatic Brain Injury

Traumatic brain injury (TBI) is a severe condition that involves physical damage to brain tissue, particularly in young individuals [66, 77]. The mortality rate for acute severe TBI is as high as 36% [78]. The current guidelines for managing TBI emphasize controlling physiological factors such as blood pressure, intracranial pressure, oxygenation, and nutrition, among others [79].

MSC therapy holds significant promise for treating TBI through various delivery methods, including direct injection, intravenous infusion, lumbar puncture, and stereotactic implantation, by mitigating oxidative stress, reducing neuroinflammation, preventing apoptosis, and alleviating mitochondrial dysfunction. They transfer healthy mitochondria, regulate antioxidants, increase Bcl‐2 expression, and reduce microglial activation and proinflammatory cytokines while increasing anti‐inflammatory cytokines. MSCs also suppress immune cells, inhibit T‐cell proliferation, and polarize microglia to a neuroprotective state, enhancing neuronal survival and tissue repair [77]. The administration of BM‐MSCs improved neurological function without toxicity [80], enhanced brain function, consciousness, and motor ability [81], and improved motor function [82]. UC‐MSCs delivered via lumbar puncture improved neurological function and self‐care [83]. Despite promising evidence, challenges in MSC therapy for TBI include optimizing timing and the lack of standardized protocols. Consistent definitions of the MSC source, TBI severity, and dosage are needed to improve efficacy.

NSCs have also been explored as a treatment for TBI in one clinical trial by Wang et al. [84], who reported improvements in neurological function and increases in the serum levels of nerve growth factor (NGF) and brain‐derived neurotrophic factor (BDNF) without SAEs after the injection of 20–40 × 10^6^ autologous MSC‐derived NSCs into 10 TBI patients.

##### 2.3.1.3 Spinal Cord Injury

SCI commonly results in the loss of sensory, motor, and autonomic function below the injury level, with a global incidence of 10.5 per 100,000. Although rapid recovery of neurological function is desired, effective strategies for repairing damaged nerve cells are still lacking [85]. Several cell types, such as MSCs, NSCs, and ESCs, have been used for SCI. Among them, MSCs are the most prevalent.

MSCs show promise for spinal cord repair through anti‐inflammatory, neuroregenerative, and vascular‐supportive mechanisms. MSCs reduce inflammation by promoting anti‐inflammatory M2 macrophages, inhibiting the toll‐like receptor 4 (TLR4) and NF‐κB pathways, and decreasing inflammasome activity. They also support axonal regeneration via pathways such as the Wnt/β‐catenin and PI3K–mTOR pathways while promoting vascular repair by releasing angiogenic factors such as vascular endothelial growth factor (VEGF), fibroblast growth factor (FGF), and platelet‐derived growth factor (PDGF). These actions aid in restoring nerve function and improving motor recovery in SCI patients [85].

MSCs have been used in multiple clinical trials to treat chronic, acute, and subacute SCI. BM‐MSCs are the most popular source of MSCs for SCI [85]. These trials employed various delivery methods, such as intrathecal infusion, intraspinal injection, in situ transplantation, and intravenous injection, with doses ranging from 1 × 10⁶ to 4 × 10⁸ cells. Data suggest that local injections may be more effective than systemic approaches, and thus, intrathecal injection offers a convenient and fast‐acting method for multiple MSC doses [86]. Notably, the low concentration of cells used for transplantation (<5 × 10^7^ cells) had outcomes comparable to those of the high concentration of cells (≥5 × 10^7^ cells) [87]. MSC administration has been shown to improve sensation near the injury site, increase American Spinal Injury Association (ASIA) Impairment Scale grades, increase ASIA sensory and motor scores, improve self‐care and muscle tone, and significantly improve movement, bowel, and bladder function in patients with chronic complete SCI [85, 87, 88]. MSCs hold promise for SCI treatment, but factors such as dosing, mechanisms, cell survival, and transplantation protocols require clarification. Further research is needed to refine MSC therapy and understand SCI processes.

NSCs have also been used to treat patients with SCI in at least four clinical trials [58]. NSCs have been shown to modulate the inflammatory response by inhibiting reactive macrophages and increasing the expression of growth factors that are beneficial for recovery, such as NGF, BDNF, insulin‐like growth factor‐1 (IGF‐1), and glial cell line‐derived neurotrophic factor (GDNF) [89]. The intraspinal injection of NSCs (15–100 × 10^6^ total cells) was proven safe and feasible, with no SAEs. Patients have shown functional recovery and improvements in motor function and spasticity [58].

ESCs can differentiate into neurons and glial cells to replace nonfunctional cells in SCI [89]. Shroff et al. [90, 91] reported that patients with SCI had increased ASIA scores and neurological function, with no SAEs observed. However, the considerable proliferative ability of ESCs poses a risk of tumor formation, which may limit their use in clinical trials.

##### 2.3.1.4 Cerebral Palsy

Cerebral palsy (CP) is a group of permanent movement and posture disorders that are often accompanied by issues such as sensation, cognition, communication, behavior, epilepsy, and musculoskeletal problems. CP is the most common physical disability in children, with an incidence of 1.6 out of 1000 in high‐income countries and 3.4 out of 1000 in low‐ and middle‐income countries [92]. Despite over 180 interventions and a 30% reduction in incidence due to prevention efforts, there is still no cure for CP [93]. Several cell types, such as MSCs and ESCs, are currently under investigation for the treatment of CP. MSC therapy for CP has been administered via the intrathecal, stereotactic, and intravenous routes. It has been shown to be safe and to improve motor function, health status, comprehension, quality of life, and overall function in children with CP. However, further randomized controlled trials are needed to determine the optimal dose, frequency, timing, and administration routes [94]. MSC therapy may promote recovery through paracrine effects, involving the secretion of cytokines that reduce inflammation, support neuronal survival, increase angiogenesis, and activate endogenous repair mechanisms. Additionally, MSCs may differentiate into neurons and glial cells to replace damaged cells. However, most data suggest that their main therapeutic effect is establishing a reparative environment conducive to neural recovery rather than through direct cell engraftment [95]. The MSC sources tested to date included UC‐MSCs, BM‐MSCs, UCB‐MSCs, and Wharton's jelly‐MSCs, with UC‐MSCs being the most commonly used and doses ranging from 2 to 22 × 10⁷ cells in total (1–4 injections) or 1 × 10⁶ cells/kg body weight.

There are few clinical studies on the use of ESC therapy in CP. These findings suggest that ESCs may migrate to hypoperfused areas of the brain, “homing” to affected regions and potentially contributing to neurogenesis in CP. ESC treatment improved motor function and enhanced cognitive function after ESC treatment [96]. An improvement in motor function was also observed in CP patients treated with 1–2 × 10^7^ autologous MSC‐derived NSCs [58].

##### 2.3.1.5 Alzheimer's Disease

Alzheimer's disease (AD) is a chronic neurodegenerative disorder characterized by progressive dementia, memory loss, and cognitive decline. Its brain pathology involves amyloid β (Aβ) plaque accumulation and the intracellular formation of neurofibrillary tangles, leading to cholinergic neuron loss [66, 97, 98].

MSCs are the most commonly used cell type for AD. A recent literature review revealed that MSCs act on AD by reducing Aβ and NFT accumulation and inflammatory cytokines (TNF‐a, IL‐1b, and ROS) and the polarization of inflammatory M1 microglia into anti‐inflammatory M2 microglia, promote neurogenesis by differentiating NSCs into neural progenitor cells, ultimately into neurons, and enhance synapse formation. Additionally, MSCs have paracrine and autocrine effects by releasing cytokines, such as growth/differentiation‐15 and galectin‐3, and neurotrophic factors, such as VEGF, BDNF, and NGF, to increase neuronal repair [66, 97, 98]. MSCs also transfer functional mitochondria and miRNAs to increase their bioenergetic profile and improve microglial clearance of accumulated protein aggregates [98]. Clinical studies in which MSCs are used to treat AD are limited. To date, MSCs have been administered via stereotactical, intracerebroventricular, or intravenous routes with cell doses ranging from 3.0 × 10⁶ to 9.0 × 10⁷ cells. Recent studies and trials on MSC therapy for AD have reported promising safety profiles with no dose‐limiting toxicities or manageable adverse events (AEs) and have shown cognitive stability or improvement in some MRI and biomarker levels in some patients [99, 100, 101]. Several ongoing MSC trials for AD are listed on ClinicalTrials.gov, with results pending (NCT03117738, NCT02833792, NCT04684602).

Gene‐editing technologies such as clustered, regularly interspaced short palindromic repeats (CRISPR) hold promise for addressing AD. In early‐onset AD, CRISPR can correct autosomal‐dominant mutations in presenilin 1 and 2 (PSEN1/PSEN2). For late‐onset AD, it offers the potential to replace the high‐risk APOE4 isoform with the protective APOE2 isoform, potentially reducing the risk of developing AD by up to 40% [102]. Additionally, human cortex‐derived NSCs can be engineered to express IGF‐1. When these modified NSCs were transplanted into AD model mice, the spatial memory was effectively restored [103]. Clinical trials for gene‐edited stem cells are still in the early stages. In one study, autologous fibroblasts genetically modified to express human NGF were implanted into eight AD patients. After 22 months, no long‐term AEs were observed, cognitive assessments indicated a slower rate of decline, and PET scans revealed significant increases in cortical glucose metabolism. Autopsy findings also showed robust growth responses to NGF [104]. However, a recent study by Ortega et al. [105] highlighted the challenges of NGF gene therapy, with limited success in clinical trials. These findings underscore the need for further research and development to establish gene therapy as a viable treatment option for AD [105].

##### 2.3.1.6 Parkinson's Disease

PD is a chronic, progressive neurodegenerative disorder characterized by motor impairment, social dysfunction, α‐synuclein aggregation, and dopamine deficiency due to neuronal loss in the substantia nigra. Affecting 2–3% of people over 65 years of age, PD led to 5.8 million disability‐adjusted life years and 329,000 deaths in 2019, with rates expected to double by 2040. Current treatments focus on symptom management, including medications such as levodopa and dopamine agonists, as well as interventions such as deep brain stimulation and lesion surgery [106]. The main cell types, such as MSCs, NSCs, ESCs, and iPSCs, have been used to treat PD.

MSCs act through multiple mechanisms, including inhibiting α‐synuclein transmission, modulating apoptosis (by upregulating Bcl2 and downregulating Bax), and reducing inflammation by decreasing astrogliosis and microgliosis. MSCs also secrete neurotrophic factors such as BDNF, cerebral dopamine neurotrophic factor, and hepatocyte growth factor (HGF), supporting dopaminergic neuron survival and facilitating motor recovery. Additionally, MSCs produce anti‐inflammatory cytokines (IL‐10 and transforming growth factor‐beta [TGF‐β]), suppress proinflammatory cytokines (TNF‐α, IFN‐γ, and IL‐1β), and may facilitate mitochondrial transfer to damaged neurons, promoting cellular repair [106, 107, 108]. MSC research for PD began in 2010 with a study by Venkataramana et al. [109], where BM‐MSCs improved motor function, disease severity, and quality of life (based on the Unified PD Rating Scale (UPDRS), Hoehn and Yahr, and Schwab and England scores), facial expression, gait, and freezing episodes with no SAEs reported. Since then, several studies have been conducted to investigate the potential of MSC therapy in PD. BM‐MSCs are feasible for treating mild to moderate PD and are safe and tolerable [110]. UC‐MSCs improved UPDRS scores and offered additional cognitive and emotional benefits over BM‐MSCs, including reduced anxiety and depression. Both cell types improved motor and daily living functions, highlighting UC‐MSCs as promising options [111, 112]. PD patients treated with AT‐MSCs also experienced no AEs over 6 months and improved Movement Disorder Society‐UPDRS scores, suggesting the potential of AT‐MSCs as PD therapy [113]. In PD, advanced glycation end products (AGEs) contribute to dopamine neuron apoptosis, but soluble AGE receptors can counter this effect. Using CRISPR–Cas9, UC‐MSCs have been engineered to secrete these receptors, reducing neuronal death and improving motor function in a PD mouse model, demonstrating promising therapeutic potential [114].

NSCs have been tested in two clinical trials for PD. One trial involved transplanting 3 × 10^7^ neural precursor cells into 21 patients, resulting in significant symptom improvement with no major side effects [115]. Another study by Madrazo et al. [116] transplanted 2 × 10^6^ neural progenitor cells into eight patients and reported no AEs or motor function improvements in seven out of eight patients. Other stem cell sources have also been investigated for PD. Two trials in South Korea (NCT06477744, NCT05887466) are testing ESC‐derived dopamine progenitor cells, which were completed in 2029 and 2026. A recent case report described a personalized cell therapy approach using autologous iPSC‐derived dopaminergic progenitor cells in a PD patient. Clinical and imaging findings have indicated potential benefits, including improvements in motor function and patient‐reported symptoms [117]. Another US‐based phase 1 trial (NCT06422208) assessing autologous iPSC‐derived dopamine neurons is expected to finish in 2026.

##### 2.3.1.7 Autism Spectrum Disorder

Autism spectrum disorder (ASD) is a neurodevelopmental disorder characterized by repetitive behaviors, limited activities, and social communication difficulties. In 2021, the WHO estimated that ASD affects one in 270 people, with a higher prevalence in men, and by 2023, the estimate had increased to one in 100 people globally [118]. Current treatments for ASD, such as psychotropic drugs, therapies, and educational support, can help manage symptoms such as irritability, seizures, and mood disorders but do not modify the underlying condition [119, 120].

The role of MSCs in treating ASD has only recently begun to be explored, and studies suggest that they may support neurogenesis and synaptogenesis, regulate synaptic function and plasticity by secreting growth factors, enhancing synaptic plasticity, restoring neurotransmitter release, and integrating into synaptic networks [121, 122]. In ASD, where there is an imbalance between Th1 and Th2 cells, excessive proinflammatory markers, and low anti‐inflammatory responses, MSCs may restore immune balance by inhibiting TNF‐α, IL‐1β, and IFN‐γ and increasing the levels of IL‐10 and IL‐4 [122]. Additionally, MSCs offer neuroprotection by reducing neural apoptosis, microglial activation, astrocyte proliferation, and oxidative stress [120, 122].

MSC therapy shows promise for ASD treatment, as it is safe and potentially effective. Lee et al. [123] treated an ASD patient with UC‐MSCs and reported improved social communication and reduced CARS scores without side effects. Sun et al. [124] administered intravenous MSCs to 12 patients and reported improvements in autism severity and social communication with no AEs. Sharifzadeh et al. [125] used intrathecal BM‐MSCs in a trial and reported specific improvements in CGI severity and CARS scores. However, further studies are needed to confirm the benefits of MSC therapy.

CRISPR gene editing offers the potential for addressing the genetic underpinnings of ASD, which is often associated with rare monogenic mutations or complex polygenic influences. The CRISPR strategy creates a versatile experimental platform to systematically explore the role of ASD‐associated genes in human cells and represents a potential treatment for ASD [126]. Recently, the US FDA approved a phase I clinical trial for JAG201 (NCT06662188), a gene replacement therapy targeting ASD associated with SHANK3 mutations and Phelan–McDermid syndrome. JAG201 utilizes an adeno‐associated virus serotype 9 vector to deliver a functional SHANK3 minigene directly to neurons in the central nervous system. This approach seeks to restore the synaptic function critical for neurodevelopment and maintaining cognitive and motor skills. The trial is expected to conclude in 2031.

##### 2.3.1.8 Amyotrophic Lateral Sclerosis

ALS is a rare, fatal neurological disease affecting upper and lower motor neurons, with an incidence of 0.6–3.8 per 100,000 people [127]. Although ALS is becoming more prevalent, Riluzole remains the only approved treatment, and a cure is still elusive.

MSCs have shown promise in preclinical and clinical studies and are administered intrathecally, intravenously, or via direct spinal cord injection. MSC therapy supports neural health by releasing neurotrophic factors for neuroprotection and neurogenesis and promoting an anti‐inflammatory environment in the central neural system (CNS). MSCs can also promote synaptic connection and remyelination of damaged axons and reduce apoptosis. In cerebrospinal fluid, MSCs increase Tregs and Th2 cells and the levels of anti‐inflammatory cytokines such as IL‐4 and IL‐10, decrease activated dendrites, and release TGF‐β, promoting CNS homeostasis and transforming microglia from an inflammatory (M1) state to an anti‐inflammatory (M2) state. These effects help regulate ALS progression and maintain CNS function [128]. The first clinical trial in which MSCs were used to treat ALS was conducted by Mazzini et al. in 2003 [129]. Since then, multiple studies have explored the therapeutic effects of MSCs on ALS, showing safety profiles without serious side effects. MSC‐treated patients experienced slower disease progression, improved forced vital capacity (FVC), increased life expectancy, and improved ALS functional rating scale (ALS‐FRC) scores [127, 128].

In addition to MSCs, NSCs are typically given at doses ranging from 5 × 10^4^ to 1 × 10^5^ cells/injection, with 1–5 injections via unilateral or/and bilateral intraspinal injection to treat patients with ALS. NSCs can slow the progression of ALS symptoms and prolong survival time by providing neuroprotection, reducing inflammation and astrocyte activation, and enhancing synaptic plasticity [62]. NSCs have also been demonstrated to migrate and integrate into the spinal cord and differentiate into neural phenotypes to delay the deterioration of motor ability in an ALS rat model. A phase 1 trial revealed that injections of NSCs are safe and tolerable [130], delay disease progression [131], and improve the ALS‐FRC or Medical Research Council scores [132]. A phase 1/2 trial using neural progenitor cells modified with a lentiviral vector to express glial‐derived neurotrophic factor (CNS10‐NPC‐GDNF) was conducted in 18 ALS patients. It revealed a trend toward improved motor function in treated limbs, although the efficacy of this approach was limited by incomplete virus penetration and an immune response [133].

#### 2.3.2 Hepatic Diseases

Liver diseases pose a serious global health threat, causing approximately two million deaths annually. Nearly half of these cases are due to liver cirrhosis (LC), followed by viral hepatitis and liver cancer. LC often develops from chronic liver conditions, such as hepatitis B, alcohol use, nonalcoholic fatty liver disease, and autoimmune liver disease. Treatments for decompensated cirrhosis or liver failure are limited, with liver transplantation being the only effective option. However, it is limited by organ shortages, high costs, immune rejection, and recurrent infections [134, 135].

MSCs are the most commonly used cell source in clinical cell therapy studies for liver diseases [136]. MSCs support liver disease treatment through several mechanisms, including differentiation, immunomodulation, antifibrosis, and ferroptosis inhibition. MSCs can differentiate into hepatocytes in vitro, aiding in tissue repair in animal models. They modulate both innate and adaptive immunity by interacting with natural killer (NK) cells, Kupffer cells, macrophages, dendritic cells, T cells, and B cells, reducing liver inflammation and damage. MSCs secrete IL‐10 and TNF‐α, inhibiting hepatic stellate cell activation and inducing apoptosis via the Fas‐FasL pathway. They also promote liver stem cell regeneration and secrete matrix metalloproteinases (MMPs) to break down the extracellular matrix (ECM). Additionally, MSCs protect hepatocytes from ferroptosis by reducing reactive oxygen species and Fe^2+^ levels. The four main routes of MSC transplantation include the hepatic artery, portal vein, peripheral vein, and intraperitoneal routes.

A systematic review and meta‐analysis of 11 randomized controlled trials assessed the efficacy and safety of MSC therapy in patients with LC. The results demonstrated that MSC infusion significantly improved liver function in LC patients, with reductions in Model for End‐Stage Liver Disease scores and increase in albumin levels. The optimal method of MSC delivery in liver disease remains controversial. Hepatic artery infusion was more effective than intravenous infusion. While hepatic artery infusion offers high MSC colonization, it also poses surgical risks, whereas the portal vein is prone to complications such as bleeding and embolism. A peripheral vein may be safer, easier, and more manageable, while the intraperitoneal route risks infection and adhesion. Further studies are needed to determine the optimal delivery route because of the clinical risks associated with hepatic artery infusion [135].

ESCs can be induced to differentiate into hepatocyte‐like cells in vitro, showing potential for liver disease treatment and organoid formation for disease modeling. However, ethical concerns, tumorigenicity risks, and immune rejection issues have prevented clinical trials using hESCs for chronic liver disease treatment. iPSC technology has been used in liver disease treatment through disease modeling. iPSCs have been reprogrammed into hepatocyte‐like cells [137], and these cells have also been used to develop disease models such as fatty liver disease and ornithine transcarboxylase deficiency [138]. Despite promising results, concerns about tumorigenicity and immunogenicity mean that iPSCs require further evaluation before clinical use. As a result, no clinical trials using iPSCs for chronic liver disease treatment have been conducted yet.

#### 2.3.3 Metabolic Diseases

Diabetes mellitus (DM) is a chronic metabolic disease characterized by elevated blood glucose levels, causing damage to blood vessels, the heart, eyes, kidneys, and nerves over time. In 2021, an estimated 537 million people had DM, a number projected to increase to 643 million by 2030 and 783 million by 2045 [139]. There are two main types of DM: type 1 DM (T1DM) and type 2 DM (T2DM).

##### 2.3.3.1 Type 1 DM

T1DM is an autoimmune disease in which the immune system destroys pancreatic insulin‐producing cells, leading to minimal or no insulin production. While exogenous insulin helps control blood sugar, it often fails to prevent complications and may cause poor glycemic control or hypoglycemia [139, 140].

Several clinical trials have evaluated the use of MSCs for treating T1DM, demonstrating that MSC therapy can increase C‐peptide levels while reducing insulin requirements and HbA1c levels [141, 142, 143, 144]. Despite these findings, a meta‐analysis of MSC trials revealed only improved HbA1c, with no significant changes in fasting glucose or C‐peptide [145]. Further large‐scale studies are needed to confirm these benefits because of the variability in MSC sources, doses, and patient numbers [140].

In vitro, the differentiation of stem cells into insulin‐producing cells represents a promising therapeutic strategy for T1DM, with iPSCs and ESCs emerging as ideal candidates for this approach. ViaCyte (ViaCyte Inc. San Diego, CA, USA) developed an immune isolation device to encapsulate pancreatic endodermal cells, which showed promise in controlling diabetes in rodents [146]. However, a clinical trial with 19 T1DM patients revealed high variability in outcomes, likely due to poor vascularization and hypoxia, leading to minimal cell survival after 12 weeks [147]. A modified device (VC‐02) with wider pores improved oxygenation but required immunosuppressive therapy due to a lack of immune protection. While some patients showed a C‐peptide response, none achieved insulin independence, and the results were limited by insufficient cell engraftment and fibrous tissue formation [148]. Vertex Pharmaceuticals (Boston, MA, USA) conducted a phase I/II trial (NCT04786262) using fully differentiated insulin‐producing cells derived from allogeneic PSCs, which also require immunosuppression. One patient achieved insulin independence, with an HbA1c of 5.2%, whereas the second showed only a 30% reduction in insulin needs. Despite promising early data, the need for immunosuppression remains a significant limitation, and further optimization is needed.

Researchers have developed a method to isolate and expand Tregs, which are often dysfunctional in T1DM while preserving their diversity and functionality. In a phase 1 trial with 14 patients, ex vivo‐expanded autologous Treg therapy proved safe, with some Tregs persisting for up to 1 year without SAEs. Notably, several patients maintained stable C‐peptide levels for more than 2 years, paving the way for a phase 2 trial to assess therapeutic efficacy [149].

##### 2.3.3.2 Type 2 DM

The most common form of diabetes is T2DM, which typically occurs in adults, where the body becomes resistant to insulin or fails to produce enough insulin. MSCs are the most commonly used cell type in clinical trials for treating T2DM. A summary of 18 clinical trials highlights that intrapancreatic and intravenous infusion methods are typically employed, with cell doses ranging from 0.3 to 300 × 10⁶ cells/kg, with 1 × 10⁶ cells/kg being the most frequently used dose. The proposed mechanisms include β‐cell regeneration, improved hepatic metabolic homeostasis, reduced insulin resistance, and the regulation of systemic inflammation [150]. MSCs play a role in initiating endogenous insulin production and stimulating the proliferation of β‐cells. However, the transdifferentiation of MSCs into β‐cells and their transplantation engraftment may not significantly contribute to the restoration of pancreatic function. Instead, MSCs secrete various cytokines and growth factors, including TGF‐β and VEGF, which enhance islet function through both paracrine and autocrine mechanisms while facilitating the vascularization process [151]. MSCs also are stimulated by inflammatory cytokines, including TNF‐α and IFN‐γ, which in turn shift to an immunosuppressive phenotype by inducing the secretion of soluble factors that mediate immunomodulatory activities, such as prostaglandin E2 (PGE2), HGF, indoleamine‐pyrrole 2,3‐dioxygenase, and IL‐10 [152].

MSCs can reduce islet cell apoptosis by decreasing the cleavage of caspase 3 [153]. MSCs can enhance the formation of autophagosomes by clearing impaired mitochondria and increasing the number of insulin granules [154]. MSC‐mediated mitochondrial transfer is a mainstay method for rescuing injured cells, restoring mitochondrial functions [155], and repairing renal proximal tubular epithelial cells in diabetic nephropathy in vivo [156]. The mitochondria of MSCs can be transferred to *β*‐cells under hypoxic conditions to increase the insulin secretion rate [157]. MSCs alleviate insulin resistance in T2DM patients by enhancing insulin signaling pathways. These compounds increase GLUT expression and increase the phosphorylation of IRS‐1 and AKT in insulin‐target tissues. MSCs also inhibit MG53, an E3 ligase that promotes IRS‐1 degradation in skeletal muscles, which aids insulin sensitivity. Additionally, MSCs suppress NLRP3 inflammasome formation, reducing inflammation and enhancing IRS‐1 and GLUT4 function in hepatic cells, further mitigating insulin resistance [150].

Clinical trials have shown promising potential for MSC therapy in T2DM. As of October 2024, a search on ClinicalTrials.gov using “mesenchymal stem cells” as the treatment and “diabetes type 2” as the condition revealed 25 registered studies, with nine completed [158]. MSC therapy significantly reduces fasting and postprandial blood glucose, HbA1c, and insulin requirements while improving C‐peptide levels and insulin resistance with mild and manageable symptoms like fever, nausea, headache, and minor hypoglycemia [159, 160].

Islet transplantation has recently shown promise for treating T2DM. Wu et al. [161] conducted the first‐in‐human trial of autologous E‐islets derived from patient‐specific iPSCs to treat T2DM. In a 59‐year‐old patient with advanced T2DM, islet transplantation improved glycemic control within 2 weeks, resolving severe hyperglycemia and hypoglycemia. By week 32, time in target range reached 99%, HbA1c decreased from 6.6 to 4.6%, and insulin was discontinued by week 11. Fasting C‐peptide levels tripled, and no tumors were detected during the 116‐week follow‐up. This study demonstrated the potential of stem cell‐derived islets to restore islet function in late‐stage T2DM [161].

Recently, Balboa et al. [162] demonstrated that insulin mutations disrupt β‐cell differentiation in a neonatal diabetes model. Using iPSCs derived from affected patients, researchers have applied CRISPR/Cas9 to correct missense mutations in the insulin gene. Single‐cell RNA sequencing revealed that, compared with corrected cells, mutant cells presented increased endoplasmic reticulum stress and reduced proliferation, highlighting the potential of gene editing to address β‐cell dysfunction [162]. Overall, cell‐based therapies show promise for treating diabetes, but further research is needed to confirm their efficacy and clinical applicability.

#### 2.3.4 Pulmonary Diseases

Pulmonary diseases encompass a wide range of chronic respiratory conditions and place a substantial burden on individuals and society due to high mortality rates and diminished quality of life [163]. Inflammation is central to many of these diseases, initiating a chain of events that leads to the loss of functional lung tissue and pathological pulmonary remodeling. Although this remodeling process aims to repair damage, it often leads to further structural changes that exacerbate respiratory dysfunction. Despite the innate regenerative capacity of the lungs, chronic inflammation under these conditions often overwhelms tissue repair mechanisms, impairing recovery [164]. Current treatments focus primarily on symptom relief and acute management but rarely address underlying pathological processes. For example, while inhaled corticosteroids and bronchodilators improve airflow and reduce inflammation in chronic obstructive pulmonary disease (COPD) patients, they do not halt the progressive decline in lung function [165]. Advanced options, such as lung transplantation, are limited by organ availability and potential complications [166]. These challenges highlight the urgent need for therapies to alter the disease course and improve long‐term outcomes.

##### 2.3.4.1 Acute Respiratory Distress Syndrome

Acute respiratory distress syndrome (ARDS) is a life‐threatening lung condition characterized by intense inflammation and fluid accumulation in the lungs’ tiny air sacs (alveoli), preventing adequate oxygen exchange. This inflammatory response, coupled with fluid buildup, impairs the ability of the lungs to fill with air, leading to a significant reduction in blood oxygen levels [167]. ARDS can rapidly progress, leading to organ failure and severe complications, often triggered by infections such as pneumonia and sepsis, trauma, or toxic exposure [168].

Preclinical studies have shown that MSCs effectively reduce inflammation and promote lung tissue regeneration in animal models of ARDS. This anti‐inflammatory effect is crucial in ARDS, addressing both lung damage and excessive immune responses. MSC‐based therapies have been shown to lower the levels of proinflammatory cytokines such as TNF‐α, IL‐1α, and IL‐6, improve survival rates, and potentially mitigate cytokine storms and inflammation‐related lung damage [169]. In response to the limitations of conventional treatments, MSCs also offer promising therapeutic options for managing ARDS because of their tissue repair properties and antimicrobial properties [170]. These attributes are particularly beneficial when infections trigger ARDS, help control inflammation, promote healing, and reduce the risk of further infection. Thus, using MSCs represents a comprehensive therapeutic strategy for improving ARDS outcomes, enabling faster recovery and minimizing lung damage.

Clinical trials on cell therapies for ARDS have evolved, with recent studies emphasizing larger cohorts and standardized outcome measures. Early studies (2013–2015) were relatively rare, often with fewer than 20 participants, and they primarily assessed safety and feasibility. Although some improvements in clinical parameters, such as the lung injury score (LIS) and SOFA score, have been reported, the lack of standardized outcome measures and variability in cell dosing have made comparisons challenging [171, 172]. Since 2017, there has been a shift toward larger patient cohorts and the incorporation of standardized outcome measures, such as the Acute Physiology and Chronic Health Evaluation II score and ventilator‐free days [173]. A 2023 study revealed that allogeneic MSCs significantly improved ventilator‐free days in patients with moderate to severe ARDS from COVID‐19, highlighting the potential of MSC therapy [174]. Despite initial success, further research is crucial to optimize cell types, dosages, and delivery methods to maximize therapeutic benefits for ARDS patients.

##### 2.3.4.2 Bronchopulmonary Dysplasia

Bronchopulmonary dysplasia (BPD) is a chronic lung condition that primarily affects premature infants who require prolonged oxygen therapy or mechanical ventilation soon after birth. The pathogenesis of BPD involves oxidative stress and inflammation, compounded by incomplete lung development, leading to damage to fragile developing lungs [175]. BPD results in inflammation, fibrosis, and long‐term respiratory problems that may persist into childhood and beyond. Infants with BPD often need ongoing respiratory support and are at increased risk of complications such as recurrent infections and pulmonary hypertension [176].

MSC therapy has emerged as a potential avenue for promoting lung repair. Clinical trials have focused on the potential of MSCs to repair lung tissue and reduce inflammation. MSCs are promising because they address oxidative stress, promote tissue repair, and mitigate inflammation, critical factors in BPD. Before 2018, research on MSC therapy was limited, with studies involving fewer than ten infants and focusing primarily on safety and feasibility. UC‐MSCs and UCB‐MSCs were selected for early‐phase studies because of their accessibility and regenerative potential. These preliminary investigations revealed that MSC therapy was well tolerated and did not cause SAEs (Table S1) [177, 178, 179]. However, the lack of standardized outcome measures made cross‐study comparisons difficult.

Recent advancements in the field have included trials with larger cohorts of high‐risk premature infants, emphasizing both safety and long‐term respiratory outcomes. The focus has shifted to quantifiable metrics, such as the duration of mechanical ventilation, oxygen dependency, and the need for respiratory support after discharge (Table S1) [180, 181]. Long‐term follow‐up remains crucial for evaluating the potential of MSC therapy to mitigate the effects of BPD [181]. A 2020 study administered MSCs to four high‐risk infants and reported a lower incidence of severe BPD among treated infants, with improvements such as reduced reliance on respiratory support [178]. These findings provide initial evidence supporting the potential of MSC therapy in high‐risk populations (Table S1). Overall, MSC therapy holds promise for severe BPD in premature infants. Future trials should include larger populations, assess long‐term safety and efficacy, and determine the optimal dosage, timing, and administration to enhance outcomes and facilitate broader application.

##### 2.3.4.3 COVID‐19

COVID‐19, caused by the coronavirus severe acute respiratory syndrome coronavirus 2 (SARS‐CoV‐2), emerged in late 2019 and primarily affects the respiratory system, although it can also damage other organs. In severe cases, COVID‐19 can trigger a hyperactive immune response known as a “cytokine storm,” leading to acute respiratory distress and organ failure [182]. High‐risk groups, including elderly individuals and individuals with preexisting conditions, are more vulnerable to severe complications.

Clinical trials have investigated the use of MSC therapy for critically ill COVID‐19 patients, focusing on UC‐MSCs owing to their safety profile and ease of accessibility. A systematic review revealed that stem cells are safe and can significantly reduce both mortality and morbidity in COVID‐19 patients. Additionally, stem cell infusion has been shown to improve pulmonary function, alleviate symptoms, and reduce inflammation [183].

One phase 2 trial involved 16 severely ill COVID‐19 patients who received four doses of UC‐MSCs (1 × 10^8^ cells per infusion) to assess the safety and efficacy of MSC therapy. The primary outcomes measured included improvements in oxygenation, reduced progression to critical illness, and decreases in inflammatory markers such as C‐reactive protein and IL‐6 [184]. The phase 1 trial conducted in 2020 highlighted significant clinical improvements in patients receiving MSC therapy compared with the placebo group. By day 14, MSC‐treated patients had improved oxygenation, lower inflammatory marker levels, and no progression to severe respiratory failure, with no significant AEs [185]. A trial involving 12 severe COVID‐19 pneumonia patients treated with UC‐MSCs at a dose of 2 × 10^6^ cells/kg reported similar results, including reduced mechanical ventilation needs and respiratory stabilization, with no patients requiring escalated care [186].

Several studies have shown the potential of MSC therapy to modulate the hyperinflammatory response, improve oxygenation, reduce lung injury and ventilator dependence, and accelerate recovery in critically ill patients [185, 186]. However, the limited scale and short‐term nature of these studies necessitate further investigation. Larger randomized trials with longer follow‐up periods are needed to confirm the efficacy and safety of MSC therapy in patients with COVID‐19. Research should optimize dosing, timing, and delivery (e.g., intravenous vs. inhaled) to enhance outcomes.

##### 2.3.4.4 Chronic Obstructive Pulmonary Disease

COPD affects approximately 300 million people globally and was responsible for 3.23 million deaths in 2019, making it the third leading cause of death worldwide [186, 187]. It is triggered primarily by long‐term exposure to harmful agents such as cigarette smoke and pollutants that cause chronic airway and lung inflammation. This leads to airway narrowing, mucus overproduction, alveolar destruction, impaired gas exchange, airflow obstruction, and trapped air, worsening breathlessness [188]. MSCs may mitigate COPD progression by secreting anti‐inflammatory cytokines and MMP inhibitors, which collectively reduce inflammation and protect the lung structure, supporting improved lung function [189].

A systematic review and meta‐analysis by Liu et al. [190] explored 20 preclinical studies on MSC treatment for COPD‐related lung injuries and showed that MSC administration leads to significant improvements in lung health, as evidenced by metrics such as the mean linear intercept, a measure of lung tissue damage; TUNEL staining, an assessment of cell death in lung tissues; and pulmonary function tests. The meta‐analysis revealed that MSCs effectively alleviated airway inflammation and enhanced anti‐inflammatory cytokine production, facilitating tissue repair and reducing acute lung injury, underscoring the potential of MSCs to mitigate chronic inflammation, promote lung regeneration, and improve lung function [190].

Clinical trials highlight the significant potential of MSC therapy as a treatment for COPD. MSCs have been demonstrated to be safe and well tolerated, with no serious AEs reported in most cases, reduced inflammation, and increased exercise capacity and quality of life. While some studies have shown only modest improvements in lung function and symptom relief, others have reported substantial benefits, particularly in alleviating dyspnea and increasing 6‐min walking test distances (Table S1). Furthermore, combining MSC therapy with advanced techniques such as endobronchial valve placement has synergistic effects, further reinforcing its potential to improve lung function [191].

#### 2.3.5 Cardiovascular Diseases

Cardiovascular diseases (CVDs) remain the leading cause of mortality worldwide. Traditional treatments focus on symptom management rather than repairing heart tissue, highlighting the need for regenerative approaches. Researchers are exploring various stem cell types to address this challenge, with MSCs and iPSCs demonstrating promise (Figure 2). MSCs have shown potential to improve cardiovascular health by promoting angiogenesis, reducing inflammation and scarring (fibrosis), protecting heart cells from death, and enhancing tissue repair. These benefits are achieved through various mechanisms, including paracrine signaling and immune modulation, which ultimately improve overall heart function. While MSCs are currently more widely studied, iPSCs offer a distinct advantage: they can be derived from a patient's own cells and then differentiated into various heart cell types. This personalized approach provides a potential source for regenerating damaged heart tissue while overcoming the challenges of immune rejection and ethical concerns associated with other cell sources [192, 193]. Recent clinical trials have explored the safety, feasibility, and efficacy of MSC and iPSC therapies, offering hope for more effective and lasting treatments, especially for advanced heart disease.

One of the earliest foundational studies was the 2012 phase I/II POSEIDON randomized trial, which confirmed the safety of autologous and allogeneic BM‐MSCs, with no significant AEs within 30 days. While efficacy was not scored, improvements in LVEF and functional capacity encouraged further research [194]. A 2015 phase II trial with 40 severe ischemic heart failure patients demonstrated the safety and efficacy of BM‐MSCs, as they significantly improved LVEF and reduced heart failure readmissions compared with placebo [195]. In 2020, Xiaojun He and colleagues [196] demonstrated that, compared with MSC therapy or coronary artery bypass grafting (CABG) alone, delivery of human UC‐MSCs with a collagen hydrogel scaffold during CABG surgery improved heart tissue preservation, cardiac function, and quality of life, highlighting a promising approach for treating CIHD.

The effects of cardiopoietic cells on chronic heart failure have also been investigated in phase III trials with 315 patients. Although the trial reported only moderate improvements in LVEF and did not fully meet its primary efficacy endpoint, the reduction in adverse cardiac events and improvements in heart function highlighted the potential of MSC therapy, even in populations with advanced heart failure [197].

Advancements in iPSC technology have paved the way for clinical translation. In 2022, Miyagawa et al. [198] conducted a groundbreaking first‐in‐human trial in which allogeneic human iPSC‐derived cardiomyocyte (hiPSC‐CM) patches were used to treat an ischemic cardiomyopathy patient. The implanted patches successfully improved cardiac function, as evidenced by enhanced wall motion, reduced wall stress, and increased myocardial blood flow, highlighting the potential of hiPSC‐CM patches for severe heart failure [198]. Following this work, several clinical trials have been conducted to further elucidate the safety and efficacy of this promising new approach for treating CVDs.

Taken together, these findings indicate that cell therapy holds immense promise for revolutionizing CVD treatment. While MSC‐based therapies have shown encouraging results in improving cardiac function and reducing AEs, iPSC technology offers the unique advantage of generating patient‐specific heart cells, potentially overcoming challenges associated with immune rejection. As research progresses and more clinical data become available, these cell therapies are poised to transform the landscape of CVD treatment, offering hope for improved outcomes and a better quality of life for patients worldwide.

#### 2.3.6 Musculoskeletal Diseases

Musculoskeletal diseases (MSDs) encompass a broad range of conditions affecting joints, bones, muscles, and tendons, such as osteoarthritis (OA), osteoporosis, rheumatoid arthritis, and sports‐related injuries. These disorders are major global health concerns, ranking as the sixth leading cause of years lived with disability (YLDs) in 2020, accounting for 42.7 million YLDs worldwide [199]. Traditional treatments, such as pharmacological therapies and physical rehabilitation, focus primarily on managing pain and inflammation but often fail to address the underlying structural damage and are associated with side effects [200].

MSCs offer a promising and innovative alternative for treating MSDs because of their ability to differentiate into various tissue types, including bone, cartilage, and muscle. Unlike conventional therapies that primarily alleviate symptoms, MSC‐based treatments aim to regenerate damaged tissues, reduce inflammation and promote healing by creating a supportive environment for tissue repair [201].

Over the past decade, MSC therapy has gained prominence as a potential treatment for OA, with recent studies further exploring its potential and challenges (Table S3). Despite reported mild and temporary effects, such as pain and swelling with injection, MSC therapy remains a safe and less invasive alternative to surgery for OA treatment [202]. A phase III trial with AT‐MSCs demonstrated significant improvements in pain and functionality at 6 months. Furthermore, “Cellistem” trials utilizing UC‐MSCs have shown their efficacy in reducing inflammation and enhancing joint function [203]. Research has consistently confirmed the short‐term benefits of MSC therapy for OA, including reduced pain, improved joint mobility, and decreased inflammation, which are often evident as early as 3 months posttreatment (Table S3) [204, 205, 206]. Cellistem is currently approved for commercial use in Chile and New Zealand for the treatment of knee OA. Cartistem, which utilizes UC‐MSCs and has shown significant success in improving joint function and alleviating pain, has been approved in South Korea for treating knee cartilage defects [207, 208]. In Europe, two additional therapies, Spherox [209] and Maci [210], offer approved options for cartilage repair, highlighting the potential of UC‐MSCs as a viable option for short‐term OA management.

Despite promising short‐term outcomes, the long‐term benefits of stem cell therapy remain inconsistent, with efficacy often tapering after 12 months. For example, studies on AT‐MSCs for OA show initial improvements in WOMAC scores that decline between 6 and 12 months [211]. This decline highlights the need to investigate factors affecting long‐term efficacy, such as the optimal dosage and the durability of stem cell activity within the joint environment.

Preclinical studies utilizing gene editing technologies, such as CRISPR/Cas9, have shown significant promise in treating MSDs. Researchers have successfully used CRISPR to correct gene mutations responsible for cleidocranial dysplasia and Duchenne muscular dystrophy (DMD) in patient‐derived iPSCs, restoring normal bone formation and dystrophin protein expression, respectively [212, 213]. Additionally, CRISPR has shown potential in targeting mutations in iPSC‐derived HSCs for lysosomal storage diseases with musculoskeletal manifestations [214]. These encouraging preclinical results pave the way for future clinical trials investigating gene editing as a novel therapeutic strategy for MSDs, offering hope for improved treatments and patient outcomes.

Collectively, advancements in MSC‐based therapies, especially for OA, show promise in addressing musculoskeletal disorders by providing pain relief, improving joint mobility, and reducing inflammation. However, long‐term efficacy and optimized treatment strategies remain key challenges. Emerging technologies such as CRISPR/Cas9 further increase the potential for innovative and effective MSD treatments, offering hope for improved patient outcomes.

#### 2.3.7 Reproductive Diseases

Reproductive disorders are diseases of the reproductive system. Cell therapy has been studied for reproductive diseases and infertility. To date, clinical studies have applied MSCs and other stem cells to treat primary ovarian deficiency (POI) (also known as primary ovarian failure (POF)) and Asherman's syndrome, known as ovarian and uterine disorders, respectively.

##### 2.3.7.1 POI/POF

POI describes an early loss of ovarian function in women younger than 40 years. The disease results in decreased estrogen levels, a lack of normal egg development, and infertility in the majority of affected women. The etiology and pathogenesis of POF are complex. Genetic defects, such as Turner syndrome or fragile X syndrome in women with only one X chromosome or those with fragile X chromosomes, are often associated with POF [215, 216]. Other pathogenic genetic variants affecting ovarian development and function from gonadogenesis to folliculogenesis and ovulation have recently been reported [217]. In addition to genetic factors, exposure to toxins and environmental factors, viral and bacterial infection, cancer treatment via radiation, chemotherapy, and pelvic surgery are common causes of POF [218, 219]. Autoimmune disorders, characterized by positivity of autoantibodies against the ovary, abnormal levels of cytokines, and dysfunction of Treg cells and Th17 cells, are also known risk factors for POF [220].

Cell therapy might act to improve POI through several mechanisms. PSCs, including female germline stem cells and iPSCs, can differentiate into several ovarian cell types, such as oocytes, estrogen‐sensitive epithelial‐like cells, and granulosa‐like cells [221, 222, 223]. These cells are functionally active, produce estrogen, and promote follicular development. MSCs, while less potent in terms of their differentiation capacity, are able to migrate to inflammatory sites and rescue the compromised cellular environment [224]. MSCs support ovarian function by secreting growth factors, modulating the immune system, reducing oxidative stress, stimulating mitochondrial transfer, promoting angiogenesis, and enhancing ovarian cell survival [225].

Treatment of POI with MSCs has been shown to improve sex hormone levels and ovarian follicle development in female animals [226, 227]. Many studies have investigated the ability of PSCs, including ESCs and iPSCs, and adult stem cells, including MSCs from diverse sources, such as BM, AT, menstrual blood, UC, amniotic fluid, the amniotic membrane, the placenta, and the endometrium, to regenerate damaged ovaries and oocytes [228]. While research on ESCs and iPSCs has been limited to in vitro differentiation and in vivo evaluation in animal models of POI [221, 229], primary clinical data on the safety and efficacy of therapeutic MSCs have been reported (Table S4).

Overall, studies of cell therapy for POI treatment are still in an early phase, with few patients and randomized controlled results. All studies involved intraovarian injection and reported no AEs. Approximately 5–10% of women with POI can naturally conceive [230], whereas cell therapy resulted in different rates of natural conceptions, such as three out of 15 (20%) after BM‐MNC treatment [231], two out of 14 (14%) and four out of 61 (7%) in two UC‐MSC studies [232, 233], and three out of 15 (20%) after menstrual blood‐derived MSC injection (compared with zero out of 16 in the control group) [234].

Cell therapy may enhance other treatments, such as ovarian autotransplantation, to preserve fertility in women with cancer. As cryopreservation and grafting of ovarian tissue often lead to follicle death due to hypoxia and a lack of blood vessels, stimulating revascularization with proangiogenic factors or cell therapy could improve outcomes. MSCs have been shown to support blood vessel formation around grafted ovarian tissue and increase follicle survival [235].

Hence, the efficacy of MSC therapy remains to be further investigated with more advanced control studies. Many issues need to be addressed: the best cell sources, impact of potential genetic/epigenetic modifications, licensing of therapeutic cell products, optimal cell dose and efficacy of repeat dosing, concomitant treatments, and use of biomaterials and scaffolds for cell therapy.

##### 2.3.7.2 Asherman's Syndrome and Endometrial Atrophy

Asherman's syndrome and endometrial atrophy are disorders of the uterus. Asherman's syndrome occurs when scar tissue is formed in the uterine cavity, causing intrauterine adhesions [236], whereas endometrial atrophy is characterized by decreased thickness of the endometrium, the inner epithelial layer of the uterus [237]. Both diseases prevent implantation of fertilized eggs, resulting in infertility. Some studies have explored cell therapy for refractory cases (Table S4). Singh et al. [238] reported in a 5‐year follow‐up study a short‐term increase in endometrial thickness after intrataurine injection of BM‐MNCs combined with hormone therapy (*n* = 25). Six of seven amenorrhea women experienced menses again, and three had successful pregnancies [238]. Similarly, cell therapy using autologous menstrual blood‐derived MSCs and the adipose‐derived stromal vascular fraction improved endometrial thickness [239, 240]. Ma et al. [240] reported a natural conception and four conceptions via embryo transfer in twelve patients (42% in total) treated with menstrual blood‐derived MSCs.

Two studies explored the use of a collagen scaffold combined with UC‐MSCs for intrauterine injection in patients with recurrent Asherman's syndrome. In a study of twenty‐six patients, therapy increased endometrial thickness and decreased the intrauterine adhesion score, which was correlated with improved endometrial proliferation and neovascularization [241]. Ten patients (38%) became pregnant within a 30‐month follow‐up, with eight successful pregnancies. These results are supported by a recent study in which endometrial thickness increased 3 months after UC‐MSC/collagen treatment [242]. Four of the eighteen women became pregnant, including one natural conception and three successful embryo transfer conceptions that resulted in the birth of three healthy babies.

Furthermore, cell therapies, such as the use of MSCs from AT, have been explored for their ability to manage sexual dysfunction in both females and males because of their ability to enhance the endocrine function of the ovary and Leydig cells [243]. This approach could represent a novel method to complement conventional treatment with sex hormone replacement therapy. However, the efficacy of this method remains to be tested.

### 2.4 Current Challenges of Cell Therapy

Cell therapy has immense therapeutic potential, but its development faces significant challenges across multiple domains: clinical, scientific, ethical, and regulatory.

#### 2.4.1 Challenges of MSC Therapy

Among various cell therapies, MSC therapy is the most prevalent in regenerative medicine. Despite decades of research with promising outcomes, clinical advancements have progressed slowly and remain relatively stagnant. Commercially approved MSC products remain limited. Most clinical trials involving MSC therapy remain in phases 1 and 2, with few advancing to phases 3 and 4, and, overall, a relatively low percentage of responses has been observed [244]. There are several challenges associated with cell therapy that should be addressed.

##### 2.4.1.1 Heterogeneity of MSCs

The heterogeneity of MSCs arises from factors such as tissue origin, donor variability, and cell isolation and culture methods, leading to phenotypic and functional differences. MSCs from different sources exhibit distinct characteristics, including surface markers, proliferation rates, differentiation potentials, and immunomodulatory properties. This variability complicates standardization, therapeutic prediction, dosing optimization, and quality control (QC) for clinical use [245, 246]. One possible solution is a matching strategy, as hypothesized by Hoang et al. [5]. This approach emphasizes selection of MSCs based on their tissue origin to enhance therapeutic efficacy for specific conditions. For example, BM‐MSCs might show promise for neurological injuries because of their neuroregenerative potential, AT‐MSCs might be better suited for reproductive and skin conditions, and UC‐MSCs might be advantageous for pulmonary diseases because of their strong immunomodulatory and proliferative abilities [5]. Taken together, the complexity of diseases, cell responses, and interactions with the microenvironment underscore the need for ongoing research to identify the most effective MSC types for different clinical applications.

##### 2.4.1.2 Challenges in Clinical Applications

The biodistribution and fate of MSCs in the human body remain unclear because of challenges in tracking them post administration. Understanding how MSCs interact with injured microenvironments further complicates their study. Additionally, species‐specific biological differences often hinder the translation of preclinical successes into clinical efficacy, posing a major obstacle to advancing MSC‐based therapies [247].

The lack of standardized protocols for timing, dosing, and delivery routes also complicates the comparison of trial outcomes and the optimization of treatment strategies. Efficient stem cell delivery is particularly challenging for neurodegenerative diseases, as the blood–brain barrier (BBB) limits cell homing to the brain, and even when successfully delivered, poor survival and limited migration into host tissues diminish therapeutic efficacy. Additionally, long‐term safety concerns, such as delayed AEs or disease recurrence, remain unresolved. Variability in study designs, methodological rigor, and outcome measures further hampers reliable assessment of safety and efficacy, underscoring the need for standardized evaluation methods [248].

Stem cell therapies face significant challenges, including strict regulatory scrutiny as advanced therapy medicine products and the high cost of producing GMP‐compliant clinical‐grade stem cells. These factors delay clinical translation and limit accessibility, posing barriers to widespread adoption.

##### 2.4.1.3 Potential Complications of MSC Therapy

One significant issue is the occurrence of an instant blood‐mediated inflammatory reaction (IBMIR) following the systemic administration of cellular products, which rarely occurs but might result in severe complications. Moll et al. [249] reported that in vitro culture of MSCs significantly increased tissue factor (TF) expression on their surface, causing IBMIR and severe outcomes in vivo. Upon intravascular infusion, TF, which acts as an initiator of the extrinsic coagulation cascade, becomes exposed to plasma coagulant factors, thereby activating them and triggering thrombi formation [250, 251]. Consequently, intravascular infusion of MSCs without appropriate anticoagulant prophylaxis can lead to mild‐to‐severe thromboembolic events [252]. Other factors, such as the presence of phosphatidylserine on the surface of MSCs, also contribute to thrombotic events [253]. Recently, Moll et al. [250] suggested improving MSC safety criteria by assessing TF levels and hemocompatibility of MSC products. Therefore, it is essential to consider these detrimental effects and develop strategies to mitigate the incidence of IBMIR and thrombosis to ensure safe cell‐based therapies.

Furthermore, MSCs have been implicated in promoting tumor growth under certain conditions [254]. Conversely, MSCs are being explored as powerful therapeutic tools for treating cancer [255]. This dual role of MSCs raises concerns when MSC therapy is used for the treatment of diseases, and more studies with long‐term follow‐up are needed. Finally, the use of allogeneic stem cells introduces immunological risks. Rejection by the host immune system can necessitate immunosuppression, which introduces its own set of complications, including increased susceptibility to infections.

#### 2.4.2 Challenges of PSC Therapy

There have been several challenges associated with PSC therapy [57, 256]. ESCs and iPSCs carry the risk of tumor formation, particularly teratomas, if their differentiation is not carefully controlled. Ensuring precise differentiation into the desired cell types is a critical challenge, as incomplete or incorrect differentiation can lead to ineffective or harmful outcomes [98, 247]. Contamination of undifferentiated ES cells could promote tumorigenesis [257]. Tumorigenicity may also be caused when reprogramming factors remain active in the final cell product or if genetic mutations occur during in vitro culture [57, 256]. Additionally, the genomic integration of viral vectors used in reprogramming raises safety concerns. Some viral tools, such as retroviral and lentiviral vectors, can integrate into the host genome. This integration may lead to mutations or the activation of oncogenes [258, 259]. Maintaining cell stemness and ensuring their potency throughout expansion is particularly challenging. Variability in protocols between laboratories often leads to inconsistent outcomes, while cell heterogeneity further complicates QC, making therapeutic efficacy less predictable.

The generation of iPSCs is costly and takes a long time [260, 261]. A major issue of iPSC therapy is low reprogramming and differentiation efficiency. Converting somatic cells into iPSCs remains a labor‐intensive process, with only a small percentage of cells successfully reprogrammed. The efficiency of reprogramming can vary significantly depending on the method used and the type of cells. Although nonintegrating methods have been developed, they remain less efficient or not as widely adopted [258, 259]. Another key challenge is epigenetic memory, where iPSCs retain epigenetic markers from their somatic origins. These residual markers can affect the cell differentiation potential and stability, potentially leading to biased differentiation or preventing the cells from achieving full pluripotency. Genomic instability is also a concern, as iPSCs can accumulate chromosomal abnormalities. These mutations may compromise the safety of iPSCs in clinical applications and increase the risk of tumorigenicity [262, 263].

Heterogeneity is a significant challenge in PSCs because of variations in morphology, growth patterns, gene expression, and differentiation potential across lines. These differences, which are influenced by genetic and epigenetic factors, complicate downstream applications such as cell therapy [57].

Finally, the ethical and regulatory issues surrounding iPSC technology must be considered. Although iPSCs bypass the ethical concerns associated with embryonic stem cells, issues related to gene editing, tissue engineering, and the potential for creating genetically modified organisms remain [264]. Moreover, regulatory frameworks for iPSC‐based therapies are still evolving, and navigating these regulations while ensuring patient safety is a complex process. Coupled with these ethical concerns are the issues of cost and accessibility. Generating iPSCs, maintaining them in culture, and ensuring their safety and efficacy are high‐cost endeavors, limiting access to this technology, especially for less resource‐rich settings [265].

#### 2.4.3 Challenges of NSC Therapy

NSCs are obtained primarily from embryonic brains, which raises ethical concerns. Current advances in technology now allow the generation of NSCs through differentiation from ESCs and iPSCs and transdifferentiation from somatic cells. However, the methods also present safety challenges that complicate the translation of these techniques into effective clinical therapies [266]. Induced NSCs (iNSCs) can be derived directly from a patient's tissue, bypassing the challenges associated with the pluripotency stage of iPSCs while recapitulating the properties and therapeutic potential of true NSCs. Autologous iNSCs offer the advantage of minimizing immunogenicity, but their therapeutic utility may be compromised by underlying genetic defects, as observed in conditions such as multiple sclerosis [267]. Additionally, while iNSCs demonstrate key features of adult NSCs, including their ability to differentiate into cortical neurons and show therapeutic benefits in AD models, significant challenges remain. Studies indicate that iNSCs predominantly differentiate into cortical glutamatergic neurons by default and exhibit limited capacity for controlled in vivo behavior, including survival, differentiation, migration, and functional integration. Additionally, there is insufficient evidence supporting their ability to replace the diverse neuronal subtypes lost in various disease conditions. Addressing these limitations will require advances in reprogramming methods, differentiation protocols, and therapeutic strategies to unlock the full clinical potential of iNSCs [268].

### 2.5 Strategies to Overcome Challenges

#### 2.5.1 Strategies for MSC Therapy

The priming of MSCs with small‐molecule cytokines, hypoxia, and 3D culture has been conducted to increase the secretion of immunomodulatory and regenerative molecules and the therapeutic efficacy and long‐term survival of these cells [36, 269]. Pretreatment of MSCs with melatonin improved the survival of transplanted AT‐MSCs and better preserved the cognitive, learning, and memory functions of Aβ‐treated AD rats [270]. The overexpression of important biomarkers, such as neurotrophic factors and miRNAs, with gene technology prior to transplantation might help increase survival and function [271]. For example, MSCs overexpressing CX3CL1 and Wnt3a, as well as microRNA‐modified MSCs, improve cognitive function and reduce neurotoxicity in AD [98]. Genetic manipulation protects stem cell‐derived islets for T1DM treatment from stress‐mediated apoptosis and dysfunction [272]. Additionally, incorporating MSCs into hydrogels or microgels to mimic their natural microenvironment significantly increased their residence time in vivo [36]. The use of biomaterials such as 3D scaffolds can also help improve cell survival and engraftment [89].

To reduce immune rejection, coupling cell administration with systemic immunosuppression and performing HLA assessments of allogeneic MSC therapies are crucial [273]. Building HLA data for MSC banks and screening patients can enable better HLA matching, minimizing rejection risks. Genetic engineering of implanted cells to evade immune recognition or to secrete immune modulatory cytokines can also be under investigation for reducing immune rejection [9]. Another strategy is to enclose the graft within a selectively permeable barrier that enables the exchange of nutrients and therapeutic molecules, such as insulin, while blocking immune components that could cause rejection.

In clinical practice, the identification of biomarkers or biological indicators may help predict patient responsiveness to therapy and select those who are most likely to benefit. Furthermore, methods to enhance MSC homing to target organs might improve clinical outcomes. Focused ultrasound temporarily opens the BBB, facilitating the entry of therapeutic molecules and promoting MSC migration to the brain. It also upregulates adhesion molecules such as VCAM‐1 and ICAM‐1, increasing MSC accumulation in targeted brain regions [58, 98]. Finally, the development of effective methods for tracking cells in humans is vital for exploring their biodistribution [274]. Understanding the pharmacokinetics and pharmacodynamics of MSCs is crucial for developing more potent therapeutic cellular products [274].

#### 2.5.2 Strategies for PSC Therapy

While iPSC technology is promising, it is still in the early stages, with a few clinical trials being conducted. Several strategies have also been discussed to increase the safety of iPSC products, including (1) the generation of safe iPSCs via advanced techniques for cell reprogramming, (2) the selection of pure differentiated cells, (3) the depletion of contaminant PSCs in the final cell product, and (4) the control of tumorigenic cells with suicide genes [275].

Several strategies are being implemented to overcome the challenge of low reprogramming efficiency. Optimizing reprogramming methods has led to more efficient protocols, including using small molecules or nonintegrating vectors, such as Sendai virus, mRNA, or protein‐based methods. These approaches eliminate the risks associated with viral integration, although they can sometimes be less efficient. Researchers are also working on improving the precision of reprogramming by tailoring the factors used, adjusting concentrations, and experimenting with new techniques to increase efficiency [276]. Additionally, nonintegrating methods are being improved and becoming more widely adopted, albeit with some compromises in efficiency [276]. Automating and streamlining the reprogramming process is another avenue being explored to increase the success rate and reduce the time required to generate iPSCs [277].

Uncontrolled tissue growth, such as teratoma formation, poses a major challenge in PSC therapies. Safeguards such as molecular “off switches” and precise growth‐regulatory mechanisms, including insights from the Hippo signaling pathway, are crucial for ensuring safety [9]. Advances in gene editing tools such as CRISPR–Cas9 have allowed the correction of genetic defects in donor cells as well as the modification of cells to reduce tumorigenicity and improve their therapeutic properties [278]. Furthermore, the establishment of efficient methods of in vitro directed differentiation is another important step in reducing the risk of teratoma. Various strategies have been developed to eliminate residual PSCs or increase the purity of differentiated PSC preparations to mitigate teratoma risk in cell therapy. These include the use of endogenous markers such as SSEA‐4 and TRA‐1‐60 for cell sorting, the exploitation of cell density gradients, and the leveraging of cytotoxic antibodies such as those targeting podocalyxin‐like protein‐1. These approaches provide viable methods for ensuring safer PSC‐based therapies [57].

Immunosuppressants prevent allograft rejection but often require lifelong use, risking severe side effects. In immune‐privileged tissues, therapy may be temporary, but trauma, disease, or aging can compromise this privilege, necessitating lifelong immunosuppression for nonprivileged tissues. Matching of HLA haplotypes is another method used to reduce rejection. Recently, gene editing technologies, particularly CRISPR, have enabled innovative strategies for creating universal PSC lines through HLA cloaking. This involves inactivating HLA genes to reduce immune rejection while addressing NK cell activation via strategies such as introducing HLA‐E or retaining HLA‐C molecules. Approaches such as B2 M deletion with HLA‐E expression or selective deletion of HLA‐A and HLA‐B alleles with intact HLA‐C offer promising solutions for generating universal donor cell lines [57].

To overcome the heterogeneity of PSCs, naive‐state conversion from a “primed” state to a “naive” state can be achieved in human PSCs via the use of inhibitors, such as ROCK, BRAF, and SRC, in the presence of activin and hLIF. However, concerns about genetic integrity and imprinting loss in naive human PSCs must be addressed to optimize their clinical utility [57].

With respect to ethical and regulatory concerns, clearer ethical guidelines are being developed to govern the use of iPSCs in research and therapy. Regulatory agencies are also establishing frameworks for clinical trials involving iPSCs, ensuring that therapies are safe and effective. Public engagement and transparency are essential in addressing concerns about gene editing, tissue engineering, and the potential misuse of iPSC technology [264]. Finally, the issues of cost and accessibility are being addressed through collaborative efforts among industry, academia, and governments. These partnerships aim to subsidize the costs of iPSC research and ensure that it becomes more widely accessible, even in less resource‐rich settings [279, 280].

#### 2.5.3 Strategies for NSC Therapy

In addition to the common strategies mentioned, improving the purity of NSCs is a priority, as the contamination of other cells may cause unexpected side effects. Deep sequencing and evaluation of the tumor formation potential of many manufactured NSC products and advanced imaging techniques are needed to monitor the physiological state of transplanted NSCs in vivo to exclude tumorigenicity and other pitfalls. While the main mechanisms of NSCs and MSCs involve cell replacement and paracrine effects, respectively, together with evidence from preclinical trials showing that the combination of MSCs and NSCs has better effects, it is anticipated that NSC‐based therapy with or without MSCs will be a major direction for the treatment of nerve diseases in the future [266].

Advancements in NSC therapy, driven by emerging technologies and stem cell research, now enable the generation of high‐quality NSCs from iPSCs or via somatic cell transdifferentiation. Genetic modifications to overexpress growth factors involved in neurogenesis and synaptogenesis have shown promise. Neurotrophin‐overexpressing NSCs have improved survival and neuroprotective properties in various neurological disease models [281]. For example, GDNF‐overexpressing NSCs effectively migrated to disease sites and integrated into the CNS in an ALS spinal cord model [282]. Similarly, GDNF‐expressing hiPSC‐NSCs exhibited enhanced neuronal differentiation compared with controls in a rodent model of cervical SCI [283].

Genetic engineering can also drive NSC differentiation, increasing therapeutic benefits. Wnt4‐overexpressing NSCs promote a neural phenotype, improving injury repair and functional integration [284]. Additionally, modifying NSCs to express deficient neurotransmitters in neurodegenerative diseases has yielded encouraging results. For example, hNSCs overexpressing the acetylcholine‐producing enzyme ChAT restored cholinergic circuits and significantly enhanced cognitive function and physical activity when transplanted into the aged rodent CNS [285]. These strategies highlight the transformative potential of genetic modifications in optimizing NSC‐based therapies.

### 2.6 Future Perspectives

The future of cell therapy holds significant potential. The development of personalized cell therapies, where treatments are tailored to the specific needs of individual patients, is a key direction. This approach could reduce the risk of immune rejection and enhance treatment outcomes.

Genetic modifications and preconditioning represent important advances for improving the effectiveness of stem cell therapy. Engineered stem cells can secrete trophic, anti‐inflammatory, and survival factors, continuously releasing neurotrophic factors to create a supportive microenvironment that promotes nerve regeneration and protection when introduced into damaged areas of the nervous system. CRISPR–Cas9 and other revolutionary genome‐editing tools enable researchers to modify HLA complexes in donor stem cells, making them immune compatible with recipients. This reduces immunological barriers, promotes successful integration, and enhances transplantation outcomes. Beyond transplantation, CRISPR–Cas9 has transformative potential for treating degenerative diseases such as AD, Huntington's disease, CVDs, and diabetes, paving the way for targeted and precise therapeutic interventions. Artificial intelligence and big data integration will play critical roles in refining stem cell research, optimizing differentiation protocols, and improving clinical trial design. There is increasing interest in “off‐the‐shelf” allogeneic stem cell products, which could make cell therapy more accessible and cost‐effective. These therapies aim to provide ready‐to‐use cell products that do not require patient‐specific customization. However, issues related to immune compatibility and safety must still be addressed.

The use of cell‐free therapies, such as stem cell‐derived exosomes, offers an alternative approach that overcomes risks such as tumorigenicity and immune rejection associated with live cell transplantation. Furthermore, technologies such as 3D bioprinting and tissue engineering are paving the way for the regeneration of complex tissues and even entire organs, potentially addressing issues beyond cell therapy.

## 3 EV‐Based Therapy: Applications and Challenges

**FIGURE 4:**
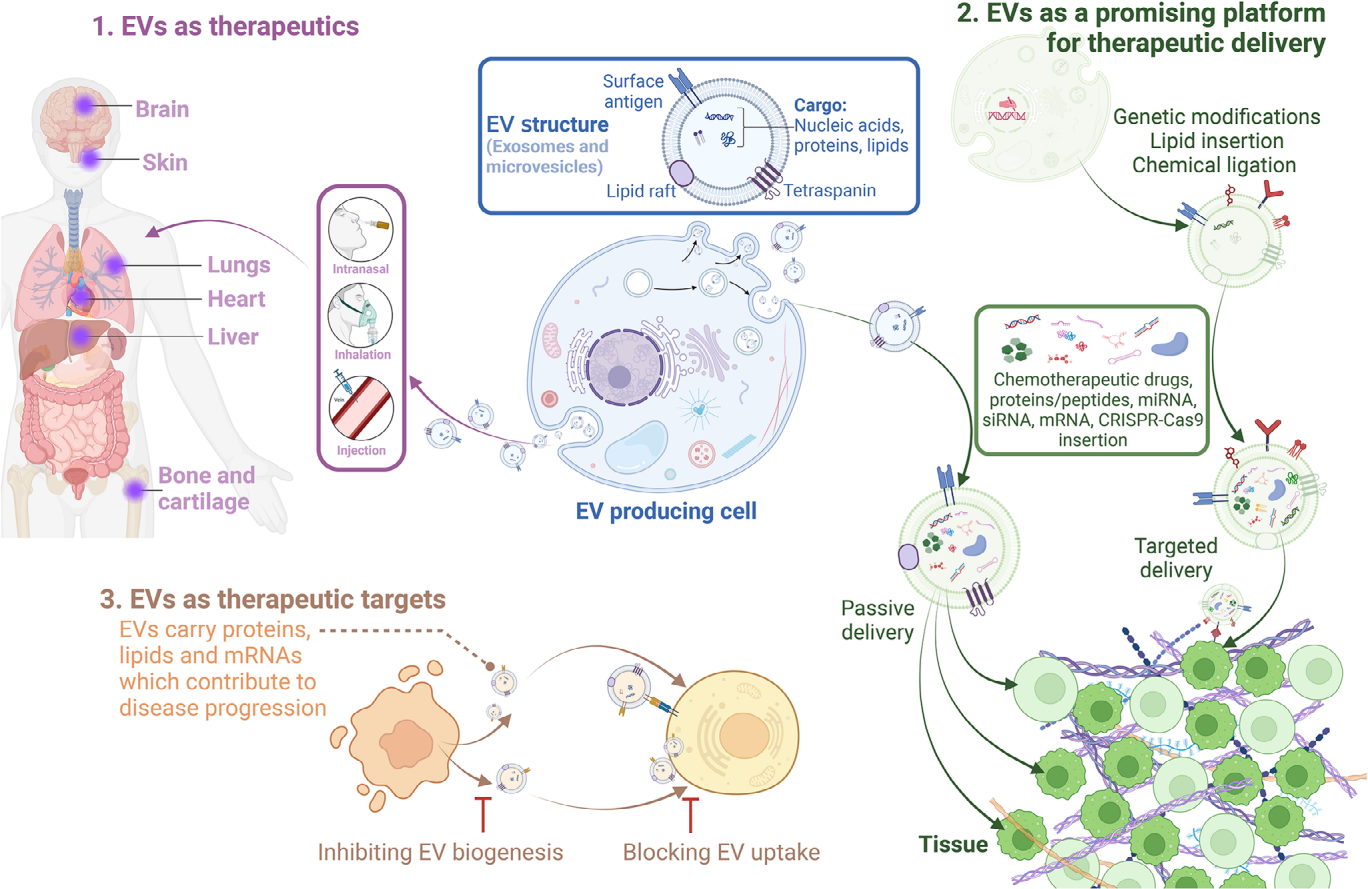
Potential roles of EVs in therapeutic applications for regenerative medicine. (1) EVs can be used as cell‐free therapeutics for treating various degenerative diseases related to the nervous system, liver, lungs, heart, bone, cartilage, and skin by enhancing tissue regeneration and repair. (2) EVs hold promise as platforms for the passive delivery of therapeutic molecules. Alternatively, surface modifications of EVs with targeting ligands facilitate the targeted delivery of therapeutics to specific tissues, minimizing off‐target effects and toxicity. (3) EVs serve as therapeutic targets to slow disease progression. By inhibiting EV biogenesis and release or blocking EV uptake, the transfer of pathogenic components from pathological EVs to healthy cells can be prevented. *Abbreviations*: clustered regularly interspaced short palindromic repeats and CRISPR‐associated protein 9 (CRISPR–Cas9), extracellular vesicles (EVs), messenger RNA (mRNA), and small interfering RNA (siRNA).

EVs have recently gained significant attention as cell‐free therapeutic agents, showing promising potential for future applications in regenerative medicine. EVs play crucial roles in cellular communication and the transfer of diverse bioactive molecules. Owing to their unique ability to encapsulate and deliver cargo between cells, EVs are powerful tools for regenerative medicine and therapeutic development. Research has demonstrated that EVs contribute to the therapeutic functions of cells by promoting the proliferation and migration of effector cells, supporting angiogenesis, modulating immune responses, and performing specialized tasks in various tissues (Figure 4). Current preclinical studies underscore the broad potential of EV‐based therapies for repairing and regenerating damaged tissues across a range of conditions, including cardiac, pulmonary, hepatic, bone, skin, and nerve regeneration. Advances in biomedical engineering have facilitated the development of techniques to enhance the capabilities of EVs for more targeted and efficient therapies. These techniques involve loading therapeutic agents or genetic material into EVs to augment their function. To improve their targeting ability, EV membranes are modified such that they can bind to specific cell types or tissues. Additionally, EVs are recognized not only as therapeutic agents but also as potential therapeutic targets for combating disease progression. By focusing on the processes of EV biogenesis, release, or uptake, we can mitigate the detrimental effects these vesicles may have on healthy tissues and organs.

### 3.1 The Fundamental Concepts of EV‐Based Therapy

EVs are lipid bilayer nanoparticles ranging from 30 to 5000 nm in size [286]. In contrast to the initial belief that EVs were cellular waste, EVs are now widely recognized as crucial vehicles for intercellular transport [286]. EVs are present in the culture media of various cell types across all organisms, from bacteria to humans, and are detected in various body fluids, such as blood, urine, saliva, serum, and breast milk [286, 287]. Owing to their membrane‐enclosed structure, EVs allow cells to safely exchange information and molecules in harsh and degrading environments [288]. These nanostructures are filled with diverse bioactive cargos, including proteins, nucleic acids, lipids, and metabolites, which vary depending on the cell type and physiological conditions [286, 289]. These biomolecules can be transferred to target cells, influencing their behavior and function in many biological processes, such as cell maintenance, stimulation of immune responses and disease progression [286, 288].

EV biogenesis is a complex process that varies depending on the cell type and the type of EV being produced and can be generally classified into two main pathways: endosomal sorting complex required for transport (ESCRT)‐dependent and ESCRT‐independent [290]. In the ESCRT‐dependent pathway, a group of ESCRT subcomplexes (TSG101, CHMP proteins) take part in and are activated step‐by‐step [290]. ESCRT‐independent pathways create membrane subdomains and tetraspanins (CD63, CD81, and CD9) to form groups on membranes and induce inward budding of vesicles, which eventually form EVs [289]. However, the specific regulatory mechanism of EV formation and release requires further research [286, 289, 290].

Owing to their ability to form, release, size, carry, and function, EVs can be categorized into three distinct main subtypes: microvesicles (MVs), exosomes, and apoptotic bodies [286, 289]. Exosomes are EVs that are typically 30–150 nm in diameter [286, 287]. They are formed by the inward budding of the plasma membrane of the early endocytic pathway and then mature into multivesicular bodies (MVBs) and trafficking functions of the cell's material [291]. MVBs are eventually degraded with all their components by the lysosome or fused with the plasma membrane to release their contents into the extracellular environment, including exosomes [291]. Compared with exosomes, MVs range from 100 to 1000 nm in size and are formed by direct shedding from the cell plasma membrane [289]. Therefore, MVs contain mainly cytosolic and plasma membrane‐associated proteins [286, 289]. Other identified proteins packaged in MVs include cytoskeletal proteins, heat shock proteins, integrins, and proteins containing posttranslational modifications, such as phosphorylation and glycosylation [289]. They can be released in response to various stimuli, such as stress, inflammation, and cell injury, through both local and distance signaling [292]. With sizes ranging between 1000 and 5000 nm, apoptotic bodies are larger vesicles formed from dying cells during programmed cell death [286, 290]. Unlike exosomes and MVs, apoptotic vesicles contain cellular debris, including organelles, nuclear fragments, and glycosylated proteins [289, 290]. They are primarily involved in the clearance of dying cells and the prevention of inflammation [286]. Compared with that of exosomes, the protein composition of apoptotic bodies is quite similar to that of cell lysates [289]. Although apoptotic bodies are considered cell debris, recent studies have implicated them in intercellular communication [293]. In this review, the term EV(s) specifically refers to exosomes or MVs, excluding apoptotic bodies.

### 3.2 EV Applications in Regenerative Medicine

#### 3.2.1 EVs as Cell‐Free Therapeutics

**FIGURE 5:**
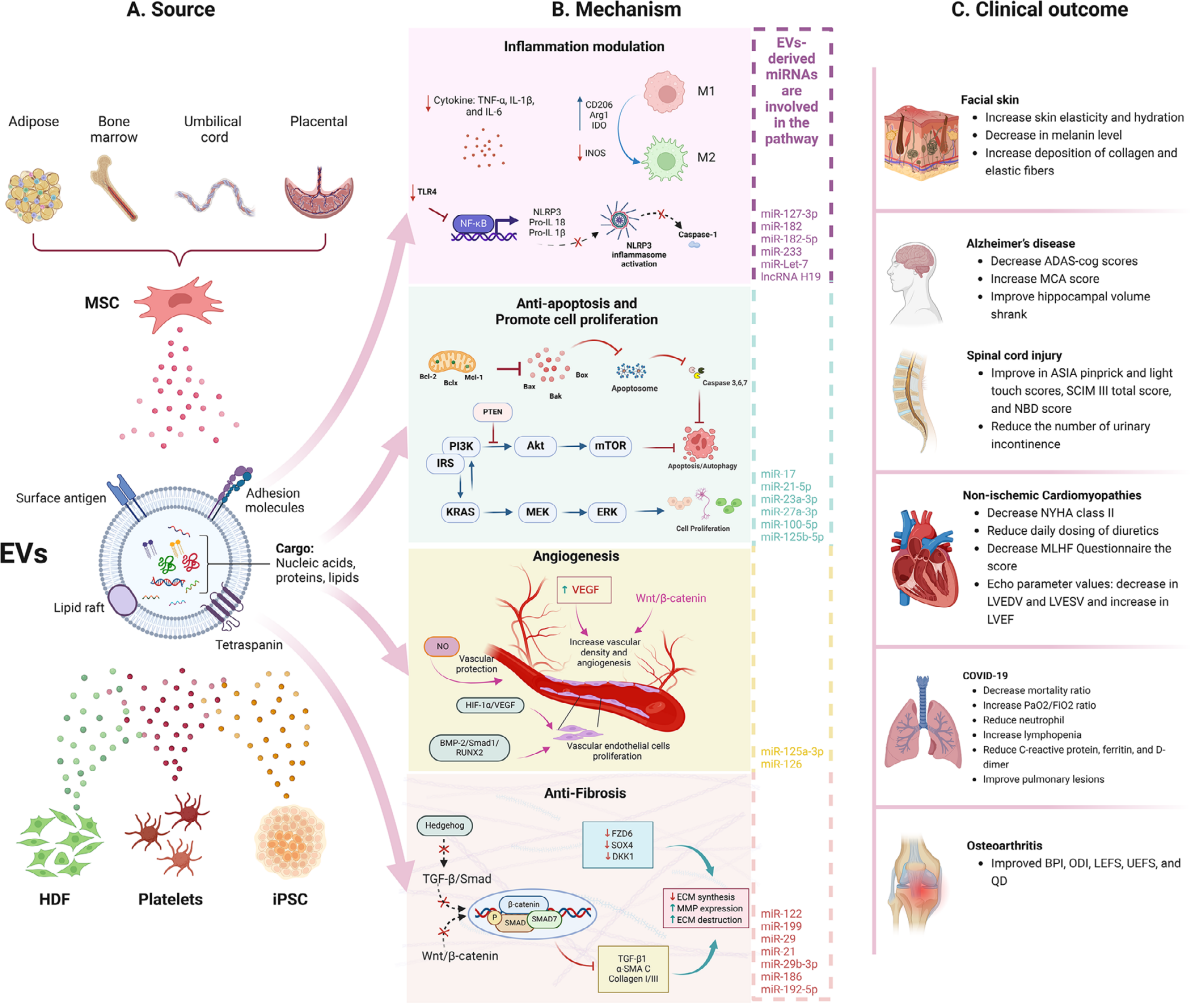
Schematic summary of EVs‐based treatment in regenerative medicine. (A) EVs derived from various cell types, including MSCs, human dermal fibroblasts, and iPSCs, deliver bioactive molecules such as nucleic acids, proteins, lipids, and membrane receptors to target cells, thereby enabling diverse biological functions. (B) The biological roles of EVs in regenerative medicine encompass immunomodulation, anti‐apoptosis, angiogenesis promotion, and cell proliferation stimulation. EVs regulate inflammation by reducing proinflammatory cytokines (e.g., TNF‐α, IL‐1β, and IL‐6), inducing M2 macrophage polarization through markers like iNOS, CD206, and Arg1, and inhibiting NLRP3 inflammasome activation via key pathways such as NF‐κB. They protect cells from apoptosis by upregulating antiapoptotic proteins (e.g., Bcl2 and caspase) and suppressing proapoptotic factors (e.g., Bax), while also regulating autophagy through the PTEN/PI3K/AKT pathway. Additionally, EVs promote cell proliferation via the ERK pathway, enhancing the growth of various cell types such as Schwann cells, hepatocytes, and cardiomyocytes. In vascular protection, EVs enhance endothelial cell populations and stimulate new capillary formation. Furthermore, EVs suppress fibrogenic signaling pathways like TGF‐β/Smad and Wnt/β‐catenin, downregulate ECM proteins (e.g., collagen I and fibronectin), promote MMPs, and reduce profibrotic factors (e.g., TGF‐β1, α‐SMA, and collagen I/III). Notably, specific miRNAs within EVs play a critical role in enhancing these biological functions. (C) Therapeutic applications of EVs span a variety of conditions, including facial skin disorders, Alzheimer's disease, spinal cord injuries, nonischemic cardiomyopathies, COVID‐19, and osteoarthritis. *Abbreviations*: Alzheimer's disease assessment scale‐cognitive section scores (ADAS‐cog), v‐akt murine thymoma viral oncogene homolog (AKT), arginase 1 (Arg1), American spinal injury association scale (ASIA), B‐cell leukemia/lymphoma 2 (bcl2), brief pain inventory (BPI), extracellular matrix (ECM), extracellular signal‐regulated kinase (ERK), extracellular vesicles (EVs), interleukin (IL), inducible nitric oxide synthase (iNOS), lower extremity functional scale (LEFS), mesenchymal stem cells (MSCs), Montreal cognitive assessment scores (MCA), Minnesota living with heart failure (MLHF), matrix metalloproteinases (MMPs), microRNAs (miRNAs), nuclear factor kappa‐light‐chain‐enhancer of activated B cells (NF‐κB), nucleotide‐binding domain, leucine‐rich–containing family, pyrin domain–containing‐3 (NLRP3), nitric oxide (NO), neurogenic bowel dysfunction (NBD), New York Heart Association (NYHA), induced pluripotent stem cells (iPSCs), Oswestry Disability Index (ODI), phosphatidylinositol 3‐kinase (PI3K), phosphatase and tensin homolog chromosome 10 (PTEN), quick dash scale (QD), spinal cord independence measure (SCIM‐III), transforming growth factor‐β (TGF‐β), tumor necrosis factor alpha (TNF‐α), and upper extremity functional scale (UEFS).

Given their wide diversity and various biological functions, EVs are garnering increasing interest from scientists and are being developed as potential cell‐free therapeutic agents. EVs exhibit low immunogenicity, toxicity, and tumorigenicity, along with good biocompatibility. Their ability to cross specific biological barriers, such as the BBB, further enhances their potential in regenerative medicine. Numerous preclinical studies on EVs have revealed promising possibilities for various therapeutic applications, especially in regenerative medicine and disease treatment. These studies indicate that EVs derived from stem cells and other cell types can effectively promote tissue repair, reduce inflammation, modulate immune responses, enhance cell proliferation, and prevent apoptosis (Figure 5).

**FIGURE 6:**
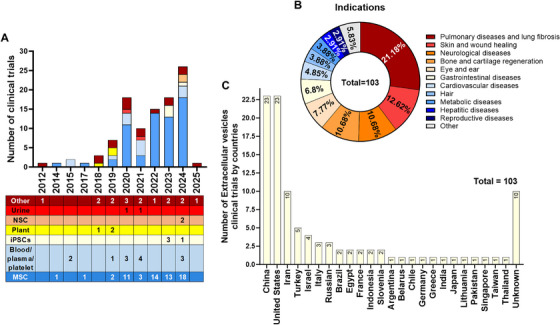
Clinical trials conducted for EV therapy. (A) Since the 2010s, clinical trials for EV therapy have undergone enormous expansion. EVs are derived from various sources, including MSCs, blood‐derived sources, iPSCs, plants, NSCs, and urinary fluid. (B) Studies are classified on the basis of disease category, with pulmonary diseases being the most common. (C) The distribution of EV studies worldwide is the highest in the United States and China. *Abbreviations*: embryonic stem cells (ESCs), extracellular vesicle (EV), mesenchymal stem cells (MSCs), neural stem cells (NSCs), and induced pluripotent stem cells (iPSCs).

More than 100 active clinical trials utilizing EV therapies as the primary intervention, are registered at https://clinicaltrials.gov/ (last update: October 2024, searching for “EVs,” “exosomes,” “MVs”) (Figure 6). Owing to the early stage of EV technology, most of these are phase I/II clinical trials aimed at assessing the safety and initial efficacy of EV therapies across a variety of clinical indications (Table 1).

**TABLE 1:** Clinical trials applying EVs for disease treatment.

No.	Title	Registration number	Phase	Study design	Disease	EVs source	Dose	Route	Outcome
1	Preclinical efficacy and clinical safety of clinical‐grade nebulized allogenic adipose mesenchymal stromal cells‐derived extracellular vesicles [294]	NCT04313647	I		Healthy people	Allogenic AT‐MSC‐EVs	2 × 10^8^ particles to 16 × 10^8^ particles	Aerosol inhalation	‐ Safe and well tolerated without AEs or SAEs
2	Clinical safety and efficacy of allogenic human adipose mesenchymal stromal cells‐derived exosomes in patients with mild to moderate Alzheimer's disease: a phase I/II clinical trial [295]	NCT04388982	I/II	Open label	Alzheimer's disease (mild and moderate)	Allogenic AT‐MSC‐exosome	(2 × 10^8^, 4 × 10^8^, 8 × 10^8^ particles) twice per week, in 12 weeks	Nasal spray administration	‐ Safe and well tolerated without AEs or SAEs ‐ Decrease Alzheimer's Disease Assessment Scale–Cognitive section (ADAS‐cog) scores ‐ Increased the basic version of Montreal Cognitive Assessment score ‐ Improve hippocampal volume shrank in the dose
3	Safety and potential effects of intrathecal injection of allogeneic human umbilical cord mesenchymal stem cell‐derived exosomes in complete subacute spinal cord injury: a first‐in‐human, single‐arm, open‐label, phase I clinical trial [296]	IRCT20200502047277N1	Phase I	Single‐arm	Subacute spinal cord injury	Allogenic UC‐MSC‐exosome	300 µg of total protein of exosome	Intrathecal injection (L4/L5 levels)	‐ Safe and well tolerated without AEs or SAEs ‐ Significant improvements in ASIA pinprick and light touch scores, SCIM III total score, and NBD score after 12 months since injection
4	Exosomes derived from bone marrow mesenchymal stem cells as treatment for severe COVID‐19 [297]	IRB 2020.01 Christ Hospital's institutional review board	I	Nonrandomized open‐label	COVID‐19	Allogeneic BM‐MSC‐Exosome (ExoFlo^TM^)	15 mL	Intravenous injection	‐ Safe and well tolerated without AEs or SAEs ‐ Increase pressure of arterial oxygen to fraction of inspired oxygen ratio (PaO_2_/FiO_2_) ‐ Reduce neutrophil count ‐ Increase lymphopenia including CD3+, CD4+, and CD8+ lymphocyte ‐ Decline acute phase reactants, with mean C‐reactive protein, ferritin, and D‐dimer reduction
5	Nebulized exosomes derived from allogenic adipose tissue mesenchymal stromal cells in patients with severe COVID‐19: a pilot study [298]	NCT04276987	IIa	Single‐arm, open‐labeled, interventional	COVID‐19	Allogeneic AT‐MSC‐Exosome	2 × 10^8^ particles per day (5 days)	Aerosol inhalation	‐ Safe and well tolerated without AEs or SAEs ‐ Improve pulmonary lesions by CT imaging
6	Nebulization therapy with umbilical cord mesenchymal stem cell‐derived exosomes for COVID‐19 pneumonia [299]	Chinese Clinical Trial Registry, ChiCTR2000030261		Pilot	COVID‐19	Allogeneic UC‐MSC‐exosome	7.66e + 0.8 to 7,00e + 0.7 particles/mL	Nebulization	Promote the absorption of pulmonary lesions in mild COVID
7	Bone marrow mesenchymal stem cell‐derived extracellular vesicle infusion for the treatment of respiratory failure from COVID‐19: A randomized, placebo‐controlled dosing clinical trial [300]	NCT04493242	II	Double‐blind, randomized, placebo‐controlled	COVID‐19	Allogeneic BM‐MSC‐exosome (ExoFlo^TM^)	10 mL, 15 mL	Intravenous injection	‐ Safe and well tolerated without AEs or SAEs ‐ Decrease 60‐day mortality ‐ Improved ventilation‐free days (VFDs)
8	Human placental mesenchymal stromal cell‐derived small extracellular vesicles as a treatment for severe COVID‐19: A double‐blind randomized controlled clinical trial [301]	IRCT20130812014333N164.	I	Double‐blind, randomized, controlled	COVID‐19	Placental mesenchymal stromal cell‐derived small extracellular vesicles (PMSC‐EVs)	1.2–5 × 10^8^ particles/kg	Intravenous injection	‐ Safe and well tolerated without AEs or SAEs ‐ Reduce mortality ratio
9	First‐in‐man use of a cardiovascular cell‐derived secretome in heart failure. Case report [302]	NCT05774509	I	Open‐label, single‐center	Nonischemic cardiomyopathies	Cardiovascular progenitor cells‐EVs (SECRET‐HF)	20 × 10^9^ particles/kg 3 times	Intravenous injection	‐ Safe and well tolerated without AEs or SAEs ‐ Decrease NYHA class II ‐ Reduce daily dosing of diuretics ‐ Decrease in the Minnesota living with heart failure (MLHF) questionnaire the score ‐ Echo parameter values: decrease in LVEDV and LVESV and increase in LVEF
10	Pilot safety study of an extracellular vesicle isolate product evaluating the treatment of osteoarthritis in combat‐related injuries [303]	IRCM‐2019‐226		Open‐label, nonrandomized	Osteoarthritis	Allogeneic BM‐MSC‐exosome (ExoFlo^TM^)	NA	Intra‐articular injection	‐ Safe and well tolerated without AEs or SAEs ‐ Improve in Brief Pain Inventory (IPI), Oswestry Disability Index, Lower Extremity Functional Scale, Upper Extremity Functional Scale, and Quick Dash Scale
11	Combination treatment with human adipose tissue stem cell‐derived exosomes and fractional CO_2_ laser for acne scars: A 12‐week prospective, double‐blind, randomized, split‐face study [304]			Double‐blind, randomized, split‐face	Acne scars	Human AT‐MSC‐exosome	9.78 × 10^10^ particles 1.63 × 10^10^ particles	Fractional carbon dioxide laser	Decrease atrophic scar volume, mean pore volume, and skin surface roughness
12	First‐in‐human clinical trial of allogeneic, platelet‐derived extracellular vesicles as a potential therapeutic for delayed wound healing [305]	ACTRN12620000944932	I	Randomized, double‐blind, placebo‐controlled, single dose	Wound healing	Allogenic platelet‐derived EVs	100 µg in 340 µL	Injected subcutaneously adjacent	‐ Safe and well tolerated without AEs or SAEs
13	Efficacy of combined treatment with human adipose tissue stem cell‐derived exosome‐containing solution and microneedling for facial skin aging: A 12‐week prospective, randomized, split‐face study [306]		I	Randomized, split‐face, comparative	Facial skin aging	Allogenic adipose tissue stem cell‐derived exosomes (ASCEs)	2 mL	Microneedling	Reduce skin wrinkle Increase skin hydration and skin elasticity Decrease melanin Increase collagen and elastic fiber deposition in skin tissue

Abbreviation: Alzheimer's disease assessment scale–cognitive section (ADAS‐cog), adverse events (AEs), allogenic adipose tissue stem cell‐derived exosomes (ASCEs), adipose tissue (AT), bone marrow (BM), computed tomography (CT), dental pulp (DP), mesenchymal stem cells (MSCs), Minnesota living with heart failure (MLHF), placental mesenchymal stromal cells (PMSCs), pressure of arterial oxygen to fraction of inspired oxygen (PaO_2_/FiO_2_), serious adverse events (SAEs), umbilical cord (UC), and umbilical cord blood (UCB), and ventilation‐free days (VFDs).

##### 3.2.1.1 Neurological Diseases

Neurological diseases present a significant challenge because of the limited regenerative capacity of the nervous system following injury or illness. This limitation has spurred interest in innovative therapeutic strategies, particularly EV‐based therapies, aimed at restoring function in patients with CNS disorders. Although the precise mechanisms remain under investigation, several promising approaches have emerged to enhance neural regeneration by targeting inflammation modulation, angiogenesis promotion, axon regeneration mediation and/or Schwann cell activation [307].

Inflammation plays a significant role in the pathology of various neurological diseases, including TBI, SCI, and neurodegenerative disorders such as AD and PD. Thus, modulating inflammation has emerged as an effective strategy to enhance the microenvironment of damaged nerves. MSC‐derived EVs (MSC‐EVs) decrease the production of proinflammatory cytokines, including TNF‐α, IL‐1β, and IL‐6, which are known to worsen tissue damage and impede repair [308, 309]. Furthermore, MSC‐EVs can modulate the immune response by influencing immune cells such as microglia and macrophages. By inducing M2 polarization, these EVs facilitate tissue repair and functional recovery. This mechanism has been observed in TBI models, where BM‐MSC‐EVs diminished M1 microglial activation and encouraged the polarization of macrophages toward the M2 phenotype by downregulating the expression of inducible nitric oxide synthase (INOS) and upregulating the expression of clusters of CD206 and arginase‐1 (Arg1) [310]. This inflammatory modulation supports axon regeneration and functional recovery.

Restricted blood flow and oxygen supply to brain tissue after injury can severely damage both neural and vascular structures. Angioneurogenesis is a process involving the stimultanous regeneration of blood flow and promotion of tissue generation. BM‐MSC‐EVs have been shown to increase the population of endothelial cells in the ischemic striatum of stroke mice and in both the lesion boundary zone and the dentate gyrus of TBI mice, thereby inducing vascularization and long‐term neuroprotection [311, 312]. Similarly, UC‐MSC‐EVs activate the Akt/mTOR pathway, which enhances neurovascular regeneration and alleviates blood‒spinal cord barrier disruption [313]. These findings underscore the importance of MSC‐EVs in facilitating the regeneration of neural and vascular tissue in the injured CNS.

MSC‐EVs also contribute to neuroprotection and axonal regeneration by providing neurotrophic factors, protecting against apoptosis and promoting neural plasticity. A variety of neurotrophic factors are released and packaged into MSC‐EVs, including BDNF, GDNF, and VEGF, which promote neuronal survival, differentiation, and regeneration [314]. In conditions such as TBI and stroke, MSC‐EVs have been shown to prevent neural cell death by promoting the expression of antiapoptotic proteins such as BCL‐2 while suppressing the proapoptotic protein Bax [315]. In an SCI model, UC‐MSC‐EVs effectively relieved nerve tissue damage and promoted regeneration in the central nervous system by activating the Akt/mTOR pathway via the miR‐29b‐3p/PTEN axis [316]. In addition, MSC‐EVs can facilitate axon regeneration following SCI by increasing the proliferation of endogenous stem cells through the ERK pathway [317, 318]. Human placental MSC‐derived exosomes markedly increased the proliferation of endogenous neural stem/progenitor cells (NSPCs) with high expression of SOX2+GFAP+, PAX6+Nestin+, and SOX1+KI67+ cells through activation of the MEK/ERK/CREB signaling pathway [318]. However, UC‐MSC‐EVs not only improved the migration of NSPCs but also induced these cells to proliferate and differentiate via the ERK1/2 signaling pathway [317].

Notably, EVs isolated from BM‐MSCs under hypoxic conditions more effectively promoted the proliferation and migration of Schwann cells, supporting their paracrine function through the circRNANkd2/miR‐214‐3p/mediator complex subunit 19 (MED19) axis. Furthermore, hypoxia‐preconditioned BM‐MSC‐EVs demonstrated enhanced functionality in facial nerve repair and regeneration following facial nerve injury [319]. Additionally, miR‐22‐3p in AT‐MSC‐derived exosomes directly inhibited the expression of phosphatase and tensin homolog deleted on chromosome 10 (PTEN), thereby activating the phosphorylation of the AKT/mTOR axis, which promoted Schwann cell proliferation and migration, contributing to the repair of the recurrent laryngeal nerve [320, 321].

The promising use of EV‐based therapies for promoting nerve regeneration and functional recovery has led to the initiation of clinical trials. A single‐arm phase I clinical trial revealed that the intrathecal administration of allogeneic human UC‐MSC‐derived exosomes significantly improved various functional scores, including ASIA pinprick and light touch scores, SCIM III total scores, and NBD scores, in patients with subacute SCI compared with baseline levels [296]. In another clinical trial, patients with mild to moderate AD were treated with intranasal AT‐MSC‐exosomes at three doses of 2, 4, and 8 × 10^8^ particles twice per week (24 treatments in total) [295]. The medium‐dose arm showed significant improvements in cognitive function, as evidenced by a decrease in AD Assessment Scale–Cognitive section (ADAS‐cog) scores and an increase in Montreal Cognitive Assessment scores at week 12. Additionally, improvements in daily living activities were noted, and hippocampal volume shrinkage was reduced. A dose of 4×10^8^ particles is suggested for further large‐scale multicenter clinical trials. Finally, the microRNA (miRNA) and protein expression profiles of AT‐MSC‐derived exosomes revealed 277 miRNAs and 1443 proteins, identifying potential neurotrophic factors for patients with AD in the current clinical trial [295]. These promising results indicate that EV‐based therapies may be potential treatments for neurodegenerative diseases such as SCI and AD.

##### 3.2.1.2 Hepatic Diseases

The liver possesses a unique ability to regenerate and repair itself, making it the only organ in the human body with a remarkable capacity. However, this regenerative ability is limited by the progression of liver disease and liver failure, which also present significant global health challenges. Cell‐free treatments based on EVs offer a highly effective alternative to organ transplantation and cellular therapy for treating liver failure. EVs derived from stem cells, such as UC‐MSCs, AT‐MSCs, and BM‐MSCs, have been most frequently reported to possess therapeutic potential for liver failure and liver injury. Additionally, EVs derived from human menstrual blood‐derived stem cells, iPSC‐derived MSCs, and liver stem cells have also shown protective effects against liver failure [322, 323].

In liver disease, inflammation is a hallmark of various conditions, including acute liver injury, nonalcoholic steatohepatitis, and autoimmune hepatitis. EVs help mitigate liver inflammation through different mechanisms. In acute liver failure, UC‐MSC‐EVs and AT‐MSC‐EVs containing miR‐17 or lncRNA H19 reduced the expression of NLPR3, caspase‐1, IL‐1β, and IL‐6, which are key factors related to hepatic injury [324, 325]. In addition, UC‐MSC‐EVs suppressed CCL4‐induced liver injury by activating the ERK and IGF‐1R/PI3K/AKT signaling pathways [326]. Human UC‐MSC‐derived exosomes have also been demonstrated to mitigate hepatic ischemia‒reperfusion (I/R) injury through the suppression of oxidative stress and the neutrophil‐mediated inflammatory response. This is achieved by inhibiting Beclin1‐ and FAS‐mediated autophagy and apoptosis while also modulating the inflammatory immune response [327]. Furthermore, UCB‐MSC‐derived exosomes presented anti‐apoptotic, prosurvival, and anti‐inflammatory effects through the modulation of the GSK3β‐mediated Wnt/β‐catenin signaling pathway or by influencing the balance between Treg and Th17 cells [328].

Fibrosis, characterized by excessive accumulation of ECM, is a key feature of chronic liver diseases, including cirrhosis. MSC‐EVs inhibit hepatic stellate cell activation and proliferation through the delivery of antifibrotic miRNAs such as miR‐122 and miR‐199 and suppress key fibrogenic signaling pathways such as the TGF‐β/Smad and Wnt/β‐catenin pathways [329]. These vesicles also enhance liver regeneration by delivering HGFs and miRNAs that promote hepatocyte proliferation, counteracting fibrotic damage. Moreover, MSC‐EVs also downregulate the expression of ECM proteins, such as collagen I and fibronectin, while promoting the expression of MMPs [330].

Menstrual blood stem cell‐derived exosomes have been shown to inhibit liver cell apoptosis and reduce the number of liver MNCs and the level of the active apoptotic protein caspase‐3 in injured livers, resulting in significantly improved liver function [322]. AT‐MSC‐derived exosomes attenuated liver I/R injury through the PGE2‐mediated ERK1/2 and GSK‑3β signaling pathways [331]. In addition, miR‐17 or lncRNA H19 in AT‐MSC‐derived exosomes promoted hepatocyte proliferation through the HGF/c‐Met pathway and related downstream channels, thereby increasing the survival rate of rats with ALF [325]. In a rat partial‐ hepatectomy model, miR‐124 in UC‐MSC‐derived exosomes promoted liver regeneration via the inhibition of Foxg1 [332]. Interestingly, both EVs isolated from normal and damaged tissue significantly attenuated the progression of apoptosis induced by CCL4 and induced the proliferation of hepatocytes by increasing the level of HGF at the site of injury [333].

##### 3.2.1.3 Pulmonary Diseases and Lung Fibrosis

Recent research has elucidated the critical role of EVs in the treatment of various pulmonary diseases, including acute lung injury (ALI), ARDS, BPD, and idiopathic pulmonary fibrosis. EVs act as natural carriers of bioactive molecules, modulating cellular functions and promoting tissue repair. The mechanisms of action of these compounds in pulmonary disease and fibrosis are multifaceted and include inflammation, fibrosis, apoptosis, and tissue regeneration.

MSC‐derived EVs are particularly effective in mitigating inflammation by modulating cytokine signaling pathways. These EVs inhibit the activation of key inflammatory pathways, such as the NF‐κB and TGF‐β pathways, leading to reduced expression of proinflammatory cytokines such as IL‐6 and TNF‐α, thereby alleviating inflammation in lung tissues [334]. BM‐MSC‐EVs decrease the infiltration of inflammatory cells, both total white blood cells and neutrophils, and MIP‐2 cytokine levels in ALI mice [334]. Similarly, AT‐MSC‐EVs containing miR‐27a‐3p are effectively taken up by alveolar macrophages, which facilitates polarization toward the M2 macrophage phenotype, a crucial process in the repair of acute lung injury. Specific miRNAs within EVs, such as miR‐21‐5p, miR‐182‐5p, miR‐23a‐3p, miR‐Let‐7, and miR‐27a‐3p, coordinate the response to pulmonary fibrosis by downregulating proinflammatory genes, enhancing phagocytosis and promoting the resolution of inflammation [335]. Additionally, these miRNAs decrease the synthesis of proinflammatory cytokines and inhibit signaling pathways associated with the activation of inflammation, including the NLRP3 inflammasome and NF‐κB. Furthermore, AT‐MSC‐EVs containing miR‐127‐3p and miR‐125b‐5p further contribute to M2 macrophage polarization, highlighting their role in mitigating inflammation and supporting tissue repair in pulmonary conditions.

EVs have also proven effective in protecting and regenerating pulmonary epithelial cells, which are crucial for maintaining alveolar integrity and overall lung function. MSC‐EVs promote epithelial proliferation, prevent apoptosis, and help restore epithelial integrity. MSC‐EVs have been shown to protect pulmonary epithelial cells from the harm caused by ALI/ADRS [336]. BM‐MSC‐EVs enhance barrier function and inhibit epithelial apoptosis by upregulating GPRC5A, which in turn increases the expression of junction proteins such as E‐cadherin, claudin‐1, occluding, and ZO‐1. Moreover, these EVs deliver miR‐425, activating the PI3K/Akt pathway to alleviate hyperoxia‐induced ALI. The transfer of lncRNA‐p21 via EVs also prevents epithelial apoptosis and ALI by modulating the expression of miR‐181 and SIRT1. MSC‐EVs that overexpress miR‐30b‐3p enhance antiapoptotic and proliferative effects, effectively countering LPS‐induced alveolar epithelial cell apoptosis. These effects are vital for preventing further lung damage during the inflammatory response.

In the context of pulmonary fibrosis, MSC‐EVs play a crucial role by delivering antifibrotic miRNAs, such as miR‐29 and miR‐21, which target profibrotic genes in myofibroblasts and effectively suppress excessive ECM production [337]. MSC‐EVs also inhibit fibrogenic signaling pathways, such as the TGF‐β/Smad and Wnt/β‐catenin pathways, which are critical for the development of lung fibrosis [334]. Specifically, miR‐29b‐3p and miR‐186 have the potential to inhibit the activation of fibroblasts by regulating FZD6, SOX4, and DKK1 [335]. Moreover, EVs suppress the synthesis of collagen and fibronectin, critical components of the ECM [338]. BM‐MSC‐EVs reduced the expression of profibrotic factors (TGF‐β1, α‐SMA, and collagen I/III) through the modulation of miR‐23a‐3p and miR‐182‐5p while also inhibiting the NF‐κB/Hedgehog pathways. Furthermore, UC‐MSC‐EVs and EVs derived from bronchial epithelial cells can directly disrupt the TGF‐β pathway, thereby inhibiting myofibroblast proliferation and collagen production [338].

The COVID‐19 pandemic caused by SARS‐CoV‐2 highlighted, the significant challenges in managing severe respiratory complications, particularly ARDS. MSC‐EVs have emerged as a promising therapeutic approach to mitigate the cytokine storm, restore suppressed antiviral defenses, and repair lung damage associated with mitochondrial dysfunction. To date, clinical trials evaluating MSC‐EVs are limited and focus mainly on patients with COVID‐19. In a phase I trial involving 24 severe cases of ARDS associated with COVID‐19 [297, 300], a single 15 mL intravenous dose of allogenic BM‐MSC‐EVs (ExoFlo) improved clinical status and oxygenation, as evidenced by an increased PaO_2_/FiO_2_ ratio, a reduced neutrophil count, and improved lymphopenia [297]. In a subsequent phase II trial, 102 patients with COVID‐19‐associated moderate to severe ARDS were enrolled at five sites across the United States and received two doses of either 10 or 15 mL of ExoFlo [300]. The results indicated that 60‐day mortality was lower in the ExoFlo‐15 group than in the placebo group and that the number of ventilation‐free days improved [300]. A similar reduction in mortality was observed in patients treated with human placental MSC‐derived small EVs, although this treatment did not significantly impact laboratory values [301]. Additionally, nebulized EVs have been shown to be safe and effective for pulmonary diseases [294, 298, 299]. Improved pulmonary lesions were observed via CT imaging in seven patients with severe COVID‐19 who received aerosol inhalation of AT‐MSC‐exosomes at a dose of 2 × 10^8^ particles/day for 5 consecutive days [298]. Nebulized UC‐MSC‐EVs also promoted the absorption of pulmonary lesions and reduced the duration of hospitalization for mild cases of COVID‐19 pneumonia [299]. In summary, MSC‐EVs demonstrate a favorable safety profile, the capacity to restore oxygenation, the ability to downregulate the cytokine storm, and the potential to reconstitute immunity.

##### 3.2.1.4 Cardiovascular Diseases

The limited regenerative capacity of cardiomyocytes poses a significant challenge for cardiovascular therapy, particularly in conditions involving extensive damage, such as myocardial infarction (MI) and I/R injury. Emerging EV‐based therapies offer promising avenues for cardiac regeneration, leveraging the bioactive cargo of EVs to modulate inflammation, prevent apoptosis, promote cell proliferation, and stimulate tissue repair [339].

Many preclinical studies have shown that MSC‐EVs exhibit potent immunomodulatory effects by altering inflammatory pathways and macrophage polarization. BM‐MSC‐EVs have immunomodulatory effects on macrophages and inhibit TLR4 through the shuttling of miR‐182, leading to attenuation of myocardial I/R in a mouse model [340]. Similarly, exosomes released from BM‐MSCs containing miR‐182‐5p not only diminish inflammation or necrosis but also ameliorate cardiac function and reduce MI via Gasdermin D [341]. In another study, BM‐MSC‐EVs induced the polarization of macrophages to the M2 phenotype via miR‐21‐5p, which resulted in a decrease in inflammation and promoted heart repair [342]. UC‐MSC‐EVs enriched with miR‐100‐5p suppressed FOXO3 to inhibit NLRP3 inflammasome activation and reduce cytokine release, protecting cardiomyocytes from hypoxia/reoxygenation‐induced pyrosis and injury [343].

In addition, other studies have indicated that MSC‐EVs can mitigate myocardial damage by preventing apoptosis and promoting CM proliferation [344, 345]. BM‐MSC‐EVs presented with miR‐486‐5p regulate the PTEN/PI3K/AKT signaling pathway to inhibit the apoptosis of injured cardiomyocytes and repair the myocardial injury caused by I/R [344]. Other studies have shown that the presence of the lncRNA HCP5 in BM‐MSC‐EVs protects myocardial cells against I/R injury by activating the IGF‐1/PI3K/AKT pathway by sponging miR‐497 [345]. miR‐143‐3p in BM‐MSC‐EVs effectively reduces apoptosis through regulating autophagy via the checkpoint kinase 2 (CHEK2/CHK2)/beclin 2 (BECN2) pathway [346].

AT‐MSC‐EVs demonstrate robust cardioprotective properties that safeguard the myocardium from I/R injury by activating the Wnt/β‐catenin signaling pathway [347], effectively prevent heart damage by targeting Bcl2 binding component 3 (BBC3/PUMA) and the ETS proto‐oncogene 1 transcription factor (ETS1) through the miR‐221/miR‐222 pathway [348], and they also protect cardiomyocytes from oxidative stress‐induced apoptosis and offer cardioprotective benefits [349].

PSCs, such as ESCs and iPSCs, possess enormous potential to differentiate into various cardiac cell types, making them promising candidates for cardiac regeneration. ESC‐derived EVs promote endogenous natural repair mechanisms and improve cardiac function following MI [350]. iPSC‐derived EVs have protective effects on heart cells in vitro, promote angiogenesis, and increase apoptosis in vivo. Injection of iPSC‐derived EVs into the mouse ischemic myocardium prevents I/R injury and provides cardioprotective effects [351].

The clinical application of EV‐based therapies has shown promising results in CVD treatment. A 59‐year‐old man with nonischemic cardiomyopathy received three intravenous infusions of the EV‐enriched secretome from cardiovascular progenitor cells derived from iPSCs (SECRET‐HF) [302]. The patient was classified as New York Heart Association (NYHA) class III and had previously been implanted with a prophylactic internal cardioverter defibrillator. Six months posttreatment with SECRET‐HF, his condition improved to NYHA class II. The patient reduced his daily diuretic dosage and had better scores on the Minnesota living with heart failure (MLHF) questionnaire as well as improved echo parameters.

##### 3.2.1.5 Musculoskeletal Disease

Bone and cartilage are composed of connective tissue and are fundamental structures that play a role in controlling body movement, providing mechanical support and mineral storage and protecting internal organs. The repair process following bone damage is highly complex and involves interactions among various cell types and biological signaling pathways. Bone regeneration includes a series of coordinated biological processes referred to as osteoconduction and osteoinduction. Compared with the repair of bone‐damaged tissue, the treatment of cartilage damage presents greater challenges and complexities, primarily due to the inherent characteristics of cartilage, which does not regenerate readily. EVs represent a promising new strategy for bone and cartilage reconstruction therapy. They have significant potential in promoting repair and regeneration by regulating the microenvironment at injury sites, stimulating bone and playing proangiogenic roles in various cell types essential for bone formation.

AT‐MSC‐EVs inhibit the secretion of IL‐1β and IL‐18 and suppress the NLRP3 inflammasome in osteoclasts, leading to reduced bone resorption and recovery from bone loss in streptozotocin‐induced diabetic osteoporosis rats [352]. To protect cartilage and bone from OA, BM‐MSC‐EVs safeguard chondrocytes from apoptosis and inhibit macrophage activation. Additionally, BM‐MSC‐EVs restore the expression of chondrocyte markers, including type II collagen and aggrecan, while suppressing the expression of catabolic markers (MMP‐13, ADAMTS5) and inflammatory markers (iNOS) [353]. Zheng et al. [354] discovered that exosomes derived from normal primary chondrocytes can restore mitochondrial function and promote the polarization of macrophages to the M2 phenotype. Additionally, intra‐articular injection of these exosomes effectively inhibited the onset and progression of OA [354].

In vitro data indicate that BM‐MSC‐derived exosomes enhance obsteoblast activity, promoting osteogenic differentiation, ultimately facilitating fracture healing via miR‐22‐5p/Anxa8 axis [355]. In a mouse model of osteoporosis, BM‐MSC‐EVs that contain MALAT1 enhance osteoblast activity by regulating the miR‐34c/SATB2 axis, thereby alleviating osteoporosis [356]. Similarly, exosomes from vascular endothelial cells can reverse glucocorticoid‐induced inhibition of osteoblasts by suppressing ferritinophagy‐dependent ferroptosis [357]. Hu et al. [358] discovered that BM‐MSC‐EVs enriched with miR‐335 enhance osteoblast differentiation and accelerate fracture healing by targeting VapB and activating the Wnt/β‐catenin pathway.

In 2020, a pilot safety study was approved by the USA Institutional Review Board to evaluate the safety of allogenic BM‐MSC‐EVs (ExoFlo) [303]. The study included a substantial number of Navy SEAL veterans, specifically thirty‐three individuals diagnosed with combat‐related injuries resulting in moderate to severe OA. These veterans received single 2 mL ExoFlo treatment for various sites of OA: knee (*n* = 58), shoulder (*n* = 32), elbow (*n* = 16), hip (*n* = 12), ankle (*n* = 8), and wrist (*n* = 6). After 6 months of follow‐up, no complications were reported, and patients demonstrated improvements in the Brief Pain Inventory, Oswestry Disability Index, Lower Extremity Functional Scale, Upper Extremity Functional Scale, and Quick Dash Scale scores.

##### 3.2.1.6 Skin and Wound Healing

Numerous investigations have indicated that MSC‐EVs, which can overcome the inherent limitations of MSCs [359], are advantageous for all phases of the skin repair process, such as hemostasis, inflammation, proliferation, and remodeling [360]. The potential functions of EVs in cosmetic applications and the treatment of various skin conditions, including antiaging, wound healing, and anti‐inflammatory effects, have been increasingly revealed. Beneficial EVs can be derived from multiple sources, such as plants, probiotics, and human MSCs [361]. Among them, MSC‐EVs have been studied the most for cutaneous applications [361]. Some studies demonstrate a role in antiwrinkle and antiaging [362, 363] of MSC‐EVs, which is meaningful for the development of novel cosmetic products. The general mechanism by which MSC‐EVs recover the functions of senescent dermal fibroblasts [364] and keratinocytes [365] is mediated by downregulation of oxidative stress and senescence‐associated molecules, which leads to increased expression of ECM components, including collagen and elastin [366, 367]. In addition, EVs derived from neonatal human dermal fibroblasts (HDFs) have been utilized to encapsulate and deliver *COL1A1* mRNA to the dermis via microneedles to reduce wrinkles in a photodamaged murine skin model [18]. The results demonstrated that COL1A1 mRNA‐loaded HDF‐EVs could dramatically induce dermal collagen grafts, leading to impressive elimination of wrinkles. Another representative application of MSC‐EVs is wound healing [360, 368].

The healing functions of MSC‐EVs may be mediated mainly by their miRNAs [369]. For example, miR‐233 in BM‐MSC‐EVs can regulate the M1‐like inflammatory phenotype to the M2‐like regenerative phenotype of macrophages [370], which is beneficial for the phase transition from inflammation to proliferation. In addition, miR‐125a‐3p in ADSC‐EVs supports angiogenesis [371], whereas miR‐126 in BM‐MSC‐EVs increases new capillary formation [372], miR‐192‐5p in AT‐MSC‐EVs can eliminate fibrosis during remodeling [373]. Notably, MSC‐EVs have also been shown to exert therapeutic effects on severe cutaneous inflammatory diseases, such as atopic dermatitis and psoriasis [374]. For example, IFN‐γ‐primed MSC‐EVs can reduce both inflammation and the expression of IL receptors for Th2 cytokines while normalizing the skin barrier in an AD murine model [375]. Moreover, IFN‐γ‐primed UC‐MSC‐EVs exhaust Th17 cells and decrease the concentration of inflammatory cytokines in psoriasis murine skin [376].

The clinical use of EVs derived from AT‐MSCs and platelets has been demonstrated to be safe for skin applications [304, 305, 306]. After 12 weeks of treatment with AT‐MSC‐EVs, patients experienced a reduction in skin wrinkles, along with increased skin elasticity and hydration and a decrease in melanin levels [306]. Additionally, histopathological analysis of facial skin tissues revealed greater deposition of collagen and elastic fibers than on the control side.

#### 3.2.2 EVs as a Promising Platform for Therapeutic Delivery

EVs have also emerged as promising delivery systems for a wide range of therapeutics. Their natural ability to carry diverse biomolecules and facilitate intercellular transfer makes them ideal for delivering chemotherapeutic drugs, proteins/peptides, and RNA‐based therapeutics such as miRNAs, siRNAs, or mRNAs. Compared with synthetic carriers such as nanoparticles and liposomes, EVs offer superior biocompatibility, reduced immunogenicity, and the ability to cross biological barriers, such as the BBB, owing to their natural origin.

##### 3.2.2.1 EVs as Therapeutic Carriers

###### 3.2.2.1.1 Delivery of Exogenous Cargos

Delivery of exogenous cargos involves the packing of drugs or therapeutic agents into EVs after their isolation through a passive or active loading process. In the passive loading method, therapeutic cargos are loaded into EVs by incubating the EVs with the desired cargo, allowing the cargo to diffuse into the EVs. For example, dopamine can be effectively incorporated into blood exosomes via this method. These dopamine‐loaded exosomes have been shown to successfully deliver dopamine to the brain, improving therapeutic effects in a PD mouse model [377]. Active loading methods, such as electroporation, sonication, and freeze‒thaw cycles, create temporary pores in the EV membrane, causing transient membrane permeability and facilitating cargo entry. Catalase, a potent antioxidant, was loaded into exosomes isolated from Raw 264.7 macrophages via incubation, freeze‒thaw cycles, or sonication. These catalase‐loaded exosomes exhibited significant neuroprotective effects on a mouse model of **PD** [378].

While the loading of exogenous cargos is simple, it faces several challenges. First, passive loading can result in low loading efficiency, especially for negatively charged and hydrophilic molecules such as RNA. Second, EV membrane integrity can be compromised under the physical effects of electroporation, sonication, and freeze‒thaw cycles, leading to vesicle aggregation and reduced stability of the final loaded EVs. Third, large‐scale production for clinical applications presents another hurdle, as ensuring consistency and quality across different batches of loaded EVs is crucial.

###### 3.2.2.1.2 Delivery of Endogenous Cargos

This method involves loading therapeutic molecules into EVs during their biogenesis. To achieve this goal, EV‐producing cells are transfected with specific plasmids so that the cells overexpress the desired molecules, which are then naturally packaged into EVs. For example, BM‐MSCs were transfected with the lentiviral vector Lv‐miR‐125b to upregulate miR‐125b. The resulting exosomes (BM‐MSCs‐Exo‐miR125b) significantly increased cell viability, attenuated apoptosis, and reduced proinflammatory cytokine levels, offering protection against myocardial I/R injury in rats [379]. In another study, human BM‐MSCs were transfected with a lentiviral vector to overexpress TSG‐6. Exosomes containing TSG‐6 from these cells exhibited anti‐inflammatory effects, protecting against scar formation in a mouse skin wound model [380]. Additionally, Tao et al. [381] introduced miR‐140‐5p into human synovial MSC‐EVs by transfecting the cells with miR‐140‐5p lentivectors. The isolated EVs reduced ECM production, effectively inhibiting OA in a rat model [381]. Endogenous loading offers several advantages for EV‐based therapeutics. The incorporation of cargos into EVs during their biogenesis in a biologically relevant way ensures a natural packing arrangement, preserving the natural structure and function of the vesicles. This method has also demonstrated increased efficiency in packaging nucleic acids or proteins into EVs [382]. However, precise control over the natural loading process is still challenging because of limited knowledge about the cargo‐loading mechanism of ongoing EV research. Moreover, constructing genetically modified cell lines for endogenous loading can be time‐consuming.

Generally, exogenous loading is preferred when precise control over a specific cargo and its concentration within EVs is necessary. In contrast, endogenous loading is favored when greater stability and more efficient cargo incorporation into EVs are needed. The selection between these methods often depends on the type of therapeutic cargo, the target cells, and the intended clinical application.

##### 3.2.2.2 Modified EVs for Targeted Delivery of Therapeutics

While efficient therapeutic loading is a critical determinant of therapeutic efficacy, ensuring the selective delivery of therapeutic‐loaded EVs to target tissues is equally important for minimizing off‐target effects and toxicity. Strategies for EV engineering or modification have gained attention as promising approaches to enhance tissue targeting. To date, genetic modification, lipid insertion, and chemical ligation are among the most common methods explored in regenerative medicine to optimize EV‐targeting capabilities [383].

###### 3.2.2.2.1 Genetic Modification

EV‐producing cells can be genetically engineered to express specific ligands or specific molecules on the surface of their vesicles. This method ensures that naturally produced EVs are inherently equipped for targeted delivery. Membrane proteins such as lysosome‐associated membrane protein 2 (Lamp2b) and tetraspanins (CD63, CD81, and CD9), which are commonly found on EVs, can serve as anchoring proteins for targeting moieties. These exosomal proteins facilitate the display of targeting molecules on the EV surface. Lamp2b is currently the most widely used protein for targeted application in regenerative medicine, whereas tetraspanins are commonly employed for cancer therapy. Lamp2b was first used by Alvarez‐Erviti et al. [384] as an anchoring protein for RVG (rabies virus glycoprotein), a neuron‐specific peptide. When expressed on the surface of exosomes derived from murine dendritic cells, Lamp2b‐RVG specifically targeted exosomes to the brain without inducing nonspecific uptake in other tissues of wild‐type mice [384]. Subsequently, the Lamp2b‐RVG system has been widely adopted for delivering diverse therapeutics to specific tissues or organs in various diseases. For example, Lamp2b‐RVG EVs loaded with exogenous BACE1 siRNA have shown promising efficacy in an AD mouse model [384]. Additionally, Lamp2b‐RVG EVs have been used to deliver HMGB1‐siRNA [385], miR‐124 [386], NGF mRNA [387], and circSCMH1 (circular RNA SCMH1) [388] for the treatment of ischemic stroke. Furthermore, Lamp2b‐RVG EVs were loaded with catalase mRNA [389], and the DNA aptamers F5R1 or F5R2 [390] were investigated for PD treatment in mice. Lamp2b has also been employed to display other targeting moieties on the surface of EVs, such as the chondrocyte‐affinity peptide (CAP) peptide [391] and the E7 peptide [392], for targeted delivery of miR‐140 and kartogenin, respectively, for OA treatment. Additionally, Lamp2b was fused with the HSTP1 (heat shock transcription factor 1) peptide to target activated hepatic stellate cells for the treatment of liver fibrosis [393].

###### 3.2.2.2.2 Lipid Insertion

The approach involves incorporating lipid segments into targeting moieties to promote their interaction with biological membranes. These lipid‐tethered targeting molecules can then be spontaneously inserted into EV membranes through simple mixing and incubation processes [394]. For example, in the study by Cui et al. [395], the RVG peptide was conjugated to DOPE‐NHS (dioleoylphosphatidylethanolamine N‐hydroxysuccinimide) to obtain DOPE‐RVG. The DOPE‐RVG complex was then attached to exosomes isolated from BMSCs through lipid insertion with the DOPE‐NHS linker. The intravenous administration of MSC‐RVG‐exos significantly improved learning and memory capabilities, reduced plaque deposition and Aβ levels, and normalized the levels of inflammatory cytokines in AD model mice [395]. Like DOPE, DSPE (1,2‐distearoyl‐sn‐glycero‐3‐phosphoethanolamine) is also commonly utilized in drug delivery systems [396]. You et al. [397] demonstrated that PEG‐Vitamin A, conjugated with DSPE to form DSPE‐PEG‐vitamin A, could be incorporated into EVs isolated from AT‐MSCs by hydrophobic insertion. Upon intravenous injection into a liver fibrosis model, these vitamin A‐coupled EVs are selectively taken up by activated hepatic stellate cells leading to a reduction in hepatic fibrogenesis. This approach has significant potential for the treatment of liver fibrosis [397].

###### 3.2.2.2.3 Chemical Ligation

This method involves the formation of covalent bonds between reactive groups on EV membrane lipids or proteins, such as ─NH2 (amino), ─COOH (carboxy), and ─SH (thiol) groups, and reactive fragment‐tagged peptides [383]. Compared with lipid insertion, covalent bonding provides greater long‐term stability for the binding of targeting ligands to EVs. For example, a c(RGDyK)‐targeting peptide was conjugated to MSC‐derived exosomes via chemical ligation to target the lesion region in the ischemic brain [398, 399]. These modified exosomes were loaded with curcumin [398] or miR21[399] for the treatment of ischemic stroke with promising outcomes.

##### 3.2.2.3 EVs as Gene Therapy Vehicles

EVs could serve as platforms for delivering gene‐editing tools, such as CRISPR–Cas9. This technology could be used to correct genetic defects, increase cellular regeneration, and/or facilitate tissue repair in conditions such as inherited diseases or injuries [400]. The CRISPR/Cas9 system consists of a Cas9 nuclease and a single guide RNA (sgRNA), which direct Cas9 to specific DNA sequences for editing. This system can be delivered to target cells in the form of DNA (plasmid), mRNA, or ribonucleoprotein (RNP) complexes [401]. While the use of EVs to deliver CRISPR/Cas9 has been extensively studied for cancer therapy [402], EVs also hold significant potential for applications in regenerative medicine. For example, EVs derived from HEK293T (human embryonic kidney) cells were transfected with Cas9 RNP. These modified EVs were shown to target specific sites on the HGF gene, resulting in the upregulation of endogenous HGF expression in an acute liver injury‐induced mouse model [403]. Similarly, Cas9 RNPs were loaded into purified exosomes isolated from hepatic stellate cells (LX‐2 cells) through electroporation. These exosomes targeted PUMA (p53 upregulated modulator of apoptosis) and Cyclin E1 (CcnE1), which are important therapeutic targets for acute liver injury and chronic liver fibrosis, respectively [404]. Together, these findings highlight the potential of Cas9 RNP EVs as a promising therapeutic strategy for the treatment of liver diseases.

In another study, Cas9 RNPs were loaded into isolated serum EVs via a transfection kit. These modified EVs caused exon deletion in the dystrophin gene, allowing dystrophin expression in muscle fibers in a DMD mouse model. This approach opens new opportunities for the rapid and safe delivery of CRISPR components to treat DMD [405]. Furthermore, exosomes from human UC‐MSCs were modified with a CAQK peptide to target brain injuries. Cas9 DNA was then loaded into these exosomes via electroporation. When administered intravenously to SCI model mice, the exosomes reached the injury site and inhibited the inflammatory response in the spinal cord, promoting recovery after SCI [406]. A recent study successfully produced Cas9 RNP‐loaded exosomes, termed NanoMEDIC, from HEK293T cells. These exosomes exhibited exon skipping with an efficiency exceeding 90% in skeletal muscle cells derived from the iPSCs of DMD patients. These results highlight the potential of NanoMEDIC for in vivo genome editing therapy to target DMD [407]. In research conducted by Osteikoetxea et al. [408], Cas9 and sgRNA‐containing plasmids were transfected into Expi293F cells to produce Cas9 RNP‐loaded EVs. These engineered EVs effectively knocked down the PCSK9 gene in vitro. Low PCSK9 gene expression reduces circulating low‐density lipoproteincholesterol, indicating the therapeutic potential of CRISPR/Cas9‐loaded EVs for treating atherosclerotic CVD.

#### 3.2.3 EVs as Therapeutic Targets

In addition to their therapeutic benefit, subsets of EVs may play important roles in various pathological processes and are directly implicated in the development and progression of many diseases [409]. For example, EVs produced by microglia, astrocytes, and endothelial cells in the brain carry misfolded proteins such as Aβ, tau, α‐synuclein, and specific miRNAs. These EVs mediate the transmission of pathogenic components in the brain, potentially accelerating neurodegeneration in conditions such as AD and PD. Certain neurotoxic EVs can also cross the BBB, further propagating neural dysfunction [410]. Similarly, EVs released during CVD can carry inflammatory and prothrombotic factors, which contribute to disease progression [410]. Therefore, therapeutically targeting these pathological EVs helps prevent the spread of pathogenic proteins, slowing disease progression. Currently, the inhibition of EV biogenesis and release or blockade of EV uptake are under investigated strategies for targeting pathogenic EVs.

##### 3.2.3.1 Inhibiting EV Biogenesis and Release

The list of inhibitors of EV biogenesis and release has been intensively reviewed by Catalano and O'Driscoll [411]. Among the listed inhibitors, GW4869 has been studied relatively extensively for cancer and regenerative therapy. GW4869 is a chemical compound commonly used as an inhibitor of neutral sphingomyelinase (nSMase), an enzyme involved in the formation and release of exosomes [412]. Inhibiting nSMase with GW4869 reduces exosome release from cells. Dinkins et al. [413] reported that GW4869 treatment reduced Aβ plaques in the brains of an AD mouse model, indicating the potential of GW4869 in targeting EV release for the treatment of AD. Similarly, inhibiting exosome release with GW4869 reduces sepsis‐induced cardiac inflammation, attenuates myocardial dysfunction, and prolongs survival in an LPS‐induced sepsis mouse model [414]. In another study, treatment with GW4869 inhibited exosome release by fibroblasts (CFs), preventing myocardial hypertrophy and cardiac fibrosis in a mouse model of cardiac hypertrophy [415].

##### 3.2.3.2 Blocking EV Uptake

Blocking EV uptake can prevent harmful signals or materials from entering healthy cells. This can be achieved by targeting EV‐mediated uptake receptors on the EV surface via specific siRNAs or antibodies. For example, the uptake of EVs from senescent BMSCs by muscle satellite cells contributes to the development of sarcopenia. Therefore, inhibiting EV uptake through blockade of CD81 with CD81‐specific siRNA or an anti‐CD81 antibody attenuated sarcopenia in aged mice [416].

The major challenges in using EVs as therapeutic targets are related to specificity and safety concerns. As EVs are involved in both physiological and pathological processes, therapy must selectively target disease‐related EVs without disrupting the normal function of healthy EVs or causing unintended side effects in healthy tissues. While both in vitro and in vivo studies have shown promise for EV‐targeting therapy in disease models, this approach remains far from clinical application. A more comprehensive understanding of EV biogenesis and uptake is needed before this strategy can be translated into an effective treatment.

### 3.3 Advantages and Challenges of EV‐Based Therapy

Unlike cells that accumulate in the lungs after intravenous injection [417, 418], EVs bypass pulmonary capture, circulate freely in the bloodstream, and efficiently cross vascular barriers into tissues because of their smaller size [419]. Research by Kim et al. [420] revealed that when bare MSC‐EVs are injected intravenously into mice, they tend to accumulate in organs such as the lung, spleen, kidney, and especially the liver. Interestingly, a recent meta‐analysis of clinical trials indicated that EV‐based therapies were safe and had a low incidence of critical side effects [421]. Moreover, there is no risk of tumor formulation in EV‐based therapies because EVs lack functional nuclei, are incapable of replication [287]. In addition, EVs can penetrate through specific biological barriers, such as the BBB and accumulate at target sites [422, 423]. Banks et al. [423] demonstrated that different kinds of EVs could effectively cross the BBB to localize in the brain via diverse mechanisms of uptake involving adsorptive transcytosis and specific transporters. As a result, EVs have even been proven to be effective drug delivery systems for a wide range of therapeutic agents, including chemicals, proteins and genetic materials [424]. While cell‐based products face significant limitations in terms of viability and storage stability [425], EV‐based products offer greater versatility with formulations such as frozen liquids, hydrogels, and freeze‐dried powders, ensuring improved stability and convenience for diverse applications. For example, EV‐containing buffer solution is used for intravenous injection to treat liver fibrosis [426, 427], a hydrogel is used for topical application to treat diabetic wounds [428, 429], and EV‐containing freeze‐dried powder is used for long‐term storage and inhalation to treat pulmonary fibrosis [430].

EV therapy has several limitations, including the lack of consistent EV isolation methods and QC [431]. Current isolation technologies have shown low efficacy in ultracentrifugation, poor homogeneity in tangential flow filtration, and/or difficulty scaling up in immunocapture capture methods. In addition, batch‐to‐batch variance affected by the cell source and culture process, lack of suitable QC parameters, and high cost of manufacturing and storage compared with nonbiological drugs are other major challenges for clinical translation, which leads to the absence of established regulations and standards [432]. The ability of GMP facilities to produce EVs at a large scale also remains challenging [433]. Furthermore, natural liver accumulation and capture by circulating immune cells of EVs upon injection hinder their delivery to other targeted organs [434], which needs to be overcome. In addition, because of the lack of clinical trials related to EV‐based products, confirming the real efficacy and long‐term safety of therapeutic EVs remains the greatest challenge.

### 3.4 Strategies to Overcome Challenges

The long‐term storage stability and targetability of EVs must be improved before moving through the next steps, such as manufacturing and clinical translation. Therefore, it is necessary to develop appropriate formulations for EVs. Lyophilized powder can be considered the most promising formulation for commercial EV‐based products because it is easy to store and deliver. However, the cryoprotectant used in the freeze‒drying process needs to be chosen carefully to avoid any negative effects on either the cellular uptake ability or the biological functions of EVs. Recently, carbohydrate polymers such as hyaluronic acid and chondroitin sulfate have emerged as biomaterials for increasing the storage stability of MSC‐EVs under mild conditions and enhancing their functions by improving their uptake into target cells and tissues [435, 436]. In addition to stability, optimizing administration routes and pharmacokinetic properties is required for evaluating safety and maximizing therapeutic efficacy of EVs. Comprehensive biodistribution and clearance studies in preclinical models are necessary to determine their bioavailability, circulation time, and tissue‐specific accumulation. These studies would supply useful data for selecting the most effective delivery methods and dosage regimens tailored to specific disease conditions.

Regulatory approval remains a major challenge due to the complexity and heterogeneity of EVs. Recently, there are public safety notifications on EV‐related products issued by the US FDA [437]. Large‐scale GMP‐compliant EV manufacturing requires closed‐system processes to minimize contamination and improve scalability. Lessons from the manufacturing of lipid nanoparticles (LNPs) and viral vectors (AAVs) can inform the development of optimized EV production pipelines [438]. Furthermore, implementing advanced EV characterization technologies is essential for accurate QC, ensuring batch consistency and regulatory compliance.

Another critical challenge is defining the mechanism of action (MoA) and standardizing EV dosage for clinical applications. Understanding the MoA is essential to predict therapeutic efficacy, minimize off‐target effects, and establish scientific rationales for potential drug combination strategies. Unlike conventional pharmaceuticals, EVs exert their effects through a combination of bioactive proteins, lipids, and RNAs. Therefore, determining dosage based on EV particle count rather than total protein concentration is recommended to ensure consistency across studies [11].

### 3.5 Future Perspectives

In addition to being therapeutics, EVs may be effective and safe drug delivery systems for various kinds of drugs, ranging from chemicals to proteins and mRNAs. Useful lessons learned from the successful manufacture and clinical application of virus‐based vaccines and mRNA‐containing LNPs [439], can potentially be applied to EVs to overcome their inherent limitations. Careful investigation of EV pharmacology is essential to clarify not only their therapeutic function but also their delivery efficacy before moving to clinical trials. Once this parameter is understood, we can develop suitable formulations of EVs with optimal safety, efficacy, and stability.

Currently, the number of EV‐related clinical trials receiving US FDA approval remains modest due to long‐term safety concerns. The US FDA fast‐track process that addresses serious conditions and unmet medical needs [440] such as rare fatal diseases represents a potential area in which the field should focus [441]. Demonstrating safety in this area may lead to enhanced approvals for EVs in other areas of regenerative medicine.

## 4 Tissue Engineering: Applications and Challenges

Tissue engineering represents a pivotal advancement in regenerative medicine, aiming to restore or replace damaged tissues and organs through innovative biological and engineering approaches. Since its emergence in the 1980s, the field has undergone transformative progress marked by foundational innovations such as stem cell discovery and scaffold development, which established the framework for tissue regeneration. Advances in key technologies such as 3D bioprinting, iPSCs, and organoid models have enabled the creation of complex, patient‐specific tissue solutions. Significant clinical applications, for example, the development of bioengineered cardiac patches, bone grafts, and lung tissue, address critical needs in cardiovascular, orthopedic, and pulmonary medicine. Despite substantial progress, challenges such as biocompatibility and immune rejection, vascularization, bioactivity, and scalable production remain. Advances in scaffold design, smart biomaterials, gene editing, and organ‐on‐a‐chip systems are driving the field forward. Emerging trends, such as smart biomaterials and personalized medicine, aim to address these issues, making tissue engineering a transformative solution for critical medical needs.

### 4.1 The Fundamental Concepts of Tissue Engineering

Tissue engineering is an interdisciplinary field that uses knowledge from multiple disciplines, including biology, chemistry, and engineering, to construct functional biological alternatives for the restoration, repair, and replacement of impaired or lost tissues and organs [442]. The progression of tissue engineering has been marked by a series of milestones that have influenced its emergence as a key domain in regenerative medicine. In the early 1980s, the invention of the first scaffolding models to serve as a structural support to facilitate cell growth and tissue regeneration officially established the notion of tissue engineering and heralded a new path for cellular therapies [443, 444]. In 1984, Gallico et al. [445] demonstrated that in vitro tissue engineering could be employed for therapeutic purposes with the first successful transplantation of cultured human epithelium. In the early 2000s, biocompatible scaffold development enabled clinical use of engineered tissues for skin regeneration [23].

In 2006, the success of tissue‐engineered autologous bladder transplants marked the next milestone in the fabrication and therapeutic use of more complex engineered organs [23]. Additionally, 3D bioprinting emerged as a transformative technology, enabling the precise layering of cells and biomaterials to form advanced tissue structures. This technology addressed critical challenges in tissue engineering through the precise spatial deposition of biomaterials, cells, and bioactive molecules to engineer complex tissue architectures, including vascularized networks and multilayered structures [446]. Recent innovations in bioinks and multimaterial printing have further amplified their impact, enhancing the biocompatibility, structural integrity, and scalability of bioprinted constructs [447]. Given the global demand for the supply of transplantable organs, 3D bioprinting is at the forefront of regenerative medicine, revolutionizing personalized healthcare and cementing its position as a driver of innovation in the field. For example, Kang et al. [448] demonstrated the fabrication of human‐scale bone, cartilage, and muscle tissue by combining biodegradable scaffolds with cell‐laden bioinks, opening new perspectives for personalized regenerative treatments.

Continuous progress in tissue engineering has rapidly transformed the therapeutic landscape for multiple diseases. In cutaneous wound healing, new fabrication technologies that employ biomaterials combined with stem cells have significantly enhanced cell engraftment and tissue regeneration [449]. In orthopedics, innovative scaffold technologies promote the regeneration of bone and cartilage, offering effective alternatives to traditional treatments for OA and skeletal injuries. Cardiovascular applications, such as bioengineered cardiac patches, enable the replacement of fibrotic tissue post‐MI and accelerate heart tissue repair [450]. Similarly, the development of bioartificial pancreases holds promise for restoring endogenous insulin production [451]. In addition to tissue and organ replacement, miniature in vitro models of organs called organoids represent a new frontier in studies on tissue anatomy, drug discovery, and disease modeling [452, 453]. While remarkable advancements have been achieved thus far, efforts to overcome existing limitations are essential to assure ongoing innovations and maximize the promising clinical impact of tissue engineering.

### 4.2 Key Factors in Regenerative Tissue Engineering

The three main components that are fundamental for the generation of tissue include scaffolds, cells, and bioactive factors to support tissue growth.

#### 4.2.1 Scaffolds

In natural tissue, scaffolds are ECM components that provide structural support to cells, mediating mechanical stability and regulating cellular functions. As a heterogeneous network consisting of fibrous glycoproteins, large proteoglycans, and small molecules, the ECM interacts with other components in vivo to aid morphogenesis and homeostasis [454]. The objective of scaffold design in engineered tissues is to closely mimic the architecture of the ECM in target tissues. Scaffolds are composed mostly of polymers that act as skeletons for cells to reside in and grow into 3D tissues. Since the interaction between cells and the ECM is one of the major hurdles in tissue engineering, considerable effort has focused on the design of an artificial ECM [449]. Recent innovations in tissue engineering call include newer fabrication techniques, such as the use of biodegradable scaffolds combined with stem cells for tissue formation. To increase safety, these scaffolds are designed to degrade gradually after they offer structural support for cells to attach to and proliferate.

Selecting an appropriate biomaterial is highly important for ensuring the mechanical integrity, biocompatibility, and functionality of 3D bioprinted models. The development of high‐performance 3D bioprinting depends heavily on the creation of advanced biomaterials, such as biocompatible matrices encapsulating living cells for building the structures of tissues. Modern biomaterials for TERM possess many properties, including suitable surface roughness and chemistry for cell attachment, a 3D interconnected porous network for cell infiltration and nutrient metabolism, simulating the formation of the ECM, controllable degradation matching cell growth and ECM formation, mechanical properties to match those of tissues at the site of implantation, and a variety of shapes and sizes [455].

##### 4.2.1.1 Bioactive Ceramics (Bioceramics)

In recent decades, bioceramics, including hydroxyapatite (HAp), zirconia, alumina, tricalcium phosphates and bioactive glasses, have gained special interest in musculoskeletal regeneration. Owing to their high biocompatibility with cells and good ECM interaction with bone, bioceramics have been frequently used in hard tissue regeneration [455 *]*.

##### 4.2.1.2 Polymers

Polymers, both natural and synthetic, are particularly effective for soft tissue regeneration, including cardiovascular and skin tissues, due to the ability to control porosity and degradation rates. Natural polymers such as alginate, gelatin, and collagen are widely used because of their biocompatibility and ECM‐mimicking properties. However, alginate often needs to be combined with other materials to support cellular interactions, as it lacks cell‐adhesive motifs. Recently, hybrid systems that integrate alginate with cellulose nanofibrils have improved mechanical strength and printability, expanding their use in tissue engineering [456].

Synthetic polymers such as poly(L‐lactic acid), poly(L‐lactic‐co‐glycolic acid) (PLGA), poly(ε‐caprolactone (PCL), and polyurethane (PU) are also of interest because of the ability to control mechanical properties and degradation rates. Owing to the availability of the click‐chemistry technique and versatile poly(ethylene glycol) (PEG), novel copolymer structures with new functions to meet tissue‐specific requirements have been created. For example, polymers can be functionalized with peptides to increase cell adhesion. The incorporation of gelatin methacrylate (GelMA) with PEG could further improve cellular interactions, increasing the feasibility of bioprinting applications [457].

##### 4.2.1.3 Decellularized ECM

The decellularized ECM (dECM) has attracted considerable attention in tissue engineering because of its ability to mimic the intricate composition of the native ECM. This intricacy plays a crucial role in providing biochemical cues that direct cell differentiation and tissue‐specific functions [458]. Recent advances in the processing of dECM have increased their solubility and printability, enabling fabrication of constructs similar to the native tissue environment. Zhe et al. [459] discussed the utilization of dECM bioinks in 3D bioprinting for tissue engineering by means of creating an enabling environment that permits repair and tissue regeneration.

#### 4.2.2 Cells and Their Communication with Engineered Scaffolds

The microenvironment directly influences cell behavior and mobility and promotes intercellular interactions through biochemical cues and mechanical support. Cells can sense and respond to mechanical and biochemical stimulation from the ECM or alter their activities in terms of cell growth, movement, and differentiation.

TERM employs various cell types, among which MSCs, iPSCs, and progenitor cells are the most commonly used, on the basis of targeted tissue and therapeutic aims. Each cell type possesses distinct regenerative capabilities and unique methods of interaction with scaffolds to achieve tissue repair and integration. Notably, the multipotent nature cellular candidate for tissue engineering. MSCs can be derived from BM, UCB, and AT and can differentiate into different cell types, including osteoblasts, chondrocytes, and adipocytes, that aid in the repair of bone, cartilage, and soft tissue [460]. The application of MSCs in TERM for inflammatory conditions is also highly attractive because of their ability to suppress local immune responses while promoting regeneration [461]. MSCs can be driven toward specific targeted lineages when seeded on scaffolds with tailored structural and biochemical cues. For example, a stiffer scaffold increases osteogenic differentiation in bone repair, whereas a softer matrix influences the ability of MSCs to differentiate into chondrocytes and adipocytes [462]. Recently, iPSCs have emerged as the next revolutionary cell source in tissue engineering because of their patient specificity and capacity to differentiate into most cell types [463]. The use of iPSCs derived from patients via a tissue engineering therapeutic approach helps minimize the risk of triggering a graft versus host immune response, hence enabling personalized and immune‐compatible tissue repair. iPSCs also offer the unique advantage of facilitating the regeneration of multiple highly specialized cell types that require high cellular adaptability, such as neural and cardiac muscle tissues [464, 465]. Ozcebe et al. [466] demonstrated that iPSCs could differentiate into neural cells and cardiomyocytes when cultivated with neural or cardiac scaffolds that mimicked the neural or cardiac ECM microenvironments, respectively. Finally, progenitor cells, while lineage committed, still have capacity to proliferate and differentiate within that lineage. This is essential in TERM for target‐specific regenerative functions; for example, neural progenitors commonly used for brain repair or endothelial progenitors involved in vascularization. Furthermore, EPCs have been shown to support neovascularization/angiogenesis processes, aiding the nutrient supply in regenerating tissues [467].

In TERM, the relationship between cells and scaffolds is crucial, as cells are dependent on both biochemical and mechanical signals of the ECM for their differentiation, proliferation, and migration. The stiffness, topography, and bioactivity of the scaffold affect the cellular response and mechanotransduction, a process wherein cells transduce mechanical signals into biochemical responses [468]. For example, MSCs cultured on scaffolds that reproduce the elastic and viscoelastic properties of the cartilage ECM exhibit chondrogenic differentiation and promote cartilage formation. In contrast, highly aligned and denser scaffold structures often promote tenogenic differentiation and are employed to guide MSCs toward tendon‐like tissue formation [469]. These cues are perceived by cells through mechanotransduction, altering their behavior to ensure effective tissue repair. Kim et al. [470] reported that scaffolds that replicate natural ECM dynamics promote faster tissue healing by enhancing cell adherence and functional integration of tissue. Understanding cell–ECM interactions is critical for developing strategies that can optimize tissue regeneration, hence improving therapeutic results.

Another key consideration in TERM is to mimic natural cell–scaffold communication by designing biomaterials that support efficient tissue repair. In addition to structural support, the ECM also provides biochemical and mechanical signals through which cells can proliferate, migrate, and differentiate. A structured scaffold microenvironment has been shown to improve tissue regeneration by enhancing mechanotransduction, a vital process for facilitating cell alignment and faster healing in engineered tissues [471]. Recently, the incorporation of EVs, which are known to modulate the behavior of cells through delivery of growth factors and other signaling molecules into target cells, has become a state‐of‐the‐art innovation in scaffold design that helps researchers more closely mimic natural cell–ECM communication and improve tissue integration and therapeutic outcomes [472].

### 4.3 Selected Applications of Tissue Engineering

**FIGURE 7:**
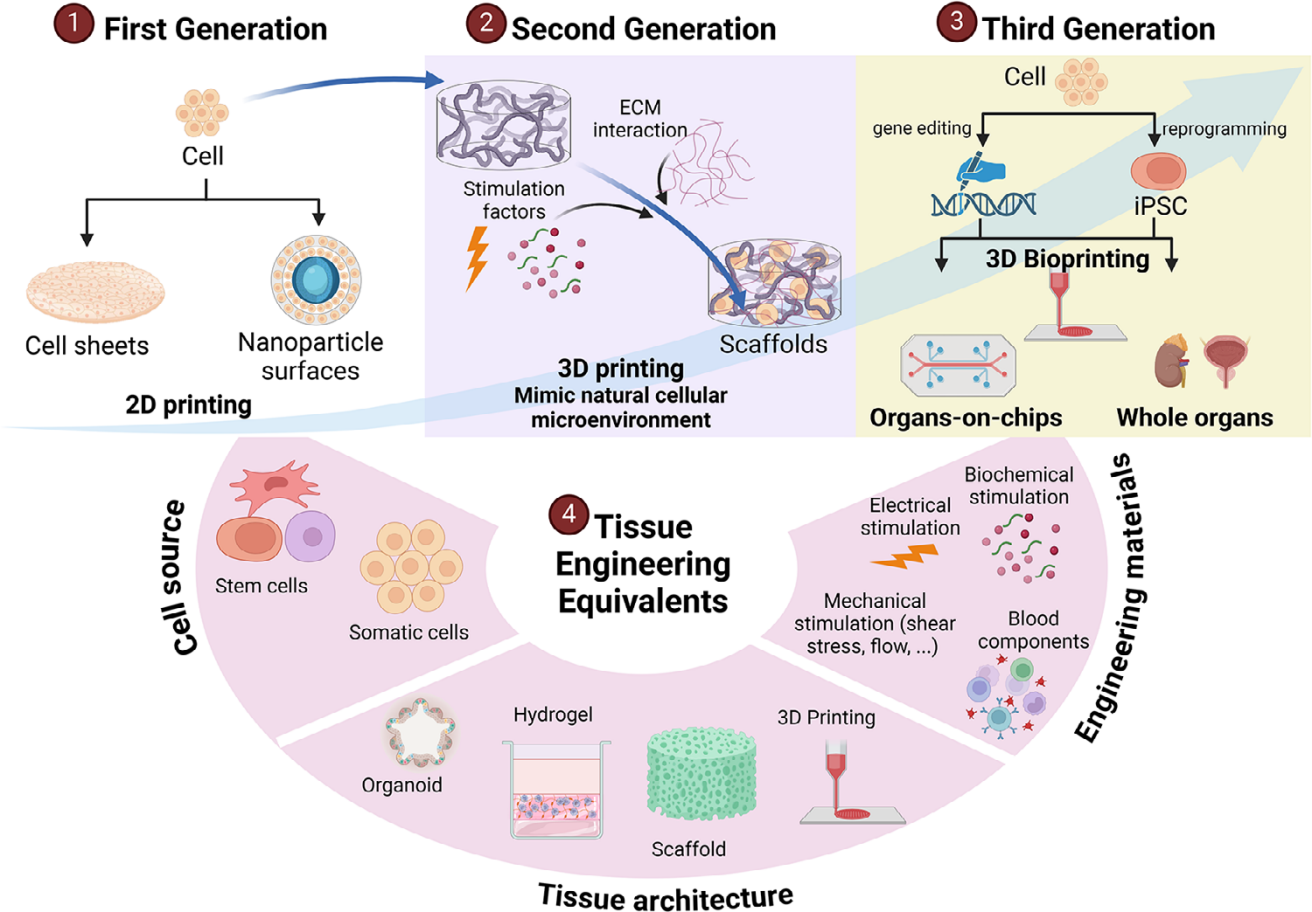
**Generations and core components in tissue engineering**. The three upper panels show the progress of tissue engineering technologies. The first generation focuses on 2D biomaterial surfaces, such as nanoparticle coatings and cell sheets, enabling basic cell adhesion and growth. The second generation introduced 3D scaffolds and emphasized cell signaling and ECM interactions, which better mimic the cellular microenvironment and support vascularization and tissue growth. Third‐generation, or patient specific, engineering now combines autologous or iPSC‐derived cells, gene editing, and advanced 3D constructs, including bioprinting and organ‐on‐chip systems, aimed at creating personalized tissues for regenerative medicine. The bottom panel depicts essential tissue engineering components for creating functional tissue models, including cell sources (stem cells or cell lines), tissue architecture (e.g., hydrogels, 3D printing, scaffolds, organoids), and engineering materials (biochemical, electrical, and mechanical stimuli plus blood components). ECM extracellular matrix. *Abbreviations*: extracellular matrix (ECM) and induced pluripotent stem cells (iPSCs).

Tissue engineering has evolved through three main generations, each representing significant advancements in therapeutic capabilities (Figure 7). The first generation, which emerged in the 1980s and 1990s, was characterized by the development of two‐dimensional (2D) systems such as nanoparticle surfaces and cell sheets. These approaches are designed primarily to deliver bioactive molecules or replace damaged cells, aiming to restore tissue function [473]. However, physiologically, a 2D layer of cells, as well as a 2D‐biomaterial approach, cannot mimic the natural behaviors of cells and tissues, resulting in limited outcomes, as noted by Langer and colleagues [474].

In the 2000s, the second generation of tissue engineering introduced three‐dimensional (3D scaffolds, which represented an important turning point toward more sophisticated regenerative strategies. These 3D scaffolds can better replicate the natural ECM, facilitating more physiological cell–cell, cell–material, and cell–structure interactions [473]. These scaffolds support in vitro angiogenesis, resulting in improved oxygen and nutrient diffusion and increased cell proliferation. Furthermore, the 3D structures guide cells to grow in typical patterns specific to each tissue, enhancing their functional integration [473]. This area was further advanced by use of mechanical stimulation by bioreactors and pulsatile pumps that simulate physiological forces, hence allowing the generation of engineered small‐diameter blood vessels [475]. For example, Niklason et al. [476] grew cells in bioreactors and then decellularized them before implanting back into patients. Similarly, the Langer group incorporated NSCs into a 3D scaffold to form microtissues that were subsequently implanted in nonhuman primates and clinically in humans [477, 478]. By mimicking the tissue microenvironment, including mechanical and biochemical signals, second‐generation tissue engineering has promoted more effective tissue regeneration.

The third generation of tissue engineering is patient specific, focusing on creating personalized tissues and organs via techniques such as autologous stem cells or allogeneic iPSCs, often from superdonors, combined with gene editing. This approach integrates advanced systemic considerations such as multiorgan‐on‐a‐chip systems, immune cell interactions, and vascularization. Highly sophisticated architectures—such as cell‐instructive organization, responsive biomaterials, and 3D bioprinting—enable the formation of complex, functional tissues and organs, including structures such as ears and bladders, tailored to individual patient needs [473].

#### 4.3.1 Musculoskeletal Tissue Engineering

Musculoskeletal tissues are one of the most regenerative tissues in the human body. These tissues can self‐heal at a relatively fast pace; however, in cases of severe injury resulting in volumetric muscle loss greater than 20%, the functional and mechanical properties of the tissue cannot recover without external intervention. 3D bioprinting is a practical method to provide a solution for severely damaged musculoskeletal tissues.

A wide variety of materials have been used for skeletal muscle regeneration. Biomaterials in bone tissue engineering provide the structural framework for regeneration, mimicking the physical and biomechanical properties of bone to support new tissue growth. Natural bioinks include gelatin, GelMA, xanthan gum, alginate, hyaluronic acid, fibrin, and dECM. Synthetic bioinks include polyglycolide (PGA), PCL, PLGA, poly(lactic acid) (PLA), PEG, poly(vinyl alcohol), and PU [479]. Currently, the most commonly used methods for the deposition and patterning of biological materials in 3D bioprinting include microextrusion, inkjet printing, and laser‐assisted printing. In terms of cell seeding technology, 3D bioprinting can be performed either with or without live cells (referred to as cellular or acellular bioprinting, respectively). The former incorporates both cells and bioreagents simultaneously during structuring, whereas the latter builds a 3D scaffold to allow host cells to integrate and penetrate for growth [480].

Omar et al. [481] studied 3D‐printed large cranial bone patches versus titanium patches in sheep and discovered that the printed bone patches, despite lacking cells and growth factors, resulted in better bone growth and a more uniform composition compared with titanium patches. The group next collected autologous bone and printed a cranial patch to cover a 13.4 × 11 cm (115 cm^2^) cranial defect in a 22‐year‐old male patient. At 21 months postimplantation, the patient's skull bone had recovered in terms of mechanical properties, structure, and uniformity like those of native bone, as confirmed by X‐ray, micro‐CT, histomorphometry, Raman spectroscopy, and electron microscopy [481]. Porous tantalum, which is effective for bone and joint replacement, promotes the adhesion, proliferation, and osteogenic differentiation of BM‐MSCs by activating the MAPK/ERK signaling pathway and regulating osteogenic genes such as ALP, type I collagen, osteonectin, and osteocalcin [482]. A retrospective analysis by Edelman et al. [483] of 82 patients demonstrated that porous tantalum significantly enhances bone fusion and offers strong orthopedic support in clinical applications. The use of biocompatible scaffolds in combination with cells has also been applied for regenerating bone defects. A concise review by Re et al. [484] reported five clinical trials in which MSCs and scaffolds were used for the treatment of bone defects, including lumbar degenerative disc disease, deep infrabony defects, maxillofacial bone defects, and femoral bone defects. BM‐MSCs are the most commonly used cells in bone tissue engineering, with calcium phosphate ceramics, such as β‐tricalcium phosphate, frequently serving as scaffolds. Clinical trials have consistently validated this approach for treating bone conditions, including fractures of the femur, tibia, and humerus, as well as lumbar degenerative disc disease, infra‐bony defects, and maxillofacial bone deficiencies [484].

#### 4.3.2 Lung Tissue Engineering

Recent progress in lung tissue engineering has placed 3D bioprinting at the center of translational technologies that can overcome donor shortages and immune rejection complications associated with lung transplantation [485]. These advancements are centered on mimicking the complex architecture and functionality of the lung, which is made possible by the use of 3D bioprinting to fabricate intricate, multilayered lung tissues. With the advent of sophisticated bioinks that incorporate live cells, hydrogels, and ECM proteins, researchers have enabled the fabrication of alveolar structures and bronchial networks that closely mimic native lung tissue. A breakthrough involves the fabrication of 3D‐printed alveolar structures embedded with epithelial and endothelial cells exhibiting the critical cell‐specific activities required for oxygen and carbon dioxide exchange, which helps reduce the reliance on donor organs and creates more physiologically relevant models for research and clinical applications [486]. Furthermore, recent studies on decellularization methods have aided in preserving the ECM and mechanical properties of the lung after decellularization to create structures that encourage faster cell repopulation and restore lung function [487]. Techniques such as vacuum‐assisted and apoptosis‐assisted decellularization have enabled the removal of cellular components while minimizing damage to the ECM [488]. This preservation aids in the construction of scaffolds that support functional recellularization with stem cells, hence promoting regeneration in manners similar to those in natural lung tissue [487]. Another crucial breakthrough in lung tissue engineering is the incorporation of iPSCs and MSCs, which represent two of the most promising cellular sources for lung cells through which both alveolar type I and II cells and endothelial cells can be generated [489, 490]. To further optimize lung models, novel scaffolds constructed from biodegradable polymers and natural materials such as collagen and decellularized lung matrices have been employed to provide structural support and enhance cell adhesion and differentiation for improved tissue integration [491]. Li et al. [491] presented a method of thermally induced phase separation, which allows the building of highly porous, architecturally controllable scaffolds to enhance the integration and proliferation of lung cells. Additionally, more emphasis has been placed on the use of bioactive scaffolds engineered to release growth factors and cytokines with the intention of enhancing cellular proliferation and vascularization in engineered lung tissues. For example, scaffolds designed to contain molecules such as VEGF can stimulate angiogenesis and tissue regeneration and significantly enhance the structure and function of bioengineered lungs [470]. These recent innovations in the field of lung tissue regeneration have addressed key considerations involving cell integration, vascular network development, and prolonged functionality.

#### 4.3.3 Cardiac Tissue Engineering

The adult mammalian heart is known as one of the least regenerative organs because of its limited self‐repair capacity [492]. To address this issue, 3D bioprinting has recently emerged as a breakthrough technology in cardiac tissue engineering (CTE), featuring precise control of the fabrication of cardiac tissues with enhanced regenerative potential. A central aspect of 3D bioprinting in CTE involves developing sophisticated bioinks with incorporated bioactive materials that can replicate the native ECM and cellular signaling. A notable innovation is hybrid hydrogels that incorporate natural polymers such as collagen with conductive materials such as graphene oxide or carbon nanotubes [493]. These materials aid in promoting electrical conductivity, an ongoing need for both contractile function and cardiomyocyte synchronization [493]. In an effort to more closely replicate the native cardiac tissue structure, nanofiber scaffolds have been developed to support mechanical strength and cellular alignment [494]. In recent years, CTE has been increasingly utilized by human iPSCs because of its ability to differentiate into a diverse range of cell types for cardiac repair. Wesley et al. [495] reported that the maturity and functional characteristics of hiPSC‐derived cardiomyocytes can be enhanced when seeded in a 3D scaffold with additional mechanical and electrical stimulants. Furthermore, recent findings have focused on building a more intricate cardiac environment by combining cardiomyocytes with other supporting cell types, including endothelial cells, fibroblasts, and immune cells, to engineer a more complex cardiac microenvironment [496]. This novel approach helps further promote tissue integration and angiogenesis after transplantation into host cardiac tissues [496].

A multitude of early‐phase clinical applications have evaluated CTE, including injectable hydrogels and cell patches for patients with ischemic heart disease [497] transplantation of decellularized whole heart scaffolds repopulated with the host's cardiomyocytes [498], 3D‐bioprinted cardiac tissues, and use of cell therapy and gene editing to enhance the reparative capabilities of seeded cells [497]. With respect to cardiac patches, Zhang et al. [499] successfully delivered injectable hydrogels to damaged heart tissues using minimal invasive surgical techniques. Therapeutic molecules were slowly released, allowing cardiac regeneration to occur gradually [499]. Another breakthrough in CTE is the successful 3D bioprinting of a patient‐specific heart patch using bioinks containing the host's own cells and the ECM derived from their AT [498]. This innovative approach optimized the bioinks through incorporation of supportive ECM and the host's cardiomyocytes at a level that would allow the engineered tissue to maintain the electrical and mechanical properties necessary for heart function, hence eliminating the risk of immune rejection and promoting repair in the heart tissue suffering from a MI [498].

While there are challenges to overcome on the path to full functional integration and scaling production for clinical use, continued studies into bioink optimization, cell coculture methods, and immune modulation strategies are key for future human clinical trials. Decellularized ECM has advanced with xenografts to improve availability and feasibility. Sander and colleagues decellularized porcine heart aortic valves using a standardized protocol, then created a 27.8 by 29.7 mm prototype using simulation and design software [500, 501]. The engineered heart valve was seeded with primary vascular‐derived cells from a human patient, using fibrin as a cell carrier, and pulsatile flow at 1 Hz. The valve's hydrodynamic functionality, durability for 3 million cycles, and collagen alignment were confirmed, marking a significant milestone for xenoengineering.

##### 4.3.3.1 Engineering Total Artificial Heart

Since the first mechanical heart transplant with the Liotta‐Cooley mechanical heart, in 1969, which kept the patient alive for 64 h [502], heart engineering has continued to develop. The 1980s saw the development of the total artificial heart (TAH) by the University of Utah. William C. DeVries and Willem J. Kolff developed the Jarvik‐7 and transplanted it into a 61‐year‐old man with chronic congestive heart failure [503]. Despite normal blood pressure and cardiac output, the patient experienced complications and died after 112 days. The TAH has since evolved, with the Jarvik‐7 prototype leading to commercial versions like SynCardia, AbioCor TAH, and CARMAT TAH, all US FDA‐approved as a bridge to transplant [502, 504]. However, TAH outcomes remain limited, with the longest life extension 1374 days (4 years) [502, 505]. Torregrossa and colleagues [505] reported a 10% device failure rate and 24% of the patients died on device support in addition to other AEs including systemic infections (53%), driveline infections (27%), thromboembolic events (19%), and hemorrhagic events (14%). TAH implants are still limited as temporary transplants due to device size and patient‐specific blood circulation needs.

Xenotransplantation of genetically humanized hearts is especially needed for patients with heart failure until suitable cardiac allografts are available. The first xenotransplantation occurred in 1984 with transplantation of a baboon heart into a human infant, who suffered from hypoplastic left heart syndrome [506]. However, the baby survived only 20 days due to graft failure [507]. Subsequently, scientists favored xenotransplanted porcine hearts over nonhuman primates (NHPs) due to the decreased risk of transmitting pathogens like simian immunodeficiency virus, which can transform into HIV [508]. The first cardiac xenotransplantation of wild‐type pigs into humans in 1997 resulted in a patient surviving 1 week before hyperacute rejection [509]. Host immune cells recognized xenoantigens on porcine vascular endothelial cells, leading to the complement mediated activation endothelial injury and activation of the coagulation cascade, causing graft failure. Decades later, Dr. Bartley Griffith performed two xenotransplantations of genetically modified pig hearts with patients surviving 2 months and 6 weeks, respectively [508]. While few engineered xeno‐TAH strains have been tested due to knowledge limits and ethical concerns, the topic remains promising as a potential solution for millions of patients worldwide.

#### 4.3.4 Tissue Engineering of Other Tissues and Organs

In 2006, Atala and colleagues [23] at Wake Forest University conducted the first clinical test of a 3D‐bioprinted bladder made from patient cells. Seven patients with myelomeningoceles underwent bladder reconstruction via autologous cells cultured on a collagen‒polyglycolic acid scaffold, with or without an omental wrap. The results revealed significant improvements in bladder pressure, volume, and compliance after reconstruction with engineered tissue, with no metabolic or renal complications, indicating enhanced bladder function [23]. However, a later study by Joseph et al. [510] using a PGA/PLA composite scaffold in 10 patients with spina bifida did not show similar improvements, with serious complications such as bowel obstruction and bladder rupture in some patients. Further investigations are necessary to confirm the safety and efficacy of tissue‐engineered grafts for augmentation cystoplasty. While initial studies have shown promise, larger, longer‐term clinical trials are needed to validate these findings and assess potential complications, ensuring that these innovative approaches can reliably improve patient outcomes.

Several researchers have reported the use of 3D‐printed ear scaffolds for whole ear replacement. It is important to approach ear reconstruction in a patient‐specific manner. Using CT scans of the ears of seven children aged 11–16 years and advanced software to reconstruct personalized ear anatomy, Jeon et al. [511] were able to print a functional external ear, becoming a better solution for microtia reconstruction than casted ear models. In addition to patient‐customized size, Mannoor et al. [512] also created an ear that exhibited aesthetic similarity to a real ear and possessed enhanced auditory sensing capabilities. Further investigation by Xie et al. [513] on a microtissue bioink ensured optimal cell viability, tailored specifically for digital light processing bioprinting. For clinical applications, Joo et al. [514] designed patient‐specific, 3D‐bioprinted ear implants for both the first and second stages of ear reconstruction surgeries. In 2022, 3DBio Therapeutics (New York, NY) successfully implanted AuriNovo, the first autologous auricular implant created through 3D bioprinting, in a 20‐year‐old woman. Using her own cartilage cells, the team created a 3D‐printed ear designed to match the shape and size of her left ear. This trial marks a significant step forward in tissue engineering and the potential for 3D‐bioprinting to address complex reconstructive needs beyond microtia, such as other cartilage and tissue replacements.

Tissue engineering has also enabled the generation of functional kidney tissue. In 2013, Song et al. [515] developed a method for decellularizing kidneys from rats, pigs, and humans to create acellular scaffolds. These scaffolds were populated with epithelial and endothelial cells and subjected to perfusion in a bioreactor. The engineered grafts produced urine in vitro and, when transplanted into rats and successfully perfused by the host's circulation, produced urine in vivo. In 2022, Shu et al. [516] also successfully generated bioengineered kidneys by using decellularized kidney scaffolds seeded with renal progenitor cells. In vitro, these bioengineered kidneys produced urine and demonstrated the ability to reabsorb albumin, glucose, and calcium. This study suggests that cell‐based kidney bioengineering with physiological secretion and reabsorption capabilities is feasible and holds significant therapeutic potential for kidney regeneration.

Several clinical trials have evaluated the safety and feasibility of tissue engineering and human islet transplantation for type 1 diabetes. ViaCyte (Vertex Pharmaceuticals) pioneered the transplantation of hESC‐derived pancreatic progenitor cells (NCT02239354). A subsequent trial (NCT04786262) evaluated hESC‐derived β cells implantation. Finally, a phase I/II clinical trial (NCT03163511) involving the transplantation of human pancreatic endoderm cells into a device was performed. Encouragingly, at the 26‐ and 52‐week marks post implantation, there was a noticeable increase in C‐peptide and insulin secretion, including postprandial C‐peptide secretion, in the recipients. These outcomes provide promising evidence for the feasibility of human islet transplantation.

### 4.4 Current Challenges

TERM has evolved significantly over the past several decades, yielding many groundbreaking advancements. However, several critical challenges persist, including identifying optimal cell sources, ensuring biocompatibility, developing specialized bioreactors, and constructing complex organs. A deeper understanding of how tissues regenerate in new environments is essential for overcoming barriers to successful tissue engraftment. Engineered constructs must utilize biomaterials that are both biocompatible and biodegradable, allowing for the gradual breakdown of implantable tissues while promoting regeneration. Addressing these obstacles is crucial to achieving the clinical efficacy, durability, and safety of tissue‐engineered therapies.

#### 4.4.1 Biocompatibility

Enhancing biocompatibility is a fundamental step in developing effective solutions for tissue engineering. Significant progress in TERM has been achieved through the development of advanced biomaterials, innovative technologies, and the identification of novel cell sources. Scaffolds must mimic the mechanical, structural, and biochemical properties of the ECM in target tissues. However, achieving the right balance between biocompatibility, biodegradability, and mechanical strength remains difficult. Many synthetic biomaterials degrade too quickly or trigger inflammatory responses, whereas natural materials may lack the necessary structural integrity. New methodologies have been developed to find biomaterials with enhanced biocompatibility. For example, in the polymer field, naturally derived materials such as acellular matrices, collagen, gelatin, hyaluronic acid, chitosan, silk, and alginate are commonly used to fabricate scaffolds for TERM [517, 518]. New synthetic polymers, such as poly(ether ether ketone), have also been developed to increase biocompatibility [519]. Hiromoto's group [520] employed octacalcium phosphate and HAp coatings to regulate the degradation rate and enhance the biocompatibility of biodegradable magnesium alloys. Zheng and colleagues [521] reported that Fe‐based scaffolds exhibited excellent long‐term biocompatibility in both porcine and rabbit models. Certain composite materials, including those that combine organic and inorganic components, can enhance bioactivity [522]. For example, an adhesive derived from the skin secretion of *Andrias davidianus* demonstrated superior elasticity and biocompatibility compared with cyanoacrylate glue in the development of artificial skin [523]. In the field of dental regeneration, Lee and Kim developed a Sr ion‐releasing nanobiocement (Sr‐NBC) via the sol‐gel method. This Sr‐NBC exhibited remarkable biocompatibility, high odontogenic potential in vitro, and facilitated new dentin formation in vivo [524].

#### 4.4.2 Immune Rejection

When cells from allogenic sources or genetically modified autologous cells are administered to recipients, they encounter the innate immune system, which includes NK cells, dendritic cells, and the complement and coagulation system. This exposure can trigger a quick and nonspecific immune response, leading to the clearance of donor graft cells [525, 526]. Additionally, the adaptive immune system can mediate immune rejection. Dendritic cells process and present antigens recognized as foreign on the surface of transplanted cells, leading to activation of helper CD4^+^ T cells and cytotoxic CD8^+^ T cells. While cytotoxic T cells destroy allogenic cells via cytotoxic substances, helper T cells assist in this process by releasing several cytokines to stimulate cytotoxic T cells. Cytokines also act on the innate immune system, causing inflammation and promoting B‐cell maturation into plasma cells. These plasma cells produce alloantigen‐specific antibodies, resulting in antibody‐mediated rejection of the cellular therapy [525, 526]. Addressing these challenges involves the selection of HLA‐matched grafts, strategic use of immunosuppressive drugs or cotransplantation of immunomodulatory cells, genetic modification of immune cells to evade detection, and/or encapsulation of therapeutic cells to mask incompatibility and enhance efficacy [527, 528].

#### 4.4.3 Bioactivity

Another challenge in developing biomaterials for TERM is engineering functional materials, which involves exploring methods to assess the cellular functionality and bioactivity of implants. Several challenges must be addressed to optimize clinical applications. For examples, insufficient vascularization can result in poor engraftment [529]. Current tissue fabrication methods result in stacking of multiple tissue layers that do not recapitulate the complexity of human tissues, which are composed of multiple cell types and layers that interact with other tissue types (e.g., bone‐cartilage and tendon‐muscle) [530]. Optimal cell density and the immaturity of engineered structures are also significant obstacles in clinical practice [531].

#### 4.4.4 Feasibility

The scalability and reproducibility of tissue engineering products are also major obstacles. Ultimately, the widespread clinical translation of engineered tissue constructs will be limited by the ability to generate sufficient tissue in a GMP‐compliant manner. To date, significant progress has been made in scaling up the production of implantable tissues. For example, 3D bioprinting technologies have enabled the creation of large, implantable constructs [23]. However, tissue engineering technologies often require expensive equipment, highly specialized expertise, and prolonged development timelines, making them inaccessible to many healthcare systems and patients. The high cost of raw materials, such as growth factors and biomaterials, creates further challenges in the large‐scale application of TERM.

### 4.5 Strategies to Overcome Challenges

Tissue engineering relies on scaffolds to support the development of new, functional tissues for medical applications [532]. Traditional tissue replacement approaches include enhancing natural repair mechanisms via growth factors and miRNA therapies [532], organ transplantation (such as liver, heart, kidney, or BM transplants) [533], and the implantation of organs derived from genetically‐modified swine [508]. With advancements in technology, innovative strategies have emerged to restore damaged organ functions, such as production of artificial organs for organ transplantation [534]. These include the creation of artificial organs for transplantation, the use of biocompatible materials such as bioglass‐based grafts that integrate with bone to promote healing [535] and the application of nanomaterials to design bone and dental implant surfaces or fibers that replicate the structure of natural bone [536].

Tissue engineering has achieved significant progress over the past decade and continues to advance rapidly. However, the range of tissues that are successfully regenerated and widely used in clinical applications remains limited to structures such as skin, bone, cartilage, capillaries, and periodontal tissues. Overcoming the challenges in TERM will be essential for driving further innovation and expanding its potential.

#### 4.5.1 Cells for TERM

The extension of cellular sources for TERM is a potential solution. To date, various multipotent cells, including autologous adult cells, ESCs, BM‐derived stem cells, tissue‐specific progenitor stem cells, UC‐derived stem cells, and iPSCs, have demonstrated effectiveness in TERM [537, 538]. Additionally, the genetic modification of stem cells through gene therapy can increase their efficiency, mitigate immunogenicity, and accelerate tissue regeneration. For example, MSCs modified with lentiviral constructs have been shown to promote cell differentiation, facilitating more effective bone repair [539]. The application of CRISPR gene editing technology has enabled researchers to generate novel cell sources for use in bone tissue engineering studies [540]. Gene editing has also been utilized to modify the ECM produced by stem cells. By targeting specific genes, researchers can alter the composition of the ECM, making it more conducive to supporting tissue regeneration. This strategy enables the customization of the cellular environment to more closely replicate natural tissue, thereby enhancing the integration and functionality of engineered tissues [541]. Another strategy involves the use of gene editing tools to promote vascularization and angiogenesis, such as by upregulating VEGF to increase vascularization in engineered tissues [542]. This is essential for the survival and integration of large or complex tissue constructs [541]. Another strategy involves the use of gene editing tools to promote vascularization and angiogenesis, such as by upregulating VEGF to increase vascularization in engineered tissues [542]. This is essential for the survival and integration of large or complex tissue constructs. Future stem cell research will likely focus on guiding stem cells to differentiate into specific cell types that closely mimic the functions of adult cells [543]. These advancements in stem cell technology are expected to play a pivotal role in driving significant progress in the field of TERM.

#### 4.5.2 Biomaterials for Scaffold Constructs

Artificial tissues and organs require improved biocompatibility and functionality to meet clinical demands. Various methods have been developed for constructing biomaterial‐based scaffolds, with 3D bioprinting and microfluidics technologies standing out for their precision and ability to create personalized materials and complex tissue constructs. For example, CRISPR‐modified cells are incorporated into bioinks for 3D bioprinting to ensure tissue‐specific properties, enhancing the functionality of printed constructs [544]. In addition to fabrication techniques, advancements in chemical and material science have significantly accelerated the development of biomaterial‐based scaffolds. Chemical modifications enhance the properties of existing materials, and new materials have been engineered for applications in TERM. These materials are processed into hydrogels, porous scaffolds, and fibers to address the diverse requirements of TERM.

Moving forward, the development of functional biomaterials tailored for specific tissue types will be a key focus. Another major challenge is achieving vascularization in implanted tissues and organs. Replicating the microenvironment of native tissues is essential for successful integration and functionality. This can be accomplished by incorporating bioactive factors such as growth factors, chemokines, cytokines, and external stimuli, including electrical, mechanical, and magnetic signals, to enhance the microenvironment and promote vascularization [545, 546]. These approaches are expected to significantly advance the field, enabling the creation of more effective tissue‐engineered solutions.

#### 4.5.3 Clinical Translation

First, efforts to reduce immunogenicity and enhance implant integration must be prioritized. Improving our understanding of the role of the immune system in tissue regeneration could play a pivotal role in achieving these objectives. Additionally, exploring how factors such as age, disease state, and the patient microbiome impact regenerative processes will be essential for driving progress across various applications in the field.

Second, scalable technologies that ensure reproducibility in large‐scale tissue engineering are urgently needed. Compared with traditional batch methods, continuous microfluidic processes provide greater production throughput while maintaining precise control over the composition and size of microtissues [547]. Another promising strategy involves leveraging automated robotics to produce consistent populations of microtissues efficiently [548].

### 4.6 Future Perspectives

Emerging trends and technologies are significantly advancing the field of TERM. These include smart biomaterials, innovative stem‐cell sources, advanced 3D bioprinting, vascular engineering, sophisticated bioreactors, organoids, and microfluidics‐based physiological platforms. Notably, organs‐on‐a‐chip offer the potential to bioengineer damaged organs or address developmental anomalies [549]. Examples include liver‐on‐a‐chip, heart‐on‐a‐chip, and skin‐on‐a‐chip systems. By integrating multiple tissue types within a single device, researchers can create body‐on‐a‐chip systems for comprehensive physiological modeling. Furthermore, advancements in biomaterials, nanotechnology, and gene therapy are being integrated into these platforms, enhancing their functionality and expanding their applications [550]. Additionally, gene editing tools provide promising alternatives for TERM development, further driving the advancement of personalized medicine.

## 5 Concluding Remarks

The extensive history of TERM is marked by groundbreaking achievements and relentless efforts to translate fundamental scientific discoveries from the bench to the bedside. Stem cell therapy has been explored for applications in nearly every disease category. Recently, stem cell therapy has made significant progress through novel technologies for cell modification and the integration of biomaterial scaffolds. EV therapies demonstrate great potential in diagnostics, cell‐free therapeutics, drug delivery, and targeted therapy. These innovations have paved the way for more effective treatments and opened new avenues for research and clinical applications.

Tissue engineering has been accelerating with rapid developments in biomaterial science, computer science, and stem cell and gene technology. Innovations such as the discovery of iPSCs, genetic modification tools, and 3D bioprinting of organs have opened new frontiers in TERM, especially for personalized medicine. However, success in the clinical translation of artificial tissues and organs remains limited. Multidisciplinary research and interdisciplinary collaboration will pave the way for future developments in TERM.

## Author Contributions

V. T. H.: study conception and design, manuscript writing, administrative support, supervision of data analysis and interpretation, and final approval of the manuscript. L. N. T.: study conception and design, manuscript writing, administrative support, supervision of data analysis and interpretation, and final approval of the manuscript. Q. T. N.: study design, manuscript writing, data analysis and interpretation, and final approval of the manuscript. L. P. H. A., L. A. T. N., and H. N. D.: manuscript writing (cell therapy section) and final approval of the manuscript. D. T. H. N., P. T. K. T., V. D. B., H. N. D., and D. S. L.: manuscript writing (EV therapy section) and final approval of the manuscript. Q. D. L., L. T. M. D., and T. H. P.: manuscript writing (tissue engineering section) and final approval of the manuscript. All the authors have read and approved the article.

## Ethic statement

Not applicable.

## Conflicts of Interest

V. T. H., Q. T. N., D. T. H. N., P. T. K. T., L. P. H. A., Q. D. L., L. T. M. D., H. N. D., T. H. P., L. A. T. N., D. S. L., and L. N. T. are employed by the College of Health Sciences – VinUniversity and Vinmec Health Care System. V. D. B. works as an external advisor for the Vinmec Health Care System.

## Supporting information



Supporting Information

## Data Availability

All the data generated or analyzed in this study are included in this published article.

## References

[1] F. Han , J. Wang , L. Ding , et al., “Tissue Engineering and Regenerative Medicine: Achievements, Future, and Sustainability in Asia,” Frontiers in Bioengineering and Biotechnology 8 (2020): 83. 32266221 10.3389/fbioe.2020.00083 PMC7105900

[2] I. E. Konstantinov , “In Search of Alexander A. Maximow: The Man Behind the unitarian Theory of Hematopoiesis,” Perspectives in Biology and Medicine 43, no. 2 (2000): 269–276. 10804590 10.1353/pbm.2000.0006

[3] E. D. Thomas , H. L. Lochte Jr. , W. C. Lu , and J. W. Ferrebee , “Intravenous Infusion of Bone Marrow in Patients Receiving Radiation and Chemotherapy,” New England Journal of Medicine 257, no. 11 (1957): 491–496. 13464965 10.1056/NEJM195709122571102

[4] A. J. Friedenstein , R. K. Chailakhyan , and U. V. Gerasimov , “Bone Marrow Osteogenic Stem Cells: In Vitro Cultivation and Transplantation in Diffusion Chambers,” Cell and Tissue Kinetics 20, no. 3 (1987): 263–272, 10.1111/j.1365-2184.1987.tb01309.x. 3690622

[5] D. M. Hoang , P. T. Pham , T. Q. Bach , et al., “Stem Cell‐based Therapy for human Diseases,” Signal Transduction and Targeted Therapy 7, no. 1 (2022): 1–41. 35933430 10.1038/s41392-022-01134-4 PMC9357075

[6] K. Takahashi , K. Tanabe , M. Ohnuki , et al., “Induction of Pluripotent Stem Cells From Adult human Fibroblasts by Defined Factors,” Cell 131, no. 5 (2007): 861–872. 18035408 10.1016/j.cell.2007.11.019

[7] K. Sugai , M. Sumida , T. Shofuda , et al., “First‐in‐human Clinical Trial of Transplantation of iPSC‐derived NS/PCs in Subacute Complete Spinal Cord Injury: Study Protocol,” Regenerative Therapy 18 (2021): 321–333. 34522725 10.1016/j.reth.2021.08.005 PMC8427225

[8] S. Takagi , M. Mandai , K. Gocho , et al., “Evaluation of Transplanted Autologous Induced Pluripotent Stem Cell‐Derived Retinal Pigment Epithelium in Exudative Age‐Related Macular Degeneration,” Ophthalmology Retina 3, no. 10 (2019): 850–859. 31248784 10.1016/j.oret.2019.04.021

[9] K. L. McKinley , M. T. Longaker , and S. Naik , “Emerging Frontiers in Regenerative Medicine,” Science 380, no. 6647 (2023): 796–798. 37228215 10.1126/science.add6492 PMC10493035

[10] A. Nagelkerke , M. Ojansivu , L. van der Koog , et al., “Extracellular Vesicles for Tissue Repair and Regeneration: Evidence, Challenges and Opportunities,” Advanced Drug Delivery Reviews 175 (2021): 113775. 33872693 10.1016/j.addr.2021.04.013

[11] G. van Niel , G. D'Angelo , and G. Raposo , “Shedding Light on the Cell Biology of Extracellular Vesicles,” Nature Reviews Molecular Cell Biology 19, no. 4 (2018): 213–228. 29339798 10.1038/nrm.2017.125

[12] Y. Couch , E. I. Buzàs , D. D. Vizio , et al., “A Brief History of Nearly EV‐erything—The Rise and Rise of Extracellular Vesicles,” Journal of Extracellular Vesicles 10, no. 14 (2021): e12144. 34919343 10.1002/jev2.12144 PMC8681215

[13] R. E. Veerman , G. G. Akpinar , M. Eldh , and S. Gabrielsson , “Immune Cell‐Derived Extracellular Vesicles—Functions and Therapeutic Applications,” Trends in Molecular Medicine 25, no. 5 (2019): 382–394. 30853173 10.1016/j.molmed.2019.02.003

[14] E. Karnas , P. Dudek , and E. K. Zuba‐Surma , “Stem Cell‐ derived Extracellular Vesicles as New Tools in Regenerative Medicine‐Immunomodulatory Role and Future Perspectives,” Frontiers in Immunology 14 (2023): 1120175. 36761725 10.3389/fimmu.2023.1120175 PMC9902918

[15] D. E. Murphy , O. G. de Jong , M. Brouwer , et al., “Extracellular Vesicle‐based Therapeutics: Natural versus Engineered Targeting and Trafficking,” Experimental & Molecular Medicine 51 (2019): 32. 30872574 10.1038/s12276-019-0223-5 PMC6418170

[16] R. C. Lai , F. Arslan , M. M. Lee , et al., “Exosome Secreted by MSC Reduces Myocardial Ischemia/Reperfusion Injury,” Stem Cell Research 4, no. 3 (2010): 214–222. 20138817 10.1016/j.scr.2009.12.003

[17] M. J. Haney , N. L. Klyachko , Y. L. Zhao , et al., “Exosomes as Drug Delivery Vehicles for Parkinson's Disease Therapy,” Journal of Controlled Release 207 (2015): 18–30. 25836593 10.1016/j.jconrel.2015.03.033 PMC4430381

[18] Y. You , Y. Tian , Z. G. Yang , et al., “Intradermally Delivered mRNA‐encapsulating Extracellular Vesicles for Collagen‐replacement Therapy,” Nature Biomedical Engineering 7, no. 7 (2023): 887. 10.1038/s41551-022-00989-w 36635419

[19] A. I. Caplan and J. E. Dennis , “Mesenchymal Stem Cells as Trophic Mediators,” Journal of Cellular Biochemistry 98, no. 5 (2006): 1076–1084. 16619257 10.1002/jcb.20886

[20] P. M. P. Lins , E. Pirlet , M. Szymonik , A. Bronckaers , and I. Nelissen , “Manufacture of Extracellular Vesicles Derived From Mesenchymal Stromal Cells,” Trends in Biotechnology 41, no. 7 (2023): 965–981. 36750391 10.1016/j.tibtech.2023.01.003

[21] J. F. Burke , I. V. Yannas , W. C. Quinby Jr. , C. C. Bondoc , and W. K. Jung , “Successful Use of a Physiologically Acceptable Artificial Skin in the Treatment of Extensive Burn Injury,” Annals of Surgery 194, no. 4 (1981): 413–428. 6792993 10.1097/00000658-198110000-00005 PMC1345315

[22] R. Langer and J. P. Vacanti , “Tissue Engineering,” Science 260, no. 5110 (1993): 920–926. 8493529 10.1126/science.8493529

[23] A. Atala , S. B. Bauer , S. Soker , J. J. Yoo , and A. B. Retik , “Tissue‐engineered Autologous Bladders for Patients Needing Cystoplasty,” Lancet 367, no. 9518 (2006): 1241–1246. 16631879 10.1016/S0140-6736(06)68438-9

[24] M. Kim , Y. J. Kim , Y. S. Kim , et al., “One‐Year Results of Ear Reconstruction With 3D Printed Implants,” Yonsei Medical Journal 65, no. 8 (2024): 456–462. 39048321 10.3349/ymj.2023.0444 PMC11284305

[25] G. Prindull , B. Prindull , and N. Meulen , “Haematopoietic Stem Cells (CFUc) in human Cord Blood,” Acta Paediatrica Scandinavica 67, no. 4 (1978): 413–416. 676726 10.1111/j.1651-2227.1978.tb16347.x

[26] A. I. Caplan , “Mesenchymal Stem Cells,” Journal of Orthopaedic Research 9, no. 5 (1991): 641–650. 1870029 10.1002/jor.1100090504

[27] R. L. Rietze and B. A. Reynolds , “Neural Stem Cell Isolation and Characterization,” Methods in Enzymology 419 (2006): 3–23. 17141049 10.1016/S0076-6879(06)19001-1

[28] J. A. Thomson , J. Itskovitz‐Eldor , S. S. Shapiro , et al., “Embryonic Stem Cell Lines Derived From human Blastocysts,” Science 282, no. 5391 (1998): 1145–1147. 9804556 10.1126/science.282.5391.1145

[29] J. A. Snowden , I. Sanchez‐Ortega , S. Corbacioglu , et al., “Indications for Haematopoietic Cell Transplantation for Haematological Diseases, Solid Tumours and Immune Disorders: Current Practice in Europe, 2022,” Bone Marrow Transplantation 57, no. 8 (2022): 1217–1239. 35589997 10.1038/s41409-022-01691-w PMC9119216

[30] I. L. Weissman and J. A. Shizuru , “The Origins of the Identification and Isolation of Hematopoietic Stem Cells, and Their Capability to Induce Donor‐specific Transplantation Tolerance and Treat Autoimmune Diseases,” Blood 112, no. 9 (2008): 3543–3553. 18948588 10.1182/blood-2008-08-078220 PMC2574516

[31] S. Pinho and P. S. Frenette , “Haematopoietic Stem Cell Activity and Interactions With the Niche,” Nature Reviews Molecular Cell Biology 20, no. 5 (2019): 303–320. 30745579 10.1038/s41580-019-0103-9 PMC6483843

[32] N. Granot and R. Storb , “History of Hematopoietic Cell Transplantation: Challenges and Progress,” Haematologica 105, no. 12 (2020): 2716–2729. 33054108 10.3324/haematol.2019.245688 PMC7716373

[33] L. Li and P. K. Mandal , “Recent Advancements in Gene Therapy for Sickle Cell Disease and β‐Thalassemia,” Frontiers in Hematology 3 (2024).

[34] X. M. Anguela and K. A. High , “Hemophilia B and Gene Therapy: A New Chapter With Etranacogene dezaparvovec,” Blood Advances 8, no. 7 (2024): 1796–1803. 38592711 10.1182/bloodadvances.2023010511 PMC11006816

[35] D. Jovic , Y. Yu , D. Wang , et al., “A Brief Overview of Global Trends in MSC‐Based Cell Therapy,” Stem Cell Reviews and Reports 18, no. 5 (2022): 1525–1545. 35344199 10.1007/s12015-022-10369-1 PMC8958818

[36] O. Levy , R. Kuai , E. M. J. Siren , et al., “Shattering Barriers Toward Clinically Meaningful MSC Therapies,” Science Advances 6, no. 30 (2020): eaba6884. 32832666 10.1126/sciadv.aba6884 PMC7439491

[37] J. Isaković , K. Šerer , B. Barišić , and D. Mitrečić , “Mesenchymal Stem Cell Therapy for Neurological Disorders: The Light or the Dark Side of the Force?,” Frontiers in Bioengineering and Biotechnology 11 (2023): 1139359. 36926687 10.3389/fbioe.2023.1139359 PMC10011535

[38] W. Chen , L. Lv , N. Chen , and E. Cui , “Immunogenicity of Mesenchymal Stromal/Stem Cells,” Scandinavian Journal of Immunology 97, no. 6 (2023): e13267. 39007962 10.1111/sji.13267

[39] A. K. Berglund , L. A. Fortier , D. F. Antczak , and L. V. Schnabel , “Immunoprivileged no More: Measuring the Immunogenicity of Allogeneic Adult Mesenchymal Stem Cells,” Stem Cell Research & Therapy 8, no. 1 (2017): 288. 29273086 10.1186/s13287-017-0742-8 PMC5741939

[40] W. Z. Zhuang , Y. H. Lin , L. J. Su , et al., “Mesenchymal Stem/Stromal Cell‐based Therapy: Mechanism, Systemic Safety and Biodistribution for Precision Clinical Applications,” Journal of Biomedical Science 28, no. 1 (2021): 28. 33849537 10.1186/s12929-021-00725-7 PMC8043779

[41] D. Lu , X. Jiao , W. Jiang , et al., “Mesenchymal Stem Cells Influence Monocyte/Macrophage Phenotype: Regulatory Mode and Potential Clinical Applications,” Biomedicine & Pharmacotherapy 165 (2023): 115042. 37379639 10.1016/j.biopha.2023.115042

[42] N. Song , M. Scholtemeijer , and K. Shah , “Mesenchymal Stem Cell Immunomodulation: Mechanisms and Therapeutic Potential,” Trends in Pharmacological Sciences 41, no. 9 (2020): 653–664. 32709406 10.1016/j.tips.2020.06.009 PMC7751844

[43] L. Muller , A. Tunger , M. Wobus , et al., “Immunomodulatory Properties of Mesenchymal Stromal Cells: An Update,” Frontiers in Cell and Developmental Biology 9 (2021): 637725. 33634139 10.3389/fcell.2021.637725 PMC7900158

[44] N. Luque‐Campos , F. A. Bustamante‐Barrientos , C. Pradenas , et al., “The Macrophage Response Is Driven by Mesenchymal Stem Cell‐Mediated Metabolic Reprogramming,” Frontiers in Immunology 12 (2021): 624746. 34149687 10.3389/fimmu.2021.624746 PMC8213396

[45] H. Min , L. Xu , R. Parrott , et al., “Mesenchymal Stromal Cells Reprogram Monocytes and Macrophages With Processing Bodies,” Stem Cells 39, no. 1 (2021): 115–128. 33166420 10.1002/stem.3292

[46] T. S. Cheung , A. Galleu , M. von Bonin , M. Bornhauser , and F. Dazzi , “Apoptotic Mesenchymal Stromal Cells Induce Prostaglandin E2 in Monocytes: Implications for the Monitoring of Mesenchymal Stromal Cell Activity,” Haematologica 104, no. 10 (2019): e438–e441. 30846505 10.3324/haematol.2018.214767 PMC6886441

[47] A. Galleu , Y. Riffo‐Vasquez , C. Trento , et al., “Apoptosis in Mesenchymal Stromal Cells Induces in Vivo Recipient‐mediated Immunomodulation,” Science Translational Medicine 9, no. 416 (2017): eaam7828. 29141887 10.1126/scitranslmed.aam7828

[48] S. H. M. Pang , J. D'Rozario , S. Mendonca , et al., “Mesenchymal Stromal Cell Apoptosis Is Required for Their Therapeutic Function,” Nature Communications 12, no. 1 (2021): 6495. 10.1038/s41467-021-26834-3 PMC8586224 34764248

[49] F. Mohamad Yusoff and Y. Higashi , “Mesenchymal Stem/Stromal Cells for Therapeutic Angiogenesis,” Cells 12, no. 17 (2023): 2162. 37681894 10.3390/cells12172162 PMC10486439

[50] C. Huang , W. Luo , Q. Wang , et al., “Human Mesenchymal Stem Cells Promote Ischemic Repairment and Angiogenesis of Diabetic Foot Through Exosome miRNA‐21‐5p,” Stem Cell Research 52 (2021): 102235. 33601096 10.1016/j.scr.2021.102235

[51] Y. L. Tan , S. P. Eng , P. Hafez , N. Abdul Karim , J. X. Law , and M. H. Ng , “Mesenchymal Stromal Cell Mitochondrial Transfer as a Cell Rescue Strategy in Regenerative Medicine: A Review of Evidence in Preclinical Models,” Stem Cells Translational Medicine 11, no. 8 (2022): 814–827. 35851922 10.1093/stcltm/szac044 PMC9397650

[52] A. N. Mukkala , M. Jerkic , Z. Khan , K. Szaszi , A. Kapus , and O. Rotstein , “Therapeutic Effects of Mesenchymal Stromal Cells Require Mitochondrial Transfer and Quality Control,” International Journal of Molecular Sciences 24, no. 21 (2023): 15788. 37958771 10.3390/ijms242115788 PMC10647450

[53] Y. Cen , G. Lou , J. Qi , M. Zheng , and Y. Liu , “A New Perspective on Mesenchymal Stem Cell‐based Therapy for Liver Diseases: Restoring Mitochondrial Function,” Cell Communication and Signaling 21, no. 1 (2023): 214. 37596671 10.1186/s12964-023-01230-0 PMC10436412

[54] Medicine AfR. Cell Therapy Products.

[55] US FDA . RYONCIL, https://www.fda.gov/vaccines‐blood‐biologics/cellular‐gene‐therapy‐products/ryoncil.

[56] G. Liu , B. T. David , M. Trawczynski , and R. G. Fessler , “Advances in Pluripotent Stem Cells: History, Mechanisms, Technologies, and Applications,” Stem Cell Reviews and Reports 16, no. 1 (2020): 3–32. 31760627 10.1007/s12015-019-09935-x PMC6987053

[57] S. Yamanaka , “Pluripotent Stem Cell‐Based Cell Therapy‐Promise and Challenges,” Cell Stem Cell 27, no. 4 (2020): 523–531. 33007237 10.1016/j.stem.2020.09.014

[58] D. Liu , L. Bobrovskaya , and X.‐F. Zhou , “Cell Therapy for Neurological Disorders: The Perspective of Promising Cells,” Biology (Basel) 10, no. 11 (2021): 1142. 34827135 10.3390/biology10111142 PMC8614777

[59] R. Rahimi Darehbagh , S. A. Seyedoshohadaei , R. Ramezani , R. Ramezani , and N. Rezaei , “Stem Cell Therapies for Neurological Disorders: Current Progress, Challenges, and Future Perspectives,” European Journal of Medical Research 29, no. 1 (2024): 386. 39054501 10.1186/s40001-024-01987-1 PMC11270957

[60] N. Xie and B. Tang , “The Application of human iPSCs in Neurological Diseases: From Bench to Bedside,” Stem Cells International 2016, no. 1 (2016): 6484713. 26880979 10.1155/2016/6484713 PMC4736583

[61] J. Cerneckis , H. Cai , and Y. Shi , “Induced Pluripotent Stem Cells (iPSCs): Molecular Mechanisms of Induction and Applications,” Signal Transduction and Targeted Therapy 9, no. 1 (2024): 112. 38670977 10.1038/s41392-024-01809-0 PMC11053163

[62] L. Yang , S.‐C. Liu , Y.‐Y. Liu , et al., “Therapeutic Role of Neural Stem Cells in Neurological Diseases,” Frontiers in Bioengineering and Biotechnology 12 (2024): 1329712. 38515621 10.3389/fbioe.2024.1329712 PMC10955145

[63] M. Singh , P. K. Pandey , A. Bhasin , M. Padma , and S. Mohanty , “Application of Stem Cells in Stroke: A Multifactorial Approach,” Frontiers in Neuroscience 14 (2020): 473. 32581669 10.3389/fnins.2020.00473 PMC7296176

[64] W. Li , L. Shi , B. Hu , et al., “Mesenchymal Stem Cell‐based Therapy for Stroke: Current Understanding and Challenges,” Frontiers in Cellular Neuroscience 15 (2021): 628940. 33633544 10.3389/fncel.2021.628940 PMC7899984

[65] Y. Zhang , N. Dong , H. Hong , J. Qi , S. Zhang , and J. Wang , “Mesenchymal Stem Cells: Therapeutic Mechanisms for Stroke,” International Journal of Molecular Sciences 23, no. 5 (2022): 2550. 35269692 10.3390/ijms23052550 PMC8910569

[66] A. Andrzejewska , S. Dabrowska , B. Lukomska , and M. Janowski , “Mesenchymal Stem Cells for Neurological Disorders,” Advanced Science 8, no. 7 (2021): 2002944. 33854883 10.1002/advs.202002944 PMC8024997

[67] H. Huang , J. Zhang , J. Lin , and S. Shi , “Efficacy and Safety of Mesenchymal Stem Cells in Patients With Acute Ischemic Stroke: A Meta‐analysis,” BMC Neurology 24, no. 1 (2024): 48. 38287288 10.1186/s12883-024-03542-1 PMC10823675

[68] J. D. Sinden , C. Hicks , P. Stroemer , I. Vishnubhatla , and R. Corteling , “Human Neural Stem Cell Therapy for Chronic Ischemic Stroke: Charting Progress From Laboratory to Patients,” Stem Cells and Development 26, no. 13 (2017): 933–947. 28446071 10.1089/scd.2017.0009 PMC5510676

[69] G. Zhang , Y. Li , J. L. Reuss , et al., “Stable Intracerebral Transplantation of Neural Stem Cells for the Treatment of Paralysis due to Ischemic Stroke,” Stem Cells Translational Medicine 8, no. 10 (2019): 999–1007. 31241246 10.1002/sctm.18-0220 PMC6766600

[70] D. Kalladka , J. Sinden , K. Pollock , et al., “Human Neural Stem Cells in Patients With Chronic Ischaemic Stroke (PISCES): A Phase 1, First‐in‐man Study,” The Lancet 388, no. 10046 (2016): 787–796. 10.1016/S0140-6736(16)30513-X 27497862

[71] K. W. Muir , D. Bulters , M. Willmot , et al., “Intracerebral Implantation of human Neural Stem Cells and Motor Recovery After Stroke: Multicentre Prospective Single‐arm Study (PISCES‐2),” Journal of Neurology, Neurosurgery & Psychiatry 91, no. 4 (2020): 396–401. 32041820 10.1136/jnnp-2019-322515 PMC7147186

[72] Y. Xia , G. Hu , Y. Chen , et al., “Embryonic Stem Cell Derived Small Extracellular Vesicles Modulate Regulatory T Cells to Protect Against Ischemic Stroke,” ACS Nano 15, no. 4 (2021): 7370–7385. 33733738 10.1021/acsnano.1c00672

[73] A. A. Taei , S. Nasoohi , G. Hassanzadeh , M. Kadivar , L. Dargahi , and M. Farahmandfar , “Enhancement of Angiogenesis and Neurogenesis by Intracerebroventricular Injection of Secretome From human Embryonic Stem Cell‐derived Mesenchymal Stem Cells in Ischemic Stroke Model,” Biomedicine & Pharmacotherapy 140 (2021): 111709. 34020250 10.1016/j.biopha.2021.111709

[74] S. Yaqubi and M. Karimian , “Stem Cell Therapy as a Promising Approach for Ischemic Stroke Treatment,” Current Research in Pharmacology and Drug Discovery 6 (2024): 100183. 38831867 10.1016/j.crphar.2024.100183 PMC11144755

[75] G. Shroff , “Comparison of nutech Functional Score With European Stroke Scale for Patients With Cerebrovascular Accident Treated With human Embryonic Stem Cells: Nfs for cva Patients Treated With hescs,” Journal of Vascular and Interventional Neurology 9, no. 4 (2017): 35. 28702118 PMC5501127

[76] R. Duan , Y. Gao , R. He , et al., “Induced Pluripotent Stem Cells for Ischemic Stroke Treatment,” Frontiers in Neuroscience 15 (2021): 628663. 34135724 10.3389/fnins.2021.628663 PMC8202685

[77] K. Zhang , Y. Jiang , B. Wang , T. Li , D. Shang , and X. Zhang , “Mesenchymal Stem Cell Therapy: A Potential Treatment Targeting Pathological Manifestations of Traumatic Brain Injury,” Oxidative Medicine and Cellular Longevity 2022, no. 1 (2022): 4645021. 35757508 10.1155/2022/4645021 PMC9217616

[78] J. S. Andreassen , K. Thorsen , K. Søreide , D. Werner , and C. Weber , “Is There a Weekend Effect on Mortality Rate and Outcome for Moderate and Severe Traumatic Brain Injury? A Population‐based, Observational Cohort Study,” Brain and Spine 2 (2022): 101699. 36506297 10.1016/j.bas.2022.101699 PMC9729811

[79] S. H. Haddad and Y. M. Arabi , “Critical Care Management of Severe Traumatic Brain Injury in Adults,” Scandinavian Journal of Trauma, Resuscitation and Emergency Medicine 20 (2012): 1–15. 22304785 10.1186/1757-7241-20-12 PMC3298793

[80] Z.‐X. Zhang , L.‐X. Guan , K. Zhang , Q. Zhang , and L.‐J. Dai , “A Combined Procedure to Deliver Autologous Mesenchymal Stromal Cells to Patients With Traumatic Brain Injury,” Cytotherapy 10, no. 2 (2008): 134–139. 18368592 10.1080/14653240701883061

[81] C. Tian , X. Wang , X. Wang , et al., “Autologous Bone Marrow Mesenchymal Stem Cell Therapy in the Subacute Stage of Traumatic Brain Injury by Lumbar Puncture,” Experimental and Clinical Transplantation 11, no. 2 (2013): 176–181. 22891928 10.6002/ect.2012.0053

[82] M. Kawabori , A. H. Weintraub , H. Imai , et al., “Cell Therapy for Chronic TBI: Interim Analysis of the Randomized Controlled STEMTRA Trial,” Neurology 96, no. 8 (2021): e1202–e1214. 33397772 10.1212/WNL.0000000000011450 PMC8055341

[83] S. Wang , H. Cheng , G. Dai , et al., “Umbilical Cord Mesenchymal Stem Cell Transplantation Significantly Improves Neurological Function in Patients With Sequelae of Traumatic Brain Injury,” Brain Research 1532 (2013): 76–84. 23942181 10.1016/j.brainres.2013.08.001

[84] Z. Wang , Y. Luo , L. Chen , and W. Liang , “Safety of Neural Stem Cell Transplantation in Patients With Severe Traumatic Brain Injury,” Experimental and Therapeutic Medicine 13, no. 6 (2017): 3613–3618. 28588689 10.3892/etm.2017.4423 PMC5450816

[85] Y. Xia , J. Zhu , R. Yang , H. Wang , Y. Li , and C. Fu , “Mesenchymal Stem Cells in the Treatment of Spinal Cord Injury: Mechanisms, Current Advances and Future Challenges,” Frontiers in Immunology 14 (2023): 1141601. 36911700 10.3389/fimmu.2023.1141601 PMC9999104

[86] L. T. de Araújo , C. T. Macêdo , P. K. F. Damasceno , et al., “Clinical Trials Using Mesenchymal Stem Cells for Spinal Cord Injury: Challenges in Generating Evidence,” Cells 11, no. 6 (2022): 1019. 35326470 10.3390/cells11061019 PMC8946989

[87] S. Muthu , M. Jeyaraman , A. Gulati , and A. Arora , “Current Evidence on Mesenchymal Stem Cell Therapy for Traumatic Spinal Cord Injury: Systematic Review and Meta‐analysis,” Cytotherapy 23, no. 3 (2021): 186–197. 33183980 10.1016/j.jcyt.2020.09.007

[88] F. Tahmasebi and S. Barati , “Effects of Mesenchymal Stem Cell Transplantation on Spinal Cord Injury Patients,” Cell and Tissue Research 389, no. 3 (2022): 373–384. 35697943 10.1007/s00441-022-03648-3

[89] C. Gu , J. Feng , A. Waqas , et al., “Technological Advances of 3D Scaffold‐based Stem Cell/Exosome Therapy in Tissues and Organs,” Frontiers in Cell and Developmental Biology 9 (2021): 709204. 34568322 10.3389/fcell.2021.709204 PMC8458970

[90] G. Shroff , “Human Embryonic Stem Cell Therapy in Chronic Spinal Cord Injury: A Retrospective Study,” Clinical and Translational Science 9, no. 3 (2016): 168–175. 27144379 10.1111/cts.12394 PMC5351327

[91] G. Shroff and R. Gupta , “Human Embryonic Stem Cells in the Treatment of Patients With Spinal Cord Injury,” Annals of Neurosciences 22, no. 4 (2015): 208. 26526627 10.5214/ans.0972.7531.220404 PMC4627203

[92] S. McIntyre , S. Goldsmith , A. Webb , et al., “Global Prevalence of Cerebral Palsy: A Systematic Analysis,” Developmental Medicine & Child Neurology 64, no. 12 (2022): 1494–1506. 35952356 10.1111/dmcn.15346 PMC9804547

[93] I. Novak , C. Morgan , M. Fahey , et al., “State of the Evidence Traffic Lights 2019: Systematic Review of Interventions for Preventing and Treating Children With Cerebral Palsy,” Current Neurology and Neuroscience Reports 20 (2020): 1–21. 32086598 10.1007/s11910-020-1022-z PMC7035308

[94] B. Xie , M. Chen , R. Hu , W. Han , and S. Ding , “Therapeutic Evidence of Human Mesenchymal Stem Cell Transplantation for Cerebral Palsy: A Meta‐Analysis of Randomized Controlled Trials,” Stem Cells International 2020, no. 1 (2020): 5701920. 32765613 10.1155/2020/5701920 PMC7387980

[95] Z.‐Y. Lv , Y. Li , and J. Liu , “Progress in Clinical Trials of Stem Cell Therapy for Cerebral Palsy,” Neural Regeneration Research 16, no. 7 (2021): 1377–1382. 33318421 10.4103/1673-5374.300979 PMC8284300

[96] G. Shroff , A. Gupta , and J. K. Barthakur , “Therapeutic Potential of human Embryonic Stem Cell Transplantation in Patients With Cerebral Palsy,” Journal of Translational Medicine 12 (2014): 1–9. 25496119 10.1186/s12967-014-0318-7 PMC4297392

[97] A. Bhatt , H. Bhardwaj , and P. Shrivastava , “Mesenchymal Stem Cell Therapy for Alzheimer's Disease: A Novel Therapeutic Approach for Neurodegenerative Diseases,” Neuroscience 555 (2024): 52–68. 39032806 10.1016/j.neuroscience.2024.07.019

[98] S. Regmi , D. D. Liu , M. Shen , et al., “Mesenchymal Stromal Cells for the Treatment of Alzheimer's Disease: Strategies and Limitations,” Frontiers in Molecular Neuroscience 15 (2022): 1011225. 36277497 10.3389/fnmol.2022.1011225 PMC9584646

[99] H. J. Kim , S. W. Seo , J. W. Chang , et al., “Stereotactic Brain Injection of human Umbilical Cord Blood Mesenchymal Stem Cells in Patients With Alzheimer's Disease Dementia: A Phase 1 Clinical Trial,” Alzheimer's & Dementia: Translational Research & Clinical Interventions 1, no. 2 (2015): 95–102. 29854930 10.1016/j.trci.2015.06.007 PMC5975048

[100] H. J. Kim , K. R. Cho , H. Jang , et al., “Intracerebroventricular Injection of human Umbilical Cord Blood Mesenchymal Stem Cells in Patients With Alzheimer's disease Dementia: A Phase I Clinical Trial,” Alzheimer's Research & Therapy 13 (2021): 1–11. 10.1186/s13195-021-00897-2 PMC8439008 34521461

[101] C. Duma , O. Kopyov , A. Kopyov , et al., “Human Intracerebroventricular (ICV) Injection of Autologous, Non‐engineered, Adipose‐derived Stromal Vascular Fraction (ADSVF) for Neurodegenerative Disorders: Results of a 3‐year Phase 1 Study of 113 Injections in 31 Patients,” Molecular Biology Reports 46, no. 5 (2019): 5257–5272. 31327120 10.1007/s11033-019-04983-5

[102] M. T. Valenti , M. Serena , L. Dalle Carbonare , and D. Zipeto , “CRISPR/Cas System: An Emerging Technology in Stem Cell Research,” World Journal of Stem Cells 11, no. 11 (2019): 937. 31768221 10.4252/wjsc.v11.i11.937 PMC6851009

[103] K. S. Chen , M. H. Noureldein , L. M. McGinley , et al., “Human Neural Stem Cells Restore Spatial Memory in a Transgenic Alzheimer's disease Mouse Model by an Immunomodulating Mechanism,” Frontiers in Aging Neuroscience 15 (2023): 1306004. 38155736 10.3389/fnagi.2023.1306004 PMC10753006

[104] M. H. Tuszynski , L. Thal , M. Pay , et al., “A Phase 1 Clinical Trial of Nerve Growth Factor Gene Therapy for Alzheimer Disease,” Nature Medicine 11, no. 5 (2005): 551–555. 10.1038/nm1239 15852017

[105] A. Ortega , B. Chernicki , G. Ou , and M. S. Parmar , “From Lab Bench to Hope: Emerging Gene Therapies in Clinical Trials for Alzheimer's Disease,” Molecular Neurobiology (2024): 1–24. 10.1007/s12035-024-04285-3 38958888

[106] P. Tambe , V. Undale , A. Sanap , R. Bhonde , and N. Mante , “The Prospective Role of Mesenchymal Stem Cells in Parkinson's Disease,” Parkinsonism & Related Disorders 127 (2024): 107087. 39142905 10.1016/j.parkreldis.2024.107087

[107] R. M. Heris , M. Shirvaliloo , S. Abbaspour‐Aghdam , et al., “The Potential Use of Mesenchymal Stem Cells and Their Exosomes in Parkinson's disease Treatment,” Stem Cell Research & Therapy 13, no. 1 (2022): 371. 35902981 10.1186/s13287-022-03050-4 PMC9331055

[108] A. Unnisa , K. Dua , and M. A. Kamal , “Mechanism of Mesenchymal Stem Cells as a Multitarget Disease‐Modifying Therapy for Parkinson's Disease,” Current Neuropharmacology 21, no. 4 (2023): 988. 35339180 10.2174/1570159X20666220327212414 PMC10227913

[109] N. K. Venkataramana , S. K. Kumar , S. Balaraju , et al., “Open‐labeled Study of Unilateral Autologous Bone‐marrow‐derived Mesenchymal Stem Cell Transplantation in Parkinson's Disease,” Translational Research 155, no. 2 (2010): 62–70. 20129486 10.1016/j.trsl.2009.07.006

[110] M. Schiess , J. Suescun , M. F. Doursout , et al., “Allogeneic Bone Marrow–derived Mesenchymal Stem Cell Safety in Idiopathic Parkinson's Disease,” Movement Disorders 36, no. 8 (2021): 1825–1834. 33772873 10.1002/mds.28582 PMC8451899

[111] Y. Qiu , Z. Wang , and H.‐S. Lu , “Umbilical Cord Mesenchymal Stem Cell Transplantation for Treatment of Parkinson's Disease in 8 Cases,” Chinese Journal of Tissue Engineering Research 15, no. 36 (2011): 6833.

[112] Y. Wang , X.‐L. Zhao , J.‐Y. Zhang , and J. Tan , “Therapeutic Applications of Umbilical Cord Mesenchymal Stem Cells in Parkinson's Disease,” Chinese Journal of Tissue Engineering Research 18, no. 6 (2014): 932.

[113] K. Shigematsu , N. Komori , K. Tahara , and H. Yamagishi , “Repeated Infusion of Autologous Adipose Tissue‐derived Stem Cells for Parkinson's Disease,” Acta Neurologica Scandinavica 145, no. 1 (2022): 119–122. 34716582 10.1111/ane.13547

[114] J. Lee , D. Bayarsaikhan , R. Arivazhagan , et al., “CRISPR/Cas9 Edited sRAGE‐MSCs Protect Neuronal Death in Parkinson's Disease Model,” International Journal of Stem Cells 12, no. 1 (2019): 114–124. 30836725 10.15283/ijsc18110 PMC6457706

[115] L. Leng and T. Zengmin , “Transplantation of Neural Precursor Cells in the Treatment of Parkinson Disease: An Efficacy and Safety Analysis,” Turkish Neurosurgery 26, no. 3 (2016): 378–383. 27161464 10.5137/1019-5149.JTN.10747-14.4

[116] I. Madrazo , O. Kopyov , M. Ávila‐Rodríguez , et al., “Transplantation of human Neural Progenitor Cells (NPC) Into Putamina of parkinsonian Patients: A Case Series Study, Safety and Efficacy Four Years After Surgery,” Cell Transplantation 28, no. 3 (2019): 269–285. 30574805 10.1177/0963689718820271 PMC6425108

[117] J. S. Schweitzer , B. Song , T. M. Herrington , et al., “Personalized iPSC‐derived Dopamine Progenitor Cells for Parkinson's Disease,” New England Journal of Medicine 382, no. 20 (2020): 1926–1932. 32402162 10.1056/NEJMoa1915872 PMC7288982

[118] WHO . Autism Spectrum Disorder. September 18, 2024. Accessed September 18, 2024. https://www.who.int/news‐room/fact‐sheets/detail/autism‐spectrum‐disorders.

[119] A. Akat and E. Karaöz , “Cell Therapies for Autism Spectrum Disorder: A Systematic Review of Clinical Applications,” Middle East Current Psychiatry 30, no. 1 (2023): 94.

[120] R. Tamouza , F. Volt , J.‐R. Richard , et al., “Possible Effect of the Use of Mesenchymal Stromal Cells in the Treatment of Autism Spectrum Disorders: A Review,” Frontiers in Cell and Developmental Biology 10 (2022): 809686. 35865626 10.3389/fcell.2022.809686 PMC9294632

[121] B. Gesundheit , P. Ashwood , A. Keating , D. Naor , M. Melamed , and J. P. Rosenzweig , “Therapeutic Properties of Mesenchymal Stem Cells for Autism Spectrum Disorders,” Medical Hypotheses 84, no. 3 (2015): 169–177. 25592283 10.1016/j.mehy.2014.12.016

[122] Q. Liu , M.‐X. Chen , L. Sun , et al., “Rational Use of Mesenchymal Stem Cells in the Treatment of Autism Spectrum Disorders,” World Journal of Stem Cells 11, no. 2 (2019): 55. 30842805 10.4252/wjsc.v11.i2.55 PMC6397804

[123] S. Y. Lee , H. Ahn , W.‐J. Jung , J. Ahn , and K. H. Lee , “A Case Study on Autism Spectrum Disorder Treatment Using Allogenic Mesenchymal Stem Cells Derived From the Human Umbilical Cord,” Case Reports: Open Access 5 (2020): 1–7.

[124] J. M. Sun , G. Dawson , L. Franz , et al., “Infusion of human Umbilical Cord Tissue Mesenchymal Stromal Cells in Children With autism Spectrum Disorder,” Stem Cells Translational Medicine 9, no. 10 (2020): 1137–1146. 32531111 10.1002/sctm.19-0434 PMC7519773

[125] N. Sharifzadeh , A. Ghasemi , J. Tavakol Afshari , et al., “Intrathecal Autologous Bone Marrow Stem Cell Therapy in Children With Autism: A Randomized Controlled Trial,” Asia‐Pacific Psychiatry 13, no. 2 (2021): e12445. 33150703 10.1111/appy.12445

[126] E. Deneault , S. H. White , D. C. Rodrigues , et al., “Complete Disruption of Autism‐susceptibility Genes by Gene Editing Predominantly Reduces Functional Connectivity of Isogenic human Neurons,” Stem Cell Reports 11, no. 5 (2018): 1211–1225. 30392976 10.1016/j.stemcr.2018.10.003 PMC6235011

[127] S. Najafi , P. Najafi , N. K. Farkhad , et al., “Mesenchymal Stem Cell Therapy in Amyotrophic Lateral Sclerosis (ALS) Patients: A Comprehensive Review of Disease Information and Future Perspectives,” Iranian Journal of Basic Medical Sciences 26, no. 8 (2023): 872. 37427325 10.22038/IJBMS.2023.66364.14572 PMC10329242

[128] S. H. Kim , K.‐W. Oh , M.‐Y. Noh , and M.‐S. Kwon , “Optimal Therapeutic Strategy of Bone Marrow‐Originated Autologous Mesenchymal Stromal/Stem Cells for ALS,” Stem Cells Translational Medicine 13, no. 4 (2024): 309–316. 38244235 10.1093/stcltm/szad095 PMC11016834

[129] L. Mazzini , F. Fagioli , R. Boccaletti , et al., “Stem Cell Therapy in Amyotrophic Lateral Sclerosis: A Methodological Approach in Humans,” Amyotrophic Lateral Sclerosis and Other Motor Neuron Disorders 4, no. 3 (2003): 158–161. 13129802 10.1080/14660820310014653

[130] J. D. Glass , V. S. Hertzberg , N. M. Boulis , et al., “Transplantation of Spinal Cord–derived Neural Stem Cells for ALS: Analysis of Phase 1 and 2 Trials,” Neurology 87, no. 4 (2016): 392–400. 27358335 10.1212/WNL.0000000000002889 PMC4977116

[131] E. L. Feldman , N. M. Boulis , J. Hur , et al., “Intraspinal Neural Stem Cell Transplantation in amyotrophic Lateral sclerosis: Phase 1 Trial Outcomes,” Annals of Neurology 75, no. 3 (2014): 363–373. 24510776 10.1002/ana.24113 PMC4005820

[132] L. Mazzini , M. Gelati , D. C. Profico , et al., “Human Neural Stem Cell Transplantation in ALS: Initial Results From a Phase I Trial,” Journal of Translational Medicine 13 (2015): 1–16. 25889343 10.1186/s12967-014-0371-2 PMC4359401

[133] R. H. Baloh , J. P. Johnson , P. Avalos , et al., “Transplantation of human Neural Progenitor Cells Secreting GDNF Into the Spinal Cord of Patients With ALS: A Phase 1/2a Trial,” Nature Medicine 28, no. 9 (2022): 1813–1822. 10.1038/s41591-022-01956-3 PMC9499868 36064599

[134] T.‐T. Li , Z.‐R. Wang , W.‐Q. Yao , E.‐Q. Linghu , F.‐S. Wang , and L. Shi , “Stem Cell Therapies for Chronic Liver Diseases: Progress and Challenges,” Stem Cells Translational Medicine 11, no. 9 (2022): 900–911. 35993521 10.1093/stcltm/szac053 PMC9492280

[135] W. Lu , J. Qu , L. Yan , et al., “Efficacy and Safety of Mesenchymal Stem Cell Therapy in Liver Cirrhosis: A Systematic Review and Meta‐analysis,” Stem Cell Research & Therapy 14, no. 1 (2023): 301. 37864199 10.1186/s13287-023-03518-x PMC10590028

[136] Clinicaltrials.gove . Clinical trials using MSCs for liver disease. September 09, 2024. Accessed September 29, 2024. https://clinicaltrials.gov/search?cond=liver%20disease&intr=mesenchymal%20stem%20cell%20OR%20mesenchymal%20stromal%20cell.

[137] Y. Nagamoto , K. Takayama , K. Ohashi , et al., “Transplantation of a human iPSC‐derived Hepatocyte Sheet Increases Survival in Mice With Acute Liver Failure,” Journal of Hepatology 64, no. 5 (2016): 1068–1075. 26778754 10.1016/j.jhep.2016.01.004

[138] A. Laemmle , M. Poms , B. Hsu , et al., “Aquaporin 9 Induction in human i PSC‐derived Hepatocytes Facilitates Modeling of Ornithine Transcarbamylase Deficiency,” Hepatology 76, no. 3 (2022): 646–659. 34786702 10.1002/hep.32247 PMC9295321

[139] M. J. Hossain , M. Al‐Mamun , and M. R. Islam , “Diabetes Mellitus, the Fastest Growing Global Public Health Concern: Early Detection Should be Focused,” Health Science Reports 7, no. 3 (2024): e2004. 38524769 10.1002/hsr2.2004 PMC10958528

[140] M. A. Ghoneim , M. M. Gabr , S. M. El‐Halawani , and A. F. Refaie , “Current Status of Stem Cell Therapy for Type 1 Diabetes: A Critique and a Prospective Consideration,” Stem Cell Research & Therapy 15, no. 1 (2024): 23. 38281991 10.1186/s13287-024-03636-0 PMC10823744

[141] J. Hu , X. Yu , Z. Wang , et al., “Long Term Effects of the Implantation of Wharton's Jelly‐derived Mesenchymal Stem Cells From the Umbilical Cord for Newly‐onset Type 1 Diabetes Mellitus,” Endocrine Journal 60, no. 3 (2013): 347–357. 23154532 10.1507/endocrj.ej12-0343

[142] P.‐O. Carlsson , E. Schwarcz , O. Korsgren , and K. Le Blanc , “Preserved β‐cell Function in Type 1 Diabetes by Mesenchymal Stromal Cells,” Diabetes 64, no. 2 (2015): 587–592. 25204974 10.2337/db14-0656

[143] D. B. Araujo , J. R. Dantas , K. R. Silva , et al., “Allogenic Adipose Tissue‐derived Stromal/Stem Cells and Vitamin D Supplementation in Patients With Recent‐onset Type 1 Diabetes Mellitus: A 3‐month Follow‐up Pilot Study,” Frontiers in Immunology 11 (2020): 993. 32582156 10.3389/fimmu.2020.00993 PMC7280537

[144] M. Izadi , A. Sadr Hashemi Nejad , M. Moazenchi , et al., “Mesenchymal Stem Cell Transplantation in Newly Diagnosed Type‐1 Diabetes Patients: A Phase I/II Randomized Placebo‐controlled Clinical Trial,” Stem Cell Research & Therapy 13, no. 1 (2022): 264. 35725652 10.1186/s13287-022-02941-w PMC9208234

[145] J. He , D. Kong , Z. Yang , et al., “Clinical Efficacy on Glycemic Control and Safety of Mesenchymal Stem Cells in Patients With Diabetes Mellitus: Systematic Review and Meta‐analysis of RCT Data,” PLoS One 16, no. 3 (2021): e0247662. 33705413 10.1371/journal.pone.0247662 PMC7951834

[146] T. C. Schulz , “Concise Review: Manufacturing of Pancreatic Endoderm Cells for Clinical Trials in Type 1 Diabetes,” Stem Cells Translational Medicine 4, no. 8 (2015): 927–931. 26062982 10.5966/sctm.2015-0058 PMC4511151

[147] R. R. Henry , J. Pettus , J. Wilensky , et al., “Initial Clinical Evaluation of VC‐01TM Combination Product—a Stem Cell–derived Islet Replacement for Type 1 Diabetes (T1D),” Diabetes 67, no. Supplement_1 (2018): 138–OR.

[148] A. J. Shapiro , D. Thompson , T. W. Donner , et al., “Insulin Expression and C‐peptide in Type 1 Diabetes Subjects Implanted With Stem Cell‐derived Pancreatic Endoderm Cells in an Encapsulation Device,” Cell Reports Medicine 2, no. 12 (2021): 100466. 35028608 10.1016/j.xcrm.2021.100466 PMC8714853

[149] J. A. Bluestone , J. H. Buckner , M. Fitch , et al., “Type 1 Diabetes Immunotherapy Using Polyclonal Regulatory T Cells,” Science Translational Medicine 7, no. 315 (2015): 315ra189–315ra189. 10.1126/scitranslmed.aad4134 PMC4729454 26606968

[150] S. Gao , Y. Zhang , K. Liang , R. Bi , and Y. Du , “Mesenchymal Stem Cells (MSCs): A Novel Therapy for Type 2 Diabetes,” Stem Cells International 2022, no. 1 (2022): 8637493. 36045953 10.1155/2022/8637493 PMC9424025

[151] K.‐S. Park , Y.‐S. Kim , J.‐H. Kim , et al., “Trophic Molecules Derived From human Mesenchymal Stem Cells Enhance Survival, Function, and Angiogenesis of Isolated Islets After Transplantation,” Transplantation 89, no. 5 (2010): 509–517. 20125064 10.1097/TP.0b013e3181c7dc99

[152] M. Shrestha , T. T. Nguyen , J. Park , et al., “Immunomodulation Effect of Mesenchymal Stem Cells in Islet Transplantation,” Biomedicine & Pharmacotherapy 142 (2021): 112042. 34403963 10.1016/j.biopha.2021.112042

[153] D. J. Borg , M. Weigelt , C. Wilhelm , et al., “Mesenchymal Stromal Cells Improve Transplanted Islet Survival and Islet Function in a syngeneic Mouse Model,” Diabetologia 57 (2014): 522–531. 24253203 10.1007/s00125-013-3109-4

[154] K. Zhao , H. Hao , J. Liu , et al., “Bone Marrow‐derived Mesenchymal Stem Cells Ameliorate Chronic High Glucose‐induced β‐cell Injury Through Modulation of Autophagy,” Cell Death & Disease 6, no. 9 (2015): e1885–e1885. 26379190 10.1038/cddis.2015.230 PMC4650435

[155] Y. Yuan , L. Yuan , L. Li , et al., “Mitochondrial Transfer From Mesenchymal Stem Cells to Macrophages Restricts Inflammation and Alleviates Kidney Injury in Diabetic Nephropathy Mice via PGC‐1α Activation,” Stem Cells 39, no. 7 (2021): 913–928. 33739541 10.1002/stem.3375

[156] N. Konari , K. Nagaishi , S. Kikuchi , and M. Fujimiya , “Mitochondria Transfer From Mesenchymal Stem Cells Structurally and Functionally Repairs Renal Proximal Tubular Epithelial Cells in Diabetic Nephropathy in Vivo,” Scientific Reports 9, no. 1 (2019): 5184. 30914727 10.1038/s41598-019-40163-y PMC6435708

[157] C. L. Rackham , E. L. Hubber , A. Czajka , A. N. Malik , A. J. King , and P. M. Jones , “Optimizing Beta Cell Function Through Mesenchymal Stromal Cell‐mediated Mitochondria Transfer,” Stem Cells 38, no. 4 (2020): 574–584. 31912945 10.1002/stem.3134 PMC7187381

[158] Clinicaltrials.gov . October 1st, 2024. Accessed October 1st, 2024. https://clinicaltrials.gov/search?cond=Diabetes%20Type%202&intr=mesenchymal%20stem%20cell%20OR%20mesenchymal%20stromal%20cell&page=1.

[159] U. E. Habiba , N. Khan , D. L. Greene , K. Ahmad , S. Shamim , and A. Umer , “Meta‐analysis Shows That Mesenchymal Stem Cell Therapy Can be a Possible Treatment for Diabetes,” Frontiers in Endocrinology (Lausanne) 15 (2024): 1380443. 10.3389/fendo.2024.1380443 PMC11116613 38800472

[160] Y. Li , F. Wang , H. Liang , et al., “Efficacy of Mesenchymal Stem Cell Transplantation Therapy for Type 1 and Type 2 Diabetes Mellitus: A Meta‐analysis,” Stem Cell Research & Therapy 12, no. 1 (2021): 273. 33957998 10.1186/s13287-021-02342-5 PMC8101194

[161] J. Wu , T. Li , M. Guo , et al., “Treating a Type 2 Diabetic Patient With Impaired Pancreatic Islet Function by Personalized Endoderm Stem Cell‐derived Islet Tissue,” Cell Discovery 10, no. 1 (2024): 45. 38684699 10.1038/s41421-024-00662-3 PMC11058776

[162] D. Balboa , J. Saarimäki‐Vire , D. Borshagovski , et al., “Insulin Mutations Impair Beta‐cell Development in a Patient‐derived iPSC Model of Neonatal Diabetes,” Elife 7 (2018): e38519. 30412052 10.7554/eLife.38519 PMC6294552

[163] S. Safiri , K. Carson‐Chahhoud , M. Noori , et al., “Burden of Chronic Obstructive Pulmonary Disease and Its Attributable Risk Factors in 204 Countries and territories, 1990–2019: Results From the Global Burden of Disease Study 2019,” Bmj 378 (2022): e069679. 35896191 10.1136/bmj-2021-069679 PMC9326843

[164] Z. N. Wang and X. X. Tang , “New Perspectives on the Aberrant Alveolar Repair of Idiopathic Pulmonary Fibrosis,” Frontiers in Cell and Developmental Biology 8 (2020): 580026. 33117807 10.3389/fcell.2020.580026 PMC7561442

[165] A. Daher and M. Dreher , “Supplemental Oxygen Therapy in Chronic Obstructive Pulmonary Disease: Is Less Is More? How Much Is Too Much?,” Current Opinion in Pulmonary Medicine 30, no. 2 (2024): 179–184. 37882582 10.1097/MCP.0000000000001025

[166] F. M. Siddiqui and J. M. Diamond , “Lung Transplantation for Chronic Obstructive Pulmonary Disease: Past, Present, and Future Directions,” Current Opinion in Pulmonary Medicine 24, no. 2 (2018): 199–204. 29227305 10.1097/MCP.0000000000000452 PMC5839672

[167] M. A. Matthay , R. L. Zemans , G. A. Zimmerman , et al., “Acute respiratory Distress Syndrome,” Nature Reviews Disease Primers 5, no. 1 (2019): 18. 10.1038/s41572-019-0069-0 PMC6709677 30872586

[168] J. C. Grotberg , D. Reynolds , and B. D. Kraft , “Management of Severe Acute respiratory Distress Syndrome: A Primer,” Critical Care 27, no. 1 (2023): 289. 37464381 10.1186/s13054-023-04572-w PMC10353255

[169] D. Murugan and L. Rangasamy , “Pooled Evidence From Preclinical and Clinical Studies for Stem Cell‐based Therapy in ARDS and COVID‐19,” Molecular and Cellular Biochemistry 478, no. 7 (2023): 1487–1518. 36394787 10.1007/s11010-022-04601-2 PMC9672621

[170] L. Chow , V. Johnson , R. Impastato , J. Coy , A. Strumpf , and S. Dow , “Antibacterial Activity of human Mesenchymal Stem Cells Mediated Directly by Constitutively Secreted Factors and Indirectly by Activation of Innate Immune Effector Cells,” Stem Cells Translational Medicine 9, no. 2 (2020): 235–249. 31702119 10.1002/sctm.19-0092 PMC6988770

[171] O. E. Simonson , D. Mougiakakos , N. Heldring , et al., “In Vivo Effects of Mesenchymal Stromal Cells in Two Patients With Severe Acute respiratory Distress Syndrome,” Stem Cells Translational Medicine 4, no. 10 (2015): 1199–1213. 26285659 10.5966/sctm.2015-0021 PMC4572899

[172] J. G. Wilson , K. D. Liu , H. Zhuo , et al., “Mesenchymal Stem (stromal) Cells for Treatment of ARDS: A Phase 1 Clinical Trial,” The Lancet Respiratory Medicine 3, no. 1 (2015): 24–32. 25529339 10.1016/S2213-2600(14)70291-7 PMC4297579

[173] M. A. Matthay , C. S. Calfee , H. Zhuo , et al., “Treatment With Allogeneic Mesenchymal Stromal Cells for Moderate to Severe Acute respiratory Distress Syndrome (START study): A Randomised Phase 2a Safety Trial,” The Lancet Respiratory Medicine 7, no. 2 (2019): 154–162. 30455077 10.1016/S2213-2600(18)30418-1 PMC7597675

[174] K. Ichikado , T. Kotani , Y. Kondoh , et al., “Clinical Efficacy and Safety of Multipotent Adult Progenitor Cells (invimestrocel) for Acute respiratory Distress Syndrome (ARDS) Caused by Pneumonia: A Randomized, Open‐label, Standard Therapy–controlled, Phase 2 Multicenter Study (ONE‐BRIDGE),” Stem Cell Research & Therapy 14, no. 1 (2023): 217. 37608287 10.1186/s13287-023-03451-z PMC10464414

[175] B. Thébaud , K. N. Goss , M. Laughon , et al., “Bronchopulmonary Dysplasia,” Nature Reviews Disease Primers 5, no. 1 (2019): 78. 10.1038/s41572-019-0127-7 PMC6986462 31727986

[176] A. R. Schmidt and C. Ramamoorthy , “Bronchopulmonary Dysplasia,” Pediatric Anesthesia 32, no. 2 (2022): 174–180. 34877749 10.1111/pan.14365

[177] Y. S. Chang , S. Y. Ahn , H. S. Yoo , et al., “Mesenchymal Stem Cells for Bronchopulmonary Dysplasia: Phase 1 Dose‐escalation Clinical Trial,” The Journal of Pediatrics 164, no. 5 (2014): 966–972. e6. 24508444 10.1016/j.jpeds.2013.12.011

[178] N. T. Liem , T. L. Anh , T. T. H. Thai , and B. V. Anh , “Bone Marrow Mononuclear Cells Transplantation in Treatment of Established Bronchopulmonary Dysplasia: A Case Report,” The American Journal of Case Reports 18 (2017): 1090. 29021519 10.12659/AJCR.905244 PMC5652889

[179] S. Y. Ahn , Y. S. Chang , J. H. Kim , S. I. Sung , and W. S. Park , “Two‐year Follow‐up Outcomes of Premature Infants Enrolled in the Phase I Trial of Mesenchymal Stem Cells Transplantation for Bronchopulmonary Dysplasia,” The Journal of Pediatrics 185 (2017): 49–54. e2. 28341525 10.1016/j.jpeds.2017.02.061

[180] S. Y. Ahn , Y. S. Chang , M. H. Lee , et al., “Stem Cells for Bronchopulmonary Dysplasia in Preterm Infants: A Randomized Controlled Phase II Trial,” Stem Cells Translational Medicine 10, no. 8 (2021): 1129–1137. 33876883 10.1002/sctm.20-0330 PMC8284779

[181] M. J. del Cerro Marín , I. G. Ormazábal , A. Gimeno‐Navarro , et al., “Repeated Intravenous Doses of human Umbilical Cord‐derived Mesenchymal Stromal Cells for Bronchopulmonary Dysplasia: Results of a Phase 1 Clinical Trial With 2‐year Follow‐up,” Cytotherapy 26, no. 6 (2024): 632–640. 38556960 10.1016/j.jcyt.2024.02.028

[182] A. Sharma , I. Ahmad Farouk , and S. K. Lal , “COVID‐19: A Review on the Novel Coronavirus Disease Evolution, Transmission, Detection, Control and Prevention,” Viruses. 13, no. 2 (2021): 202. 33572857 10.3390/v13020202 PMC7911532

[183] E. Arabpour , S. Khoshdel , N. Tabatabaie , A. Akhgarzad , M. Zangiabadian , and M. J. Nasiri , “Stem Cells Therapy for COVID‐19: A Systematic Review and Meta‐analysis,” Frontiers in Medicine 8 (2021): 737590. 34912818 10.3389/fmed.2021.737590 PMC8666565

[184] Y. Feng , J. Huang , J. Wu , et al., “Safety and Feasibility of Umbilical Cord Mesenchymal Stem Cells in Patients With COVID‐19 Pneumonia: A Pilot Study,” Cell Proliferation 53, no. 12 (2020): e12947. 33205469 10.1111/cpr.12947 PMC7705911

[185] F. Meng , R. Xu , S. Wang , et al., “Human Umbilical Cord‐derived Mesenchymal Stem Cell Therapy in Patients With COVID‐19: A Phase 1 Clinical Trial,” Signal Transduction and Targeted Therapy 5, no. 1 (2020): 172. 32855385 10.1038/s41392-020-00286-5 PMC7450163

[186] L. Shu , C. Niu , R. Li , et al., “Treatment of Severe COVID‐19 With human Umbilical Cord Mesenchymal Stem Cells,” Stem Cell Research & Therapy 11 (2020): 1–11. 32811531 10.1186/s13287-020-01875-5 PMC7432540

[187] H. Y. Li , T. Y. Gao , W. Fang , et al., “Global, Regional and National Burden of Chronic Obstructive Pulmonary Disease Over a 30‐year Period: Estimates From the 1990 to 2019 Global Burden of Disease Study,” Respirology (Carlton, Vic.) 28, no. 1 (2023): 29–36. 36054068 10.1111/resp.14349 PMC10087739

[188] M. Hikichi , K. Mizumura , S. Maruoka , and Y. Gon , “Pathogenesis of Chronic Obstructive Pulmonary Disease (COPD) Induced by Cigarette Smoke,” Journal of Thoracic Disease 11, no. Suppl 17 (2019): S2129. 31737341 10.21037/jtd.2019.10.43 PMC6831915

[189] W. Broekman , P. P. Khedoe , K. Schepers , H. Roelofs , J. Stolk , and P. S. Hiemstra , “Mesenchymal Stromal Cells: A Novel Therapy for the Treatment of Chronic Obstructive Pulmonary Disease?,” Thorax 73, no. 6 (2018): 565–574. 29653970 10.1136/thoraxjnl-2017-210672 PMC5969341

[190] X. Liu , Q. Fang , and H. Kim , “Preclinical Studies of Mesenchymal Stem Cell (MSC) Administration in Chronic Obstructive Pulmonary Disease (COPD): A Systematic Review and Meta‐analysis,” PLoS One 11, no. 6 (2016): e0157099. 27280283 10.1371/journal.pone.0157099 PMC4900582

[191] H. G. de Oliveira , F. F. Cruz , M. A. Antunes , et al., “Combined Bone Marrow‐derived Mesenchymal Stromal Cell Therapy and One‐way Endobronchial Valve Placement in Patients With Pulmonary Emphysema: A Phase I Clinical Trial,” Stem Cells Translational Medicine 6, no. 3 (2017): 962–969. 28186686 10.1002/sctm.16-0315 PMC5442791

[192] E. C. van Doorn , J. H. Amesz , A. H. Sadeghi , N. M. de Groot , O. C. Manintveld , and Y. J. Taverne , “Preclinical Models of Cardiac Disease: A Comprehensive Overview for Clinical Scientists,” Cardiovascular Engineering and Technology 15, no. 2 (2024): 232–249. 38228811 10.1007/s13239-023-00707-w PMC11116217

[193] S. Funakoshi and Y. Yoshida , “Recent Progress of iPSC Technology in Cardiac Diseases,” Archives of Toxicology 95, no. 12 (2021): 3633–3650. 34657219 10.1007/s00204-021-03172-3 PMC8522114

[194] J. M. Hare , J. E. Fishman , G. Gerstenblith , et al., “Comparison of Allogeneic vs Autologous Bone Marrow–derived Mesenchymal Stem Cells Delivered by Transendocardial Injection in Patients With Ischemic Cardiomyopathy: The POSEIDON Randomized Trial,” Jama 308, no. 22 (2012): 2369–2379. 23117550 10.1001/jama.2012.25321 PMC4762261

[195] A. B. Mathiasen , A. A. Qayyum , E. Jørgensen , et al., “Bone Marrow‐derived Mesenchymal Stromal Cell Treatment in Patients With Severe Ischaemic Heart Failure: A Randomized Placebo‐controlled Trial (MSC‐HF trial),” European Heart Journal 36, no. 27 (2015): 1744–1753. 25926562 10.1093/eurheartj/ehv136

[196] X. He , Q. Wang , Y. Zhao , et al., “Effect of Intramyocardial Grafting Collagen Scaffold With Mesenchymal Stromal Cells in Patients With Chronic Ischemic Heart Disease: A Randomized Clinical Trial,” JAMA Network Open 3 (2020): e2016236. 32910197 10.1001/jamanetworkopen.2020.16236 PMC7489863

[197] J. Bartunek , A. Terzic , B. A. Davison , et al., “Cardiopoietic Cell Therapy for Advanced Ischaemic Heart Failure: Results at 39 Weeks of the Prospective, Randomized, Double Blind, Sham‐controlled CHART‐1 Clinical Trial,” European Heart Journal 38, no. 9 (2017): 648–660. 28025189 10.1093/eurheartj/ehw543 PMC5381596

[198] S. Miyagawa , S. Kainuma , T. Kawamura , et al., “Case Report: Transplantation of human Induced Pluripotent Stem Cell‐derived Cardiomyocyte Patches for Ischemic Cardiomyopathy,” Frontiers in Cardiovascular Medicine 9 (2022): 950829. 36051285 10.3389/fcvm.2022.950829 PMC9426776

[199] T. K. Gill , M. M. Mittinty , L. M. March , et al., “Global, Regional, and National Burden of Other Musculoskeletal Disorders, 1990–2020, and Projections to 2050: A Systematic Analysis of the Global Burden of Disease Study 2021,” The Lancet Rheumatology 5, no. 11 (2023): e670–e682. 37927903 10.1016/S2665-9913(23)00232-1 PMC10620749

[200] B. M. Fullen , H. Wittink , A. De Groef , et al., “Musculoskeletal Pain: Current and Future Directions of Physical Therapy Practice,” Archives of Rehabilitation Research and Clinical Translation 5, no. 1 (2023): 100258. 36968175 10.1016/j.arrct.2023.100258 PMC10036231

[201] V. Molnar , E. Pavelić , K. Vrdoljak , et al., “Mesenchymal Stem Cell Mechanisms of Action and Clinical Effects in Osteoarthritis: A Narrative Review,” Genes 13, no. 6 (2022): 949. 35741711 10.3390/genes13060949 PMC9222975

[202] J. Matas , C. García , D. Poblete , et al., “A Phase I Dose‐escalation Clinical Trial to Assess the Safety and Efficacy of Umbilical Cord‐derived Mesenchymal Stromal Cells in Knee Osteoarthritis,” Stem Cells Translational Medicine 13, no. 3 (2024): 193–203. 38366909 10.1093/stcltm/szad088 PMC10940813

[203] K.‐I. Kim , M. C. Lee , J. H. Lee , et al., “Clinical Efficacy and Safety of the Intra‐articular Injection of Autologous Adipose‐derived Mesenchymal Stem Cells for Knee Osteoarthritis: A Phase III, Randomized, Double‐blind, Placebo‐controlled Trial,” The American Journal of Sports Medicine 51, no. 9 (2023): 2243–2253. 37345256 10.1177/03635465231179223

[204] D. Jevotovsky , A. Alfonso , T. Einhorn , and E. Chiu , “Osteoarthritis and Stem Cell Therapy in Humans: A Systematic Review,” Osteoarthritis and Cartilage 26, no. 6 (2018): 711–729. 29544858 10.1016/j.joca.2018.02.906

[205] T. G. Wiggers , M. Winters , N. A. Van den Boom , H. J. Haisma , and M. H. Moen , “Autologous Stem Cell Therapy in Knee Osteoarthritis: A Systematic Review of Randomised Controlled Trials,” British Journal of Sports Medicine 55, no. 20 (2021): 1161–1169. 34039582 10.1136/bjsports-2020-103671

[206] P. Peláez , E. Damiá , M. Torres‐Torrillas , et al., “Cell and Cell Free Therapies in Osteoarthritis,” Biomedicines 9, no. 11 (2021): 1726. 34829953 10.3390/biomedicines9111726 PMC8615373

[207] M. Lee , S. Y. Jeong , J. Ha , et al., “Low Immunogenicity of Allogeneic human Umbilical Cord Blood‐derived Mesenchymal Stem Cells in Vitro and in Vivo,” Biochemical and Biophysical Research Communications 446, no. 4 (2014): 983–989. 24657442 10.1016/j.bbrc.2014.03.051

[208] J.‐S. Song , K.‐T. Hong , N.‐M. Kim , et al., “Implantation of Allogenic Umbilical Cord Blood‐derived Mesenchymal Stem Cells Improves Knee Osteoarthritis Outcomes: Two‐year Follow‐up,” Regenerative Therapy 14 (2020): 32–39. 31988992 10.1016/j.reth.2019.10.003 PMC6965506

[209] C. Albrecht . Arthroscopic cartilage cell transplantation (Spherox).

[210] P. Behrens , T. Bitter , B. Kurz , and M. Russlies , “Matrix‐associated Autologous Chondrocyte Transplantation/Implantation (MACT/MACI)—5‐year Follow‐up,” The Knee 13, no. 3 (2006): 194–202. 16632362 10.1016/j.knee.2006.02.012

[211] B. Sadri , M. Hassanzadeh , A. Bagherifard , et al., “Cartilage Regeneration and Inflammation Modulation in Knee Osteoarthritis Following Injection of Allogeneic Adipose‐derived Mesenchymal Stromal Cells: A Phase II, Triple‐blinded, Placebo Controlled, Randomized Trial,” Stem Cell Research & Therapy 14, no. 1 (2023): 162. 37316949 10.1186/s13287-023-03359-8 PMC10268462

[212] A. Saito , A. Ooki , T. Nakamura , et al., “Targeted Reversion of Induced Pluripotent Stem Cells From Patients With human Cleidocranial Dysplasia Improves Bone Regeneration in a Rat Calvarial Bone Defect Model,” Stem Cell Research & Therapy 9 (2018): 1–10. 29357927 10.1186/s13287-017-0754-4 PMC5778688

[213] C. Long , J. R. McAnally , J. M. Shelton , A. A. Mireault , R. Bassel‐Duby , and E. N. Olson , “Prevention of Muscular Dystrophy in Mice by CRISPR/Cas9–mediated Editing of Germline DNA,” Science 345, no. 6201 (2014): 1184–1188. 25123483 10.1126/science.1254445 PMC4398027

[214] C. M. van Gelder , A. A. Vollebregt , I. Plug , A. T. van der Ploeg , and A. J. Reuser , “Treatment Options for Lysosomal Storage Disorders: Developing Insights,” Expert Opinion on Pharmacotherapy 13, no. 16 (2012): 2281–2299. 23009070 10.1517/14656566.2012.729039

[215] M. Fukami , “Ovarian Dysfunction in Women With Turner Syndrome,” Frontiers in Endocrinology (Lausanne) 14 (2023): 1160258. 10.3389/fendo.2023.1160258 PMC10076527 37033245

[216] S. L. Sherman , “Premature Ovarian Failure in the Fragile X Syndrome,” American Journal of Medical Genetics 97, no. 3 (2000): 189–194. 11449487 10.1002/1096-8628(200023)97:3<189::AID-AJMG1036>3.0.CO;2-J

[217] H. Ke , S. Tang , T. Guo , et al., “Landscape of Pathogenic Mutations in Premature Ovarian Insufficiency,” Nature Medicine 29, no. 2 (2023): 483–492. 10.1038/s41591-022-02194-3 PMC9941050 36732629

[218] R. Beranger , P. Hoffmann , S. Christin‐Maitre , and V. Bonneterre , “Occupational Exposures to Chemicals as a Possible Etiology in Premature Ovarian Failure: A Critical Analysis of the Literature,” Reproductive Toxicology 33, no. 3 (2012): 269–279. 22281303 10.1016/j.reprotox.2012.01.002

[219] M. Ebrahimi and F. Akbari Asbagh , “Pathogenesis and Causes of Premature Ovarian Failure: An Update,” International Journal of Fertility and Sterility 5, no. 2 (2011): 54–65. 24963360 PMC4059950

[220] H. Gao , L. Gao , and W. Wang , “Advances in the Cellular Immunological Pathogenesis and Related Treatment of Primary Ovarian Insufficiency,” American Journal of Reproductive Immunology 88, no. 5 (2022): e13622. 36087022 10.1111/aji.13622

[221] C. Yamashiro , K. Sasaki , Y. Yabuta , et al., “Generation of human Oogonia From Induced Pluripotent Stem Cells in Vitro,” Science 362, no. 6412 (2018): 356–360. 30237246 10.1126/science.aat1674

[222] K. Zou , Z. Yuan , Z. Yang , et al., “Production of Offspring From a Germline Stem Cell Line Derived From Neonatal Ovaries,” Nature Cell Biology 11, no. 5 (2009): 631–636. 19363485 10.1038/ncb1869

[223] Y. A. R. White , D. C. Woods , Y. Takai , O. Ishihara , H. Seki , and J. L. Tilly , “Oocyte Formation by Mitotically Active Germ Cells Purified From Ovaries of Reproductive‐age Women,” Nature Medicine 18, no. 3 (2012): 413–421. 10.1038/nm.2669 PMC3296965 22366948

[224] Z. Li , M. Zhang , Y. Tian , Q. Li , and X. Huang , “Mesenchymal Stem Cells in Premature Ovarian Insufficiency: Mechanisms and Prospects,” Frontiers in Cell and Developmental Biology 9 (2021): 718192. 34414193 10.3389/fcell.2021.718192 PMC8369507

[225] S. Sadeghi , N. Mosaffa , B. Huang , and F. Ramezani Tehrani , “Protective Role of Stem Cells in POI: Current Status and Mechanism of Action, a Review Article,” Heliyon 10, no. 1 (2024): e23271. 38169739 10.1016/j.heliyon.2023.e23271 PMC10758796

[226] X. Wang , T. Li , X. Bai , Y. Zhu , M. Zhang , and L. Wang , “Therapeutic Prospect on Umbilical Cord Mesenchymal Stem Cells in Animal Model With Primary Ovarian Insufficiency: A Meta‐analysis,” Frontiers in Medicine 10 (2023): 1211070. 37324123 10.3389/fmed.2023.1211070 PMC10264577

[227] C. Guo , Y. Ma , Y. Situ , et al., “Mesenchymal Stem Cells Therapy Improves Ovarian Function in Premature Ovarian Failure: A Systematic Review and Meta‐analysis Based on Preclinical Studies,” Frontiers in Endocrinology 14 (2023): 1165574. 37484938 10.3389/fendo.2023.1165574 PMC10361781

[228] H. K. Kim and T. J. Kim , “Current Status and Future Prospects of Stem Cell Therapy for Infertile Patients With Premature Ovarian Insufficiency,” Biomolecules 14, no. 2 (2024): 242. 38397479 10.3390/biom14020242 PMC10887045

[229] K. Bahrehbar , M. Rezazadeh Valojerdi , F. Esfandiari , R. Fathi , S. N. Hassani , and H. Baharvand , “Human Embryonic Stem Cell‐derived Mesenchymal Stem Cells Improved Premature Ovarian Failure,” World Journal of Stem Cells 12, no. 8 (2020): 857–878. 32952863 10.4252/wjsc.v12.i8.857 PMC7477659

[230] R. W. Rebar , “Premature Ovarian Failure,” Obstetrics and Gynecology 113, no. 6 (2009): 1355–1363. 19461434 10.1097/AOG.0b013e3181a66843

[231] S. Herraiz , M. Romeu , A. Buigues , et al., “Autologous Stem Cell Ovarian Transplantation to Increase Reproductive Potential in Patients Who Are Poor Responders,” Fertility and Sterility 110, no. 3 (2018): 496–505. e1. 29960701 10.1016/j.fertnstert.2018.04.025

[232] L. Ding , G. Yan , B. Wang , et al., “Transplantation of UC‐MSCs on Collagen Scaffold Activates Follicles in Dormant Ovaries of POF Patients With Long History of Infertility,” Science China Life Sciences 61, no. 12 (2018): 1554–1565. 29546669 10.1007/s11427-017-9272-2

[233] L. Yan , Y. Wu , L. Li , et al., “Clinical Analysis of human Umbilical Cord Mesenchymal Stem Cell Allotransplantation in Patients With Premature Ovarian Insufficiency,” Cell Proliferation 53, no. 12 (2020): e12938. 33124125 10.1111/cpr.12938 PMC7705906

[234] S. Zafardoust , S. Kazemnejad , M. Darzi , M. Fathi‐Kazerooni , H. Rastegari , and A. Mohammadzadeh , “Improvement of Pregnancy Rate and Live Birth Rate in Poor Ovarian Responders by Intraovarian Administration of Autologous Menstrual Blood Derived‐ Mesenchymal Stromal Cells: Phase I/II Clinical Trial,” Stem Cell Reviews and Reports 16, no. 4 (2020): 755–763. 32198596 10.1007/s12015-020-09969-6

[235] D. D. Manavella , L. Cacciottola , S. Pomme , et al., “Two‐step Transplantation With Adipose Tissue‐derived Stem Cells Increases Follicle Survival by Enhancing Vascularization in Xenografted Frozen‐thawed human Ovarian Tissue,” Human Reproduction 33, no. 6 (2018): 1107–1116. 29635371 10.1093/humrep/dey080

[236] E. Dreisler and J. J. Kjer , “Asherman's Syndrome: Current Perspectives on Diagnosis and Management,” International Journal of Women's Health 11 (2019): 191–198. 10.2147/IJWH.S165474 PMC6430995 30936754

[237] P. Wangikar , T. Ahmed , and S. Vangala . Chapter 76 ‐ Toxicologic Pathology of the Reproductive System. In: Gupta RC , ed. “Reproductive and Developmental Toxicology”. (Academic Press, 2011): 1003–1026.

[238] N. Singh , B. Shekhar , S. Mohanty , S. Kumar , T. Seth , and B. Girish , “Autologous Bone Marrow‐derived Stem Cell Therapy for Asherman's Syndrome and Endometrial Atrophy: A 5‐year Follow‐up Study,” Journal of Human Reproductive Sciences 13, no. 1 (2020): 31. 32577066 10.4103/jhrs.JHRS_64_19 PMC7295252

[239] S. Y. Lee , J. E. Shin , H. Kwon , D. H. Choi , and J. H. Kim , “Effect of Autologous Adipose‐Derived Stromal Vascular Fraction Transplantation on Endometrial Regeneration in Patients of Asherman's Syndrome: A Pilot Study,” Reproductive Sciences 27, no. 2 (2020): 561–568. 32046396 10.1007/s43032-019-00055-y

[240] H. Ma , M. Liu , Y. Li , et al., “Intrauterine Transplantation of Autologous Menstrual Blood Stem Cells Increases Endometrial Thickness and Pregnancy Potential in Patients With Refractory Intrauterine Adhesion,” Journal of Obstetrics and Gynaecology Research 46, no. 11 (2020): 2347–2355. 32856391 10.1111/jog.14449

[241] Y. Cao , H. Sun , H. Zhu , et al., “Allogeneic Cell Therapy Using Umbilical Cord MSCs on Collagen Scaffolds for Patients With Recurrent Uterine Adhesion: A Phase I Clinical Trial,” Stem Cell Research & Therapy 9, no. 1 (2018): 192. 29996892 10.1186/s13287-018-0904-3 PMC6042450

[242] Y. Zhang , L. Shi , X. Lin , et al., “Unresponsive Thin Endometrium Caused by Asherman Syndrome Treated With Umbilical Cord Mesenchymal Stem Cells on Collagen Scaffolds: A Pilot Study,” Stem Cell Research & Therapy 12, no. 1 (2021): 420. 34294152 10.1186/s13287-021-02499-z PMC8296628

[243] L. Nguyen Thanh , P. T. M. Dam , H. P. Nguyen , et al., “Can Autologous Adipose‐Derived Mesenchymal Stem Cell Transplantation Improve Sexual Function in People With Sexual Functional Deficiency?,” Stem Cell Reviews and Reports 17, no. 6 (2021): 2153–2163. 34129158 10.1007/s12015-021-10196-w

[244] I. Mastrolia , E. M. Foppiani , A. Murgia , et al., “Challenges in Clinical Development of Mesenchymal Stromal/Stem Cells: Concise Review,” Stem Cells Translational Medicine 8, no. 11 (2019): 1135–1148. 31313507 10.1002/sctm.19-0044 PMC6811694

[245] A. B. Česnik and U. Švajger , “The Issue of Heterogeneity of MSC‐based Advanced Therapy Medicinal Products–a Review,” Frontiers in Cell and Developmental Biology 12 (2024): 1400347. 39129786 10.3389/fcell.2024.1400347 PMC11310176

[246] S. Chen , B. Liang , and J. Xu , “Unveiling Heterogeneity in MSCs: Exploring Marker‐based Strategies for Defining MSC Subpopulations,” Journal of Translational Medicine 22, no. 1 (2024): 459. 38750573 10.1186/s12967-024-05294-5 PMC11094970

[247] S. Ikehara , Grand challenges in stem cell treatments. (Frontiers Media SA, 2013): 2. biology d. 10.3389/fcell.2013.00002 PMC4206984 25364707

[248] A. Musiał‐Wysocka , M. Kot , and M. Majka , “The Pros and Cons of Mesenchymal Stem Cell‐based Therapies,” Cell Transplantation 28, no. 7 (2019): 801–812. 31018669 10.1177/0963689719837897 PMC6719501

[249] G. Moll , I. Rasmusson‐Duprez , L. von Bahr , et al., “Are Therapeutic human Mesenchymal Stromal Cells Compatible With human Blood?,” Stem Cells 30, no. 7 (2012): 1565–1574. 22522999 10.1002/stem.1111

[250] G. Moll , J. A. Ankrum , S. D. Olson , and J. A. Nolta , “Improved MSC Minimal Criteria to Maximize Patient Safety: A Call to Embrace Tissue Factor and Hemocompatibility Assessment of MSC Products,” Stem Cells Translational Medicine 11, no. 1 (2022): 2–13. 35641163 10.1093/stcltm/szab005 PMC8895495

[251] O. Repetto and V. De Re , “Coagulation and Fibrinolysis in Gastric Cancer,” Annals of the New York Academy of Sciences 1404, no. 1 (2017): 27–48. 28833193 10.1111/nyas.13454

[252] H. Caplan , S. D. Olson , A. Kumar , et al., “Mesenchymal Stromal Cell Therapeutic Delivery: Translational Challenges to Clinical Application,” Frontiers in Immunology 10 (2019): 1645. 31417542 10.3389/fimmu.2019.01645 PMC6685059

[253] V. T. Hoang , D. S. Le , D. M. Hoang , et al., “Impact of Tissue Factor Expression and Administration Routes on Thrombosis Development Induced by Mesenchymal Stem/Stromal Cell Infusions: Re‐evaluating the Dogma,” Stem Cell Research & Therapy 15, no. 1 (2024): 56. 38414067 10.1186/s13287-023-03582-3 PMC10900728

[254] S. M. Ridge , F. J. Sullivan , and S. A. Glynn , “Mesenchymal Stem Cells: Key Players in Cancer Progression,” Molecular Cancer 16, no. 1 (2017): 31. 28148268 10.1186/s12943-017-0597-8 PMC5286812

[255] T. Lan , M. Luo , and X. J. Wei , “Mesenchymal Stem/Stromal Cells in Cancer Therapy,” Journal of Hematology & Oncology 14 (2021): 1–16. 34789315 10.1186/s13045-021-01208-w PMC8596342

[256] R. W. Y. Yeo and S. K. Lim , “Embryonic Stem Cells for Therapies–challenges and Possibilities,” Embryonic Stem Cells—Basic Biology to Bioengineering (2011).

[257] H. Liu , S. Reiter , X. Zhou , et al., “Insight Into the Mechanisms and the Challenges on Stem Cell‐based Therapies for Cerebral Ischemic Stroke,” Frontiers in Cellular Neuroscience 15 (2021): 637210. 33732111 10.3389/fncel.2021.637210 PMC7959708

[258] M. Ohnuki and K. Takahashi , “Present and Future Challenges of Induced Pluripotent Stem Cells,” Philosophical Transactions of the Royal Society B: Biological Sciences 370, no. 1680 (2015): 20140367. 10.1098/rstb.2014.0367 PMC4633996 26416678

[259] K. Okita and S. Yamanaka , “Induced Pluripotent Stem Cells: Opportunities and Challenges,” Philosophical Transactions of the Royal Society B: Biological Sciences 366, no. 1575 (2011): 2198–2207. 10.1098/rstb.2011.0016 PMC3130417 21727125

[260] C. Zhong , M. Liu , X. Pan , and H. Zhu , “Tumorigenicity Risk of iPSCs in Vivo: Nip It in the Bud,” Precision Clinical Medicine 5, no. 1 (2022): pbac004. 35692443 10.1093/pcmedi/pbac004 PMC9026204

[261] Y. Liang , H. Zhang , Q.‐S. Feng , et al., “The Propensity for Tumorigenesis in human Induced Pluripotent Stem Cells Is Related With Genomic Instability,” Chinese Journal of Cancer 32, no. 4 (2013): 205. 22704487 10.5732/cjc.012.10065 PMC3845575

[262] G. Liang and Y. Zhang , “Embryonic Stem Cell and Induced Pluripotent Stem Cell: An Epigenetic Perspective,” Cell Research 23, no. 1 (2013): 49–69. 23247625 10.1038/cr.2012.175 PMC3541668

[263] M. Yoshihara , Y. Hayashizaki , and Y. Murakawa , “Genomic Instability of iPSCs: Challenges towards Their Clinical Applications,” Stem Cell Reviews and Reports 13 (2017): 7–16. 27592701 10.1007/s12015-016-9680-6 PMC5346115

[264] A. E. Omole , A. O. J. Fakoya , K. C. Nnawuba , and K. H. Haider , “Common Ethical Considerations of human‐induced Pluripotent Stem Cell Research,” Handbook of stem cell therapy. (Springer, 2022): 1–17.

[265] A. B. Moy , A. Kamath , S. Ternes , and J. Kamath , “The Challenges to Advancing Induced Pluripotent Stem Cell‐Dependent Cell Replacement Therapy,” Medical Research Archives 11, no. 11 (2023): 4784. 38188933 10.18103/mra.v11i11.4784 PMC10768945

[266] Y. Tang , P. Yu , and L. J. Cheng , “Current Progress in the Derivation and Therapeutic Application of Neural Stem Cells,” Cell Death & Disease 8, no. 10 (2017): e3108–e3108. 29022921 10.1038/cddis.2017.504 PMC5682670

[267] S. Pluchino , J. A. Smith , and L. Peruzzotti‐Jametti , “Promises and Limitations of Neural Stem Cell Therapies for Progressive Multiple Sclerosis,” Trends in Molecular Medicine 26, no. 10 (2020): 898–912. 32448751 10.1016/j.molmed.2020.04.005

[268] C. Yue , S. Feng , Y. Chen , and N. Jing , “The Therapeutic Prospects and Challenges of human Neural Stem Cells for the Treatment of Alzheimer's Disease,” Cell Regen 11, no. 1 (2022): 28. 36050613 10.1186/s13619-022-00128-5 PMC9437172

[269] V. Miceli , G. Zito , M. Bulati , et al., “Different Priming Strategies Improve Distinct Therapeutic Capabilities of Mesenchymal Stromal/Stem Cells: Potential Implications for Their Clinical Use,” World Journal of Stem Cells 15, no. 5 (2023): 400. 37342218 10.4252/wjsc.v15.i5.400 PMC10277962

[270] E. Nasiri , A. Alizadeh , A. M. Roushandeh , R. Gazor , N. Hashemi‐Firouzi , and Z. Golipoor , “Melatonin‐pretreated Adipose‐derived Mesenchymal Stem Cells Efficeintly Improved Learning, Memory, and Cognition in an Animal Model of Alzheimer's Disease,” Metabolic Brain Disease 34 (2019): 1131–1143. 31129766 10.1007/s11011-019-00421-4

[271] M. I. Phillips and Y. L. Tang , “Genetic Modification of Stem Cells for Transplantation,” Advanced Drug Delivery Reviews 60, no. 2 (2008): 160–172. 18031863 10.1016/j.addr.2007.08.035 PMC2734411

[272] N. C. Leite , G. C. Pelayo , and D. A. Melton , “Genetic Manipulation of Stress Pathways Can Protect Stem‐cell‐derived Islets From Apoptosis in Vitro,” Stem Cell Reports 17, no. 4 (2022): 766–774. 35245439 10.1016/j.stemcr.2022.01.018 PMC9023776

[273] M. Kot , M. Baj‐Krzyworzeka , R. Szatanek , A. Musiał‐Wysocka , M. Suda‐Szczurek , and M. Majka , “The Importance of HLA Assessment in “off‐the‐shelf” Allogeneic Mesenchymal Stem Cells Based‐therapies,” International Journal of Molecular Sciences 20, no. 22 (2019): 5680. 31766164 10.3390/ijms20225680 PMC6888380

[274] Y. Shan , M. Zhang , E. Tao , et al., “Pharmacokinetic Characteristics of Mesenchymal Stem Cells in Translational Challenges,” Signal Transduction and Targeted Therapy 9, no. 1 (2024): 242. 39271680 10.1038/s41392-024-01936-8 PMC11399464

[275] S. Pellegrini , V. Zamarian , and V. Sordi , “Strategies to Improve the Safety of iPSC‐derived β Cells for β Cell Replacement in Diabetes,” Transplant International 35 (2022): 10575. 36090777 10.3389/ti.2022.10575 PMC9448870

[276] S. Park , Y. Gwon , S. A. Khan , K.‐J. Jang , and J. Kim , “Engineering Considerations of iPSC‐based Personalized Medicine,” Biomaterials Research 27, no. 1 (2023): 67. 37420273 10.1186/s40824-023-00382-x PMC10326963

[277] V. Truong , K. Viken , Z. Geng , et al., “Automating human Induced Pluripotent Stem Cell Culture and Differentiation of iPSC‐derived Retinal Pigment Epithelium for Personalized Drug Testing,” Slas Technology: Translating Life Sciences Innovation 26, no. 3 (2021): 287–299. 10.1177/2472630320972110 PMC8140989 33292045

[278] A. Dimitri , F. Herbst , and J. A. Fraietta , “Engineering the next‐generation of CAR T‐cells With CRISPR‐Cas9 Gene Editing,” Molecular Cancer 21, no. 1 (2022): 78. 35303871 10.1186/s12943-022-01559-z PMC8932053

[279] H. Lin , Q. Li , Q. Du , et al., “Integrated Generation of Induced Pluripotent Stem Cells in a Low‐cost Device,” Biomaterials 189 (2019): 23–36. 30384126 10.1016/j.biomaterials.2018.10.027

[280] S. Yamanaka , “Pluripotent Stem Cell‐based Cell Therapy—promise and Challenges,” Cell Stem Cell 27, no. 4 (2020): 523–531. 33007237 10.1016/j.stem.2020.09.014

[281] R. De Gioia , F. Biella , G. Citterio , et al., “Neural Stem Cell Transplantation for Neurodegenerative Diseases,” International Journal of Molecular Sciences 21, no. 9 (2020): 3103. 32354178 10.3390/ijms21093103 PMC7247151

[282] G. M. Thomsen , P. Avalos , A. A. Ma , et al., “Transplantation of Neural Progenitor Cells Expressing Glial Cell Line‐derived Neurotrophic Factor Into the Motor Cortex as a Strategy to Treat Amyotrophic Lateral sclerosis,” Stem Cells 36, no. 7 (2018): 1122–1131. 29656478 10.1002/stem.2825

[283] M. Khazaei , C. S. Ahuja , H. Nakashima , et al., “GDNF Rescues the Fate of Neural Progenitor Grafts by Attenuating Notch Signals in the Injured Spinal Cord in Rodents,” Science Translational Medicine 12, no. 525 (2020): eaau3538. 31915299 10.1126/scitranslmed.aau3538

[284] X. Li , Z. Peng , L. Long , et al., “Wnt4‐modified NSC Transplantation Promotes Functional Recovery After Spinal Cord Injury,” The FASEB Journal 34, no. 1 (2020): 82–94. 31914702 10.1096/fj.201901478RR

[285] D. Park , Y.‐H. Yang , D. K. Bae , et al., “Improvement of Cognitive Function and Physical Activity of Aging Mice by human Neural Stem Cells Over‐expressing Choline Acetyltransferase,” Neurobiology of Aging 34, no. 11 (2013): 2639–2646. 23731954 10.1016/j.neurobiolaging.2013.04.026

[286] S. Mathivanan , P. Fonseka , C. Nedeva , and I. Atukorala . New Frontiers: Extracellular Vesicles. vol 97. Springer; 2021.

[287] C. Théry , K. W. Witwer , E. Aikawa , et al., “Minimal Information for Studies of Extracellular Vesicles 2018 (MISEV2018): A Position Statement of the International Society for Extracellular Vesicles and Update of the MISEV2014 Guidelines,” Journal of Extracellular Vesicles 7, no. 1 (2018): 1535750. 30637094 10.1080/20013078.2018.1535750 PMC6322352

[288] G. Berumen Sánchez , K. E. Bunn , H. H. Pua , and M. Rafat , “Extracellular Vesicles: Mediators of Intercellular Communication in Tissue Injury and Disease,” Cell Communication and Signaling 19, no. 1 (2021): 104. 34656117 10.1186/s12964-021-00787-y PMC8520651

[289] L. M. Doyle and M. Z. Wang , “Overview of Extracellular Vesicles, Their Origin, Composition, Purpose, and Methods for Exosome Isolation and Analysis,” Cells 8, no. 7 (2019): 727. 31311206 10.3390/cells8070727 PMC6678302

[290] S. Gandham , X. Su , J. Wood , et al., “Technologies and Standardization in Research on Extracellular Vesicles,” Trends in Biotechnology 38, no. 10 (2020): 1066–1098. 32564882 10.1016/j.tibtech.2020.05.012 PMC7302792

[291] S. Gurung , D. Perocheau , L. Touramanidou , and J. Baruteau , “The Exosome Journey: From Biogenesis to Uptake and Intracellular Signalling,” Cell Communication and Signaling 19, no. 1 (2021): 47. 33892745 10.1186/s12964-021-00730-1 PMC8063428

[292] V. R. Minciacchi , M. R. Freeman , and D. Di Vizio , “Extracellular Vesicles in Cancer: Exosomes, Microvesicles and the Emerging Role of Large Oncosomes,” Seminars in Cell & Developmental Biology 40 (2015): 41–51. 25721812 10.1016/j.semcdb.2015.02.010 PMC4747631

[293] X. Zou , Q. Lei , X. Luo , et al., “Advances in Biological Functions and Applications of Apoptotic Vesicles,” Cell Communication and Signaling 21, no. 1 (2023): 260. 37749626 10.1186/s12964-023-01251-9 PMC10519056

[294] M. M. Shi , Q. Y. Yang , A. Monsel , et al., “Preclinical Efficacy and Clinical Safety of Clinical‐grade Nebulized Allogenic Adipose Mesenchymal Stromal Cells‐derived Extracellular Vesicles,” Journal of Extracellular Vesicles 10, no. 10 (2021): e12134. 34429860 10.1002/jev2.12134 PMC8363910

[295] X. Xie , Q. Song , C. Dai , et al., “Clinical Safety and Efficacy of Allogenic human Adipose Mesenchymal Stromal Cells‐derived Exosomes in Patients With Mild to Moderate Alzheimer's Disease: A Phase I/II Clinical Trial,” General Psychiatry 36, no. 5 (2023): e101143. 37859748 10.1136/gpsych-2023-101143 PMC10582850

[296] M. Akhlaghpasand , R. Tavanaei , M. Hosseinpoor , et al., “Safety and Potential Effects of Intrathecal Injection of Allogeneic human Umbilical Cord Mesenchymal Stem Cell‐derived Exosomes in Complete Subacute Spinal Cord Injury: A First‐in‐human, Single‐arm, Open‐label, Phase I Clinical Trial,” Stem Cell Research & Therapy 15, no. 1 (2024): 264. 39183334 10.1186/s13287-024-03868-0 PMC11346059

[297] V. Sengupta , S. Sengupta , A. Lazo , P. Woods , A. Nolan , and N. Bremer , “Exosomes Derived From Bone Marrow Mesenchymal Stem Cells as Treatment for Severe COVID‐19,” Stem Cells and Development 29, no. 12 (2020): 747–754. 32380908 10.1089/scd.2020.0080 PMC7310206

[298] Y. G. Zhu , M. M. Shi , A. Monsel , et al., “Nebulized Exosomes Derived From Allogenic Adipose Tissue Mesenchymal Stromal Cells in Patients With Severe COVID‐19: A Pilot Study,” Stem Cell Research & Therapy 13, no. 1 (2022): 220. 35619189 10.1186/s13287-022-02900-5 PMC9135389

[299] M. Chu , H. Wang , L. Bian , et al., “Nebulization Therapy With Umbilical Cord Mesenchymal Stem Cell‐Derived Exosomes for COVID‐19 Pneumonia,” Stem Cell Reviews and Reports 18, no. 6 (2022): 2152–2163. 35665467 10.1007/s12015-022-10398-w PMC9166932

[300] A. L. Lightner , V. Sengupta , S. Qian , et al., “Bone Marrow Mesenchymal Stem Cell‐Derived Extracellular Vesicle Infusion for the Treatment of Respiratory Failure from COVID‐19: A Randomized, Placebo‐Controlled Dosing Clinical Trial,” Chest 164, no. 6 (2023): 1444–1453. 37356708 10.1016/j.chest.2023.06.024 PMC10289818

[301] M. H. Zamanian , A. H. Norooznezhad , Z. Hosseinkhani , et al., “Human Placental Mesenchymal Stromal Cell‐derived Small Extracellular Vesicles as a Treatment for Severe COVID‐19: A Double‐blind Randomized Controlled Clinical Trial,” Journal of Extracellular Vesicles 13, no. 7 (2024): e12492. 39051747 10.1002/jev2.12492 PMC11270582

[302] P. Menasche , N. K. Renault , A. Hagege , et al., “First‐in‐man Use of a Cardiovascular Cell‐derived Secretome in Heart Failure. Case Report,” EBioMedicine 103 (2024): 105145. 38713924 10.1016/j.ebiom.2024.105145 PMC11096705

[303] J. East and M. Dordevic , “Pilot Safety Study of an Extracellular VesicleIsolate Product for Treatment ofOsteoarthritis in Combat‐Related Injuries:One Year Follow up,” Genesis Scientific Journals 2, no. 2 (2021): 1–10.

[304] H. H. Kwon , S. H. Yang , J. Lee , et al., “Combination Treatment With Human Adipose Tissue Stem Cell‐derived Exosomes and Fractional CO2 Laser for Acne Scars: A 12‐week Prospective, Double‐blind, Randomized, Split‐face Study,” Acta Dermato‐Venereologica 100, no. 18 (2020): adv00310. 33073298 10.2340/00015555-3666 PMC9309822

[305] J. Johnson , S. Q. K. Law , M. Shojaee , et al., “First‐in‐human Clinical Trial of Allogeneic, Platelet‐derived Extracellular Vesicles as a Potential Therapeutic for Delayed Wound Healing,” Journal of Extracellular Vesicles 12, no. 7 (2023): e12332. 37353884 10.1002/jev2.12332 PMC10290200

[306] G. H. Park , H. H. Kwon , J. Seok , et al., “Efficacy of Combined Treatment With human Adipose Tissue Stem Cell‐derived Exosome‐containing Solution and Microneedling for Facial Skin Aging: A 12‐week Prospective, Randomized, Split‐face Study,” Journal of Cosmetic Dermatology 22, no. 12 (2023): 3418–3426. 37377400 10.1111/jocd.15872

[307] B. Yavuz , E. C. Mutlu , Z. Ahmed , B. Ben‐Nissan , and A. Stamboulis , “Applications of Stem Cell‐Derived Extracellular Vesicles in Nerve Regeneration,” International Journal of Molecular Sciences 25, no. 11 (2024): 5863. 38892052 10.3390/ijms25115863 PMC11172915

[308] B. Wei , M. Wei , H. Huang , T. Fan , Z. Zhang , and X. Song , “Mesenchymal Stem Cell‐Derived Exosomes: A Promising Therapeutic Strategy for Age‐Related Diseases,” Cell Proliferation (2024): e13795. 39704104 10.1111/cpr.13795 PMC12099225

[309] G. Singh , A. Mehra , S. Arora , et al., “Exosome‐mediated Delivery and Regulation in Neurological Disease Progression,” International Journal of Biological Macromolecules 264, no. Pt 2 (2024): 130728. 38467209 10.1016/j.ijbiomac.2024.130728

[310] H. Ni , S. Yang , F. Siaw‐Debrah , et al., “Exosomes Derived from Bone Mesenchymal Stem Cells Ameliorate Early Inflammatory Responses Following Traumatic Brain Injury,” Frontiers in Neuroscience 13 (2019): 14. 30733666 10.3389/fnins.2019.00014 PMC6354067

[311] T. R. Doeppner , J. Herz , A. Gorgens , et al., “Extracellular Vesicles Improve Post‐Stroke Neuroregeneration and Prevent Postischemic Immunosuppression,” Stem Cells Translational Medicine 4, no. 10 (2015): 1131–1143. 26339036 10.5966/sctm.2015-0078 PMC4572905

[312] Y. Zhang , M. Chopp , Y. Meng , et al., “Effect of Exosomes Derived From Multipluripotent Mesenchymal Stromal Cells on Functional Recovery and Neurovascular Plasticity in Rats After Traumatic Brain Injury,” Journal of Neurosurgery 122, no. 4 (2015): 856–867. 25594326 10.3171/2014.11.JNS14770 PMC4382456

[313] Z. Zhu , X. Zhang , H. Hao , et al., “Exosomes Derived from Umbilical Cord Mesenchymal Stem Cells Treat Cutaneous Nerve Damage and Promote Wound Healing,” Frontiers in Cellular Neuroscience 16 (2022): 913009. 35846563 10.3389/fncel.2022.913009 PMC9279568

[314] H. R. Hofer and R. S. Tuan , “Secreted Trophic Factors of Mesenchymal Stem Cells Support Neurovascular and Musculoskeletal Therapies,” Stem Cell Research & Therapy 7, no. 1 (2016): 131. 27612948 10.1186/s13287-016-0394-0 PMC5016979

[315] K. Sankarappan and A. K. Shetty , “Promise of Mesenchymal Stem Cell‐derived Extracellular Vesicles for Alleviating Subarachnoid Hemorrhage‐induced Brain Dysfunction by Neuroprotective and Antiinflammatory Effects,” Brain, Behavior, & Immunity ‐ Health 40 (2024): 100835. 10.1016/j.bbih.2024.100835 PMC11334735 39165307

[316] X. Xiao , W. Li , D. Rong , et al., “Human Umbilical Cord Mesenchymal Stem Cells‐derived Extracellular Vesicles Facilitate the Repair of Spinal Cord Injury via the miR‐29b‐3p/PTEN/Akt/mTOR Axis,” Cell Death Discovery 7, no. 1 (2021): 212. 34381025 10.1038/s41420-021-00572-3 PMC8357833

[317] X. Hu , Z. Liu , X. Zhou , et al., “Small Extracellular Vesicles Derived From Mesenchymal Stem Cell Facilitate Functional Recovery in Spinal Cord Injury by Activating Neural Stem Cells via the ERK1/2 Pathway,” Frontiers in Cellular Neuroscience 16 (2022): 954597. 36106012 10.3389/fncel.2022.954597 PMC9464810

[318] W. Zhou , M. Silva , C. Feng , et al., “Exosomes Derived From human Placental Mesenchymal Stem Cells Enhanced the Recovery of Spinal Cord Injury by Activating Endogenous Neurogenesis,” Stem Cell Research & Therapy 12, no. 1 (2021): 174. 33712072 10.1186/s13287-021-02248-2 PMC7953814

[319] H. Wang , H. Zhao , Z. Chen , et al., “Hypoxic Bone Mesenchymal Stem Cell‐Derived Exosomes Direct Schwann Cells Proliferation, Migration, and Paracrine to Accelerate Facial Nerve Regeneration via circRNA_Nkd2/miR‐214‐3p/MED19 Axis,” International Journal of Nanomedicine 19 (2024): 1409–1429. 38371458 10.2147/IJN.S443036 PMC10871042

[320] J. Xie , B. Jin , D. W. Li , et al., “Effect of Laminin‐binding BDNF on Induction of Recurrent Laryngeal Nerve Regeneration by miR‐222 Activation of mTOR Signal Pathway,” American Journal of Translational Research 7, no. 6 (2015): 1071–1080. 26279751 PMC4532740

[321] J. Yang , B. Wang , Y. Wang , et al., “Exosomes Derived From Adipose Mesenchymal Stem Cells Carrying miRNA‐22‐3p Promote Schwann Cells Proliferation and Migration Through Downregulation of PTEN,” Disease Markers 2022 (2022): 7071877. 36148159 10.1155/2022/7071877 PMC9489425

[322] L. Chen , B. Xiang , X. Wang , and C. Xiang , “Exosomes Derived From human Menstrual Blood‐derived Stem Cells Alleviate Fulminant Hepatic Failure,” Stem Cell Research & Therapy: 8, no. 1 (2017): 9. 28115012 10.1186/s13287-016-0453-6 PMC5260032

[323] Y. Pan , W. F. Tan , M. Q. Yang , J. Y. Li , and D. A. Geller , “The Therapeutic Potential of Exosomes Derived From Different Cell Sources in Liver Diseases,” American Journal of Physiology Gastrointestinal and Liver Physiology 322, no. 4 (2022): G397–G404. 35107032 10.1152/ajpgi.00054.2021 PMC8917924

[324] L. Jiang , S. Zhang , H. Hu , et al., “Exosomes Derived From human Umbilical Cord Mesenchymal Stem Cells Alleviate Acute Liver Failure by Reducing the Activity of the NLRP3 Inflammasome in Macrophages,” Biochemical and Biophysical Research Communications 508, no. 3 (2019): 735–741. 30528233 10.1016/j.bbrc.2018.11.189

[325] Y. Jin , J. Wang , H. Li , et al., “Extracellular Vesicles Secreted by Human Adipose‐derived Stem Cells (hASCs) Improve Survival Rate of Rats With Acute Liver Failure by Releasing lncRNA H19,” EBioMedicine 34 (2018): 231–242. 30077720 10.1016/j.ebiom.2018.07.015 PMC6116414

[326] W. Jiang , Y. Tan , M. Cai , et al., “Human Umbilical Cord MSC‐Derived Exosomes Suppress the Development of CCl(4)‐Induced Liver Injury Through Antioxidant Effect,” Stem Cells International 2018 (2018): 6079642. 29686713 10.1155/2018/6079642 PMC5857330

[327] W. Lu , H. Tang , S. Li , L. Bai , and Y. Chen , “Extracellular Vesicles as Potential Biomarkers and Treatment Options for Liver Failure: A Systematic Review up to March 2022,” Frontiers in Immunology 14 (2023): 1116518. 36911706 10.3389/fimmu.2023.1116518 PMC9992400

[328] K. Xie , L. Liu , J. Chen , and F. Liu , “Exosomal miR‐1246 Derived From human Umbilical Cord Blood Mesenchymal Stem Cells Attenuates Hepatic Ischemia Reperfusion Injury by Modulating T Helper 17/Regulatory T Balance,” Iubmb Life 71, no. 12 (2019): 2020–2030. 31433911 10.1002/iub.2147

[329] M. Kou , L. Huang , J. Yang , et al., “Mesenchymal Stem Cell‐derived Extracellular Vesicles for Immunomodulation and Regeneration: A next Generation Therapeutic Tool?,” Cell Death & Disease 13, no. 7 (2022): 580. 35787632 10.1038/s41419-022-05034-x PMC9252569

[330] J. Janockova , L. Slovinska , D. Harvanova , T. Spakova , and J. Rosocha , “New Therapeutic Approaches of Mesenchymal Stem Cells‐derived Exosomes,” Journal of Biomedical Science 28, no. 1 (2021): 39. 34030679 10.1186/s12929-021-00736-4 PMC8143902

[331] Y. Zhang , Y. Li , Q. Wang , et al., “Attenuation of Hepatic Ischemia‑Reperfusion Injury by Adipose Stem Cell‑Derived Exosome Treatment via ERK1/2 and GSK‑3beta Signaling Pathways,” International Journal of Molecular Medicine 49, no. 2 (2022): 13. 34878156 10.3892/ijmm.2021.5068 PMC8711591

[332] X. J. Song , L. Zhang , Q. Li , Y. Li , F. H. Ding , and X. Li , “hUCB‐MSC Derived Exosomal miR‐124 Promotes Rat Liver Regeneration After Partial Hepatectomy via Downregulating Foxg1,” Life Sciences 265 (2021): 118821. 33275988 10.1016/j.lfs.2020.118821

[333] J. Lee , S. R. Kim , C. Lee , et al., “Extracellular Vesicles From in Vivo Liver Tissue Accelerate Recovery of Liver Necrosis Induced by Carbon Tetrachloride,” Journal of Extracellular Vesicles 10, no. 10 (2021): e12133. 34401049 10.1002/jev2.12133 PMC8357636

[334] L. Zhou , H. Luo , and J. W. Lee , “Role of Extracellular Vesicles in Lung Diseases,” Chinese Medical Journal 135, no. 15 (2022): 1765–1780. 35866573 10.1097/CM9.0000000000002118 PMC9521785

[335] E. F. Landry , J. M. Vaughn , T. J. Vicale , and R. Mann , “Inefficient Accumulation of Low Levels of Monodispersed and Feces‐associated Poliovirus in Oysters,” Applied and Environmental Microbiology 44, no. 6 (1982): 1362–1369. 6297388 10.1128/aem.44.6.1362-1369.1982 PMC242197

[336] Q. Hu , S. Zhang , Y. Yang , et al., “Extracellular Vesicles in the Pathogenesis and Treatment of Acute Lung Injury,” Military Medical Research 9, no. 1 (2022): 61. 36316787 10.1186/s40779-022-00417-9 PMC9623953

[337] N. Basalova , G. Sagaradze , M. Arbatskiy , et al., “Secretome of Mesenchymal Stromal Cells Prevents Myofibroblasts Differentiation by Transferring Fibrosis‐Associated microRNAs Within Extracellular Vesicles,” Cells 9, no. 5 (2020): 1272. 32443855 10.3390/cells9051272 PMC7290371

[338] K. Lv , Y. Wang , P. Lou , et al., “Extracellular Vesicles as Advanced Therapeutics for the Resolution of Organ Fibrosis: Current Progress and Future Perspectives,” Frontiers in Immunology 13 (2022): 1042983. 36341339 10.3389/fimmu.2022.1042983 PMC9630482

[339] Q. Li , Q. Feng , H. Zhou , et al., “Mechanisms and Therapeutic Strategies of Extracellular Vesicles in Cardiovascular Diseases,” MedComm 4, no. 6 (2023): e454. 38124785 10.1002/mco2.454 PMC10732331

[340] J. Zhao , X. Li , J. Hu , et al., “Mesenchymal Stromal Cell‐derived Exosomes Attenuate Myocardial Ischaemia‐reperfusion Injury Through miR‐182‐regulated Macrophage Polarization,” Cardiovascular Research 115, no. 7 (2019): 1205–1216. 30753344 10.1093/cvr/cvz040 PMC6529919

[341] R. Yue , S. Lu , Y. Luo , et al., “Mesenchymal Stem Cell‐derived Exosomal microRNA‐182‐5p Alleviates Myocardial Ischemia/Reperfusion Injury by Targeting GSDMD in Mice,” Cell Death Discovery 8, no. 1 (2022): 202. 35422485 10.1038/s41420-022-00909-6 PMC9010441

[342] D. Shen and Z. He , “Mesenchymal Stem Cell‐derived Exosomes Regulate the Polarization and Inflammatory Response of Macrophages via miR‐21‐5p to Promote Repair After Myocardial Reperfusion Injury,” Annals of Translational Medicine 9, no. 16 (2021): 1323. 34532460 10.21037/atm-21-3557 PMC8422151

[343] C. Liang , Y. Liu , H. Xu , et al., “Exosomes of Human Umbilical Cord MSCs Protect against Hypoxia/Reoxygenation‐Induced Pyroptosis of Cardiomyocytes via the miRNA‐100‐5p/FOXO3/NLRP3 Pathway,” Frontiers in Bioengineering and Biotechnology 8 (2020): 615850. 33520966 10.3389/fbioe.2020.615850 PMC7844314

[344] X. H. Sun , X. Wang , Y. Zhang , and J. Hui , “Exosomes of Bone‐marrow Stromal Cells Inhibit Cardiomyocyte Apoptosis Under Ischemic and Hypoxic Conditions via miR‐486‐5p Targeting the PTEN/PI3K/AKT Signaling Pathway,” Thrombosis Research 177 (2019): 23–32. 30844685 10.1016/j.thromres.2019.02.002

[345] K. S. Li , Y. Bai , J. Li , et al., “LncRNA HCP5 in hBMSC‐derived Exosomes Alleviates Myocardial Ischemia Reperfusion Injury by Sponging miR‐497 to Activate IGF1/PI3K/AKT Pathway,” International Journal of Cardiology 342 (2021): 72–81. 34311013 10.1016/j.ijcard.2021.07.042

[346] G. Chen , M. Wang , Z. Ruan , L. Zhu , and C. Tang , “Mesenchymal Stem Cell‐derived Exosomal miR‐143‐3p Suppresses Myocardial Ischemia‐reperfusion Injury by Regulating Autophagy,” Life Sciences 280 (2021): 119742. 34166712 10.1016/j.lfs.2021.119742

[347] X. Cui , Z. He , Z. Liang , Z. Chen , H. Wang , and J. Zhang , “Exosomes from Adipose‐derived Mesenchymal Stem Cells Protect the Myocardium against Ischemia/Reperfusion Injury through Wnt/Beta‐Catenin Signaling Pathway,” Journal of Cardiovascular Pharmacology 70, no. 4 (2017): 225–231. 28582278 10.1097/FJC.0000000000000507 PMC5642342

[348] T. C. Lai , T. L. Lee , Y. C. Chang , et al., “MicroRNA‐221/222 Mediates ADSC‐Exosome‐Induced Cardioprotection against Ischemia/Reperfusion by Targeting PUMA and ETS‐1,” Frontiers in Cell and Developmental Biology 8 (2020): 569150. 33344446 10.3389/fcell.2020.569150 PMC7744807

[349] Z. Liu , Y. Xu , Y. Wan , J. Gao , Y. Chu , and J. Li , “Exosomes From Adipose‐derived Mesenchymal Stem Cells Prevent Cardiomyocyte Apoptosis Induced by Oxidative Stress,” Cell Death Discovery 5 (2019): 79. 30911413 10.1038/s41420-019-0159-5 PMC6425027

[350] M. Khan , E. Nickoloff , T. Abramova , et al., “Embryonic Stem Cell‐derived Exosomes Promote Endogenous Repair Mechanisms and Enhance Cardiac Function Following Myocardial Infarction,” Circulation Research 117, no. 1 (2015): 52–64. 25904597 10.1161/CIRCRESAHA.117.305990 PMC4482130

[351] Y. Wang , L. Zhang , Y. Li , et al., “Exosomes/Microvesicles From Induced Pluripotent Stem Cells Deliver Cardioprotective miRNAs and Prevent Cardiomyocyte Apoptosis in the Ischemic Myocardium,” International Journal of Cardiology 192 (2015): 61–69. 26000464 10.1016/j.ijcard.2015.05.020 PMC4469495

[352] L. Zhang , Q. Wang , H. Su , and J. Cheng , “Exosomes From Adipose Derived Mesenchymal Stem Cells Alleviate Diabetic Osteoporosis in Rats Through Suppressing NLRP3 Inflammasome Activation in Osteoclasts,” Journal of Bioscience and Bioengineering 131, no. 6 (2021): 671–678. 33849774 10.1016/j.jbiosc.2021.02.007

[353] S. Cosenza , M. Ruiz , K. Toupet , C. Jorgensen , and D. Noel , “Mesenchymal Stem Cells Derived Exosomes and Microparticles Protect Cartilage and Bone From Degradation in Osteoarthritis,” Scientific Reports 7, no. 1 (2017): 16214. 29176667 10.1038/s41598-017-15376-8 PMC5701135

[354] L. Zheng , Y. Wang , P. Qiu , et al., “Primary Chondrocyte Exosomes Mediate Osteoarthritis Progression by Regulating Mitochondrion and Immune Reactivity,” Nanomedicine (London) 14, no. 24 (2019): 3193–3212. 10.2217/nnm-2018-0498 31855117

[355] W. Li , L. Li , R. Cui , X. Chen , H. Hu , and Y. Qiu , “Bone Marrow Mesenchymal Stem Cells Derived Exosomal Lnc TUG1 Promotes Bone Fracture Recovery via miR‐22‐5p/Anxa8 Axis,” Human Cell 36, no. 3 (2023): 1041–1053. 36952210 10.1007/s13577-023-00881-y PMC10110643

[356] X. Yang , J. Yang , P. Lei , and T. Wen , “LncRNA MALAT1 Shuttled by Bone Marrow‐derived Mesenchymal Stem Cells‐secreted Exosomes Alleviates Osteoporosis Through Mediating microRNA‐34c/SATB2 Axis,” Aging (Albany NY) 11, no. 20 (2019): 8777–8791. 31659145 10.18632/aging.102264 PMC6834402

[357] R. Z. Yang , W. N. Xu , H. L. Zheng , et al., “Exosomes Derived From Vascular Endothelial Cells Antagonize Glucocorticoid‐induced Osteoporosis by Inhibiting Ferritinophagy With Resultant Limited Ferroptosis of Osteoblasts,” Journal of Cellular Physiology 236, no. 9 (2021): 6691–6705. 33590921 10.1002/jcp.30331

[358] H. Hu , D. Wang , L. Li , H. Yin , G. He , and Y. Zhang , “Role of microRNA‐335 Carried by Bone Marrow Mesenchymal Stem Cells‐derived Extracellular Vesicles in Bone Fracture Recovery,” Cell Death & Disease 12, no. 2 (2021): 156. 33542183 10.1038/s41419-021-03430-3 PMC7862274

[359] T. Manzoor , A. Saleem , N. Farooq , et al., “Extracellular Vesicles Derived From Mesenchymal Stem Cells—a Novel Therapeutic Tool in Infectious Diseases,” Inflammation and Regeneration 43, no. 1 (2023): 17. 36849892 10.1186/s41232-023-00266-6 PMC9970864

[360] J.‐Y. Ding , M.‐J. Chen , L.‐F. Wu , et al., “Mesenchymal Stem Cell‐derived Extracellular Vesicles in Skin Wound Healing: Roles, Opportunities and Challenges,” Military Medical Research 10, no. 1 (2023): 36. 37587531 10.1186/s40779-023-00472-w PMC10433599

[361] J. Wang , S. Yuan , Y. Tu , Z. Lv , H. Cheng , and X. Ding , “Extracellular Vesicles in Skin Health, Diseases, and Aging,” Interdisciplinary Medicine 2, no. 3 (2024): e20240011.

[362] J. Y. Hong , T. R. Kwon , J. H. Kim , B. C. Lee , and B. J. Kim , “Prospective, Preclinical Comparison of the Performance Between Radiofrequency Microneedling and Microneedling Alone in Reversing Photoaged Skin,” Journal of Cosmetic Dermatology 19, no. 5 (2020): 1105–1109. 31490628 10.1111/jocd.13116

[363] W. Gao , L.‐M. Yuan , Y. Zhang , et al., “miR‐1246‐overexpressing Exosomes Suppress UVB‐induced Photoaging via Regulation of TGF‐β/Smad and Attenuation of MAPK/AP‐1 Pathway,” Photochemical & Photobiological Sciences 22, no. 1 (2023): 135–146. 36114328 10.1007/s43630-022-00304-1

[364] M. Deng , T. Z. Yu , D. Li , et al., “Human Umbilical Cord Mesenchymal Stem Cell‐derived and Dermal Fibroblast‐derived Extracellular Vesicles Protect Dermal Fibroblasts From Ultraviolet Radiation‐induced Photoaging in Vitro,” Photochemical & Photobiological Sciences 19 (2020): 406–414. 32125331 10.1039/c9pp00421a

[365] T. Wang , Z. Jian , A. Baskys , et al., “MSC‐derived Exosomes Protect Against Oxidative Stress‐induced Skin Injury via Adaptive Regulation of the NRF2 Defense System,” Biomaterials 257 (2020): 120264. 32791387 10.1016/j.biomaterials.2020.120264

[366] W. Zhang , X. Bai , B. Zhao , et al., “Cell‐free Therapy Based on Adipose Tissue Stem Cell‐derived Exosomes Promotes Wound Healing via the PI3K/Akt Signaling Pathway,” Experimental Cell Research 370, no. 2 (2018): 333–342. 29964051 10.1016/j.yexcr.2018.06.035

[367] J. S. Choi , W. L. Cho , Y. J. Choi , et al., “Functional Recovery in Photo‐damaged human Dermal Fibroblasts by human Adipose‐derived Stem Cell Extracellular Vesicles,” Journal of Extracellular Vesicles 8, no. 1 (2019): 1565885. 30719241 10.1080/20013078.2019.1565885 PMC6346706

[368] Y. Shimizu , E. H. Ntege , Y. Inoue , N. Matsuura , H. Sunami , and Y. Sowa , “Optimizing Mesenchymal Stem Cell Extracellular Vesicles for Chronic Wound Healing: Bioengineering, Standardization, and Safety,” Regenerative Therapy 26 (2024): 260–274. 38978963 10.1016/j.reth.2024.06.001 PMC11228664

[369] J. Kim , E. H. Kim , H. Lee , and J. H. Sung , “Bang OYJIJoMS. Clinical‐scale Mesenchymal Stem Cell‐derived Extracellular Vesicle Therapy for Wound Healing,” International Journal of Molecular Sciences 24, no. 5 (2023): 4273. 36901703 10.3390/ijms24054273 PMC10001880

[370] X. He , Z. Dong , Y. Cao , et al., “MSC‐derived Exosome Promotes M2 Polarization and Enhances Cutaneous Wound Healing,” Stem Cells International 2019, no. 1 (2019): 7132708. 31582986 10.1155/2019/7132708 PMC6754952

[371] L. Pi , L. Yang , B.‐R. Fang , X.‐X. Meng , L. J. M. Qian , and C. Biochemistry , “Exosomal microRNA‐125a‐3p From human Adipose‐derived Mesenchymal Stem Cells Promotes Angiogenesis of Wound Healing Through Inhibiting PTEN,” Molecular and Cellular Biochemistry 477, no. 1 (2022): 115–127. 34581942 10.1007/s11010-021-04251-w

[372] L. Zhang , P. Ouyang , G. He , et al., “Exosomes From microRNA‐126 Overexpressing Mesenchymal Stem Cells Promote Angiogenesis by Targeting the PIK3R2‐mediated PI3K/Akt Signalling Pathway,” Journal of Cellular and Molecular Medicine 25, no. 4 (2021): 2148–2162. 33350092 10.1111/jcmm.16192 PMC7882955

[373] Y. Li , J. Zhang , J. Shi , et al., “Exosomes Derived From human Adipose Mesenchymal Stem Cells Attenuate Hypertrophic Scar Fibrosis by miR‐192‐5p/IL‐17RA/Smad Axis,” Stem Cell Research & Therapy 12 (2021): 1–16. 33789737 10.1186/s13287-021-02290-0 PMC8010995

[374] J. Yang , M. Xiao , K. Ma , et al., “Therapeutic Effects of Mesenchymal Stem Cells and Their Derivatives in Common Skin Inflammatory Diseases: Atopic Dermatitis and Psoriasis,” Frontiers in Immunology 14 (2023): 1092668. 36891306 10.3389/fimmu.2023.1092668 PMC9986293

[375] J. Kim , S. K. Lee , M. Jung , et al., “Extracellular Vesicles From IFN‐γ‐primed Mesenchymal Stem Cells Repress Atopic Dermatitis in Mice,” Journal of Nanobiotechnology 20, no. 1 (2022): 526. 36496385 10.1186/s12951-022-01728-8 PMC9741801

[376] W. Zhang , J. Lin , P. Shi , et al., “Small Extracellular Vesicles Derived From MSCs Have Immunomodulatory Effects to Enhance Delivery of ASO‐210 for Psoriasis Treatment,” Frontiers in Cell and Developmental Biology 10 (2022): 842813. 35359454 10.3389/fcell.2022.842813 PMC8960430

[377] M. Qu , Q. Lin , L. Huang , et al., “Dopamine‐loaded Blood Exosomes Targeted to Brain for Better Treatment of Parkinson's Disease,” Journal of Controlled Release 287 (2018): 156–166. 30165139 10.1016/j.jconrel.2018.08.035

[378] M. J. Haney , N. L. Klyachko , Y. Zhao , et al., “Exosomes as Drug Delivery Vehicles for Parkinson's Disease Therapy,” Journal of Controlled Release 207 (2015): 18–30. 25836593 10.1016/j.jconrel.2015.03.033 PMC4430381

[379] Q. Chen , Y. Liu , X. Ding , et al., “Bone Marrow Mesenchymal Stem Cell‐secreted Exosomes Carrying microRNA‐125b Protect Against Myocardial Ischemia Reperfusion Injury via Targeting SIRT7,” Molecular and Cellular Biochemistry 465, no. 1‐2 (2020): 103–114. 31858380 10.1007/s11010-019-03671-z PMC6955239

[380] L. Jiang , Y. Zhang , T. Liu , et al., “Exosomes Derived From TSG‐6 Modified Mesenchymal Stromal Cells Attenuate Scar Formation During Wound Healing,” Biochimie 177 (2020): 40–49. 32800897 10.1016/j.biochi.2020.08.003

[381] S. C. Tao , T. Yuan , Y. L. Zhang , W. J. Yin , S. C. Guo , and C. Q. Zhang , “Exosomes Derived From miR‐140‐5p‐overexpressing human Synovial Mesenchymal Stem Cells Enhance Cartilage Tissue Regeneration and Prevent Osteoarthritis of the Knee in a Rat Model,” Theranostics 7, no. 1 (2017): 180–195. 28042326 10.7150/thno.17133 PMC5196895

[382] Z. Erana‐Perez , M. Igartua , E. Santos‐Vizcaino , and R. M. Hernandez , “Genetically Engineered Loaded Extracellular Vesicles for Drug Delivery,” Trends in Pharmacological Sciences 45, no. 4 (2024): 350–365. 38508958 10.1016/j.tips.2024.02.006

[383] Q. Liu , D. Li , X. Pan , and Y. Liang , “Targeted Therapy Using Engineered Extracellular Vesicles: Principles and Strategies for Membrane Modification,” Journal of Nanobiotechnology 21, no. 1 (2023): 334. 37717008 10.1186/s12951-023-02081-0 PMC10505332

[384] L. Alvarez‐Erviti , Y. Seow , H. Yin , C. Betts , S. Lakhal , and M. J. A. Wood , “Delivery of siRNA to the Mouse Brain by Systemic Injection of Targeted Exosomes,” Nature Biotechnology 29, no. 4 (2011): 341–345. 10.1038/nbt.1807 21423189

[385] M. Kim , G. Kim , D. W. Hwang , and M. Lee , “Delivery of High Mobility Group Box‐1 siRNA Using Brain‐Targeting Exosomes for Ischemic Stroke Therapy,” Journal of Biomedical Nanotechnology 15, no. 12 (2019): 2401–2412. 31748020 10.1166/jbn.2019.2866

[386] J. Yang , X. Zhang , X. Chen , L. Wang , and G. Yang , “Exosome Mediated Delivery of miR‐124 Promotes Neurogenesis After Ischemia,” Molecular Therapy Nucleic Acids 7 (2017): 278–287. 28624203 10.1016/j.omtn.2017.04.010 PMC5415550

[387] J. Yang , S. Wu , L. Hou , et al., “Therapeutic Effects of Simultaneous Delivery of Nerve Growth Factor mRNA and Protein via Exosomes on Cerebral Ischemia,” Molecular Therapy Nucleic Acids 21 (2020): 512–522. 32682291 10.1016/j.omtn.2020.06.013 PMC7365960

[388] L. Yang , B. Han , Z. Zhang , et al., “Extracellular Vesicle‐Mediated Delivery of Circular RNA SCMH1 Promotes Functional Recovery in Rodent and Nonhuman Primate Ischemic Stroke Models,” Circulation 142, no. 6 (2020): 556–574. 32441115 10.1161/CIRCULATIONAHA.120.045765

[389] R. Kojima , D. Bojar , G. Rizzi , et al., “Designer Exosomes Produced by Implanted Cells Intracerebrally Deliver Therapeutic Cargo for Parkinson's Disease Treatment,” Nature Communications 9, no. 1 (2018): 1305. 10.1038/s41467-018-03733-8 PMC5880805 29610454

[390] X. Ren , Y. Zhao , F. Xue , et al., “Exosomal DNA Aptamer Targeting α‐Synuclein Aggregates Reduced Neuropathological Deficits in a Mouse Parkinson's Disease Model,” Molecular Therapy Nucleic Acids 17 (2019): 726–740. 31437653 10.1016/j.omtn.2019.07.008 PMC6709346

[391] Y. Liang , X. Xu , X. Li , et al., “Chondrocyte‐Targeted MicroRNA Delivery by Engineered Exosomes Toward a Cell‐Free Osteoarthritis Therapy,” ACS Applied Materials and Interfaces Journal 12, no. 33 (2020): 36938–36947. 10.1021/acsami.0c10458 32814390

[392] X. Xu , Y. Liang , X. Li , et al., “Exosome‐mediated Delivery of Kartogenin for Chondrogenesis of Synovial Fluid‐derived Mesenchymal Stem Cells and Cartilage Regeneration,” Biomaterials 269 (2021): 120539. 33243424 10.1016/j.biomaterials.2020.120539

[393] Y. Lin , M. Yan , Z. Bai , et al., “Huc‐MSC‐derived Exosomes Modified With the Targeting Peptide of aHSCs for Liver Fibrosis Therapy,” Journal of Nanobiotechnology 20, no. 1 (2022): 432. 36183106 10.1186/s12951-022-01636-x PMC9526331

[394] R. H. Fang , C. M. Hu , K. N. Chen , et al., “Lipid‐insertion Enables Targeting Functionalization of Erythrocyte Membrane‐cloaked Nanoparticles,” Nanoscale 5, no. 19 (2013): 8884–8888. 23907698 10.1039/c3nr03064d PMC3831007

[395] G.‐H. Cui , H.‐D. Guo , H. Li , et al., “RVG‐modified Exosomes Derived From Mesenchymal Stem Cells Rescue Memory Deficits by Regulating Inflammatory Responses in a Mouse Model of Alzheimer's Disease,” Immunity & Ageing 16, no. 1 (2019): 10. 31114624 10.1186/s12979-019-0150-2 PMC6515654

[396] J. Che , C. I. Okeke , Z. B. Hu , and J. Xu , “DSPE‐PEG: A Distinctive Component in Drug Delivery System,” Current Pharmaceutical Design 21, no. 12 (2015): 1598–1605. 25594410 10.2174/1381612821666150115144003

[397] D. G. You , B. H. Oh , V. Q. Nguyen , et al., “Vitamin A‐coupled Stem Cell‐derived Extracellular Vesicles Regulate the Fibrotic Cascade by Targeting Activated Hepatic Stellate Cells in Vivo,” Journal of Controlled Release 336 (2021): 285–295. 34174353 10.1016/j.jconrel.2021.06.031

[398] T. Tian , H. X. Zhang , C. P. He , et al., “Surface Functionalized Exosomes as Targeted Drug Delivery Vehicles for Cerebral Ischemia Therapy,” Biomaterials 150 (2018): 137–149. 29040874 10.1016/j.biomaterials.2017.10.012

[399] H. Zhang , J. Wu , J. Wu , et al., “Exosome‐mediated Targeted Delivery of miR‐210 for Angiogenic Therapy After Cerebral Ischemia in Mice,” Journal of Nanobiotechnology 17, no. 1 (2019): 29. 30782171 10.1186/s12951-019-0461-7 PMC6379944

[400] A. H. Berggreen , J. L. Petersen , L. Lin , K. Benabdellah , and Y. Luo , “CRISPR Delivery With Extracellular Vesicles: Promises and Challenges,” Journal of Extracellular Biology 2, no. 9 (2023): e111. 38938376 10.1002/jex2.111 PMC11080907

[401] L. You , R. Tong , M. Li , Y. Liu , J. Xue , and Y. Lu , “Advancements and Obstacles of CRISPR‐Cas9 Technology in Translational Research,” Molecular Therapy Methods & Clinical Development 13 (2019): 359–370. 30989086 10.1016/j.omtm.2019.02.008 PMC6447755

[402] B. Yan and Y. Liang , “New Therapeutics for Extracellular Vesicles: Delivering CRISPR for Cancer Treatment,” International Journal of Molecular Sciences 23, no. 24 (2022): 15758. 36555398 10.3390/ijms232415758 PMC9779094

[403] D. Lainšček , L. Kadunc , M. M. Keber , I. H. Bratkovič , R. Romih , and R. Jerala , “Delivery of an Artificial Transcription Regulator dCas9‐VPR by Extracellular Vesicles for Therapeutic Gene Activation,” ACS Synthetic Biology 7, no. 12 (2018): 2715–2725. 30513193 10.1021/acssynbio.8b00192

[404] T. Wan , J. Zhong , Q. Pan , T. Zhou , Y. Ping , and X. Liu , “Exosome‐mediated Delivery of Cas9 ribonucleoprotein Complexes for Tissue‐specific Gene Therapy of Liver Diseases,” Science Advances 8, no. 37 (2022): eabp9435. 36103526 10.1126/sciadv.abp9435 PMC9473578

[405] N. Majeau , A. Fortin‐Archambault , C. Gérard , J. Rousseau , P. Yaméogo , and J. P. Tremblay , “Serum Extracellular Vesicles for Delivery of CRISPR‐CAS9 Ribonucleoproteins to Modify the Dystrophin Gene,” Molecular Therapy 30, no. 7 (2022): 2429–2442. 35619556 10.1016/j.ymthe.2022.05.023 PMC9263317

[406] B. Wang , M. Chang , R. Zhang , et al., “Spinal Cord Injury Target‐immunotherapy With TNF‐α Autoregulated and Feedback‐controlled human Umbilical Cord Mesenchymal Stem Cell Derived Exosomes Remodelled by CRISPR/Cas9 Plasmid,” Biomaterials Advances 133 (2022): 112624. 35525736 10.1016/j.msec.2021.112624

[407] P. Gee , M. S. Y. Lung , Y. Okuzaki , et al., “Extracellular Nanovesicles for Packaging of CRISPR‐Cas9 Protein and sgRNA to Induce Therapeutic Exon Skipping,” Nature Communications 11, no. 1 (2020): 1334. 10.1038/s41467-020-14957-y PMC7070030 32170079

[408] X. Osteikoetxea , A. Silva , E. Lázaro‐Ibáñez , et al., “Engineered Cas9 Extracellular Vesicles as a Novel Gene Editing Tool,” Journal of Extracellular Vesicles 11, no. 5 (2022): e12225. 35585651 10.1002/jev2.12225 PMC9117459

[409] Y. Yuana , A. Sturk , and R. Nieuwland , “Extracellular Vesicles in Physiological and Pathological Conditions,” Blood Reviews 27, no. 1 (2013): 31–39. 23261067 10.1016/j.blre.2012.12.002

[410] M. A. Kumar , S. K. Baba , H. Q. Sadida , et al., “Extracellular Vesicles as Tools and Targets in Therapy for Diseases,” Signal Transduction and Targeted Therapy 9, no. 1 (2024): 27. 38311623 10.1038/s41392-024-01735-1 PMC10838959

[411] M. Catalano and L. O'Driscoll , “Inhibiting Extracellular Vesicles Formation and Release: A Review of EV Inhibitors,” Journal of Extracellular Vesicles 9, no. 1 (2020): 1703244. 32002167 10.1080/20013078.2019.1703244 PMC6968539

[412] K. Trajkovic , C. Hsu , S. Chiantia , et al., “Ceramide Triggers Budding of Exosome Vesicles Into Multivesicular Endosomes,” Science 319, no. 5867 (2008): 1244–1247. 18309083 10.1126/science.1153124

[413] M. B. Dinkins , S. Dasgupta , G. Wang , G. Zhu , and E. Bieberich , “Exosome Reduction in Vivo Is Associated With Lower Amyloid Plaque Load in the 5XFAD Mouse Model of Alzheimer's Disease,” Neurobiology of Aging 35, no. 8 (2014): 1792–1800. 24650793 10.1016/j.neurobiolaging.2014.02.012 PMC4035236

[414] K. Essandoh , L. Yang , X. Wang , et al., “Blockade of Exosome Generation With GW4869 Dampens the Sepsis‐induced Inflammation and Cardiac Dysfunction,” Biochimica Et Biophysica Acta 1852, no. 11 (2015): 2362–2371. 26300484 10.1016/j.bbadis.2015.08.010 PMC4581992

[415] L. Lyu , H. Wang , B. Li , et al., “A Critical Role of Cardiac Fibroblast‐derived Exosomes in Activating Renin Angiotensin System in Cardiomyocytes,” Journal of Molecular and Cellular Cardiology 89, no. Pt B (2015): 268–279. 26497614 10.1016/j.yjmcc.2015.10.022 PMC4988239

[416] H. Dai , W. Zheng , J. Luo , et al., “Inhibiting Uptake of Extracellular Vesicles Derived From Senescent Bone Marrow Mesenchymal Stem Cells by Muscle Satellite Cells Attenuates Sarcopenia,” Journal of Orthopaedic Translation 35 (2022): 23–36. 35846725 10.1016/j.jot.2022.06.002 PMC9260455

[417] E. Eggenhofer , V. Benseler , A. Kroemer , et al., “Mesenchymal Stem Cells Are Short‐lived and Do Not Migrate Beyond the Lungs After Intravenous Infusion,” Frontiers in Immunology 3 (2012): 297. 23056000 10.3389/fimmu.2012.00297 PMC3458305

[418] M. Sanchez‐Diaz , M. I. Quinones‐Vico , R. Sanabria de la Torre , et al., “Biodistribution of Mesenchymal Stromal Cells After Administration in Animal Models and Humans: A Systematic Review,” Journal of Clinical Medicine 10, no. 13 (2021): 2925. 34210026 10.3390/jcm10132925 PMC8268414

[419] B. Zeng , Y. Li , J. Xia , et al., “Micro Trojan Horses: Engineering Extracellular Vesicles Crossing Biological Barriers for Drug Delivery,” Bioengineering & Translational Medicine 9, no. 2 (2024): e10623. 38435823 10.1002/btm2.10623 PMC10905561

[420] D. H. Kim , V. K. Kothandan , H. W. Kim , et al., “Noninvasive Assessment of Exosome Pharmacokinetics in Vivo: A Review,” Pharmaceutics 11, no. 12 (2019): 649. 31817039 10.3390/pharmaceutics11120649 PMC6956244

[421] M. Van Delen , J. Derdelinckx , K. Wouters , I. Nelissen , and N. Cools , “A Systematic Review and Meta‐analysis of Clinical Trials Assessing Safety and Efficacy of human Extracellular Vesicle‐based Therapy,” Journal of Extracellular Vesicles 13, no. 7 (2024): e12458. 38958077 10.1002/jev2.12458 PMC11220457

[422] H. M. Ramos‐Zaldívar , I. Polakovicova , E. Salas‐Huenuleo , et al., “Extracellular Vesicles Through the Blood‐brain Barrier: A Review,” Fluids and Barriers of the CNS 19, no. 1 (2022): 60. 35879759 10.1186/s12987-022-00359-3 PMC9310691

[423] W. A. Banks , P. Sharma , K. M. Bullock , K. M. Hansen , N. Ludwig , and T. L. Whiteside , “Transport of Extracellular Vesicles Across the Blood‐Brain Barrier: Brain Pharmacokinetics and Effects of Inflammation,” International Journal of Molecular Sciences 21, no. 12 (2020): 4407. 32575812 10.3390/ijms21124407 PMC7352415

[424] I. K. Herrmann , M. J. A. Wood , and G. Fuhrmann , “Extracellular Vesicles as a next‐generation Drug Delivery Platform,” Nature Nanotechnology 16, no. 7 (2021): 748–759. 10.1038/s41565-021-00931-2 34211166

[425] D. T. Harris , “Long‐term Frozen Storage of Stem Cells: Challenges and Solutions,” Journal of Biorepository Science for Applied Medicine (2016): 9–20.

[426] S. Bruno , C. Pasquino , M. B. Herrera Sanchez , et al., “HLSC‐Derived Extracellular Vesicles Attenuate Liver Fibrosis and Inflammation in a Murine Model of Non‐alcoholic Steatohepatitis,” Molecular Therapy 28, no. 2 (2020): 479–489. 31757759 10.1016/j.ymthe.2019.10.016 PMC7001005

[427] H. S. Han , H. Lee , D. You , et al., “Human Adipose Stem Cell‐derived Extracellular Nanovesicles for Treatment of Chronic Liver Fibrosis,” Journal of Controlled Release 320 (2020): 328–336. 31981658 10.1016/j.jconrel.2020.01.042

[428] Z. Yan , T. Zhang , Y. Wang , S. Xiao , and J. Gao , “Extracellular Vesicle Biopotentiated Hydrogels for Diabetic Wound Healing: The Art of Living Nanomaterials Combined With Soft Scaffolds,” Materials Today Bio 23 (2023): 100810. 10.1016/j.mtbio.2023.100810 PMC10550777 37810755

[429] X. Geng , Y. Qi , X. Liu , Y. Shi , H. Li , and L. Zhao , “A Multifunctional Antibacterial and Self‐healing Hydrogel Laden With Bone Marrow Mesenchymal Stem Cell‐derived Exosomes for Accelerating Diabetic Wound Healing,” Biomaterials Advances 133 (2022): 112613. 35527135 10.1016/j.msec.2021.112613

[430] P. C. Dinh , D. Paudel , H. Brochu , et al., “Inhalation of Lung Spheroid Cell Secretome and Exosomes Promotes Lung Repair in Pulmonary Fibrosis,” Nature Communications 11, no. 1 (2020): 1064. 10.1038/s41467-020-14344-7 PMC7048814 32111836

[431] K. Zhang and K. Cheng , “Stem Cell‐derived Exosome versus Stem Cell Therapy,” Nature Reviews Bioengineering 1, no. 9 (2023): 608–609. 10.1038/s44222-023-00064-2 PMC10092910 37359776

[432] S. Hua , M. B. C. de Matos , J. M. Metselaar , and G. Storm , “Current Trends and Challenges in the Clinical Translation of Nanoparticulate Nanomedicines: Pathways for Translational Development and Commercialization,” Frontiers in Pharmacology 9 (2018): 790. 30065653 10.3389/fphar.2018.00790 PMC6056679

[433] K. Cheng and R. Kalluri , “Guidelines for Clinical Translation and Commercialization of Extracellular Vesicles and Exosomes Based Therapeutics,” Extracellular Vesicle 2 (2023): 100029.

[434] T. Driedonks , L. Jiang , B. Carlson , et al., “Pharmacokinetics and Biodistribution of Extracellular Vesicles Administered Intravenously and Intranasally to Macaca nemestrina,” Journal of Extracellular Biology 1, no. 10 (2022): e59. 36591537 10.1002/jex2.59 PMC9799283

[435] V. D. Bui , J. Jeon , V. H. Duong , et al., “Chondroitin Sulfate‐based Microneedles for Transdermal Delivery of Stem Cell‐derived Extracellular Vesicles to Treat Rheumatoid Arthritis,” Journal of Controlled Release 375 (2024): 105–115. 39218160 10.1016/j.jconrel.2024.08.050

[436] V. D. Bui , S. Son , W. Xavier , et al., “Dissolving Microneedles for Long‐term Storage and Transdermal Delivery of Extracellular Vesicles,” Biomaterials 287 (2022): 121644. 35772350 10.1016/j.biomaterials.2022.121644

[437] C. K. Wang , T. H. Tsai , and C. H. Lee , “Regulation of Exosomes as Biologic Medicines: Regulatory Challenges Faced in Exosome Development and Manufacturing Processes,” Clinical and Translational Science 17, no. 8 (2024): e13904. 39115257 10.1111/cts.13904 PMC11307316

[438] S. Fripont , C. Marneffe , M. Marino , M. Y. Rincon , and M. G. Holt , “Production, Purification, and Quality Control for Adeno‐associated Virus‐based Vectors,” Journal of Visualized Experiments: JoVE no. 143 (2019). 10.3791/58960 30774140

[439] S. J. Shepherd , X. Han , A. J. Mukalel , et al., “Throughput‐scalable Manufacturing of SARS‐CoV‐2 mRNA Lipid Nanoparticle Vaccines,” Proceedings of the National Academy of Sciences of the United States of America 120, no. 33 (2023): e2303567120. 37556502 10.1073/pnas.2303567120 PMC10438381

[440] E. E. Kepplinger , “FDA's Expedited Approval Mechanisms for New Drug Products,” Biotechnology Law Report 34, no. 1 (2015): 15–37. 25713472 10.1089/blr.2015.9999 PMC4326266

[441] P. Pakter , “Rare Disease Care in Europe–Gaping Unmet Needs,” Rare 2 (2024): 100018.

[442] A. E. Eldeeb , S. Salah , and N. A. Elkasabgy , “Biomaterials for Tissue Engineering Applications and Current Updates in the Field: A Comprehensive Review,” Aaps Pharmscitech [Electronic Resource] 23, no. 7 (2022): 267. 36163568 10.1208/s12249-022-02419-1 PMC9512992

[443] M. J. Evans and M. H. Kaufman , “Establishment in Culture of Pluripotential Cells From Mouse Embryos,” Nature 292, no. 5819 (1981): 154–156. 7242681 10.1038/292154a0

[444] L. V. Thomas . Polymeric Biomaterials in Tissue Engineering: Retrospect and Prospects. In: B. Bhaskar , P. Sreenivasa Rao , N. Kasoju , V. Nagarjuna , R. R. Baadhe , eds. “Biomaterials in Tissue Engineering and Regenerative Medicine: From Basic Concepts to State of the Art Approaches” (Singapore: Springer, 2021): 89–118.

[445] G. G. Gallico 3rd , N. E. O'Connor , C. C. Compton , O. Kehinde , and H. Green , “Permanent Coverage of Large Burn Wounds With Autologous Cultured human Epithelium,” New England Journal of Medicine 311, no. 7 (1984): 448–451. 6379456 10.1056/NEJM198408163110706

[446] S. V. Murphy and A. Atala , “3D bioprinting of Tissues and Organs,” Nature Biotechnology 32, no. 8 (2014): 773–785. 10.1038/nbt.2958 25093879

[447] P. S. Gungor‐Ozkerim , I. Inci , Y. S. Zhang , A. Khademhosseini , and M. R. Dokmeci , “Bioinks for 3D Bioprinting: An Overview,” Biomaterials Science 6, no. 5 (2018): 915–946. 29492503 10.1039/c7bm00765e PMC6439477

[448] H.‐W. Kang , S. J. Lee , I. K. Ko , C. Kengla , J. J. Yoo , and A. Atala , “A 3D Bioprinting System to Produce human‐scale Tissue Constructs With Structural Integrity,” Nature Biotechnology 34, no. 3 (2016): 312–319. 10.1038/nbt.3413 26878319

[449] S. M. Riha , M. Maarof , and M. B. Fauzi , “Synergistic Effect of Biomaterial and Stem Cell for Skin Tissue Engineering in Cutaneous Wound Healing: A Concise Review,” Polymers (Basel) 13, no. 10 (2021): 1546. 34065898 10.3390/polym13101546 PMC8150744

[450] T. Kitsuka , F. Takahashi , J. Reinhardt , et al., “Advances in Cardiac Tissue Engineering,” Bioengineering 9, no. 11 (2022): 696. 36421097 10.3390/bioengineering9110696 PMC9687338

[451] R. Ramli , M. Reddy , and N. Oliver , “Artificial Pancreas: Current Progress and Future Outlook in the Treatment of Type 1 Diabetes,” Drugs 79, no. 10 (2019): 1089–1101. 31190305 10.1007/s40265-019-01149-2

[452] M. A. Lancaster and J. A. Knoblich , “Generation of Cerebral Organoids From human Pluripotent Stem Cells,” Nature Protocols 9, no. 10 (2014): 2329–2340. 25188634 10.1038/nprot.2014.158 PMC4160653

[453] H. Clevers , “Modeling Development and Disease With Organoids,” Cell 165, no. 7 (2016): 1586–1597. 27315476 10.1016/j.cell.2016.05.082

[454] Y. Kim , H. Ko , I. K. Kwon , and K. Shin , “Extracellular Matrix Revisited: Roles in Tissue Engineering,” International Neurourology Journal 20, no. Suppl 1 (2016): S23–S29. 27230457 10.5213/inj.1632600.318 PMC4895908

[455] M. M. Farag , “Recent Trends on Biomaterials for Tissue Regeneration Applications: Review,” Journal of Materials Science 58, no. 2 (2023): 527–558.

[456] N. Kasula , P. Dhokare , A. Bhattacharyya , and I. Noh , “Recent Advances in 3D Bioprinting of Polysaccharide‐based Bioinks for Fabrication of Bioengineered Tissues,” Molecular Systems Design & Engineering 9 (2024): 977–999.

[457] P. S. Gungor‐Ozkerim , I. Inci , Y. S. Zhang , A. Khademhosseini , and M. R. Dokmeci , “Bioinks for 3D Bioprinting: An Overview,” Biomaterials Science 6, no. 5 (2018): 915–946. 29492503 10.1039/c7bm00765e PMC6439477

[458] E. Isaeva , E. Beketov , N. Arguchinskaya , S. Ivanov , P. Shegay , and А. Kaprin , “Decellularized Extracellular Matrix for Tissue Engineering,” Современные технологии в медицине 14, no. 3 (2022): 57–68. 10.17691/stm2022.14.3.07 PMC10090917 37064810

[459] M. Zhe , X. Wu , P. Yu , et al., “Recent Advances in Decellularized Extracellular Matrix‐based Bioinks for 3D Bioprinting in Tissue Engineering,” Materials 16, no. 8 (2023): 3197. 37110034 10.3390/ma16083197 PMC10143913

[460] S. Dabrowska , A. Andrzejewska , M. Janowski , and B. Lukomska , “Immunomodulatory and Regenerative Effects of Mesenchymal Stem Cells and Extracellular Vesicles: Therapeutic Outlook for Inflammatory and Degenerative Diseases,” Frontiers in Immunology 11 (2021): 591065. 33613514 10.3389/fimmu.2020.591065 PMC7893976

[461] R. Ju , X. Gao , C. Zhang , W. Tang , W. Tian , and M. He , “Exogenous MSC Based Tissue Regeneration: A Review of Immuno‐protection Strategies From Biomaterial Scaffolds,” Journal of Materials Chemistry B 12, no. 36 (2024): 8868–8882. 39171946 10.1039/d4tb00778f

[462] D. Zujur , Z. Al‐Akashi , A. Nakamura , et al., “Enhanced Chondrogenic Differentiation of iPS Cell‐derived Mesenchymal Stem/Stromal Cells via Neural Crest Cell Induction for Hyaline Cartilage Repair. Original Research,” Frontiers in Cell and Developmental Biology 11 (2023): 1140717. 37234772 10.3389/fcell.2023.1140717 PMC10206169

[463] M. Dottori , W.‐J. Li , G. Minchiotti , A. Rosa , and F. Sangiuolo , “Editorial: Reviews in Induced Pluripotent Stem Cells,” Frontiers in Cell and Developmental Biology 11 (2023): 1197891. 37215079 10.3389/fcell.2023.1197891 PMC10193027

[464] Z. Liu , J. Zhou , H. Wang , M. Zhao , and C. Wang , “Current Status of Induced Pluripotent Stem Cells in Cardiac Tissue Regeneration and Engineering,” Regenerative Medicine Research 1, no. 1 (2013): 6. 25984325 10.1186/2050-490X-1-6 PMC4376510

[465] Y. Yang , B. Ma , J. Chen , et al., “Epigenetic Regulation and Factors That Influence the Effect of iPSCs‐derived Neural Stem/Progenitor Cells (NS/PCs) in the Treatment of Spinal Cord Injury,” Clinical Epigenetics 16, no. 1 (2024): 30. 38383473 10.1186/s13148-024-01639-5 PMC10880347

[466] S. G. Ozcebe , M. Tristan , and P. Zorlutuna , “Adult Human Heart ECM Improves Human iPSC‐CM Function via Mitochondrial and Metabolic Maturation,” BioRxiv (2023), 2023.10.31.565062. 10.1093/stmcls/sxaf005 PMC12080356 39862185

[467] S. Rashidi , G. Bagherpour , Z. Abbasi‐Malati , et al., “Endothelial Progenitor Cells for Fabrication of Engineered Vascular Units and Angiogenesis Induction,” Cell Proliferation 57, no. 9 (2024): e13716. 39051852 10.1111/cpr.13716 PMC11503262

[468] S. Ribeiro , A. Watigny , Y. Bayon , M. Biggs , and D. Zeugolis , “It Takes Two to Tango: Controlling Human Mesenchymal Stromal Cell Response via Substrate Stiffness and Surface Topography,” Advanced NanoBiomed Research 4 (2023): 2300042.

[469] Q. Zhang , Y. Hu , X. Long , et al., “Preparation and Application of Decellularized ECM‐Based Biological Scaffolds for Articular Cartilage Repair: A Review,” Frontiers in Bioengineering and Biotechnology 10 (2022): 908082. 35845417 10.3389/fbioe.2022.908082 PMC9280718

[470] Y.‐H. Kim , S. Vijayavenkataraman , and G. Cidonio , “Biomaterials and Scaffolds for Tissue Engineering and Regenerative Medicine,” BMC Methods 1, no. 1 (2024): 2.

[471] F. Han , Q. Meng , E. Xie , et al., “Engineered Biomimetic Micro/Nano‐materials for Tissue Regeneration,” Frontiers in Bioengineering and Biotechnology 11 (2023): 1205792. 37469449 10.3389/fbioe.2023.1205792 PMC10352664

[472] H.‐C. Yan , T.‐T. Yu , J. Li , et al., “The Delivery of Extracellular Vesicles Loaded in Biomaterial Scaffolds for Bone Regeneration,” Frontiers in Bioengineering and Biotechnology 8 (2020): 1015. 32974327 10.3389/fbioe.2020.01015 PMC7466762

[473] D. N. Tavakol , S. Fleischer , T. Falcucci , et al., “Emerging Trajectories for Next Generation Tissue Engineers,” ACS Biomaterials Science & Engineering 8, no. 11 (2022): 4598–4604. 34878769 10.1021/acsbiomaterials.1c01428 PMC9174348

[474] R. Langer , “Chemical and Biological Approaches to Regenerative Medicine and Tissue Engineering,” Molecular Frontiers Journal 03, no. 02 (2019): 122–128.

[475] L. E. Niklason , J. Gao , W. M. Abbott , et al., “Functional Arteries Grown in Vitro,” Science 284, no. 5413 (1999): 489–493. 10205057 10.1126/science.284.5413.489

[476] J. H. Lawson , M. H. Glickman , M. Ilzecki , et al., “Bioengineered human Acellular Vessels for Dialysis Access in Patients With End‐stage Renal Disease: Two Phase 2 Single‐arm Trials,” The Lancet 387, no. 10032 (2016): 2026–2034. 10.1016/S0140-6736(16)00557-2 PMC4915925 27203778

[477] J. R. Slotkin , C. D. Pritchard , B. Luque , et al., “Biodegradable Scaffolds Promote Tissue Remodeling and Functional Improvement in Non‐human Primates With Acute Spinal Cord Injury,” Biomaterials 123 (2017): 63–76. 28167393 10.1016/j.biomaterials.2017.01.024

[478] Y. D. Teng , E. B. Lavik , X. Qu , et al., “Functional Recovery Following Traumatic Spinal Cord Injury Mediated by a Unique Polymer Scaffold Seeded With Neural Stem Cells,” Proceedings of the National Academy of Sciences of the United States of America 99, no. 5 (2002): 3024–3029. 11867737 10.1073/pnas.052678899 PMC122466

[479] S. Sabetkish , P. Currie , and L. Meagher , “Recent Trends in 3D Bioprinting Technology for Skeletal Muscle Regeneration,” Acta Biomaterialia 181: 46–66. 38697381 10.1016/j.actbio.2024.04.038

[480] M. Asadian , K. V. Chan , M. Norouzi , et al., “Fabrication and Plasma Modification of Nanofibrous Tissue Engineering Scaffolds,” Nanomaterials 10, no. 1 (2020): 119. 31936372 10.3390/nano10010119 PMC7023287

[481] O. Omar , T. Engstrand , L. Kihlström Burenstam Linder , et al., “In Situ Bone Regeneration of Large Cranial Defects Using Synthetic Ceramic Implants With a Tailored Composition and Design,” Proceedings of the National Academy of Sciences of the United States of America 117, no. 43 (2020): 26660–26671. 33046631 10.1073/pnas.2007635117 PMC7604495

[482] X. Dou , X. Wei , G. Liu , et al., “Effect of Porous Tantalum on Promoting the Osteogenic Differentiation of Bone Marrow Mesenchymal Stem Cells in Vitro Through the MAPK/ERK Signal Pathway,” Journal of Orthopaedic Translation 19 (2019): 81–93. 31844616 10.1016/j.jot.2019.03.006 PMC6896724

[483] A. R. Edelmann , D. Patel , R. K. Allen , C. J. Gibson , A. M. Best , and S. Bencharit , “Retrospective Analysis of Porous Tantalum Trabecular Metal–enhanced Titanium Dental Implants,” The Journal of Prosthetic Dentistry 121, no. 3 (2019): 404–410. 30396711 10.1016/j.prosdent.2018.04.022

[484] F. Re , E. Borsani , R. Rezzani , L. Sartore , and D. Russo , “Bone Regeneration Using Mesenchymal Stromal Cells and Biocompatible Scaffolds: A Concise Review of the Current Clinical Trials,” Gels 9, no. 5 (2023): 389. 37232981 10.3390/gels9050389 PMC10217263

[485] S. Shakir , T. L. Hackett , and L. B. Mostaço‐Guidolin , “Bioengineering Lungs: An Overview of Current Methods, Requirements, and Challenges for Constructing Scaffolds,” Frontiers in Bioengineering and Biotechnology 10 (2022): 1011800. 36394026 10.3389/fbioe.2022.1011800 PMC9649450

[486] W. L. Ng , T. C. Ayi , Y. C. Liu , S. L. Sing , W. Y. Yeong , and B. H. Tan , “Fabrication and Characterization of 3D Bioprinted Triple‐layered Human Alveolar Lung Models,” International Journal of Bioprinting 7, no. 2 (2021): 332. 33997432 10.18063/ijb.v7i2.332 PMC8114097

[487] A. Neishabouri , A. Soltani Khaboushan , F. Daghigh , A.‐M. Kajbafzadeh , and M. Majidi Zolbin , “Decellularization in Tissue Engineering and Regenerative Medicine: Evaluation, Modification, and Application Methods,” Frontiers in Bioengineering and Biotechnology 10 (2022): 805299. 35547166 10.3389/fbioe.2022.805299 PMC9081537

[488] Y. H. Song , M. A. Maynes , N. Hlavac , et al., “Development of Novel Apoptosis‐assisted Lung Tissue Decellularization Methods,” Biomaterials Science 9, no. 9 (2021): 3485–3498. 33949462 10.1039/d1bm00032b

[489] S. Alsobaie , T. Alsobaie , A. Alshammary , and S. Mantalaris , “Differentiation of human Induced Pluripotent Stem Cells Into Functional Lung Alveolar Epithelial Cells in 3D Dynamic Culture,” Frontiers in Bioengineering and Biotechnology 11 (2023): 1173149. 37388774 10.3389/fbioe.2023.1173149 PMC10303808

[490] W. Wruck , N. Graffmann , L.‐S. Spitzhorn , and J. Adjaye , “Human Induced Pluripotent Stem Cell‐Derived Mesenchymal Stem Cells Acquire Rejuvenation and Reduced Heterogeneity,” Frontiers in Cell and Developmental Biology 9 (2021): 717772. 34604216 10.3389/fcell.2021.717772 PMC8481886

[491] J. Li , X. Chen , M. Hu , et al., “The Application of Composite Scaffold Materials Based on Decellularized Vascular Matrix in Tissue Engineering: A Review,” BioMedical Engineering OnLine 22, no. 1 (2023): 62. 37337190 10.1186/s12938-023-01120-z PMC10278309

[492] A. H. Nguyen , P. Marsh , L. Schmiess‐Heine , et al., “Cardiac Tissue Engineering: State‐of‐the‐art Methods and Outlook,” Journal of Biological Engineering 13, no. 1 (2019): 57. 31297148 10.1186/s13036-019-0185-0 PMC6599291

[493] M. M. Rana and H. De la Hoz Siegler , “Evolution of Hybrid Hydrogels: Next‐Generation Biomaterials for Drug Delivery and Tissue Engineering,” Gels 10, no. 4 (2024): 216. 38667635 10.3390/gels10040216 PMC11049329

[494] M. M. Peters , J. K. Brister , E. M. Tang , et al., “Self‐organizing Behaviors of Cardiovascular Cells on Synthetic Nanofiber Scaffolds,” APL Bioengineering 7, no. 4 (2023): 046114. 38046543 10.1063/5.0172423 PMC10693444

[495] W. LaBarge , S. Mattappally , R. Kannappan , et al., “Maturation of Three‐dimensional, hiPSC‐derived Cardiomyocyte Spheroids Utilizing Cyclic, Uniaxial Stretch and Electrical Stimulation,” PLoS One 14, no. 7 (2019): e0219442. 31276558 10.1371/journal.pone.0219442 PMC6611624

[496] K. Zheng , Y. Hao , C. Xia , et al., “Effects and Mechanisms of the Myocardial Microenvironment on Cardiomyocyte Proliferation and Regeneration,” Frontiers in Cell and Developmental Biology 12 (2024): 1429020. 39050889 10.3389/fcell.2024.1429020 PMC11266095

[497] Z. S. Razavi , M. Soltani , G. Mahmoudvand , et al., “Advancements in Tissue Engineering for Cardiovascular Health: A Biomedical Engineering Perspective,” Frontiers in Bioengineering and Biotechnology 12 (2024): 1385124. 38882638 10.3389/fbioe.2024.1385124 PMC11176440

[498] A. Akbarzadeh , S. Sobhani , A. Soltani Khaboushan , and A.‐M. Kajbafzadeh , “Whole‐Heart Tissue Engineering and Cardiac Patches: Challenges and Promises,” Bioengineering 10, no. 1 (2023): 106. 36671678 10.3390/bioengineering10010106 PMC9855348

[499] P. Li , J. Hu , J. Wang , J. Zhang , L. Wang , and C. Zhang , “The Role of Hydrogel in Cardiac Repair and Regeneration for Myocardial Infarction: Recent Advances and Future Perspectives,” Bioengineering 10, no. 2 (2023): 165. 36829659 10.3390/bioengineering10020165 PMC9952459

[500] B. Sanders , S. Loerakker , E. S. Fioretta , et al., “Improved Geometry of Decellularized Tissue Engineered Heart Valves to Prevent Leaflet Retraction,” Annals of Biomedical Engineering 44, no. 4 (2016): 1061–1071. 26183964 10.1007/s10439-015-1386-4 PMC4826662

[501] P. E. Dijkman , A. Driessen‐Mol , L. Frese , S. P. Hoerstrup , and F. P. T. Baaijens , “Decellularized Homologous Tissue‐engineered Heart Valves as off‐the‐shelf Alternatives to Xeno‐ and Homografts,” Biomaterials 33, no. 18 (2012): 4545–4554. 22465337 10.1016/j.biomaterials.2012.03.015

[502] P. Mohacsi and P. Leprince , “The CARMAT Total Artificial Heart,” European Journal of Cardio‐Thoracic Surgery 46, no. 6 (2014): 933–934. 25228743 10.1093/ejcts/ezu333

[503] W. C. DeVries , J. L. Anderson , L. D. Joyce , et al., “Clinical Use of the Total Artificial Heart,” New England Journal of Medicine 310, no. 5 (1984): 273–278. 6690950 10.1056/NEJM198402023100501

[504] G. Torregrossa , A. Anyanwu , F. Zucchetta , and G. Gerosa , “SynCardia: The Total Artificial Heart,” Annals of Cardiothoracic Surgery 3, no. 6 (2014): 612–620. 25512904 10.3978/j.issn.2225-319X.2014.11.07 PMC4250553

[505] G. Torregrossa , M. Morshuis , R. Varghese , et al., “Results with Syncardia Total Artificial Heart beyond 1 Year,” ASAIO Journal 60, no. 6 (2014): 626–634. 25158888 10.1097/MAT.0000000000000132

[506] N. Stafford , “Leonard L Bailey: In 1984 he Transplanted a Baboon Heart Into a human Infant Known as “Baby Fae”,” Bmj 366 (2019): l4669.

[507] L. L. Bailey , S. L. Nehlsen‐Cannarella , W. Concepcion , and W. B. Jolley , “Baboon‐to‐Human Cardiac Xenotransplantation in a Neonate,” Jama 254, no. 23 (1985): 3321–3329. 2933538

[508] M. Mohiuddin , Tiny Matters. (Pig hearts in people: Xenotransplantation's long history, current promise, and the ethical use of brain‐dead people in research, 2024). August 21.

[509] L. Peterson , M. H. Yacoub , D. Ayares , et al., “Physiological Basis for Xenotransplantation From Genetically Modified Pigs to Humans,” Physiological Reviews 104, no. 3 (2024): 1409–1459. 38517040 10.1152/physrev.00041.2023 PMC11390123

[510] D. B. Joseph , J. G. Borer , R. E. De Filippo , S. J. Hodges , and G. A. McLorie , “Autologous Cell Seeded Biodegradable Scaffold for Augmentation Cystoplasty: Phase II Study in Children and Adolescents With Spina Bifida,” The Journal of Urology 191, no. 5 (2014): 1389–1395. 24184366 10.1016/j.juro.2013.10.103

[511] B. Jeon , C. Lee , M. Kim , T. H. Choi , S. Kim , and S. Kim , “Fabrication of Three‐dimensional Scan‐to‐print Ear Model for Microtia Reconstruction,” Journal of Surgical Research 206, no. 2 (2016): 490–497. 27884347 10.1016/j.jss.2016.08.004

[512] M. S. Mannoor , Z. Jiang , T. James , et al., “3D Printed Bionic Ears,” Nano Letters 13, no. 6 (2013): 2634–2639. 23635097 10.1021/nl4007744 PMC3925752

[513] X. Xie , S. Wu , S. Mou , N. Guo , Z. Wang , and J. Sun , “Microtissue‐Based Bioink as a Chondrocyte Microshelter for DLP Bioprinting,” Advanced Healthcare Materials 11, no. 22 (2022): 2201877. 36085440 10.1002/adhm.202201877 PMC11468467

[514] O. Y. Joo , T. H. Kim , Y. S. Kim , et al., “Fabrication of 3D‐Printed Implant for Two‐Stage Ear Reconstruction Surgery and Its Clinical Application,” Yonsei Medical Journal 64, no. 4 (2023): 291–296. 36996901 10.3349/ymj.2022.0547 PMC10067794

[515] J. J. Song , J. P. Guyette , S. E. Gilpin , G. Gonzalez , J. P. Vacanti , and H. C. Ott , “Regeneration and Experimental Orthotopic Transplantation of a Bioengineered Kidney,” Nature Medicine 19, no. 5 (2013): 646–651. 10.1038/nm.3154 PMC3650107 23584091

[516] C.‐Y. Hsu , P.‐L. Chi , H.‐Y. Chen , et al., “Kidney Bioengineering by Using Decellularized Kidney Scaffold and Renal Progenitor Cells,” Tissue and Cell 74 (2022): 101699. 34891081 10.1016/j.tice.2021.101699

[517] G. Satchanska , S. Davidova , and P. D. Petrov , “Natural and Synthetic Polymers for Biomedical and Environmental Applications,” Polymers 16, no. 8 (2024): 1159. 38675078 10.3390/polym16081159 PMC11055061

[518] A. Aazmi , D. Zhang , C. Mazzaglia , et al., “Biofabrication Methods for Reconstructing Extracellular Matrix Mimetics,” Bioactive Materials 31 (2024): 475–496. 37719085 10.1016/j.bioactmat.2023.08.018 PMC10500422

[519] C. Gao , Y. Wang , F. Han , et al., “Antibacterial Activity and Osseointegration of Silver‐coated Poly(ether ether ketone) Prepared Using the Polydopamine‐assisted Deposition Technique,” Journal of Materials Chemistry B 5, no. 47 (2017): 9326–9336. 32264535 10.1039/c7tb02436c

[520] S. Hiromoto and T. Yamazaki , “Micromorphological Effect of Calcium Phosphate Coating on Compatibility of Magnesium Alloy With Osteoblast,” Science and Technology of Advanced Materials 18, no. 1 (2017): 96–109. 28179963 10.1080/14686996.2016.1266238 PMC5259964

[521] W. Lin , L. Qin , H. Qi , et al., “Long‐term in Vivo Corrosion Behavior, Biocompatibility and Bioresorption Mechanism of a Bioresorbable Nitrided Iron Scaffold,” Acta Biomaterialia 54 (2017): 454–468. 28315492 10.1016/j.actbio.2017.03.020

[522] S. Ullah , I. Zainol , and R. H. Idrus , “Incorporation of Zinc Oxide Nanoparticles Into Chitosan‐collagen 3D Porous Scaffolds: Effect on Morphology, Mechanical Properties and Cytocompatibility of 3D Porous Scaffolds,” International Journal of Biological Macromolecules 104, no. Pt A (2017): 1020–1029. 28668615 10.1016/j.ijbiomac.2017.06.080

[523] J. Deng , Y. Tang , Q. Zhang , et al., “A Bioinspired Medical Adhesive Derived From Skin Secretion of Andrias davidianus for Wound Healing,” Advanced Functional Materials 29, no. 31 (2019): 1809110.

[524] N. Mandakhbayar , A. El‐Fiqi , J.‐H. Lee , and H.‐W. Kim , “Evaluation of Strontium‐Doped Nanobioactive Glass Cement for Dentin‐Pulp Complex Regeneration Therapy,” ACS Biomaterials Science & Engineering 5, no. 11 (2019): 6117–6126. 33405665 10.1021/acsbiomaterials.9b01018

[525] S. Petrus‐Reurer , M. Romano , S. Howlett , J. L. Jones , G. Lombardi , and K. Saeb‐Parsy , “Immunological Considerations and Challenges for Regenerative Cellular Therapies,” Communications Biology 4, no. 1 (2021): 798. 34172826 10.1038/s42003-021-02237-4 PMC8233383

[526] Q. Li and P. Lan , “Activation of Immune Signals During Organ Transplantation,” Signal Transduction and Targeted Therapy 8, no. 1 (2023): 110. 36906586 10.1038/s41392-023-01377-9 PMC10008588

[527] C. Y. X. Chua , A. Y. Jiang , T. Eufrasio‐da‐Silva , et al., “Emerging Immunomodulatory Strategies for Cell Therapeutics,” Trends in Biotechnology 41, no. 3 (2023): 358–373. 36549959 10.1016/j.tibtech.2022.11.008

[528] L. C. Kadyk , R. M. Okamura , and S. Talib , “Enabling Allogeneic Therapies: CIRM‐funded Strategies for Immune Tolerance and Immune Evasion,” Stem Cells Translational Medicine 9, no. 9 (2020): 959–964. 32585084 10.1002/sctm.20-0079 PMC7445020

[529] D. S. Masson‐Meyers and L. Tayebi , “Vascularization Strategies in Tissue Engineering Approaches for Soft Tissue Repair,” Journal of Tissue Engineering and Regenerative Medicine 15, no. 9 (2021): 747–762. 34058083 10.1002/term.3225 PMC8419139

[530] A. Sanchez‐Rubio , V. Jayawarna , E. Maxwell , M. J. Dalby , and M. Salmeron‐Sanchez , “Keeping It Organized: Multicompartment Constructs to Mimic Tissue Heterogeneity,” Advanced Healthcare Materials 12, no. 17 (2023): 2202110. 36938891 10.1002/adhm.202202110 PMC11469230

[531] G. Maroli and T. Braun , “The Long and Winding Road of Cardiomyocyte Maturation,” Cardiovascular Research 117, no. 3 (2021): 712–726. 32514522 10.1093/cvr/cvaa159

[532] A. S. Mao and D. J. Mooney , “Regenerative Medicine: Current Therapies and Future Directions,” Proceedings of the National Academy of Sciences of the United States of America 112, no. 47 (2015): 14452–14459. 26598661 10.1073/pnas.1508520112 PMC4664309

[533] K. D. Nordham and S. Ninokawa , “The History of Organ Transplantation,” Baylor University Medical Center Proceedings 35, no. 1 (2022): 124–128. 34970061 10.1080/08998280.2021.1985889 PMC8682823

[534] M. Hofer and M. P. Lutolf , “Engineering Organoids,” Nature Reviews Materials 6, no. 5 (2021): 402–420. 33623712 10.1038/s41578-021-00279-y PMC7893133

[535] A. Oryan , S. Alidadi , A. Moshiri , and N. Maffulli , “Bone Regenerative Medicine: Classic Options, Novel Strategies, and Future Directions,” Journal of Orthopaedic Surgery and Research 9, no. 1 (2014): 18. 24628910 10.1186/1749-799X-9-18 PMC3995444

[536] A. El‐Sayed and M. Kamel , “Advances in Nanomedical Applications: Diagnostic, Therapeutic, Immunization, and Vaccine Production,” Environmental Science and Pollution Research 27, no. 16 (2020): 19200–19213. 31529348 10.1007/s11356-019-06459-2

[537] R. Augustine , P. Dan , A. Hasan , et al., “Stem Cell‐based Approaches in Cardiac Tissue Engineering: Controlling the Microenvironment for Autologous Cells,” Biomedicine & Pharmacotherapy 138 (2021): 111425. 33756154 10.1016/j.biopha.2021.111425

[538] I.‐S. Hong , “Enhancing Stem Cell‐Based Therapeutic Potential by Combining Various Bioengineering Technologies,” Frontiers in Cell and Developmental Biology 10 (2022): 901661. 35865629 10.3389/fcell.2022.901661 PMC9294278

[539] H. Lin , Y. Tang , T. P. Lozito , N. Oyster , B. Wang , and R. S. Tuan , “Efficient in Vivo Bone Formation by BMP‐2 Engineered human Mesenchymal Stem Cells Encapsulated in a Projection Stereolithographically Fabricated Hydrogel Scaffold,” Stem Cell Research & Therapy 10, no. 1 (2019): 254. 31412905 10.1186/s13287-019-1350-6 PMC6694509

[540] A. S. Cakmak , S. Fuerkaiti , D. Karaguzel , C. Karaaslan , and M. Gumusderelioglu , “Enhanced Osteogenic Potential of Noggin Knockout C2C12 Cells on BMP‐2 Releasing Silk Scaffolds,” ACS Biomaterials Science & Engineering Journal 9, no. 11 (2023): 6175–6185. 10.1021/acsbiomaterials.3c00506 PMC10646847 37796024

[541] S. Prithiviraj , A. G. Garcia , K. Linderfalk , et al., Compositional editing of extracellular matrices by CRISPR/Cas9 engineering of human mesenchymal stem cell lines. (eLife Sciences Publications, Ltd, 2024). 10.7554/eLife.96941 PMC11952750 40152921

[542] C. Zhong , S. He , Y. Huang , et al., “Scaffold‐based Non‐viral CRISPR Delivery Platform for Efficient and Prolonged Gene Activation to Accelerate Tissue Regeneration,” Acta Biomaterialia 173 (2024): 283–297. 37913843 10.1016/j.actbio.2023.10.029

[543] C. Li , Z. Mills , and Z. Zheng , “Novel Cell Sources for Bone Regeneration,” MedComm 2, no. 2 (2021): 145–174. 34766140 10.1002/mco2.51 PMC8491221

[544] P. Chen , L. Cui , G. Chen , et al., “The Application of BMP‐12‐overexpressing Mesenchymal Stem Cells Loaded 3D‐printed PLGA Scaffolds in Rabbit Rotator Cuff Repair,” International Journal of Biological Macromolecules 138 (2019): 79–88. 31295489 10.1016/j.ijbiomac.2019.07.041

[545] D. Hao , J.‐M. Lopez , J. Chen , A. M. Iavorovschi , N. M. Lelivelt , and A. Wang , “Engineering Extracellular Microenvironment for Tissue Regeneration,” Bioengineering 9, no. 5 (2022): 202. 35621480 10.3390/bioengineering9050202 PMC9137730

[546] J. Gao , X. Yu , X. Wang , Y. He , and J. Ding , “Biomaterial–Related Cell Microenvironment in Tissue Engineering and Regenerative Medicine,” Engineering 13 (2022): 31–45.

[547] M. Schot , N. Araújo‐Gomes , B. van Loo , T. Kamperman , and J. Leijten , “Scalable Fabrication, Compartmentalization and Applications of Living Microtissues,” Bioactive Materials 19 (2022): 392–405. 35574053 10.1016/j.bioactmat.2022.04.005 PMC9062422

[548] I. Decoene , G. Nasello , R. F. Madeiro de Costa , et al., “Robotics‐Driven Manufacturing of Cartilaginous Microtissues for Skeletal Tissue Engineering Applications,” Stem Cells Translational Medicine 13, no. 3 (2024): 278–292. 38217535 10.1093/stcltm/szad091 PMC10940839

[549] B. Zhang , A. Korolj , B. F. L. Lai , and M. Radisic , “Advances in Organ‐on‐a‐chip Engineering,” Nature Reviews Materials 3, no. 8 (2018): 257–278.

[550] K. Thakare , L. Jerpseth , Z. Pei , A. Elwany , F. Quek , and H. Qin , “Bioprinting of Organ‐on‐Chip Systems: A Literature Review From a Manufacturing Perspective,” Journal of Manufacturing and Materials Processing 5, no. 3 (2021): 91.

