# Supplementary material for: Tissue Engineering and Regenerative Medicine: Perspectives and Challenges
Source: MedComm (2020). 2025 Apr 24;6(5):e70192. doi: 10.1002/mco2.70192 (PMC12022429; doi:10.1002/mco2.70192)
Supplement: Supplementary file 1 — Supporting Information [file MCO2-6-e70192-s001.docx]

**Supplementary materials**

**Title: Tissue Engineering and Regenerative Medicine: Perspectives and Challenges**

Van T. Hoang^1,2*^, Quyen Thi Nguyen^1,2*^, Trang Thi Kieu Phan^1,2^, Trang H. Pham^1,2^, Nhung Thi Hong Dinh^1,2^, Le Phuong Hoang Anh^1,2^, Lan Thi Mai Dao^1,2^, Van Dat Bui^1,3^, Hong-Nhung Dao^1,2^, Duc Son Le^1,2^, Anh Thi Lan Ngo^1,2^, Quang-Duong Le^1,2^, Liem Nguyen Thanh^1,2*^

^1^Vinmec Research Institute of Stem Cell and Gene Technology, College of Health Science, VinUniversity, Vinhomes Ocean Park, Gia Lam District, Hanoi 1310, Vietnam

^2^Vinmec Health Care System, 458 Minh Khai, Hanoi 11622, Vietnam

^3^School of Chemical Engineering, College of Engineering, Sungkyunkwan University (SKKU), Suwon 16419, Republic of Korea

***Co-first authors**

**Corresponding authors:**

Liem Nguyen Thanh, PhD, Vinmec Research Institute of Stem Cell and Gene Technology, College of Health Science, VinUniversity, Vinhomes Ocean Park, Gia Lam District, Hanoi 1310, Vietnam. Phone: (+84 24) 3 975 1418. Email: [liem.nt@vinuni.edu.vn](mailto:liem.nt@vinuni.edu.vn)**,** [v.liemnt@vinmec.com](mailto:v.liemnt@vinmec.com);

Van T. Hoang, PhD., Vinmec Research Institute of Stem Cell and Gene Technology, College of Health Science, VinUniversity, Vinhomes Ocean Park, Gia Lam District, Hanoi 1310, Vietnam. Phone: (+84 24) 3 975 2301. Email: [van.ht@vinuni.edu.vn](mailto:van.ht@vinuni.edu.vn)**,** [v.vanht8@vinmec.com](mailto:v.vanht8@vinmec.com);

**Running title: Tissue Engineering and Regenerative Medicine**

**Table S1 Clinical Trials Utilizing Cell Therapy for the Treatment of Pulmonary Diseases**

| **Year** | **Study title** | **Disease** | **Number of Treated patients** | **Cell dose** | **Phase** | **Cell source** |
| --- | --- | --- | --- | --- | --- | --- |
| 2015 | In vivo effects of mesenchymal stromal cells in two patients with severe acute respiratory distress syndrome ^1^ | ARDS | 2 were treated with MSCs | Both patients received 2x10^6^ cells per kilogram | case study | BM-MSC |
| 2015 | Mesenchymal stem (stromal) cells for treatment of ARDS: a phase 1 clinical trial ^2^ | ARDS | A total of 9 patients were treated across the three dosing groups (3 patients per dose level) | 1x10^6^, 5x10^6^, and 10x10^6^ cells/kg | phase 1 | Allogeneic BM-MSC |
| 2019 | Treatment with allogeneic mesenchymal stromal cells for moderate to severe acute respiratory distress syndrome (START study): a randomized phase 2a safety trial^3^ | ARDS | 40 patients received MSCs, while 20 patients received placebo (total study sample = 60 patients). | Patients were randomly assigned 2:1 to receive either 10×10^6^/kg predicted bodyweight MSCs or placebo | phase 2a | BM-MSC |
| 2020 | Human umbilical cord-derived mesenchymal stem cells for acute respiratory distress syndrome^4^ | ARDS | 9 patients with moderate-to-severe acute respiratory distress syndrome (ARDS) | Low dose: 1x10^6^ cells/kg (administered to 3 patients). Intermediate dose: 5x10^6^ cells/kg (administered to 3 patients). High dose: 10x10^6^ cells/kg (administered to 3 patients). | phase I (safety and feasibility study) | UC-MSC |
| 2021 | Safety and efficacy of multipotent adult progenitor cells in acute respiratory distress syndrome (MUST-ARDS): a multicentre, randomised, double-blind, placebo-controlled phase 1/2 trial^5^ | ARDS | Cohorts 1 and 2: 6 patients.Cohort 3: 20 patients (cell therapy group) + 10 patients (placebo group). | Cohort 1: 300 million cells (3 patients).Cohort 2: 900 million cells (3 patients).Cohort 3: 900 million cells (20 patients) or placebo (10 patients) | phase 1/2 trial | Bone marrow-derived multipotent adult progenitor cells |
| 2021 | Repair of acute respiratory distress syndrome by stromal cell administration (REALIST) trial: a phase 1 trial^6^ | ARDS | 9 patients with moderate-to-severe ARDS | 100×10^6^ cells (3 patients) 200×10^6^ cells (3 patients) 400×10^6^ cells (3 patients) of a single intravenous | phase 1 | CD362 enriched umbilical cord-derived mesenchymal stromal cells (ORBCEL-C) |
| 2023 | Clinical efficacy and safety of multipotent adult progenitor cells (invimestrocel) for acute respiratory distress syndrome (ARDS) caused by pneumonia: a randomized, open-label, standard therapy-controlled, phase 2 multicenter study (ONE-BRIDGE)^7^ | ARDS | 20 patients | 9.0×10^8^ cells | phase 2 | BM_MSC (HLCM051) |
| 2014 | Mesenchymal stem cells for bronchopulmonary dysplasia: phase 1 dose-escalation clinical trial^8^ | BPD | 9 preterm infants | Low Dose: 1×10^7^ cells/kg (administered to the first 3 patients). High Dose: 2×10^7^ cells/kg (administered to the next 6 patients). | phase 1  dose-escalation | Allogeneic human umbilical cord blood (hUCB)-MSC |
| 2017 | Two-Year Follow-Up Outcomes of Premature Infants Enrolled in the Phase I Trial of Mesenchymal Stem Cells Transplantation for Bronchopulmonary Dysplasia^9^ | BPD | 9 preterm infants | Low dose: 1×10^7^ cells/kg in 2 mL/kg of saline (administered to the first 3 infants).High dose: 2×10^7^ cells/kg in 4 mL/kg of saline (administered to the next 6 infants). | follow - up from | Allogeneic human umbilical cord blood (hUCB)-MSC |
| 2017 | Bone Marrow Mononuclear Cells Transplantation in Treatment of Established Bronchopulmonary Dysplasia: A Case Report^10^ | BPD | 1 patient | 620x10^6^ MNCs, with 43.85x10^6^ hHSCs (CD45 dim CD34+), were administered as follows: Intratracheal: 0.5 ml BM MNCs, 4 times every 30 min. Intravenous: 2 ml/h via endotracheal, nebulization, and central venous catheter over 60 min. | case study | Autologous BM MNCs |
| 2018 | Airway Delivery of Bone Marrow–Derived Mesenchymal Stem Cells Reverses Bronchopulmonary Dysplasia Superimposed with Acute Respiratory Distress Syndrome in an Infant^11^ | BPD | 1 patient (a 10-month-old female infant) | 6.25x10^6^ cells/kg (total dose: 5x10^7^ cells for an 8.0 kg patient) | case study | BM-MSC |
| 2018 | Off-label mesenchymal stromal cell treatment in two infants with severe bronchopulmonary dysplasia: clinical course and biomarkers profile^12^ | BPD | 2 infants | Patient 1: Increasing weekly doses starting from 1.1 million cells/kg up to 13.9 million cells/kg.Patient 2: 5 million cells/kg per week for 3 consecutive weeks, totaling 15 million cells/kg. | Off-label treatment | Allogenic BM-MSC |
| 2019 | Safety of Intratracheal Administration of Human Umbilical Cord Blood Derived Mesenchymal Stromal Cells in Extremely Low Birth Weight Preterm Infants^13^ | BPD | 12 preterm infants | Lower dose group: 1.0×10⁷ cells/kg (administered as 2 mL/kg in 2 aliquots). Higher dose group: 2.0×10⁷ cells/kg (administered as 4 mL/kg in 4 aliquots). | Phase 1, dose-escalation trial | UCB-MSC |
| 2020 | Allogeneic administration of human umbilical cord-derived mesenchymal stem/stromal cells for bronchopulmonary dysplasia: preliminary outcomes in four Vietnamese infants^14^ | BPD | 4 patients | Each patient received 1 million cells/kg of patient body weight (PBW) per dose.Two doses were administered intravenously with a 7-day interval between them. | Preliminary safety study | Allogeneic UC-MSC |
| 2021 | Stem cells for bronchopulmonary dysplasia in preterm infants: a randomized controlled phase II trial^15^ | BPD | 33 patients in the MSC group (compared to 33 in the placebo group), making a total of 66 patients enrolled in the study | 1x10^7^ cells/kg administered intratracheally | Phase II, double-blind, randomized, placebo-controlled clinical trial | UCB-MSC |
| 2023 | Mesenchymal stem cell application in a newborn with severe intraventricular hemorrhage and respiratory distress^16^ | BPD | 1 patient | Intratracheal: 10×10^6^ cells/kg (10 million cells per kilogram) Intravenous: 10×10^6^ cells/kg (10 million cells per kilogram) Intraventricular: 5×10^6^ cells/kg (5 million cells per kilogram) | case study | UCB-MSC |
| 2024 | Repeated intravenous doses of human umbilical cord-derived mesenchymal stromal cells for bronchopulmonary dysplasia: results of a phase 1 clinical trial with 2-year follow-up^17^ | BPD | 10 patients | 5×10^6^ cells/kg per dose, administered three times at weekly intervals | Phase 1, open-label, single-arm, multi-center clinical trial | UC-MSC |
| 2024 | The safety and efficacy of stem cells for the treatment of severe community-acquired bacterial pneumonia: A randomized clinical trial^18^ | CABP | 42 patients | 1.6x10^8^ cells per infusion, administered twice (on Day 1 and Day 3) - Intravenous infusion of treatment ( | Phase 1b/2a | Expanded allogeneic adipose-derived mesenchymal stem cells (eASCs) |
| 2011 | Unicentric study of cell therapy in chronic obstructive pulmonary disease/pulmonary emphysema^19^ | COPD | 4 patients | 1×10^8^ mononuclear cells/mL infused into a peripheral vein with a final volume of 30 mL | This was a pilot clinical trial registered: NCT01110252 | BMMNC |
| 2013 | A placebo-controlled, randomized trial of mesenchymal stem cells in COPD^20^ | COPD | 62 patients were randomized into the study (30 patients in MSC group and 32 patients in placebo group) | Patients received four monthly infusions (100x10^6^ cells/infusion) and were subsequently followed for 2 years after the first infusion | phase 2 | BM-MSC |
| 2013 | Phase I clinical trial of cell therapy in patients with advanced chronic obstructive pulmonary disease: follow-up of up to 3 years^21^ | COPD | 4 patients | 1x10^8^ cells/kg were infused into a peripheral vein | phase 1 | BMMNC |
| 2015 | Prospective phase 1 open clinical trial to study the safety of adipose-derived mesenchymal stem cells (ADMSCs) in COPD and combined pulmonary fibrosis and emphysema (CPFE)^22^ | COPD | 8 patients | A mean of 160x10^6^ cells (range: 182-300 million cells) per patient, administered in 3 infusions. | phase 1 | Autologous AD-MSC |
| 2017 | Combined bone marrow-derived mesenchymal stromal cell therapy and one-way  endobronchial valve placement in patients with pulmonary emphysema: a phase I clinical trial^23^ | COPD | 5 patients | allogeneic bone marrow-derived MSCs (10^8^ cells) | phase 1 pilot study | BM-MSC |
| 2017 | Autologous stromal vascular fraction in the intravenous treatment of  end-stage chronic obstructive pulmonary disease: a phase I trial of safety and tolerability^24^ | COPD | 12 patients | 150 - 300x10^6^ cells infused intravenously | phase 1 | AD-MSC |
| 2018 | Mesenchymal stromal cell infusion modulates systemic immunological responses in stable COPD patients: a phase I pilot study^25^ | COPD | 9 COPD patients (1 GOLD stage I, 2 GOLD II, 3 GOLD III, 3 GOLD IV) | COPD patients received two infusions of allogeneic BM-MSCs at a dose of 2x10^6^ MSCs per kilogram of body weight | phase 1 pilot study | Allogeneic BM-MSC |
| 2020 | Improving effects of mesenchymal stem cells on symptoms of chronic obstructive pulmonary disease^26^ | COPD | 5 patients | All the patients were administered a total of 4 doses of UCTD MSCs by intravenous infusion at two-week intervals. | phase 1/2 | UC-MSC |
| 2020 | Allogeneic umbilical cord-derived mesenchymal stem cell transplantation for treating chronic obstructive pulmonary disease: a pilot clinical study^27^ | COPD | 20 patients (9 patients at stage C and 11 at stage D of COPD) | 1.5x10^6^ UC-MSCs per kilogram of body weight | Pilot clinical trial | UC-MSC |
| 2020 | Safety and feasibility of umbilical cord mesenchymal stem cells in patients with COVID-19 pneumonia: a pilot study^28^ | COVID-19 | 16 patients | 1x10^8^ cells per infusion.Four rounds of transplantation were administered with one-day intervals in between. | pilot study | UC-MSC |
| 2020 | Treatment of severe COVID-19 with human umbilical cord mesenchymal stem cells^29^ | COVID-19 | 12 patients | 2×10^6^ cells/kg. Intravenous administration of MSCs infused over approximately 1 hour | pilot study | UC-MSC |
| 2020 | Umbilical cord blood-derived mesenchymal stem cells in treating a critically ill COVID-19 patient^30^ | COVID-19 | 1 | 1.5×10^6^ cells per kilogram of the patient’s weight, infused intravenously every 48 hours, with a total of five infusions | case study | UCB-MSC |
| 2020 | Human umbilical cord-derived mesenchymal stem cell therapy in patients with COVID-19: a phase 1 clinical trial^31^ | COVID-19 | 9 | 3×10^7^ cells per infusion, administered intravenously in three cycles on days 0, 3, and 6 | phase 1 | UC-MSC |
| 2020 | Mesenchymal stem cells derived from perinatal tissues for treatment of critically ill COVID-19-induced ARDS patients: a case series^32^ | COVID-19 | 11 patients (6 received UC-MSCs and 5 received PL-MSCs). | a total of 600×10^6^ MSCs, administered in three infusions of 200×10^6^ each, given every other day | phase 1 | UC-MSC and P-MSC |
| 2020 | Intravenous infusion of human umbilical cord Wharton's jelly-derived mesenchymal stem cells as a potential treatment for patients with COVID-19 pneumonia^33^ | COVID-19 | One patient, a critically ill 54-year-old man with severe COVID-19 | 1×10^6^ cells per kilogram of patient weight, infused intravenously. | case study | UC-MSC |
| 2021 | Mesenchymal stem cell therapy for severe COVID-19: 1-year follow-up results of a randomized, double-blind, placebo-controlled trial^34^ | COVID-19 | 65 patients received UC-MSCs, with 35 receiving a placebo (total of 100 patients enrolled) | 4.0×10^7^ cells per procedure, administered intravenously on day 0, day 3, and day 6, for a total of three infusions | phase 2 | UC-MSC |
| 2021 | Administration of umbilical cord mesenchymal stem cells in patients with severe COVID-19 pneumonia^35^ | COVID-19 | 31 patients with severe or critical COVID-19 pneumonia | 1x10^6^ cells per kilogram of body weight, suspended in 100 mL of normal saline. The total volume of infusion ranged from 100 to 300 mL. | not mentioned | UC-MSC |
| 2021 | Mesenchymal stem cell treatment improves outcome of COVID-19 patients via multiple immunomodulatory mechanisms^36^ | COVID-19 | 29 patients were treated with UC-MSCs (compared to 29 in the placebo group, total 58 patients). | 1x10^7^ cells per kilogram body weight | Phase II clinical trial (randomized, single-blind, placebo-controlled) | UC-MSC |
| 2021 | Effect of human umbilical cord-derived mesenchymal stem cells on lung damage in severe COVID-19 patients: a randomized, double-blind, placebo-controlled phase 2 trial^37^ | COVID-19 | 65 patients received UC-MSCs (out of 100 patients in total, with 35 in the placebo group) | 4x10^7^ cells per infusion, given on day 0, day 3, and day 6 | phase 2 | UC-MSC |
| 2021 | Clinical experience on umbilical cord mesenchymal stem cell treatment in 210 severe and critical COVID-19 cases in Turkey^38^ | COVID-19 | 210 patients (99 critically severe and 111 severe unintubated patients) | 1–2x10^6^ cells per kilogram, intravenously | Phase I: Severe unintubated patients Phase II: Critically severe intubated patients | UC-MSC |
| 2021 | Cell therapy in patients with COVID-19 using Wharton’s jelly mesenchymal  stem cells: a phase 1 clinical trial^39^ | COVID-19 | 5 patients with severe COVID-19 | 150x10^6^ cells per injection, administered three times (on days 0, 3, and 6) intravenously | pilot study | UC-MSC |
| 2021 | The systematic effect of mesenchymal stem cell therapy in critical  COVID-19 patients: a prospective double controlled trial^40^ | COVID-19 | 10 patients | 3x10^6^ cells/kg, administered intravenously on treatment days 0, 3, and 6. | not mention | UC-MSC |
| 2023 | Human mesenchymal stem cell therapy in severe COVID-19 patients: 2-year follow-up results of a randomized, double-blind, placebo-controlled trial^41^ | COVID-19 | 65 patients received MSCs and 35 received a placebo, totaling 100 patients in the trial | 4.0×10⁷ cells per dose, administered intravenously, three times at 3-day intervals | phase 2 | UC-MSC |
| 2023 | SARS-CoV-2-specific T cell therapy for severe COVID-19: a randomized phase 1/2 trial^42^ | COVID-19 | 60 patients received CoV-2-ST+SoC, 30 received SoC only (control), totaling 90 participants in the trial. | n/a | phase 1/2 | Partially human leukocyte antigen (HLA)-matched, convalescent donor-derived SARS-CoV-2-specific T cells (CoV-2-STs). |
| 2019 | First-in-human high-cumulative-dose stem cell therapy in idiopathic pulmonary fibrosis with rapid lung function decline^43^ | Idiopathic pulmonary fibrosis | 10 patients received MSC treatment (total 20 patients in the study, with 10 receiving MSCs and 10 receiving a placebo) | each patient received two IV doses of allogeneic MSCs (2x10^8^ cells) every 3 months, totaling 1.6x10^9^ MSCs | Phase 1/2a | BM-MSC |
| 2016 | A phase I study for intravenous autologous mesenchymal stromal cell administration to patients with severe emphysema^44^ | Emphysema | Of the 10 initially included, three were excluded: two for insufficient MSC expansion and one due to inability to undergo the second surgery | Patients received two intravenous infusions of BM-MSCs at a dose of 1–2x10^6^ BM-MSCs per kilogram of body weight, one week apart | Phase 1 | BM-MSC |

**Table S2: Clinical trials utilizing cell therapy for the treatment of cardiovascular diseases**

| **Year** | **Study title** | **Disease** | **Number of Treated patient** | **Cell dose** | **Phase** | **Cell source** |
| --- | --- | --- | --- | --- | --- | --- |
| 2014 | Adipose-derived regenerative cells in patients with ischemic cardiomyopathy: the PRECISE trial^45^ | Chronic ischemic cardiomyopathy | 21 patients | 0.4, 0.8, and 1.2×10^6^ cells/kg | phase 1 | AD-MSC |
| 2020 | Intramyocardial transplantation of umbilical cord mesenchymal stromal cells in chronic ischemic cardiomyopathy: a controlled, randomized clinical trial (HUC-HEART trial)^46^ | Chronic ischemic cardiomyopathy | 26 in HUC-MSC group, 12 in BM-MNC group, and 16 in control (no cells) | HUC-MSC group: 23×10^6^ cells.BM-MNC group: 70×10^7^ cells. | phase 1/2 | HUC-MSCs and autologous BM-MNCs |
| 2017 | Adipose-derived stromal cells for treatment of patients with chronic ischemic heart disease (MyStromalCell Trial): a randomized placebo-controlled study.^47^ | Chronic Ischemic Heart Disease | 40 patients | 72.0 ± 44.9x10^6^ cells | phase 2 (randomized, double-blind, placebo-controlled study) | AD-MSC stimulated with VEGF-A165. |
| 2020 | Effect of intramyocardial grafting collagen scaffold with mesenchymal stromal cells in patients with chronic ischemic heart disease: a randomized clinical trial.^48^ | Chronic ischemic heart disease | Collagen/cell: 18 patients; Cell: 17 patients; Control: 15 patients. | Collagen/cell: 1×10^8^ hUC-MSCs in 1.5 mL PBS + 1 mL collagen; Cell-only: 1×10^8^ hUC-MSCs in 2.5 mL PBS. | phase 1 | UC-MSC |
| 2017 | The Athena trials: autologous adipose-derived regenerative cells for refractory chronic myocardial ischemia with left ventricular dysfunction.^49^ | Chronic myocardial ischemia | 17 patients in total across both trials. | 40x10^6^ cells (n=14); 80x10^6^ cells (n=3) | not mentioned | autologous AD-MSC |
| 2023 | Report of a phase 1 clinical trial for safety assessment of human placental mesenchymal stem cells therapy in patients with critical limb ischemia (CLI)^50^ | critical limb ischemia | 9 patients | 20×10⁶ (low) and 60×10⁶ (high) cells, given intramuscularly twice, two months apart | Phase 1 dose-escalation clinical trial. | P-MSCs |
| 2013 | Cardiopoietic stem cell therapy in heart failure: the C-CURE (cardiopoietic stem Cell therapy in heart failURE) multicenter randomized trial with lineage-specified biologics.^51^ | Heart failure | 21 patients | 605x10^6^ to 1,168x10^6^ cells | Phase 1/2 clinical trial | BM-MSC |
| 2017 | Safety and efficacy of the intravenous infusion of umbilical cord mesenchymal stem cells in patients with heart failure: a phase 1/2 randomized controlled trial (RIMECARD Trial [randomized clinical trial of intravenous infusion umbilical cord mesenchymal stem cells on cardiopathy])^52^ | Heart failure | 15 patients | 1x10^6^ cells/kg. | Phase 1/2, randomized, double-blind, placebo-controlled clinical trial. | UC-MSC |
| 2017 | Dose comparison study of allogeneic mesenchymal stem cells in patients with ischemic cardiomyopathy (The TRIDENT Study)^53^ | Ischemic Cardiomyopathy | 30 patients (15 in each group: 20 M and 100 M) | 20x10^6^ (20 M) and 100x10^6^ (100 M) cells | Phase 2 | allogenic BM-MSC |
| 2012 | Comparison of allogeneic vs autologous bone marrow-derived mesenchymal stem cells delivered by transendocardial injection in patients with ischemic cardiomyopathy: the POSEIDON randomized trial^54^ | Ischemic cardiomyopathy | 30 patients (15 received autologous MSCs, 15 received allogeneic MSCs). | 20×10⁶, 100×10⁶, or 200×10⁶ cells via transendocardial injection | Phase 1/2 randomized comparison (POSEIDON study) | BM-MSC |
| 2013 | Transendocardial mesenchymal stem cells and mononuclear bone marrow cells for ischemic cardiomyopathy: the TAC-HFT randomized trial.^55^ | Ischemic cardiomyopathy | 19 patients received MSCs, and 19 patients received BMCs. | not mentioned | Phase 1 and 2 | Autologus BM-MSC and Autologous BMCs |
| 2017 | Cryopreserved off-the-shelf allogeneic adipose-derived stromal cells for therapy in patients with ischemic heart disease and heart failure—a safety study.^56^ | Ischemic heart failure | 10 patients | 110x10^6^ cells | phase 1 | AD-MSC |
| 2017 | Cardiopoietic cell therapy for advanced ischemic heart failure: results at 39 weeks of the prospective, randomized, double blind, sham-controlled CHART-1 clinical trial.^57^ | Ischemic heart failure | 120 patients received stem cell (out of 315 who were randomized) | 24x10^6^ cells | Phase 2/3 | BM Cardiopoietic cells (produced through cardiogenic conditioning of patients’ mesenchymal stem cells) |
| 2021 | A Phase II study of autologous mesenchymal stromal cells and c-kit positive cardiac cells, alone or in combination, in patients with ischemic heart failure: the CCTRN CONCERT-HF trial.^58^ | Ischemic heart failure | Total of 125 patients enrolled, randomized (1:1:1:1) into four groups: • MSCs + CPCs: 33 patients. • MSCs: 29 patients. • CPCs: 31 patients. • Placebo: 32 patients. | MSCs: 150×10^6^ cells.CPCs: 5 × 10^6^ cells. | Phase 2 | Autologous BM-MSC and C-kit positive cardiac cells (CPCs). |
| 2017 | Randomized comparison of allogeneic versus autologous mesenchymal stem cells for nonischemic dilated cardiomyopathy: POSEIDON-DCM trial.^59^ | Nonischemic Dilated Cardiomyopathy | 34 (16 autohMSCs, 18 allo-hMSCs). | 100x10^6^ cells | Phase 1/2, randomized pilot study | autologous and allogeneic BM-MSC |
| 2019 | Autologous adipose-derived stromal cell treatment for patients with refractory angina (MyStromalCell Trial): 3-years follow-up results.^60^ | Refractory angina | 40 patients | 72 ± 45x10^6^ cells | Phase 2, double-blinded, placebo-controlled study | Autologous AD-MSC |
| 2015 | Bone marrow-derived mesenchymal stromal cell treatment in patients with severe ischemic heart failure: a randomized placebo-controlled trial (MSC-HF trial)^61^ | Severe ischemic heart failure | 40 patients | 77.5 ± 67.9x10^6^ cells | Phase 2 (randomized, double-blind, placebo-controlled study). | BM-MSC |

**Table S3 Recent clinical trials utilizing cell therapy for the treatment of musculoskeletal diseases**

| **Year** | **Study title** | **Disease** | **No of treated patient** | **Cell dose** | **Phase** | **Cell source** |
| --- | --- | --- | --- | --- | --- | --- |
| 2023 | Clinical Efficacy and Safety of the Intra-articular Injection of Autologous Adipose-Derived Mesenchymal Stem Cells for Knee Osteoarthritis: A Phase III, Randomized, Double-Blind, Placebo-Controlled Trial^62^ | Osteoarthritis | 125 patients received cells; 127 were controls. | 1 × 10⁸ cells | phase 3 | Autologous AD-MSCs |
| 2024 | Safety and Tolerability of Intra-Articular Injection of Adipose-Derived Mesenchymal Stem Cells GXCPC1 in 11 Subjects With Knee Osteoarthritis: A Nonrandomized Pilot Study Without a Control Arm^63^ | Osteoarthritis | 11 patients : 5 low-dose, 6 high-dose. | Low dose: 6.7 × 10⁶ GXCPC1 cellsHigh dose: 4 × 10⁷ GXCPC1 cells | Phase 1 | Allogeneic AD-MSC labeled as GXCPC1 |
| 2024 | A Phase I Dose-Escalation Clinical Trial to Assess the Safety and Efficacy of Umbilical Cord-Derived Mesenchymal Stromal Cells in Knee Osteoarthritis^64^ | Osteoarthritis | 16 low dose, 16 middle dose, 8 high dose (recruitment stopped early due to adverse events). | Low dose: 2×10⁶, middle dose: 20×10⁶, high dose: 80×10⁶ cells. | Phase 1 | UC-MSC labeled as Cellistem |
| 2023 | First Human Report of Relief of Lumbar and Cervical Discogenic and Arthritic Back Pain after Epidural and Facet Joint Mesenchymal Stem Cell Injection: A Case Report^65^ | arthritic | 1 patient | IV infusion: 87×10⁶ MSCs; Injections: 1×10⁶ per lumbar facet joint (8 total), 5×10⁶ M in lumbar epidural space. | case report | Allogeneic UC-MSC |
| 2023 | Cartilage regeneration and inflammation modulation in knee osteoarthritis following injection of allogeneic adipose-derived mesenchymal stromal cells: a phase II, triple-blinded, placebo controlled, randomized trial^66^ | Osteoarthritis | treated group: 20 patients; placebo group: 20 patients | 100 × 10⁶ cells per injection. | Phase 2 triple-blinded randomized clinical trial | Allogeneic (AD-MSCs) |
| 2023 | Three-dimensional texture analyses of multiquantitative relaxation time maps for evaluating cartilage repair with the treatment of allogeneic human adipose-derived mesenchymal progenitor cells^67^ | Cartilage repair | 18 patients | Doses: Low: 1.0×10⁷, Middle: 2.0×10⁷, High: 5.0×10⁷ cells. | Phase 1/2a clinical trial | Allogeneic AD-MSC |

**Table S4 Recent clinical trials utilizing cell therapy for the treatment of reproductive diseases**

| **Year** | **Study title** | **Disease** | **No of treated patient** | **Cell dose** | **Phase** | **Cell source** |
| --- | --- | --- | --- | --- | --- | --- |
| 2016 | Autologous Stem Cells Therapy, The First Baby of Idiopathic Premature Ovarian Failure^68^ | Primary ovarian failure | 10 patients | NA | Uncontrolled pilot study | Autologous BM-MSCs |
| 2018 | Transplantation of UC-MSCs on collagen scaffold activates follicles in dormant ovaries of POF patients with long history of infertility^69^ | Primary ovarian failure | Allogenic UC-MSCs on collagen scaffold (8 patients) and  UC-MSCs (6 patients) | 10x10^6^ | Single-center randomized controlled trial | Allogenic UC-MSCs with or without collagen scaffold |
| 2018 | Role of autologous bone marrow-derived stem cell therapy for follicular recruitment in premature ovarian insufficiency: Review of literature and a case report of world's first baby with ovarian autologous stem cell therapy in a perimenopausal woman of age 45 year^70^ | Primary ovarian failure | 1 patient | 120 ml BM aspirate | Case report | Autologous BM-derived stem cells |
| 2018 | Autologous stem cell ovarian transplantation to increase reproductive potential in patients who are poor responders^71^ | Primary ovarian failure | 15 patients | Apheresis product containing 50x10^6^ CD133^+^ cells | Uncontrolled pilot study | Autologous stem cells from mobilized peripheral blood |
| 2020 | Intraovarian injection of autologous human mesenchymal stem cells increases estrogen production and reduces menopausal symptoms in women with premature ovarian failure: two case reports and a review of the literature^72^ | Primary ovarian failure | 2 patients | NA | Case report | Autologous BM-MSCs |
| 2020 | Clinical analysis of human umbilical cord mesenchymal stem cell allotransplantation in patients with premature ovarian insufficiency^73^ | Primary ovarian failure | 61 patients | 5×10^6^ cells in each ovary, up to three injections | Nonrandomized clinical trial | Allogenic UC-MSCs |
| 2021 | Evaluation of safety, feasibility and efficacy of intra-ovarian transplantation of autologous adipose derived mesenchymal stromal cells in idiopathic premature ovarian failure patients: non-randomized clinical trial, phase I, first in human^74^ | Primary ovarian failure | 9 patients | 5x10^6^  10x10^6^  15x10^6^ | Nonrandomized clinical trial, phase I | Autologous ADSCs |
| 2018 | Allogeneic cell therapy using umbilical cord MSCs on collagen scaffolds for patients with recurrent uterine adhesion: a phase I clinical trial^75^ | Asherman’s syndrome | 26 patients | 10×10^6^ | Non-controlled, phase I clinical trial | Allogenic UC-MSCs with a collagen scaffold |
| 2020 | Autologous bone marrow-derived stem cell therapy for Asherman's syndrome and endometrial atrophy: A 5-year follow-up study^76^ | Asherman’s syndrome and endometrial atrophy | 25 patients | 65.3± 37.2×10^6^ | Single-arm longitudinal study | BM-MNCs |
| 2020 | Effect of Autologous Adipose-Derived Stromal Vascular Fraction Transplantation on Endometrial Regeneration in Patients of Asherman’s Syndrome: a Pilot Study^77^ | Asherman’s syndrome | 6 patients | 4.6±0.7×10^6^ | A pilot study | Autologous Adipose-Derived Stromal Vascular Fraction |
| 2020 | Intrauterine transplantation of autologous menstrual blood stem cells increases endometrial thickness and pregnancy potential in patients with refractory intrauterine adhesion^78^ | Asherman’s syndrome | 12 patients | NA | NA | Menstrual blood-derived MSCs |
| 2021 | Unresponsive thin endometrium caused by Asherman syndrome treated with umbilical cord mesenchymal stem cells on collagen scaffolds: a pilot study^79^ | Asherman’s syndrome | 18 patients | 20×10^6^ | A pilot study | UC-MSCs embedded in collagen |
| 2021 | Can Autologous Adipose-Derived Mesenchymal Stem Cell Transplantation Improve Sexual Function in People with Sexual Functional Deficiency?^80^ | Sexual Functional Deficiency | 31 patients | 10^6^ cells/Kg of weight | Non-controlled, phase I clinical trial | Autologous adipose tissue-derived MSCs |

**References**

1. Simonson OE, Mougiakakos D, Heldring N, et al. In vivo effects of mesenchymal stromal cells in two patients with severe acute respiratory distress syndrome. *Stem cells translational medicine*. 2015;4(10):1199-1213.

2. Wilson JG, Liu KD, Zhuo H, et al. Mesenchymal stem (stromal) cells for treatment of ARDS: a phase 1 clinical trial. *The Lancet Respiratory Medicine*. 2015;3(1):24-32.

3. Matthay MA, Calfee CS, Zhuo H, et al. Treatment with allogeneic mesenchymal stromal cells for moderate to severe acute respiratory distress syndrome (START study): a randomised phase 2a safety trial. *The Lancet Respiratory Medicine*. 2019;7(2):154-162.

4. Yip H-K, Fang W-F, Li Y-C, et al. Human umbilical cord-derived mesenchymal stem cells for acute respiratory distress syndrome. *Critical care medicine*. 2020;48(5):e391-e399.

5. Bellingan G, Jacono F, Bannard-Smith J, et al. Safety and efficacy of multipotent adult progenitor cells in acute respiratory distress syndrome (MUST-ARDS): a multicentre, randomised, double-blind, placebo-controlled phase 1/2 trial. *Intensive care medicine*. 2022;48:36-44.

6. Gorman E, Shankar-Hari M, Hopkins P, et al. Repair of acute respiratory distress syndrome by stromal cell administration (REALIST) trial: a phase 1 trial. *EClinicalMedicine*. 2021;41

7. Ichikado K, Kotani T, Kondoh Y, et al. Clinical efficacy and safety of multipotent adult progenitor cells (invimestrocel) for acute respiratory distress syndrome (ARDS) caused by pneumonia: a randomized, open-label, standard therapy–controlled, phase 2 multicenter study (ONE-BRIDGE). *Stem Cell Research & Therapy*. 2023;14(1):217.

8. Chang YS, Ahn SY, Yoo HS, et al. Mesenchymal stem cells for bronchopulmonary dysplasia: phase 1 dose-escalation clinical trial. *The Journal of pediatrics*. 2014;164(5):966-972. e6.

9. Ahn SY, Chang YS, Kim JH, Sung SI, Park WS. Two-year follow-up outcomes of premature infants enrolled in the phase I trial of mesenchymal stem cells transplantation for bronchopulmonary dysplasia. *The Journal of pediatrics*. 2017;185:49-54. e2.

10. Liem NT, Anh TL, Thai TTH, Anh BV. Bone marrow mononuclear cells transplantation in treatment of established bronchopulmonary dysplasia: a case report. *The American Journal of Case Reports*. 2017;18:1090.

11. Lin H-C, Wang C-C, Chou H-W, et al. Airway delivery of bone marrow–derived mesenchymal stem cells reverses bronchopulmonary dysplasia superimposed with acute respiratory distress syndrome in an infant. *Cell Medicine*. 2018;10:2155179018759434.

12. Álvarez-Fuente M, Arruza L, Lopez-Ortego P, et al. Off-label mesenchymal stromal cell treatment in two infants with severe bronchopulmonary dysplasia: clinical course and biomarkers profile. *Cytotherapy*. 2018;20(11):1337-1344.

13. Powell SB, Silvestri JM. Safety of intratracheal administration of human umbilical cord blood derived mesenchymal stromal cells in extremely low birth weight preterm infants. *The Journal of pediatrics*. 2019;210:209-213. e2.

14. Nguyen LT, Trieu TT, Bui HT, et al. Allogeneic administration of human umbilical cord-derived mesenchymal stem/stromal cells for bronchopulmonary dysplasia: preliminary outcomes in four Vietnamese infants. *Journal of Translational Medicine*. 2020;18:1-12.

15. Ahn SY, Chang YS, Lee MH, et al. Stem cells for bronchopulmonary dysplasia in preterm infants: a randomized controlled phase II trial. *Stem cells translational medicine*. 2021;10(8):1129-1137.

16. Akduman H, Dilli D, Çakmakçı E, Çelen Yoldaş T, Öztoprak Ü, Kaya B. Mesenchymal stem cell application in a newborn with severe intraventricular hemorrhage and respiratory distress. Taylor & Francis; 2023:751-754.

17. del Cerro Marín MJ, Ormazábal IG, Gimeno-Navarro A, et al. Repeated intravenous doses of human umbilical cord-derived mesenchymal stromal cells for bronchopulmonary dysplasia: results of a phase 1 clinical trial with 2-year follow-up. *Cytotherapy*. 2024;26(6):632-640.

18. Laterre P-F, García MS, van der Poll T, et al. The safety and efficacy of stem cells for the treatment of severe community-acquired bacterial pneumonia: A randomized clinical trial. *Journal of Critical Care*. 2024;79:154446.

19. Ribeiro-Paes JT, Bilaqui A, Greco OT, et al. Unicentric study of cell therapy in chronic obstructive pulmonary disease/pulmonary emphysema. *International journal of chronic obstructive pulmonary disease*. 2011:63-71.

20. Weiss DJ, Casaburi R, Flannery R, LeRoux-Williams M, Tashkin DP. A placebo-controlled, randomized trial of mesenchymal stem cells in COPD. *Chest*. 2013;143(6):1590-1598.

21. Stessuk T, Ruiz MA, Greco OT, Bilaqui A, Ribeiro-Paes MJdO, Ribeiro-Paes JT. Phase I clinical trial of cell therapy in patients with advanced chronic obstructive pulmonary disease: follow-up of up to 3 years. *Revista Brasileira de Hematologia e Hemoterapia*. 2013;35:352-357.

22. Tzilas V, Bouros E, Fourla D, et al. Prospective phase 1 open clinical trial to study the safety of adipose derived mesenchymal stem cells (ADMSCs) in COPD and combined pulmonary fibrosis and emphysema (CPFE). Eur Respiratory Soc; 2015.

23. de Oliveira HG, Cruz FF, Antunes MA, et al. Combined bone marrow-derived mesenchymal stromal cell therapy and one-way endobronchial valve placement in patients with pulmonary emphysema: a phase I clinical trial. *Stem cells translational medicine*. 2017;6(3):962-969.

24. Comella K, Blas JAP, Ichim T, Lopez J, Limon J, Moreno RC. Autologous stromal vascular fraction in the intravenous treatment of end-stage chronic obstructive pulmonary disease: a phase I trial of safety and tolerability. *Journal of Clinical Medicine Research*. 2017;9(8):701.

25. Armitage J, Tan DB, Troedson R, et al. Mesenchymal stromal cell infusion modulates systemic immunological responses in stable COPD patients: a phase I pilot study. *European Respiratory Journal*. 2018;51(3)

26. Karaoz E, Kalemci S, Ece F. Improving effects of mesenchymal stem cells on symptoms of chronic obstructive pulmonary disease. *Bratislavske lekarske listy*. 2020;121(3):188-191.

27. Le Thi Bich P, Nguyen Thi H, Dang Ngo Chau H, et al. Allogeneic umbilical cord-derived mesenchymal stem cell transplantation for treating chronic obstructive pulmonary disease: a pilot clinical study. *Stem cell research & therapy*. 2020;11:1-14.

28. Feng Y, Huang J, Wu J, et al. Safety and feasibility of umbilical cord mesenchymal stem cells in patients with COVID‐19 pneumonia: A pilot study. *Cell Proliferation*. 2020;53(12):e12947.

29. Shu L, Niu C, Li R, et al. Treatment of severe COVID-19 with human umbilical cord mesenchymal stem cells. *Stem cell research & therapy*. 2020;11:1-11.

30. Tao J, Nie Y, Wu H, et al. Umbilical cord blood-derived mesenchymal stem cells in treating a critically ill COVID-19 patient. *The Journal of Infection in Developing Countries*. 2020;14(10):1138-1145.

31. Meng F, Xu R, Wang S, et al. Human umbilical cord-derived mesenchymal stem cell therapy in patients with COVID-19: a phase 1 clinical trial. *Signal transduction and targeted therapy*. 2020;5(1):172.

32. Hashemian S-MR, Aliannejad R, Zarrabi M, et al. Mesenchymal stem cells derived from perinatal tissues for treatment of critically ill COVID-19-induced ARDS patients: a case series. *Stem cell research & therapy*. 2021;12:1-12.

33. Zhang Y, Ding J, Ren S, et al. Intravenous infusion of human umbilical cord Wharton’s jelly-derived mesenchymal stem cells as a potential treatment for patients with COVID-19 pneumonia. *Stem cell research & therapy*. 2020;11:1-6.

34. Shi L, Yuan X, Yao W, et al. Human mesenchymal stem cells treatment for severe COVID-19: 1-year follow-up results of a randomized, double-blind, placebo-controlled trial. *EBioMedicine*. 2022;75

35. Guo Z, Chen Y, Luo X, He X, Zhang Y, Wang J. Administration of umbilical cord mesenchymal stem cells in patients with severe COVID-19 pneumonia. *Critical care*. 2020;24:1-3.

36. Zhu R, Yan T, Feng Y, et al. Mesenchymal stem cell treatment improves outcome of COVID-19 patients via multiple immunomodulatory mechanisms. *Cell research*. 2021;31(12):1244-1262.

37. Shi L, Huang H, Lu X, et al. Effect of human umbilical cord-derived mesenchymal stem cells on lung damage in severe COVID-19 patients: a randomized, double-blind, placebo-controlled phase 2 trial. *Signal transduction and targeted therapy*. 2021;6(1):58.

38. O. Ercelen N, Pekkoc-Uyanik KC, Alpaydin N, Gulay GR, Simsek M. Clinical experience on umbilical cord mesenchymal stem cell treatment in 210 severe and critical COVID-19 cases in Turkey. *Stem Cell Reviews and Reports*. 2021;17:1917-1925.

39. Saleh M, Vaezi AA, Aliannejad R, et al. Cell therapy in patients with COVID-19 using Wharton’s jelly mesenchymal stem cells: a phase 1 clinical trial. *Stem cell research & therapy*. 2021;12:1-13.

40. Adas G, Çukurova Z, Yasar KK, et al. The systematic effect of mesenchymal stem cell therapy in critical COVID-19 patients: a prospective double controlled trial. *Cell Transplantation*. 2021;30:09636897211024942.

41. Li T-T, Zhang B, Fang H, et al. Human mesenchymal stem cell therapy in severe COVID-19 patients: 2-year follow-up results of a randomized, double-blind, placebo-controlled trial. *EBioMedicine*. 2023;92

42. Papadopoulou A, Karavalakis G, Papadopoulou E, et al. SARS-CoV-2-specific T cell therapy for severe COVID-19: a randomized phase 1/2 trial. *Nature Medicine*. 2023;29(8):2019-2029.

43. Averyanov A, Koroleva I, Konoplyannikov M, et al. First-in-human high-cumulative-dose stem cell therapy in idiopathic pulmonary fibrosis with rapid lung function decline. *Stem cells translational medicine*. 2020;9(1):6-16.

44. Stolk J, Broekman W, Mauad T, et al. A phase I study for intravenous autologous mesenchymal stromal cell administration to patients with severe emphysema. *QJM: An International Journal of Medicine*. 2016;109(5):331-336.

45. Perin EC, Sanz-Ruiz R, Sánchez PL, et al. Adipose-derived regenerative cells in patients with ischemic cardiomyopathy: The PRECISE Trial. *American heart journal*. 2014;168(1):88-95. e2.

46. Ulus AT, Mungan C, Kurtoglu M, et al. Intramyocardial transplantation of umbilical cord mesenchymal stromal cells in chronic ischemic cardiomyopathy: a controlled, randomized clinical trial (HUC-HEART Trial). *International journal of stem cells*. 2020;13(3):364-376.

47. Qayyum AA, Mathiasen AB, Mygind ND, et al. Adipose‐derived stromal cells for treatment of patients with chronic ischemic heart disease (MyStromalCell trial): a randomized placebo‐controlled study. *Stem Cells International*. 2017;2017(1):5237063.

48. He X, Wang Q, Zhao Y, et al. Effect of intramyocardial grafting collagen scaffold with mesenchymal stromal cells in patients with chronic ischemic heart disease: a randomized clinical trial. JAMA Netw Open. 2020; 3: e2016236. *Chinese Journal of Tissue Engineering Research www CJTER com*. 2020;

49. Henry TD, Pepine CJ, Lambert CR, et al. The Athena trials: Autologous adipose‐derived regenerative cells for refractory chronic myocardial ischemia with left ventricular dysfunction. *Catheterization and Cardiovascular Interventions*. 2017;89(2):169-177.

50. Shirbaghaee Z, Heidari Keshel S, Rasouli M, et al. Report of a phase 1 clinical trial for safety assessment of human placental mesenchymal stem cells therapy in patients with critical limb ischemia (CLI). *Stem Cell Research & Therapy*. 2023;14(1):174.

51. Bartunek J, Behfar A, Dolatabadi D, et al. Cardiopoietic stem cell therapy in heart failure: the C-CURE (Cardiopoietic stem Cell therapy in heart failURE) multicenter randomized trial with lineage-specified biologics. *Journal of the American College of Cardiology*. 2013;61(23):2329-2338.

52. Bartolucci J, Verdugo FJ, González PL, et al. Safety and efficacy of the intravenous infusion of umbilical cord mesenchymal stem cells in patients with heart failure: a phase 1/2 randomized controlled trial (RIMECARD trial [randomized clinical trial of intravenous infusion umbilical cord mesenchymal stem cells on cardiopathy]). *Circulation research*. 2017;121(10):1192-1204.

53. Florea V, Rieger AC, DiFede DL, et al. Dose comparison study of allogeneic mesenchymal stem cells in patients with ischemic cardiomyopathy (the TRIDENT study). *Circulation research*. 2017;121(11):1279-1290.

54. Hare JM, Fishman JE, Gerstenblith G, et al. Comparison of allogeneic vs autologous bone marrow–derived mesenchymal stem cells delivered by transendocardial injection in patients with ischemic cardiomyopathy: the POSEIDON randomized trial. *Jama*. 2012;308(22):2369-2379.

55. Heldman AW, DiFede DL, Fishman JE, et al. Transendocardial mesenchymal stem cells and mononuclear bone marrow cells for ischemic cardiomyopathy: the TAC-HFT randomized trial. *Jama*. 2014;311(1):62-73.

56. Kastrup J, Haack-Sørensen M, Juhl M, et al. Cryopreserved off-the-shelf allogeneic adipose-derived stromal cells for therapy in patients with ischemic heart disease and heart failure—a safety study. *Stem Cells Translational Medicine*. 2017;6(11):1963-1971.

57. Bartunek J, Terzic A, Davison BA, et al. Cardiopoietic cell therapy for advanced ischaemic heart failure: results at 39 weeks of the prospective, randomized, double blind, sham-controlled CHART-1 clinical trial. *European heart journal*. 2017;38(9):648-660.

58. Bolli R, Mitrani RD, Hare JM, et al. A Phase II study of autologous mesenchymal stromal cells and c‐kit positive cardiac cells, alone or in combination, in patients with ischaemic heart failure: the CCTRN CONCERT‐HF trial. *European journal of heart failure*. 2021;23(4):661-674.

59. Hare JM, DiFede DL, Rieger AC, et al. Randomized comparison of allogeneic versus autologous mesenchymal stem cells for nonischemic dilated cardiomyopathy: POSEIDON-DCM trial. *Journal of the American College of Cardiology*. 2017;69(5):526-537.

60. Qayyum AA, Mathiasen AB, Helqvist S, et al. Autologous adipose-derived stromal cell treatment for patients with refractory angina (MyStromalCell Trial): 3-years follow-up results. *Journal of Translational Medicine*. 2019;17:1-9.

61. Mathiasen AB, Qayyum AA, Jørgensen E, et al. Bone marrow-derived mesenchymal stromal cell treatment in patients with severe ischaemic heart failure: a randomized placebo-controlled trial (MSC-HF trial). *European heart journal*. 2015;36(27):1744-1753.

62. Kim K-I, Lee MC, Lee JH, et al. Clinical efficacy and safety of the intra-articular injection of autologous adipose-derived mesenchymal stem cells for knee osteoarthritis: a phase III, randomized, double-blind, placebo-controlled trial. *The American journal of sports medicine*. 2023;51(9):2243-2253.

63. Chen C-F, Chen Y-C, Fu Y-S, et al. Safety and Tolerability of Intra-Articular Injection of Adipose-Derived Mesenchymal Stem Cells GXCPC1 in 11 Subjects With Knee Osteoarthritis: A Nonrandomized Pilot Study Without a Control Arm. *Cell Transplantation*. 2024;33:09636897231221882.

64. Matas J, García C, Poblete D, et al. A phase I dose-escalation clinical trial to assess the safety and efficacy of umbilical cord-derived mesenchymal stromal cells in knee osteoarthritis. *Stem Cells Translational Medicine*. 2024;13(3):193-203.

65. Sharan J, Barmada A, Prodromos C, Candido K. First human report of relief of lumbar and cervical discogenic and arthritic back pain after epidural and facet joint mesenchymal stem cell injection: a case report. *Current stem cell research & therapy*. 2023;18(7):1013-1015.

66. Sadri B, Hassanzadeh M, Bagherifard A, et al. Cartilage regeneration and inflammation modulation in knee osteoarthritis following injection of allogeneic adipose-derived mesenchymal stromal cells: a phase II, triple-blinded, placebo controlled, randomized trial. *Stem Cell Research & Therapy*. 2023;14(1):162.

67. Zhao X, Ruan J, Li J, Dai C, Pei M, Zhou Y. Three-dimensional texture analyses of multi-quantitative relaxation time maps for evaluating cartilage repair with the treatment of allogeneic human adipose-derived mesenchymal progenitor cells. *Magnetic Resonance Imaging*. 2024;110:7-16.

68. Edessy M, Hosni HN, Shady Y, Waf Y, Bakr S, Kamel M. Autologous Stem Cells Therapy, The First Baby of Idiopathic Premature Ovarian Failure. *Acta Medica International*. 2016 2016;3(1):19-19. doi:10.5530/ami.2016.1.7

69. Ding L, Yan G, Wang B, et al. Transplantation of UC-MSCs on collagen scaffold activates follicles in dormant ovaries of POF patients with long history of infertility. *Sci China Life Sci*. 2018/12// 2018;61(12):1554-1565. doi:10.1007/s11427-017-9272-2

70. Gupta S, Lodha P, Karthick M, Tandulwadkar S. Role of autologous bone marrow-derived stem cell therapy for follicular recruitment in premature ovarian insufficiency: Review of literature and a case report of world's first baby with ovarian autologous stem cell therapy in a perimenopausal woman of age 45 year. *J Hum Reprod Sci*. 2018 2018;11(2):125. doi:10.4103/jhrs.JHRS_57_18

71. Herraiz S, Romeu M, Buigues A, et al. Autologous stem cell ovarian transplantation to increase reproductive potential in patients who are poor responders. *Fertility and Sterility*. 2018/08// 2018;110(3):496-505.e1. doi:10.1016/j.fertnstert.2018.04.025

72. Igboeli P, El Andaloussi A, Sheikh U, et al. Intraovarian injection of autologous human mesenchymal stem cells increases estrogen production and reduces menopausal symptoms in women with premature ovarian failure: two case reports and a review of the literature. *J Med Case Reports*. 2020/12// 2020;14(1):108. doi:10.1186/s13256-020-02426-5

73. Yan L, Wu Y, Li L, et al. Clinical analysis of human umbilical cord mesenchymal stem cell allotransplantation in patients with premature ovarian insufficiency. *Cell Prolif*. 2020/12// 2020;53(12)doi:10.1111/cpr.12938

74. Mashayekhi M, Mirzadeh E, Chekini Z, et al. Evaluation of safety, feasibility and efficacy of intra-ovarian transplantation of autologous adipose derived mesenchymal stromal cells in idiopathic premature ovarian failure patients: non-randomized clinical trial, phase I, first in human. *Journal of Ovarian Research*. 2021/12// 2021;14(1):5-5. doi:10.1186/s13048-020-00743-3

75. Cao Y, Sun H, Zhu H, et al. Allogeneic cell therapy using umbilical cord MSCs on collagen scaffolds for patients with recurrent uterine adhesion: a phase I clinical trial. *Stem Cell Res Ther*. 2018/12// 2018;9(1):192. doi:10.1186/s13287-018-0904-3

76. Singh N, Shekhar B, Mohanty S, Kumar S, Seth T, Girish B. Autologous bone marrow-derived stem cell therapy for Asherman's syndrome and endometrial atrophy: A 5-year follow-up study. *J Hum Reprod Sci*. 2020 2020;13(1):31. doi:10.4103/jhrs.JHRS_64_19

77. Lee SY, Shin JE, Kwon H, Choi DH, Kim JH. Effect of Autologous Adipose-Derived Stromal Vascular Fraction Transplantation on Endometrial Regeneration in Patients of Asherman’s Syndrome: a Pilot Study. *Reproductive Sciences*. 2020/02// 2020;27(2):561-568. doi:10.1007/s43032-019-00055-y

78. Ma H, Liu M, Li Y, et al. Intrauterine transplantation of autologous menstrual blood stem cells increases endometrial thickness and pregnancy potential in patients with refractory intrauterine adhesion. *J Obstet Gynaecol Res*. 2020/11// 2020;46(11):2347-2355. doi:10.1111/jog.14449

79. Zhang Y, Shi L, Lin X, et al. Unresponsive thin endometrium caused by Asherman syndrome treated with umbilical cord mesenchymal stem cells on collagen scaffolds: a pilot study. *Stem Cell Res Ther*. 2021/12// 2021;12(1):420. doi:10.1186/s13287-021-02499-z

80. Nguyen Thanh L, Dam PTM, Nguyen HP, et al. Can Autologous Adipose-Derived Mesenchymal Stem Cell Transplantation Improve Sexual Function in People with Sexual Functional Deficiency? *Stem Cell Rev Rep*. Dec 2021;17(6):2153-2163. doi:10.1007/s12015-021-10196-w
